# Sex-dependent dynamics of metabolism in primary mouse hepatocytes

**DOI:** 10.1007/s00204-021-03118-9

**Published:** 2021-07-09

**Authors:** Luise Hochmuth, Christiane Körner, Fritzi Ott, Daniela Volke, Kaja Blagotinšek Cokan, Peter Juvan, Mario Brosch, Ute Hofmann, Ralf Hoffmann, Damjana Rozman, Thomas Berg, Madlen Matz-Soja

**Affiliations:** 1 Faculty of Medicine, Rudolf-Schönheimer-Institute of Biochemistry, Leipzig University, Leipzig, Germany; 2 Center for Biotechnology and Biomedicine, Leipzig University, Leipzig, Germany; 3 Centre for Functional Genomics and Bio-Chips, Institute of Biochemistry and Molecular Genetics, Faculty of Medicine, University of Ljubljana, Ljubljana, Slovenia; 4 Department of Medicine I, Gastroenterology and Hepatology, University Hospital Carl-Gustav-Carus, Technische Universität Dresden (TU Dresden), Dresden, Germany; 5 Dr. Margarete Fischer-Bosch Institute of Clinical Pharmacology, University of Tübingen, Stuttgart, Germany; 6 Division of Hepatology, Clinic and Polyclinic for Oncology, Infectious Diseases, and Pneumology, University Hospital Leipzig, Gastroenterology, HepatologyLeipzig, Germany

**Keywords:** Liver, Hepatocytes, Sexual dimorphism, Lipid metabolism, Cytochrome P450, Drug metabolism

## Abstract

The liver is one of the most sexually dimorphic organs. The hepatic metabolic pathways that are subject to sexual dimorphism include xenobiotic, amino acid and lipid metabolism. Non-alcoholic fatty liver disease and hepatocellular carcinoma are among diseases with sex-dependent prevalence, progression and outcome. Although male and female livers differ in their abilities to metabolize foreign compounds, including drugs, sex-dependent treatment and pharmacological dynamics are rarely applied in all relevant cases. Therefore, it is important to consider hepatic sexual dimorphism when developing new treatment strategies and to understand the underlying mechanisms in model systems. We isolated primary hepatocytes from male and female C57BL6/N mice and examined the sex-dependent transcriptome, proteome and extracellular metabolome parameters in the course of culturing them for 96 h. The sex-specific gene expression of the general xenobiotic pathway altered and the female-specific expression of *Cyp2b13* and *Cyp2b9* was significantly reduced during culture. Sex-dependent differences of several signaling pathways increased, including genes related to serotonin and melatonin degradation. Furthermore, the ratios of male and female gene expression were inversed for other pathways, such as amino acid degradation, beta-oxidation, androgen signaling and hepatic steatosis. Because the primary hepatocytes were cultivated without the influence of known regulators of sexual dimorphism, these results suggest currently unknown modulatory mechanisms of sexual dimorphism in vitro. The large sex-dependent differences in the regulation and dynamics of drug metabolism observed during cultivation can have an immense influence on the evaluation of pharmacodynamic processes when conducting initial preclinical trials to investigate potential new drugs.

**Supplementary Information:**

The online version contains supplementary material available at 10.1007/s00204-021-03118-9.

## Introduction

Over the past decade, medical research has focused on the development of patient-oriented therapies. Different approaches, such as personalized, stratified and precision medicine, based on biomedical investigations with large amounts of data, have been discussed (Erikainen and Chan 2019). Although much investment has been made in researching these patient-oriented approaches, the long-known differences between male and female metabolic and pathological processes are still poorly understood. However, sexual dimorphism plays an important role, especially in liver metabolism. The primary regulators of hepatic sexual dimorphism are testosterone and estradiol signaling as well as the sex-dependent expression of growth hormone (GH) (Waxman and O'Connor 2006; Zheng et al. 2018). While female mice exhibit continuous low-level secretion of GH, the secretion of GH in males is pulsatile (Waxman and O'Connor 2006). The release of GH leads to the phosphorylation, activation and deoxyribonucleic acid (DNA) binding of signal transducer and activator of transcription 5b (STAT5b), which exhibits the same pulsatile activity pattern in males (Waxman and O'Connor 2006; Conforto et al. 2012). GH is secreted by the pituitary gland and is regulated through gonadal sex hormones (Meinhardt and Ho 2007). However, other factors also influence GH-STAT5 signaling, such as the transcription factors hepatocyte nuclear factor 4 alpha (HNF4a), B-cell CLL/lymphoma 6 (BCL6) and zinc fingers and homeoboxes 2 (ZHX2) (Laz et al. 2007; Meyer et al. 2009; Creasy et al. 2016). There is a cross-talk between GH and steroid hormone regulation, which allowed the construction of mathematical models of female and male human hepatocytes (Cvitanović Tomaš et al. 2018; Cvitanović et al. 2017), underlining the need to treat both sexes separately.

Important signaling pathways influenced by hepatic sexual dimorphism include amino acid (AA), lipid, drug and xenobiotic metabolism, which is most clearly reflected in the clinic. Several liver diseases related to disturbed lipid metabolism, such as non-alcoholic fatty liver disease (NAFLD) and hepatocellular carcinoma (HCC), show sex dimorphic prevalence. Men are more likely to contract NAFLD than premenopausal women (Lonardo et al. 2019). This difference, however, disappears after female menopause, revealing the impact of sex hormones on NAFLD (DiStefano 2020). Furthermore, HCC is more likely to affect men than women (Wu et al. 2019). Again, the impact of sex hormones is seen after menopause, when the incidence of HCC rises also in women (Ruggieri et al. 2010; Kohi 2016). In addition to the different frequencies of liver diseases, males and females also differ with respect to the metabolism of endobiotics and xenobiotics, including drugs. Reasons for these differences include the sex-specific expression of genes from hepatic signaling pathways and the downstream metabolic genes (Cokan et al. 2020), like the genes of the cytochrome P450 (*Cyp*) superfamily that are, directly or indirectly, regulated by GH or sex hormones (Shapiro et al. 1995).

Despite the large differences in liver metabolism between the sexes (Rando and Wahli 2011; Lorbek et al. 2013), this is not yet sufficiently applied in medical practice (Wilson and Buetow 2020; Natri et al. 2019). Already during the development of new drugs, hepatocyte cell culture systems are used for hepatotoxicity risk assessment (Gómez-Lechón et al. 2003) and sex-specific characteristics need to be considered. Therefore, we investigated the dynamics of the transcriptome, proteome and extracellular metabolome in cultured primary hepatocytes from male and female mice during four consecutive days.

## Materials and methods

### Maintaining and feeding of the mice

The C57BL/6 N mice used in this study were maintained according to European (Directive 2010/63/EU) and German guidelines for the care and safe use of experimental animals. The animal experiments were approved by the Landesdirektion Sachsen (permission numbers: TVV44/16; T04/14). Further details are given in Online Resource 1.

### The isolation and culture of primary mouse hepatocytes

Primary hepatocytes from male and female C57BL/6 N mice were isolated using a collagenase perfusion technique and subsequently cleared from other liver cells by differential centrifugation as previously described (Gebhardt et al. 2003; Matz-Soja et al. 2014). A detailed description of culture conditions is given in Online Resource 1. Samples for proteomic, transcriptomic, extracellular metabolomic and qPCR analyses were collected after 24, 48, 72 and 96 h (Online Resource 1 Fig. S1).

### Microarray-based gene expression analysis

To analyze the sex-specific differences RNA was isolated from primary hepatocytes of five mice per sex using the ReliaPrep RNA Miniprep Kit (Promega GmbH, Walldorf, Germany) according to the manufacturer’s instructions. Subsequently, sample preparation and Clariom S Assays for mice (Thermo Fisher Scientific Inc., Waltham, USA) were conducted according to the manufacturer’s instructions. Further specifications of data analysis are described in the Online Resource 1, raw and normalized data were deposited with GEO under the accession number GSE166969, the Tables S1–S53 (Online Resource 2) include detailed results of activation Z-score analysis.

### Proteome Analysis

Primary mouse hepatocytes (1.8 × 10^6^) of five mice per sex were used for the proteome analysis. The detailed method description is given in Online Resource 1, metadata of proteome analysis are provided in Online Resource 3 and Tables S1-S53 (Online Resource 2) include detailed results of activation Z-score analysis.

### RNA isolation and quantitative real-time PCR (qPCR)

RNA isolation and qPCR procedures were performed as previously described (Spormann et al. 2020). Five male and four female mice were used. The 14–3-3 protein zeta/delta (*Ywhaz*) was amplified as a reference gene. The primer sequences are shown in Online Resource 1 Table S54. The results are presented as the means of biological replicates ± standard error of the mean (SEM). Statistical significance was calculated with two-way analysis of variance (ANOVA) using GraphPad Prism 7 software (GraphPad Software). P values are indicated as *p* < 0.05 (*), *p* < 0.01 (**) and *p* < 0.001 (***).

### Extracellular metabolome analysis

For extracellular metabolome analysis, we used the medium supernatants of primary hepatocytes from five male and five female mice. Proteinogenic AA, ornithine, urea, pyruvate, acetoacetate, hydroxybutyrate, fumarate, α-ketoglutarate, malate, and citrate were determined by GC–MS analysis as previously described (Hofmann et al. 2008; Maier et al. 2010). The quantification of lactate and bile acids is specified in Online Resource 1. The metadata of metabolome analysis are provided in Online Resource 4.

## Results

**Fig. 1:**
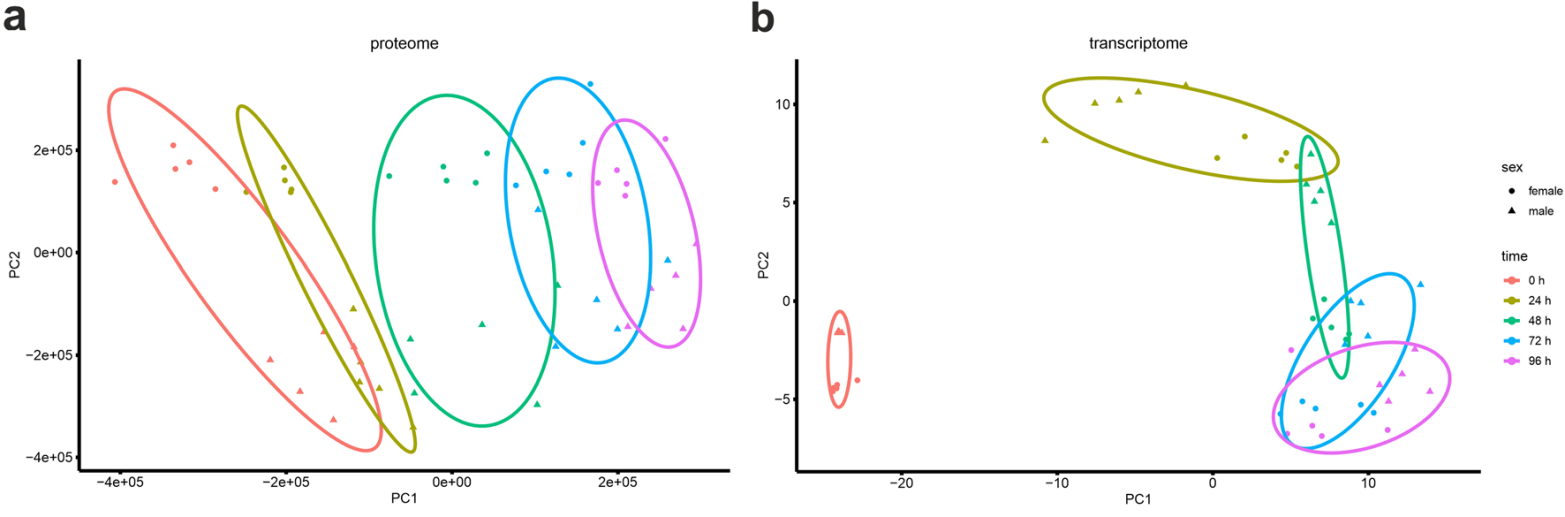
PCA plots of the measured. **a** Proteins during proteome analysis and **b** their corresponding genes. N = 5

To examine sex-specific dimorphism in primary mouse hepatocytes and its alterations during cultivation, we isolated hepatocytes from C57BL/6 N mice of both sexes and cultured them without the addition of sex hormones or growth factors (GF) for up to 96 h (Online Resource 1 Fig. S1). The principal component analysis (PCA) plots, based on all measured proteins and their corresponding genes, exhibited distinct differences in the dynamics of sex-specific changes during the culture of hepatocytes (Fig. 1). At all time points, there is a clear separation between the expression of female (circle) and male (triangle) proteins (Fig. 1a) and genes (Fig. 1b), although the differences between sexes became more diffuse in the course of cultivation. At the transcriptome level, different time points show great shifts in gene expression, with the most important shift after the 1st day of cultivation (24 h). During further primary hepatocyte culture, the gene expression pattern almost returned to the initial level in the vertical direction, while it shifted in the horizontal direction (Fig. 1b). In contrast to gene expression, the proteome changed only slightly during the different time points of cultivation of primary hepatocytes (Fig. 1a). Similar to the transcription level, the protein translation pattern shifted in the horizontal direction during culture. However, at the protein level, the clusters between male and female hepatocytes are more distinct. In the course of cultivation, the protein expression of male hepatocytes approached that of female hepatocytes, suggesting feminization of the proteome (Fig. 1a).

### sex-dependent gene expression of androgen signaling changes after 48 h of hepatocyte culture

**Fig. 2:**
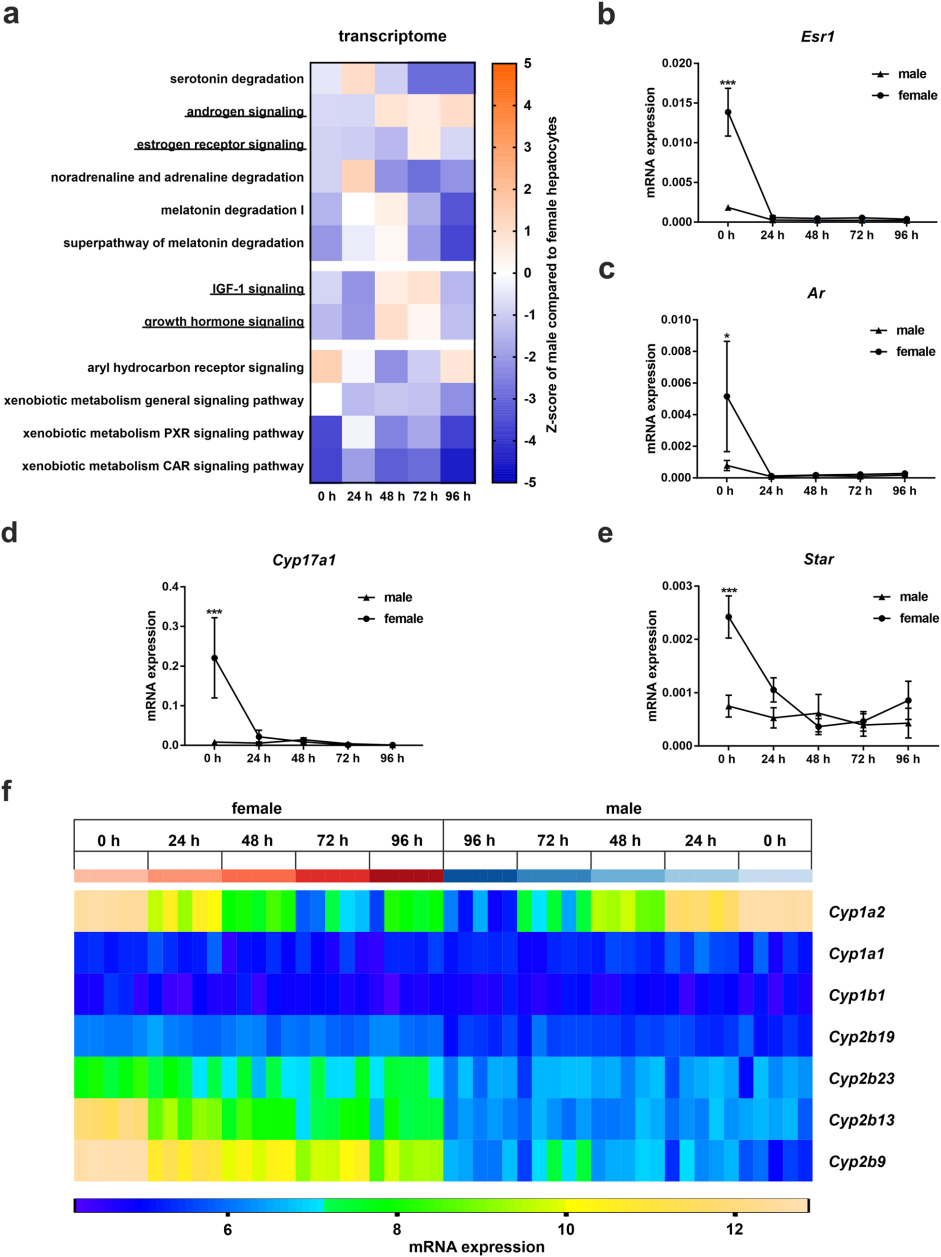
Expression of genes involved in hormone, growth factor and xenobiotic metabolism during cultivation of primary murine male and female hepatocytes. **a** Activation Z-score analysis of RNA array data of primary hepatocytes from male and female mice cultured for 96 h. Colors show the activation Z-score of male compared to female hepatocytes. Activation Z-score was calculated using IPA software with a p value cut-off of 0.05. **b**–**e** qPCR analyses of genes related to steroid metabolism were normalized on Y*whaz*. Error bars show SEM. Significance was calculated with two-way analysis of variance (ANOVA). Stars show the significance between hepatocytes from male and female mice at a certain time point *p ≤ 0.05, ***p ≤ 0.001. **f** Gene array analysis of cytochrome P450 family members involved in xenobiotic metabolism. Depicted are gene expression intensities. *N* = 4–5

Sex-specific differences strongly depend upon hormone stimulation in vivo*.* Although it is possible to add sexual signaling molecules in vitro, it is extremely difficult to mimic the combinations and oscillations given in vivo. Therefore, we examined whether sex differences in expression are maintained during primary mouse hepatocyte culture and how they affect other aspects of hepatic metabolism. We compared male and female gene expression over time using IPA software for inference of literature-based cause–effect relationships and gene set enrichment (Krämer et al. 2014). We compared predictions of the behavior of all metabolic processes based on the abundance of individual genes measured in the primary hepatocytes and focused on pathways associated with hormone, GF and xenobiotic metabolism (Fig. 2a).

Our results showed that the sex-specific pathway activity related to androgen signaling tends to alter during culture, switching from less to higher activity in male hepatocytes compared to female hepatocytes after 48 h of cultivation (Fig. 2a upper panel, Online Resource 2 Table S2). Besides, IPA predicted that the impact of androgen signaling on sex determination was higher in the male hepatocytes at 0 h and lower at 24–96 h compared to that of the female hepatocytes (Online Resource 5 interactive pdf—androgen signaling). In addition, the activity of estrogen receptor signaling showed the trend to remain a lower activity in male than in female hepatocytes over time, except at 72 h (Fig. 2a upper panel, Online Resource 2 Table S3). Other hormone pathways exhibited an even stronger inhibition in male hepatocytes compared to female hepatocytes with various exceptions at different time points (Fig. 2a upper panel, Online Resource 2 Tables S1, S4–6).

Another important regulator of sex-specific gene expression is GH (Waxman and O'Connor 2006). Transcription related to signaling of the insulin-like GF (IGF)-1 and GH revealed a lower activity of these pathways in male hepatocytes than in female hepatocytes from 0 to 24 h, shifting to higher activity from 48–72 h and back to reduced activity levels at 96 h (Fig. 2a middle panel, Online Resource 2 Tables S7–8). However, STAT5 signaling, which is the primary regulator of GH-dependent sexual dimorphism (Udy et al. 1997), was lower at 0 h and upregulated from 24 to 96 h in male hepatocytes compared to female hepatocytes (Online Resource 5 interactive pdf—GH signaling).

qPCR measurements of certain genes involved in steroid metabolism exposed that the primary effect of sexual dimorphism is lost after 24 h of hepatocyte culture (Fig. 2b–e). In the case of estrogen receptor 1 (*Esr1*), androgen receptor (*Ar*), steroidogenic acute regulatory protein (*Star*) and *Cyp17a1*, the female gene expression pattern seemed to be masculinized during culture.

### The sex-specific gene expression patterns of *Cyp1a2* and of female-specific *Cyp2b13* and *Cyp2b9* are altered during hepatocyte culture

Xenobiotic metabolism is one of the most important aspects of hepatic functions that are influenced by sexual dimorphism due to its impact on drug efficacy during medical treatments. Similar to hormone and GF metabolism, gene expression related to xenobiotic pathways changed during primary hepatocyte culture. Interestingly, the activation Z-score of aryl hydrocarbon receptor and xenobiotic pregnane X receptor (PXR) signaling after 96 h of culture was more similar to that in vivo than to any of the other cultivation time points examined in this study (Fig. 2a lower panel, Online Resource 2 Tables S9, S11). In contrast to that, the activity of the general xenobiotic pathway in male hepatocytes compared to female hepatocytes decreased during the culture period ending in significant inhibition of this pathway at 96 h (activation Z-score: 0 to -2.2) (Fig. 2a lower panel, Online Resource 2 Table S10). An examination of genes related to the *Cyp* family revealed that the gene expression of *Cyp1a2* was significantly reduced during cell culture in male and female hepatocytes. Furthermore, it is striking that female-specific mRNA expression of *Cyp2b13* and *Cyp2b9* was significantly reduced during cell culture, while the expression of *Cyp1a1*, *Cyp1b1*, *Cyp2b19* and *Cyp2b23* remained relatively constant (Fig. 2f).

### Sex-specific gene expression pattern of fatty acid beta-oxidation and bile acid synthesis changes after 48 h of cell culture

**Fig. 3:**
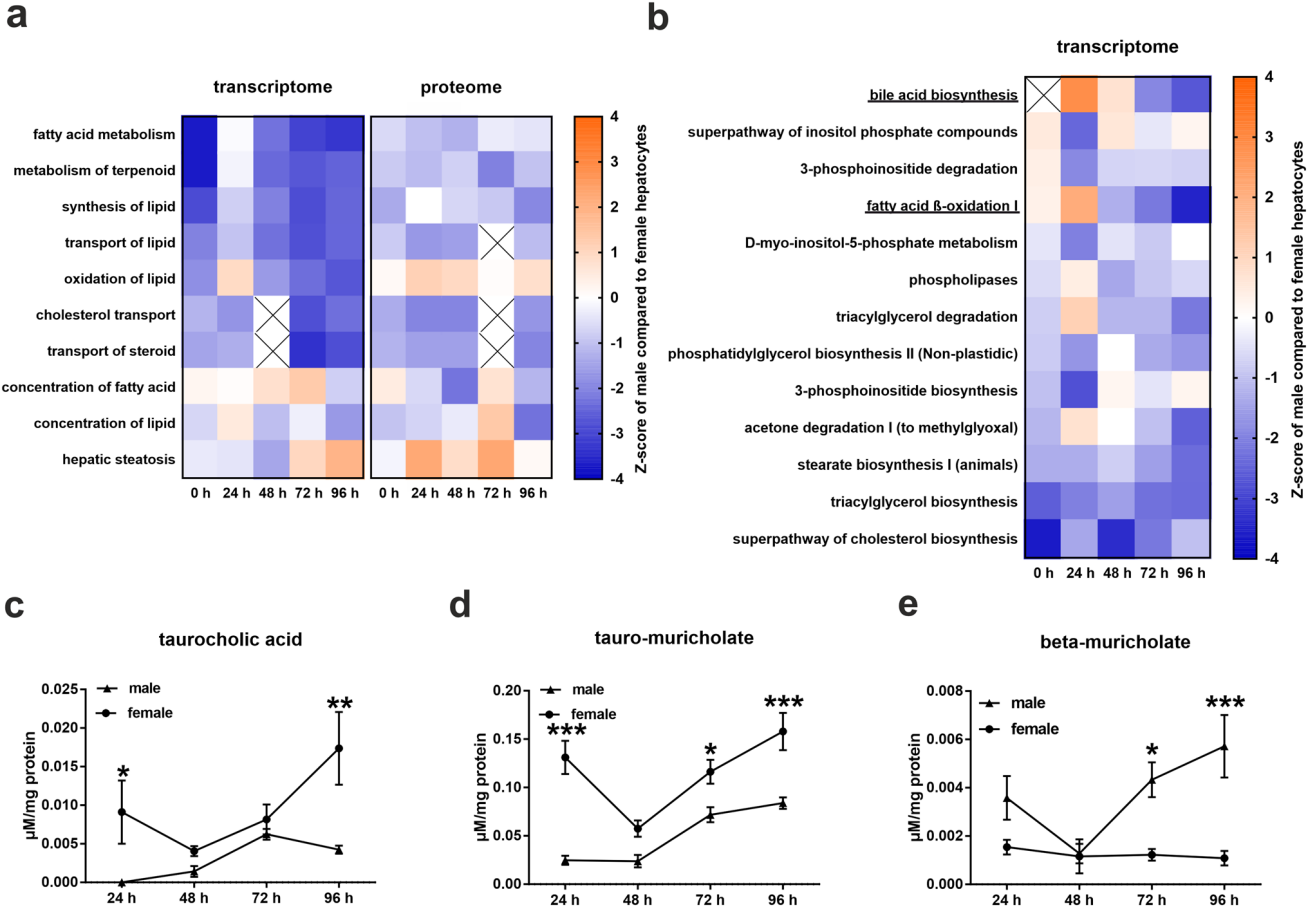
Alteration in sex-specific hepatic lipid metabolism during cell culture. **a**, **b** Activation Z-score analysis of the RNA array and proteome analyses related to lipid metabolism. Colors show the activation Z-score of male compared to female hepatocytes. Activation Z-score was calculated using IPA software with a *p* value cut-off of 0.05. Crossed squares mark non-significant values. **c**–**e** Extracellular metabolome analysis. Error bars show SEM. Significance was calculated with two-way-ANOVA. Stars show the significance between hepatocytes from male and female mice at a certain time point. *p ≤ 0.05, **p ≤ 0.01, ***p ≤ 0.001. *N* = 5

Hepatic lipid metabolism is highly influenced by steroid action (Della Torre et al. 2016). Our data showed that many of the genes and proteins differentially regulated between male and female hepatocytes are related to lipid metabolism-associated biofunctions and diseases (Fig. 3a). Most of these pathways (e.g., fatty acid and terpenoid metabolism, lipid synthesis) exhibited a lower activity in male compared to female hepatocytes (Online Resource 2 Tables S13-S32). In contrast to the gene expression pattern, the proteome revealed a higher lipid oxidation activity in male compared to female hepatocytes (Online Resource 2 Table S27). Interestingly, the expression pattern of genes related to hepatic steatosis changed from lower to higher activity in male compared to female hepatocytes after 72 h, while the sex ratio on protein level fluctuated in an oscillation-like manner, with peaks at 24 and 72 h (Online Resource 2 Tables S22, S32).

Furthermore, we investigated the gene expression of canonical lipid pathways. Our results revealed that the activity pattern of bile acid synthesis and beta-oxidation is inverse to androgen signaling, significantly switching from higher to lower activity in male hepatocytes compared to female hepatocytes after 72 h and 48 h, respectively (Fig. 3b, Online Resource 2 Tables S33, S36). In addition, the data exhibited a discrepancy between the activity of bile acid synthesis, which was higher in male hepatocytes from 24 to 48 h, and the levels of taurocholic acid and tauro-muricholate in the supernatant of the hepatocytes, which were significantly higher in the medium of female hepatocytes after 24 h of culture (Fig. 3c, d). After 48 h of culture, the levels of these two bile acids and beta-muricholate were similar in the medium of hepatocytes of both sexes, while they were significantly lower after 72 h (only beta-muricholate and tauro-muricholate) and 96 h (Fig. 3c–e). The majority of the other pathways related to lipid metabolism, which differed in activity between male and female hepatocytes, showed predominantly lower activity in male hepatocytes throughout cell culture with slight variations. It is striking that the 24 h values often deviate strongly from the other time points, either due to an amplification or a reversal of the sex-specific difference.

### Female hepatocytes consume more amino acids than male hepatocytes

**Fig. 4:**
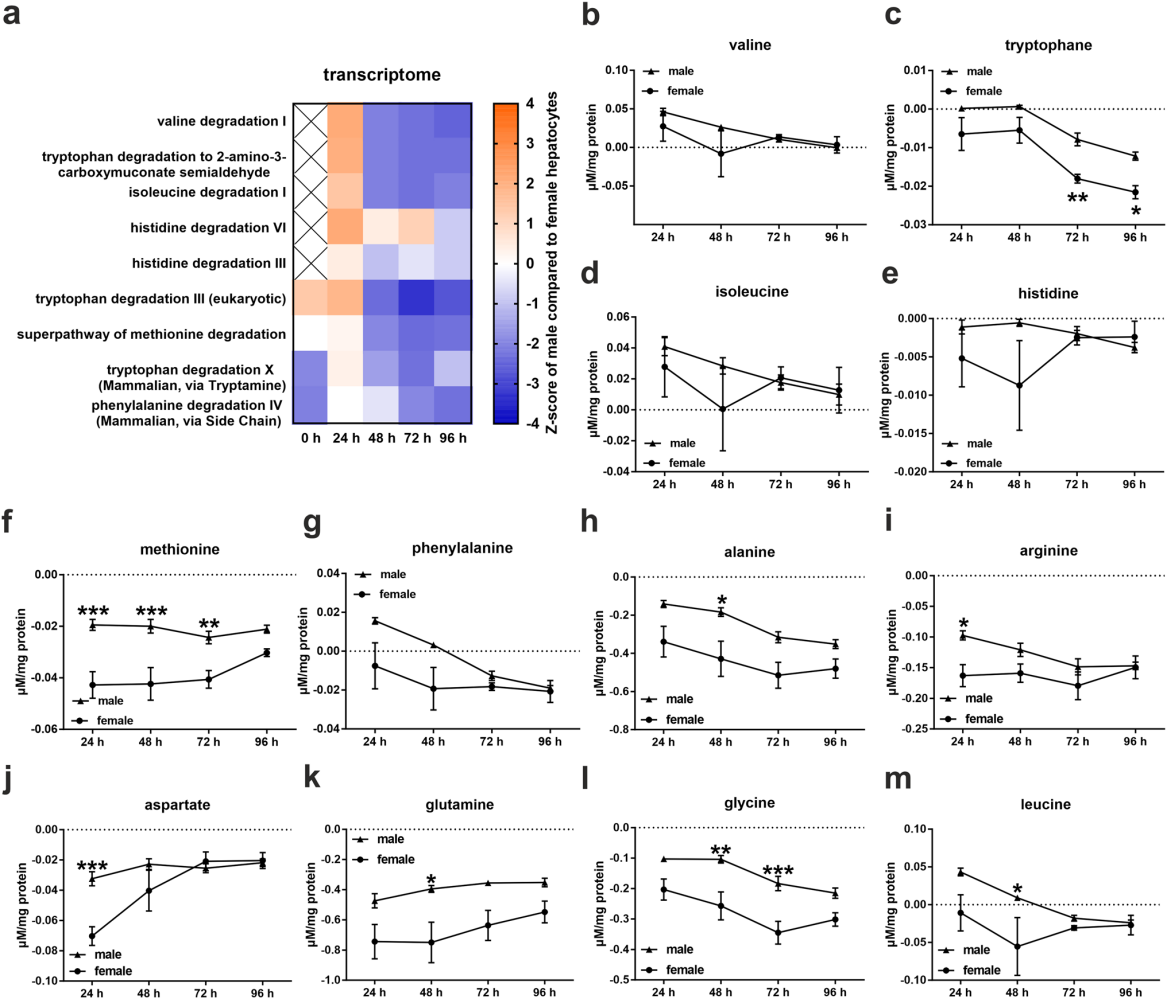
Sex-specific amino acid (AA) consumption of hepatocytes. **a** Activation Z-score analysis of the RNA array of genes related to AA degradation. Colors show the activation Z-score of male compared to female hepatocytes. Activation Z-score was calculated using IPA software with a p value cut-off of 0.05. **b-m** Extracellular metabolome analysis. Error bars show SEM. Significance was calculated with two-way ANOVA. Stars show the significance between female and male mouse hepatocytes. *P ≤ 0.05, **P ≤ 0.01, ***P ≤ 0.001. *N* = 5

It is known that hepatic transcription of genes related to AA metabolism is lower in males than in females (Della Torre et al. 2018). We found the same result for several AA degradation pathways between 48 and 96 h of culture, although most of them exhibited an inverse expression pattern, with higher activity in the male hepatocytes than in the female hepatocytes at 24 h (Fig. 4a, Online Resource 2 Tables S45-S51). The metabolomic analysis demonstrated that the medium of the male hepatocytes contained increased AA levels throughout the cell culture period, suggesting that female hepatocytes use up more AA, corresponding to the higher activity in AA degradation in the females (Fig. 4b–m, Online Resource 1 Fig. S2a-g). Only the consumption of serine was significantly lower in the medium of female hepatocytes at 96 h of cell culture (Online Resource 1 Fig. S2e).

In contrast to the abovementioned pathways, the activity of pathways related to the tricarboxylic acid (TCA) and urea cycle significantly differed only at one or two single time points between the male and female hepatocytes (data not shown). In addition, the extracellular metabolomic analysis showed no significant sex-specific difference between the amount of acetoacetate, fumarate, 3-hydroxybutyrate or pyruvate in the media of the hepatocytes of both sexes (Online Resource 1 Fig. S3a, c-d, g). Only levels of alpha-ketoglutarate were significantly higher in the medium of the female hepatocytes at 24 and 48 h, while citrate was higher in the female medium at 96 h and malate was higher in the medium of the males at 24 h (Online Resource 1 Fig. S3b, e–f). The levels of lactate remained higher in the medium of the female hepatocytes, except at 48 h (Online Resource 1 Fig. S3h). The levels of urea were similar to those of lactate, although it was only slightly increased in the female hepatocytes at 24 and 72 h (Online Resource 1 Fig. S3i). Ornithine showed no sex-specific differences during cell culture (Online Resource 1 Fig. S3j).

**Fig. 5:**
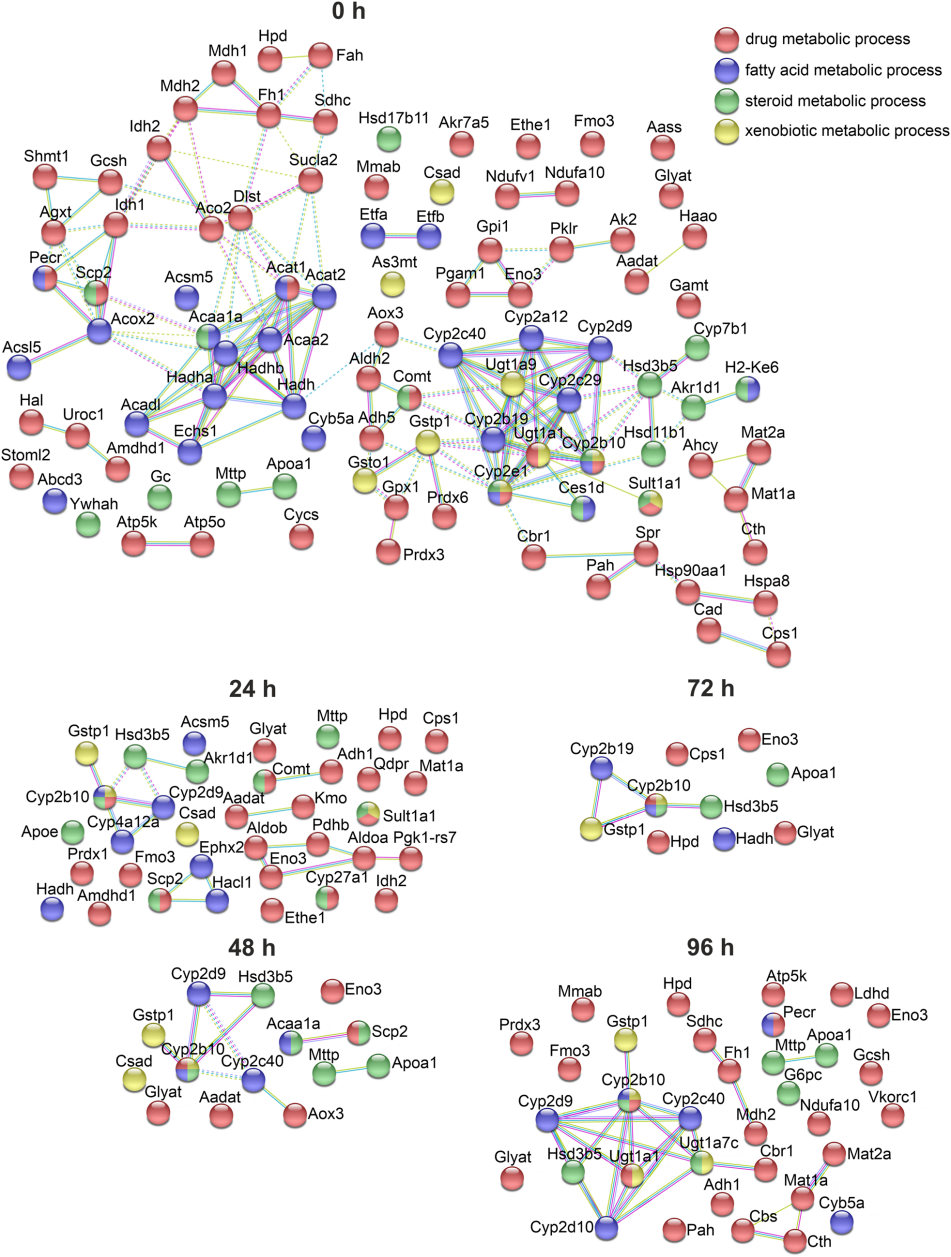
Sex-specific expression of proteins involved in drug, steroid, fatty acid and xenobiotic metabolism during the cultivation of primary murine hepatocytes. Proteome data were analyzed with String 5.18 using a confidence of 0.7 and MCL clustering with an inflation parameter of 3. Only proteins with a *p* value < 0.01 between male and female hepatocytes were used for the analysis. Edges represent protein–protein associations: blue—known interactions from curated databases, violet—known interaction experimentally determined, yellow—texmining. Inter-cluster edges are represented by dashed lines. *N* = 5

In contrast to the variable dynamics of the gene expression in different signaling pathways, the amount of all differentially translated proteins associated with drug, xenobiotic, steroid and fatty acid metabolism were strongly reduced after 24 h of culture between the hepatocytes of male and female mice (Fig. 5). Although the amount of these proteins decreased further during the culture of primary hepatocytes, an increase in sexual dimorph proteins related to drug, fatty acid, steroid and xenobiotic metabolism was measurable during the period from 72 to 96 h.

## Discussion

The liver is one of the most important metabolic organs, regulating important biological functions, such as lipid, glucose, xenobiotic and drug metabolism. In addition, it exhibits a strong sexual dimorphism, which leads to different tolerances, e.g., alcohol and caffeine, between women and men (Frezza et al. 1990; Relling et al. 1992). What seems to be very obvious in these everyday examples has been long ignored with respect to drug doses. Even today, many diseases are still treated in a sex-neutral way, although they show sex-specific trends. Reasons for ignoring these sex-specific differences in the development of new treatments are primarily the greater effort required for the necessary examinations. During the development of new drugs, different cell models are used to optimize candidate selection (Gómez-Lechón et al. 2003). Besides cell lines or differentiated pluripotent stem cells, primary hepatocytes are still the “gold standard” for hepatic in vitro studies, because they maintain many phenotypic, hepatic functions in comparison to other models, although they lose these characteristics after a couple of days (Collins et al. 2019). For considering sex-specific characteristics of primary cell culture in future pharmaceutical investigations, it is important to understand the molecular mechanisms of sex-specific dimorphisms and their development when setting up experiments. Thus, we conducted this study to examine the sexual dimorphism during the culture of primary mouse hepatocytes.

### Regulation of sexual dimorphism in gene expression during the culture of primary hepatocytes

As hepatic sexual dimorphism is primarily regulated by the release of sex hormones and GH (Udy et al. 1997; Waxman and O'Connor 2006; Zheng et al. 2018), which reach the liver mainly through the circulatory system, one would expect that the sex-specific differences in primary hepatocytes to be lost or at least attenuated, during culture in the absence of further stimuli. However, our results showed that sex differences in gene expression related to serotonin, adrenaline and melatonin degradation increased. Meanwhile, the ratio of gene expression associated with androgen signaling tended to be reversed between male and female hepatocytes during the culturing. In contrast, gene expression related to estrogen signaling remained almost the same the entire time, although the amount of mRNA of *Esr1* was significantly reduced in female hepatocytes. Similar observations were made for mRNA expression related to GH, xenobiotic, lipid and AA metabolism. Since the sources of the regulators of sexual dimorphism are not available during the primary hepatocyte culture, other mechanisms, unknown so far, must be critical for these phenomena. This topic should be addressed in further studies.

### The impact of altered sexual dimorphic expression during the culture of primary mouse hepatocytes on medically relevant parameters

To investigate the effects and side effects of drugs on hepatocytes, it is important to know whether sex-specific differences in drug metabolism are maintained during cell culture. The sex-specific difference in gene expression in the general xenobiotic pathway significantly increased over 96 h. However, a closer examination of certain important members of the cytochrome P450 family revealed that the highly female-specific expression of *Cyp2b13* and *Cyp2b9* was significantly reduced during culture. CYP2B enzymes metabolize many drugs and xenobiotics, such as nicotine, cytostatics, herbicides and pesticides (Turpeinen and Zanger 2012). Therefore, reduction of the sex difference may lead to biased results in various drug and toxicity studies in vitro.

The expression of *Cyp1a2* was consistent with the activity of the general xenobiotic pathway, with similar expression between the hepatocytes of both sexes in the beginning. However, the reduction in its expression over time differed between the male and female hepatocytes, creating a significant sex-specific difference after 96 h with higher gene expression in female hepatocytes. During cultivation, the level of CYP1A2 was reduced in the male hepatocytes to a level similar to that in the female hepatocytes (data not shown). CYP1A2 is an important hepatic enzyme that metabolizes drugs, such as caffeine, warfarin and theophylline (Wójcikowski and Daniel 2009; Plowchalk and Rowland Yeo 2012). In addition, the function of CYP1A2 is related to the maximum liver function capacity (LiMAx)-test. Therefore, it is important to understand the behavior of the *Cyp1a2* gene and protein expression during primary hepatocyte culture. Our results illustrate the importance to understand the dynamics of sexual differences in gene and protein expression during cell culture, because they could affect the outcome of pharmacodynamic examinations on primary hepatocytes. Oxidative stress can cause a reduction in *Cyp1a2* expression (Rahman and Thomas 2012). However, this seems to be unlikely in our experiment since other hypoxia-sensitive *Cyps* did not change during cell culture. Therefore, other mechanisms must play a role in the observed changes in sex-specific gene and protein expression of individual CYPs. These effects should be considered when using primary hepatocytes for long-term drug studies.

Another group of metabolic pathways that are highly affected in their sex-dependent expression over time involves lipid metabolism. This observation is crucial, as alterations in lipid metabolism can cause several severe liver diseases, such as hepatitis, NAFLD and HCC. It is known that these conditions show high sexual dimorphism regarding their development, progression and outcome (Ballestri et al. 2017). We showed that sex-specific differences in the expression of genes related to hepatic steatosis tended to be reversed after 72 h of culture, while at the protein level, sex-specific differences correlated with hepatic steatosis seemed to oscillate during culture. Therefore, it is necessary to consider sexual dimorphism, while studying lipid-associated liver damage, such as steatosis. For example, the antidiabetic drug pioglitazone caused a significant reduction in hepatic steatosis; however, it also showed several sex-specific behaviors in various contexts (Cusi et al. 2016; Park et al. 2016; Gensel et al. 2019). In addition, it would be interesting to examine which mechanisms cause these changes. Therefore, further studies should be conducted.

### Sexual dimorphism in amino acid consumption is lost during the cultivation of primary hepatocytes

A study from 2018 revealed that the livers of female mice tend to use more AA to maintain lipid synthesis after short-term fasting than the livers of male mice (Della Torre et al. 2018). This phenomenon is regulated by ESR1 (Della Torre et al. 2018). We made a similar observation in our study. Our results revealed that hepatocytes of the female mice consumed more AA than the male hepatocytes. During cultivation, the AA consumption of the male and female hepatocytes approached similar levels, which was accompanied by a reduction in the sex-specific gene expression of *Esr1*. Despite these results, the activity of AA degradation was significantly higher in the male hepatocytes than in the female hepatocytes at 24 h. However, this activity pattern significantly reversed during further culture, suggesting that there must be additional regulatory mechanisms that control the sex-specific regulation of AA metabolism. In addition, it is necessary to consider that the medium was changed every 24 h. This means that the measured AA consumption for every time point represents only the previous 24 h, while the measured transcriptome data represent the entire period from isolation to the corresponding time of collection. These different states may explain the discrepancies between the extracellular metabolome and transcriptome data.

### Which culture period is best suited for conducting experiments related to sexual dimorphism?

In our study, we examined the influence of ordinary cell culture on the sex-dependent gene and protein expression of primary mouse hepatocytes. Our results indicate that the culture period for primary hepatocytes needs to be adapted to the research issue and may be different for gene and protein analysis. The largest change in gene regulation was seen after 24 h of culture. This was certainly caused by the stress the cells endured during isolation and their adaption to the new environment. Usually, 24–48 h of culture are used in studies, which may be worth considering since the PCA plot showed that the sex-specific gene expression pattern after 72 h was most similar to the initial state. Nevertheless, at the protein level, the smallest changes in the PCA plot were observed after 24 h. However, sex-specific differences in medically relevant mechanisms were greatly reduced after 24 h of culture and increased again from 72 to 96 h of culture.

For a better reproduction of in vivo liver conditions in culture, various approaches, like sandwich-cultured hepatocytes, organ-on-a-chip or precision-cut tissue slices, are developing (Collins et al. 2019). Especially the cell heterogeneity and the spatial organization of liver parenchyma are essential and differently focused by the mentioned approaches. Since in our study pure hepatocyte cell culture was used, we suggest co-culture systems for future investigations. Even if the metabolic impact of non-parenchymal cells is much lower than that of hepatocytes, the cell–cell interactions and the exchange of metabolites may benefit the maintenance of sex specificity. Furthermore, the application of precision-cut tissue slices would preserve the tissue architecture and are an interesting tool to study toxicity (de Graaf et al. 2010).

Several studies have shown that it is possible to feminize the gene expression of several genes in males, both in vivo and in vitro, by adding continuous levels of GH to mimic the GH release in females (Lau-Corona et al. 2017; Thangavel et al. 2004). Nevertheless, both studies reported that it was not possible to simulate the complete gene expression pattern of females and that female mice and hepatocytes were more sensitive to GH treatment than male mice and their hepatocytes (Lau-Corona et al. 2017; Thangavel et al. 2004). This outcome indicates the irreversible differences in the liver metabolism of males and females that cannot be overcome by the simple addition of growth or sex hormones in cell culture. Further studies are urgent to improve our understanding of sexual dimorphisms in liver metabolism. Additionally, we need to either perform studies in primary hepatocytes of both sexes or create suitable experimental models to take into account the natural sex-dependent metabolism.

## Conclusions

Our study demonstrated that the sex-specific phenotype of primary mouse hepatocytes is attenuated during cell culture. However, in some cases, sex differences increase or even reverse during hepatocyte culture. These results raise many questions. Besides well-known factors controlling hepatic sexual dimorphism (van Nas et al. 2009; Waxman and O'Connor 2006), our results suggest that there may be a hitherto unknown mechanism that controls sex-specific regulation. This study shows the crucial importance of understanding sex-dependent differences and regulatory mechanisms in primary hepatocyte metabolism over time. Therefore, further studies should be conducted to identify new mechanisms of the sex-specific regulation of liver metabolism.

## Supplementary Information

Below is the link to the electronic supplementary material.

Supplementary file1 (PDF 436 kb)

Supplementary file2 (DOCX 836 kb)

Supplementary file3 (XLSX 600 kb)

Supplementary file4 (XLSX 101 kb)

Supplementary file5 (PDF 18016 kb)

## Data Availability

Raw and normalized data of microarray analyses were deposited with GEO under the accession number GSE166969. Metadata of proteome and extracellular metabolome analyses are available in the Electronic supplementary material (EMS3 and EMS4).
