# Supplementary material for: Sex-dependent dynamics of metabolism in primary mouse hepatocytes
Source: Arch Toxicol. 2021 Jul 9;95(9):3001–13. doi: 10.1007/s00204-021-03118-9 (PMC8380230; doi:10.1007/s00204-021-03118-9)
Supplement: Supplementary file 1 — Supplementary file1 (PDF 436 kb) [file 204_2021_3118_MOESM1_ESM.pdf]

– SUPPLEMENTARY INFORMATION –

ARCHIVES OF TOXICOLOGY

Sex-dependent dynamics of metabolism in primary mouse hepatocytes

Luise Hochmuth, Christiane Körner, Fritzi Ott, Daniela Volke, Kaja Blagotinšek Cokan, Peter Juvan, Mario Brosch, Ute Hofmann, Ralf Hoffmann, Damjana Rozman, Thomas Berg, Madlen Matz-Soja

Correspondence: Madlen Matz-Soja (madlen.matz@medizin.uni-leipzig.de), Division of Hepatology, Clinic and Polyclinic for Oncology, Gastroenterology, Hepatology, Infectious Diseases, and Pneumology, University Hospital Leipzig, Leipzig, Germany

Supplementary Materials and Methods

Maintaining and Feeding of the Mice

The mice were housed in a pathogen-free facility with a 12:12 h light:dark cycle. Animals were fed *ad libitum* with regular chow (Ssniff® V1534-300 composed of 24.0 kJ% protein, 67 kJ% carbohydrate, 9 kJ% fat; Ssniff® Spezialdiäten GmbH, Soest, Germany) and tap water throughout life. Five to ten male and female mice were sacrificed during primary hepatocyte isolation procedure at the age of three months after administration of an anesthetic consisting of ketamine, xylazine and atropine between 8 and 11 am (zeitgeber time 2-5). The sample size was estimated according to our experience from previous, liver-related studies (Matz-Soja et al. 2014; Schmidt-Heck et al. 2015; Matz-Soja et al. 2016). The number of biological replicates varied by experiment (N=4–5) as specified in each figure.

Culture of Primary Mouse Hepatocytes

The isolated hepatocytes from each individual mouse were suspended in Williams Medium E (Biochrom GmbH, Berlin, Germany, product No.: F1115) containing 10% foetal bovine serum (FBS) (PAA Laboratories GmbH, Pasching, Austria, Lot No.: A15111-3719), 20 µM glutamine (Carl Roth GmbH & Co. KG, Karlsruhe, Germany, Cat. No.: 3772.1), penicillin and streptomycin (Capricorn Scientific GmbH, Ebsdorfergrund, Germany, Cat. No. PS-B), and 90 pM dexamethasone (Sigma-Aldrich, St. Louis, USA, Cat. No. D1756) and seeded into cell culture plates coated with collagen type I. After 2 to 3 h, the medium was changed to serum-free (sf) medium. After 24, 48, and 72 h of culture, fresh sf medium was applied. Samples for proteomic, transcriptomic, extracellular metabolomic and qPCR analyses were collected at all time points (Fig. S1). Mice in different stages of the reproductive cycle were used in our experiments, which is in line with the situation in human where the phase of the estrous cycle is usually not considered during treatments.

Microarray-based gene expression analysis

Data analysis were performed using R and Bioconductor software packages. We normalized raw (CEL) expression data using the RMA algorithm from package oligo (Carvalho and Irizarry 2010). Quality control and outlier detection were performed using package arrayQualityMetrics before and after normalization (Kauffmann et al. 2009). Package limma (Smyth 2004) was used to fit individual

normalized gene expression data using a linear regression model ~Sex*CultureTime. Statistical significance of gene expression differences between sexes was estimated at each time point. Empirical Bayes approach was applied for variance stabilization and Benjamini-Hochberg procedure was used to control false discovery rate (FDR) of differential expression at alpha = 0.05. No log fold change cut-off was used for selection of differentially expressed genes.

KEGG pathways (Kanehisa et al. 2019) and TRANSFAC database ver. 2020.1 (Matys et al. 2006) were used for functional enrichment studies. Gene sets containing five or more elements were constructed and tested for enrichment using the PGSEA package (Furge and Dykema 2012). In the case of transcription factor (TFs) enrichment, factors were merged based on their ID irrespective of their binding sites. Statistical significance of gene set enrichment was estimated using the same linear regression approach as for individual genes.

Log2 fold change of up- and down-regulated genes from male compared to female hepatocytes were used to conduct an activation Z-score analysis with the Ingenuity Pathway Analysis (IPA) software (QIAGEN, Venlo, Netherlands) using a p-value cutoff of 0.05. GraphPad Prism 7.03 (GraphPad Software, San Diego, CA, USA) was used for visualization of the activation Z-score analyses. The principal component plot (PCA) was created using R software (R Core Team 2020) and CRAN software packages gplots (Warnes et al. 2020), ggplot2 (Wickham 2009), ggrepel (Slowikowski 2020) and ggpubr (Kassambara 2020).

Proteome Analysis

Primary mouse hepatocytes ($1.8x10^6$) of 5 mice per sex were suspended in 500 µL of lysis buffer (60 mmol/L Tris-HCl, pH 6.8; 10% (w/v) glycerine; and 2% (w/v) SDS). The cell suspension was heated at 95 °C for 5 min and centrifuged at 12,000 x g for 5 min to collect cellular debris. Proteins in the supernatant were digested using the filter-aided sample preparation method (Ostasiewicz et al. 2010) with the following modifications. 90 µL of supernatant of each sample (0.33 mg/mL) was mixed with 10 µL of lysis buffer containing 1 mol/L dithiothreitol (DTT) to yield a final concentration of 0.1 mol/L DTT in the sample. Before adding the sample to the filter device, samples were mixed with 150 µL of urea solution (8 M urea in 0.1 M Tris/HCl, pH 8.5), followed by steps performed according to a previously described method (Ostasiewicz et al. 2010).

Tryptic digests of the proteome samples were diluted with 0.2% formic acid to yield a concentration of 0.1 µg/µL. 20 µL of this sample solution was mixed with 170 µL of aqueous acetonitrile (3%, v/v) containing 0.1% formic acid and 10 µL enolase digest (200 fmol/µL, Waters GmbH, Eschborn). 100 ng of this sample was analyzed on a Waters nanoACQUITY Ultra Performance Liquid Chromatography (nano UPLC) system coupled online to an electrospray ionization quadrupole time-of-flight mass spectrometer (ESI-QTOF-MS, Synapt G2Si MS, Waters, MS Technologies, Manchester, UK). Samples were loaded on a C18-trap column (2G-V/M Trap Symmetry, Waters, 180 µm internal diameter (ID), 2 cm length, and 5 µm particle size) using full loop injection. This analysis was followed by separation on a C18-column (nanoACQUITY UPLC Peptide BEH, 75 µm ID, 10 cm length, and 1.7 µm particle size) at a column temperature of 35°C using a linear gradient from 3 to 48% aqueous acetonitrile (0.1% formic acid) in 90 min to 90% aqueous acetonitrile (0.1% formic acid) in 4 min. Spectra were recorded by data-independent acquisition in positive ion MSe mode using the following settings: m/z 50 to 2000, sampling

cone of 30 V, source offset of 80 V, source temperature of 100°C, cone gas flow of 20 L/h, purge gas flow of 600 mL/h, nanoflow gas pressure of 0.2 bar, and a scan time of 0.9 s. Fragmentation was triggered in the trap before the IMS cell, using a collision energy ramp from 14 to 40 V. The doubly protonated signal of Glu-1-Fibrinopeptide B at *m/z* 785.8426 was acquired as lockmass.

After acquisition, the MassLynx Software package (Version 4.2 SCN983, Waters) triggered automatic processing of the acquired data using a Symphony data pipeline (Symphony Software, Version 1.0, Waters). The Symphony data pipeline consits out of four different tasks. Data were transfer to a GPU-computing-cluster (2 knots, 4 virtual machines each), where the best low and high-energy values were calculated using PLGS_Threshold_Inspector.exe (https://sourceforge.net/projects/plgsthresholdin/ version 2.4.1). Data were transferred back to an analysis computer and were loaded to the Progenesis QI for proteomics software (version 4.2, Nonlinear Dynamics, Waters) using the previously calculated best values. A database search was performed within Progenesis QI for proteomics using the PLGS search engine with the following parameters: database UniProt mouse (17,027 sequences, downloaded 05.02.2020, with the addition of the enolase entry P00924), two missed cleavage sites, trypsin as the “digester reagent”, and methionine oxidation and cysteine carbamidomethylation as variable modifications. Using the IPA plugin in Progenesis QI for proteomics, data were exported for further statistical analysis using IPA software (QIAGEN) similar to the RNA array activation Z-score analysis. The figures of the amount of sexual dimorph proteins related to drug, fatty acid, steroid and xenobiotic metabolism during the culture were created with STRING 11.0 (Szklarczyk et al. 2019) using a p-value cutoff of 0.01. The protein-protein associations are based on textmining, experiments and databases. A minimum required interaction score of 0.7 and a MCL clustering of 3 were used. The PCA plot was created similar to the one of the RNA array using only proteins with at least three unique peptides.

Extracellular Metabolome Analysis

Lactate and bile acids were determined by negative ESI LC–MS/MS in multiple-reaction-monitoring (MRM) mode on an Agilent 6460 triple-quadrupole mass spectrometer (Agilent, Waldbronn, Germany) coupled to an Agilent 1200 high-performance liquid chromatography (HPLC) system. Ion pairing reversed-phase (RP)-HPLC separation of lactate was performed similar to a previously described method (Maier et al. 2010), and the MRM transitions were 92 → 45 and 89 → 43 for lactate and the internal standard [13C3]lactate, respectively. The bile acids ß-muricholate, tauromuricholate and taurocholate were separated on a Poroshell 120 EC-C18 column (100 × 2.1 mm, 2.7 µm particle size, Agilent) using a gradient of (A) 12 mm ammonium acetate in water and (B) acetonitrile as mobile phases at a flow rate of 0.5 ml/min. MRM transitions were 407.3 → 407.3 for ß-muricholate, 514.3 → 80 for taurocholate and tauromuricholate, and 518.3 → 80 for the internal standards [2H4]tauromuricholate and [2H4]taurocholate. For further analyses, the data were normalized to the total protein amount of each sample. The results are presented as the means of biological replicates ± SEM. Statistical significance was calculated with two-way ANOVA using GraphPad Prism 7 software (GraphPad Software). P-values are indicated as $p < 0.05$ (*), $p < 0.01$ (**) and $p < 0.001$ (***).

Supplementary Figures

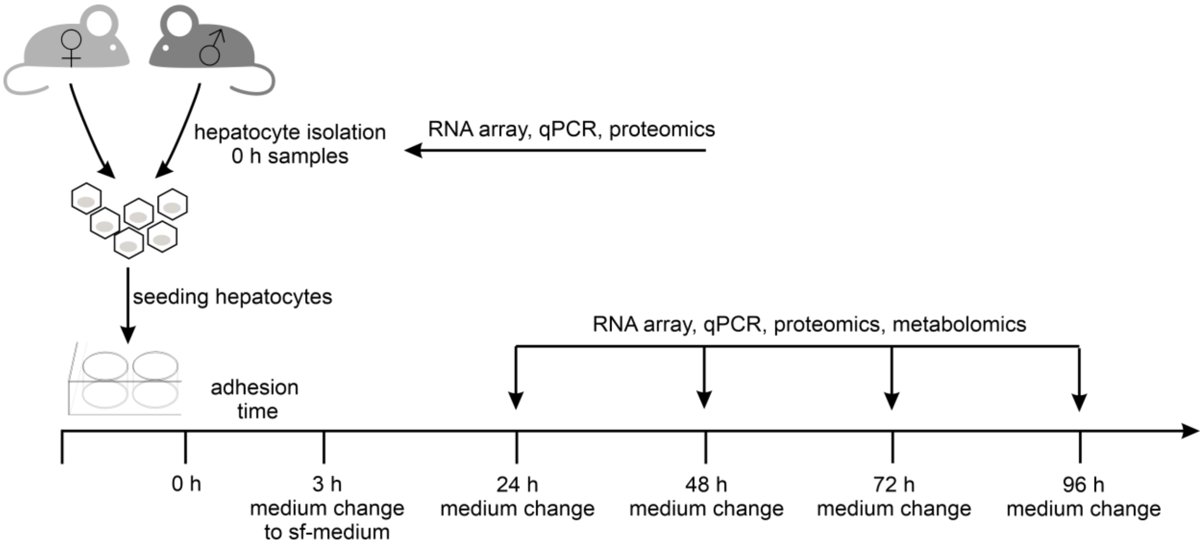

Fig. S1 Schematic representation of the experimental study design. Primary hepatocytes of each mouse were isolated and cultured individually up to 96 h. sf – serum-free medium

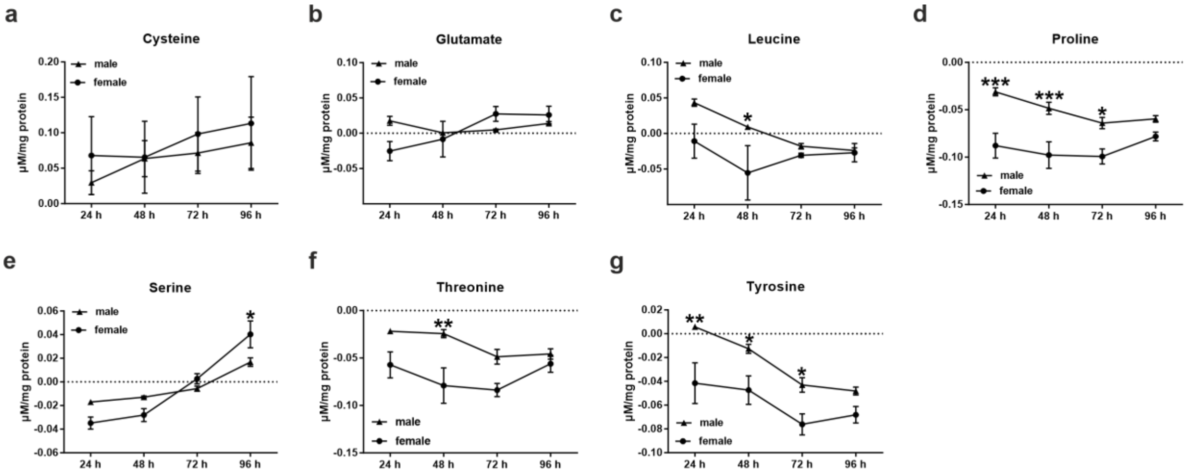

Fig. S2 Sex specific amino acid (AA) consumption of hepatocytes. A-G: Extracellular metabolome analysis. Error bars show SEM. Significance was calculated with two-way-ANOVA. Stars show the significance between hepatocytes from male and female mice at a certain time point. * - P ≤ 0.05, ** - P ≤ 0.01, *** - P ≤ 0.001. N=5

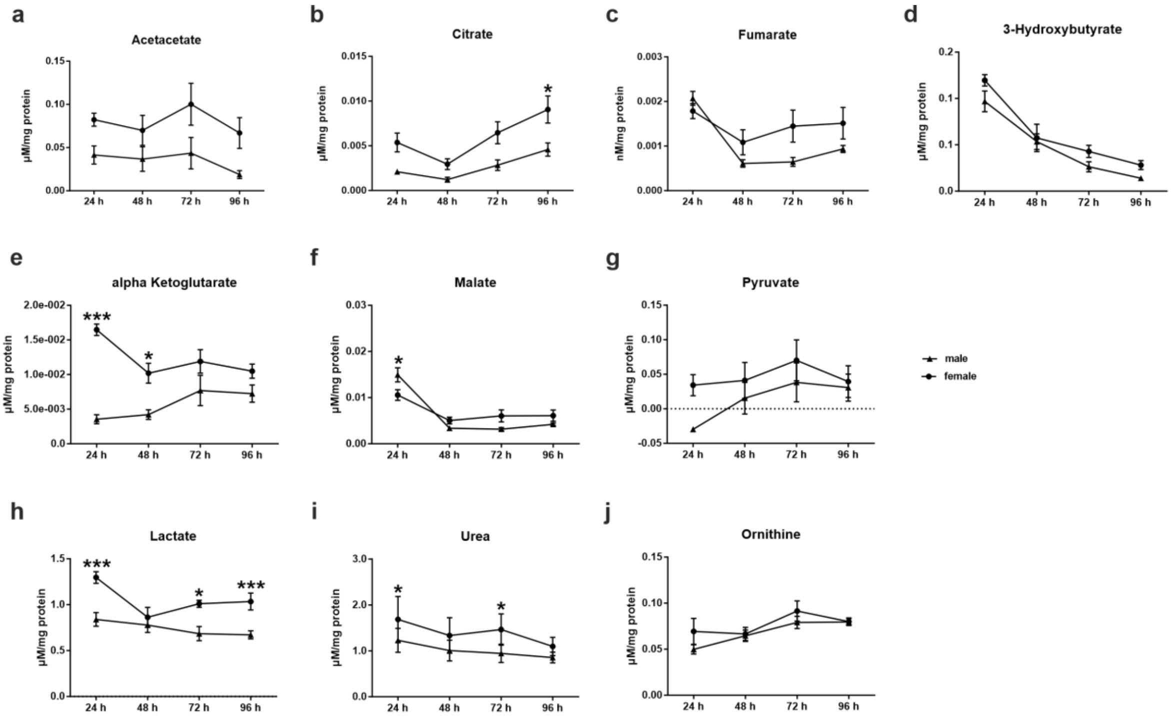

Fig. S3 Metabolome analysis of molecules related to tricarboxylic acid cycle and urea cycle. A-J: Extracellular metabolome analysis. Error bars show SEM. Significance was calculated with two-way-ANOVA. Stars show the significance between hepatocytes from male and female mice at a certain time point. * - P ≤ 0.05, ** - P ≤ 0.01, *** - P ≤ 0.001. N = 5

Supplementary Tables

Table S1-S53 Activation Z-score of gene and protein expression male vs. female. Activation Z-score was calculated with IPA software from Qiagen. (see separate Word file 'EMS 2')

Table S54 Primer sequences for qPCR. f: forward primer; r: reverse primer.

Gene Names	Primer Sequences (5' – 3')	
Ar	f	ccagtcccaattgtgtcaaa
	r	tccctggtactgtccaaacg
Cyp17a1	f	catcccacacaaggctaaca
	r	cagtgcccagagattgatga
Esr1	f	tgcaatgactatgcctctgg
	r	ttgtagctggacacatgtagtcatt
Star	f	tgttcctcgctacgttcaag
	r	gtcgaacttgacccatccac
Ywhaz	f	ttacttggccgaggttgct
	r	tgctgtgactggtccacaat

Supplementary References

Carvalho BS, Irizarry RA (2010) A framework for oligonucleotide microarray preprocessing. Bioinformatics:2363–2367. https://doi.org/10.1093/bioinformatics/btq431

Furge K, Dykema K (2012) PGSEA: Parametric Gene Set Enrichment Analysis

Kanehisa M, Sato Y, Furumichi M, Morishima K, Tanabe M (2019) New approach for understanding genome variations in KEGG. Nucleic Acids Res 47(D1):D590-D595. https://doi.org/10.1093/nar/gky962

Kassambara A (2020) ggpubr: "ggplot2" Based Pbulication Ready Plots

Kauffmann A, Gentleman R, Huber W (2009) arrayQualityMetrics--a bioconductor package for quality assessment of microarray data. Bioinformatics:415–416. https://doi.org/10.1093/bioinformatics/btn647

Maier K, Hofmann U, Reuss M, Mauch K (2010) Dynamics and control of the central carbon metabolism in hepatoma cells. BMC Syst Biol 4:54. https://doi.org/10.1186/1752-0509-4-54

Matys V, Kel-Margoulis OV, Fricke E, Liebich I, Land S, Barre-Dirrie A, Reuter I, Chekmenev D, et al. (2006) TRANSFAC and its module TRANSCompel: transcriptional gene regulation in eukaryotes. Nucleic Acids Res 34(Database issue):D108-10. https://doi.org/10.1093/nar/gkj143

Matz-Soja M, Aleithe S, Marbach E, Böttger J, Arnold K, Schmidt-Heck W, Kratzsch J, Gebhardt R (2014) Hepatic Hedgehog signaling contributes to the regulation of IGF1 and IGFBP1 serum levels. Cell Commun Signal 12:11. https://doi.org/10.1186/1478-811X-12-11

Matz-Soja M, Rennert C, Schönefeld K, Aleithe S, Boettger J, Schmidt-Heck W, Weiss TS, Hovhannisyan A, et al. (2016) Hedgehog signaling is a potent regulator of liver lipid metabolism and reveals a GLI-code associated with steatosis. Elife 5. https://doi.org/10.7554/eLife.13308

Ostasiewicz P, Zielinska DF, Mann M, Wiśniewski JR (2010) Proteome, phosphoproteome, and N-glycoproteome are quantitatively preserved in formalin-fixed paraffin-embedded tissue and analyzable by high-resolution mass spectrometry. J Proteome Res 9(7):3688–3700. https://doi.org/10.1021/pr100234w.

R Core Team (2020) R: A language and environment for statistical computing. R Foundation for Statistical Computing. R Foundation for Statistical Computing, Vienna, Austria

Schmidt-Heck W, Matz-Soja M, Aleithe S, Marbach E, Guthke R, Gebhardt R (2015) Fuzzy modeling reveals a dynamic self-sustaining network of the GLI transcription factors controlling important metabolic regulators in adult mouse hepatocytes. Mol Biosyst 11(8):2190–2197. https://doi.org/10.1039/c5mb00129c

Slowikowski K (2020) ggrepel: Automatically Position Non-Overlapping Text Labels with "ggplot2"

Smyth GK (2004) Linear models and empirical bayes methods for assessing differential expression in microarray experiments. Stat Appl Genet Mol Biol. https://doi.org/10.2202/1544-6115.1027

Szklarczyk D, Gable AL, Lyon D, Junge A, Wyder S, Huerta-Cepas J, Simonovic M, Doncheva NT, et al. (2019) STRING v11: protein-protein association networks with increased coverage, supporting functional discovery in genome-wide experimental datasets. Nucleic Acids Res 47(D1):D607-D613. https://doi.org/10.1093/nar/gky1131

Warnes GR, Bolker B, Bonebakker L, Gentleman R, Huber W, Liaw A, Lumley T, Maechler M, Magnusson A, Moeller S, Schwartz M, Venables B, Galili T (2020) gplots: Various R Programming Tools for Plotting Data

Wickham H (2009) ggplot2: Elegant Graphics for Data Analysis. Use R. Springer-Verlag New York, New York, NY
