# Supplementary material for: Sex-dependent dynamics of metabolism in primary mouse hepatocytes
Source: Arch Toxicol. 2021 Jul 9;95(9):3001–13. doi: 10.1007/s00204-021-03118-9 (PMC8380230; doi:10.1007/s00204-021-03118-9)
Supplement: Supplementary file 2 — Supplementary file2 (DOCX 836 kb) [file 204_2021_3118_MOESM2_ESM.docx]

**– Supplementary Information –**

**Archives of Toxicology**

**Sex-dependent dynamics of metabolism in primary mouse hepatocytes**

Luise Hochmuth, Christiane Körner, Fritzi Ott, Daniela Volke, Kaja Blagotinšek Cokan, Peter Juvan, Mario Brosch, Ute Hofmann, Ralf Hoffmann, Damjana Rozman, Thomas Berg, Madlen Matz-Soja

**Correspondence:** Madlen Matz-Soja ([madlen.matz@medizin.uni-leipzig.de](mailto:madlen.matz@medizin.uni-leipzig.de)), Division of Hepatology, Clinic and Polyclinic for Oncology, Gastroenterology, Hepatology, Infectious Diseases, and Pneumology, University Hospital Leipzig, Leipzig, Germany

**Supplementary Tables**

**Color scheme:**

| **Z-score** | | | | |
| --- | --- | --- | --- | --- |
| -0,5 < Z-score < 0,5 | |  |  |  |
| 0,5 = Z-score < 1 | 1 = Z-score < 1,5 | 1,5 = Z-score < 2 | 2 = Z-score < 2,5 | Z-score ≥ 2,5 |
| -0,5 = Z-score > -1 | -1 = Z-score > -1,5 | -1,5 = Z-score > -2 | -2 = Z-score > -2,5 | Z-score ≤ -2,5 |

**Table S1:** Activation Z-score of genes involved in the degradation of serotonin male vs. female. Activation Z-score was calculated with IPA software from Qiagen.

| **Pathway Summary** | -0,5 | 1,091 | -0,943 | -2,887 | -2,887 |
| --- | --- | --- | --- | --- | --- |
| **Genes in the Serotonin Degradation network** | **0 h** | **24 h** | **48 h** | **72 h** | **96 h** |
| ***Ugt2B17*** | 0,557028 | 2,213161 | 1,292475 | 0,591189 | -0,39135 |
| ***Adh4*** | 0,843657 | 1,099951 | -0,02072 | -0,12201 | -0,06158 |
| ***Ugt2B28*** | 0,774388 | 0,659801 | 0,273055 | -0,19352 | -0,16884 |
| ***Aldh4a1*** | -0,18994 | 1,145047 | 0,371212 | 0,082255 | -0,3707 |
| ***Smox*** | -0,38514 | 0,265107 | 0,58581 | 0,252859 | 0,094358 |
| ***Adh5*** | 0,051015 | 0,302692 | 0,253513 | 0,082737 | 0,112555 |
| ***Akr1a1*** | 0,332914 | 0,263026 | -0,02438 | -0,09479 | -0,01213 |
| ***Aldh3a1*** | -0,01797 | 0,141252 | 0,02739 | 0,166023 | 0,14445 |
| ***Ugt2b7*** | 0,55257 | -0,32078 | 0,463908 | -0,14661 | -0,11281 |
| ***Aldh7a1*** | 0,220354 | 0,819824 | -0,32258 | -0,21583 | -0,08076 |
| ***Adhfe1*** | 0,120446 | 0,657312 | 0,167675 | -0,18753 | -0,33779 |
| ***Adh6a*** | -0,09923 | 0,191731 | -0,05508 | 0,066197 | 0,147457 |
| ***Aldh1a2*** | -0,04424 | 0,074551 | -0,0644 | 0,130846 | 0,115865 |
| ***B3gat3*** | 0,044436 | -0,0449 | -0,00396 | 0,117245 | 0,087244 |
| ***Sult1b1*** | -0,06713 | 0,322959 | 0,273638 | -0,20484 | -0,14874 |
| ***Large2*** | 0,198625 | 0,126268 | -0,19906 | -0,11465 | 0,127858 |
| ***Aldh3b2*** | -0,23295 | 0,191515 | 0,047374 | -0,23983 | 0,346124 |
| ***B4gat1*** | 0,028608 | 0,044543 | 0,062539 | -0,05311 | -0,00796 |
| ***Ugt2a1*** | -0,01249 | 0,005613 | 0,013367 | 0,025455 | 0,025249 |
| ***Ext2*** | 0,055656 | 0,027359 | -0,09587 | -0,06187 | 0,062621 |
| ***Sult1c3*** | -0,34907 | 0,08808 | 0,041571 | -0,07432 | 0,237151 |
| ***Ugt2a3*** | 0,006423 | 1,216072 | -0,00087 | -0,60617 | -0,79121 |
| ***Csgalnact1*** | 0,013946 | -0,10522 | 0,066224 | -0,03088 | -0,17641 |
| ***Adh7*** | -0,01942 | 0,083544 | -0,20337 | 0,123429 | -0,29415 |
| ***Aldh2*** | -0,05226 | 0,209599 | -0,10349 | -0,25722 | -0,14803 |
| ***Dhrs4*** | 0,191709 | 0,457548 | -0,36069 | -0,35123 | -0,30604 |
| ***Large1*** | 0,509803 | -1,06261 | -0,15702 | 0,061787 | 0,240405 |
| ***Adh1c*** | -0,16409 | 0,881367 | -0,32695 | -0,40502 | -0,41997 |
| ***Aldh3b1*** | -0,26231 | 0,03008 | -0,35622 | 0,084234 | 0,031547 |
| ***Aldh3b3*** | -0,98556 | 0,053856 | 0,106851 | 0,059383 | 0,170798 |
| ***Ugt2b10*** | -0,06221 | -0,04937 | 0,124047 | -0,33365 | -0,35 |
| ***Aldh1a3*** | -0,15936 | -0,26665 | -0,27743 | -0,07322 | 0,015636 |
| ***Aldh9a1*** | -0,30241 | 0,092659 | -0,28733 | -0,27964 | -0,1646 |
| ***Aldh1a1*** | 0,149176 | -0,68979 | -0,46523 | -0,18232 | 0,010048 |
| ***Ugt3a1*** | -0,17178 | 1,538057 | 0,565115 | -1,43647 | -1,80589 |
| ***Aldh1b1*** | -0,4054 | -0,14953 | -0,38866 | -0,21044 | -0,2086 |
| ***Dhrs9*** | -0,26926 | -0,47193 | -0,06055 | -0,13292 | -0,43896 |
| ***Aldh3a2*** | -0,07275 | -0,76701 | -0,39468 | -0,41599 | -0,25463 |
| ***Sult1d1*** | -0,75893 | -0,67835 | -0,02509 | -0,09733 | -0,65697 |
| ***Maob*** | -0,68574 | -0,33792 | -0,72165 | -0,47743 | -0,326 |
| ***Sult1a1*** | -0,73322 | -1,18578 | -0,9819 | -0,42152 | -0,10652 |
| ***Maoa*** | -0,70329 | -0,4951 | -1,32169 | -1,29354 | -0,54919 |

**Table S2:** Activation Z-score of genes involved in androgen signaling male vs. female. Activation Z-score was calculated with IPA software from Qiagen.

| **Pathway Summary** | -0,775 | -0,816 | 0,832 | 0,535 | 1,069 |
| --- | --- | --- | --- | --- | --- |
| **Genes in the androgen signaling network** | **0 h** | **24 h** | **48 h** | **72 h** | **96 h** |
| ***Jun*** | 0,719868 | 0,883187 | 0,549306 | 0,296255 | 0,375915 |
| ***Dnajb1*** | 0,551586 | 0,401353 | 0,556759 | 0,357208 | 0,339142 |
| ***Gnb5*** | -0,14654 | 0,755772 | 0,324911 | 0,554182 | 0,2182 |
| ***Gnao1*** | 0,259911 | 0,201797 | 0,213923 | 0,232768 | 0,291226 |
| ***Gnai1*** | 0,24009 | 0,085879 | 0,3033 | 0,310778 | 0,25472 |
| ***Polr2l*** | 0,309257 | 0,111477 | 0,41412 | 0,095528 | 0,24882 |
| ***Gna12*** | 0,650449 | 0,30947 | 0,083986 | -0,01054 | 0,062736 |
| ***Mras*** | 0,174646 | 1,003178 | -0,15429 | -0,08634 | 0,102783 |
| ***Prkcg*** | -0,04543 | 0,032092 | 0,447063 | 0,40637 | 0,164773 |
| ***Polr2j*** | 0,521095 | 0,128572 | 0,269868 | -0,03234 | 0,09455 |
| ***Cacnb1*** | -0,05609 | 0,25814 | 0,319247 | 0,026194 | 0,236718 |
| ***Cacna1i*** | -0,10633 | 0,237755 | 0,235186 | 0,048031 | 0,36444 |
| ***Cacnb4*** | 0,010777 | 0,304538 | 0,312479 | -0,0494 | 0,181523 |
| ***Cacna1s*** | 0,045355 | 0,098768 | 0,128293 | 0,065133 | 0,402048 |
| ***Polr2a*** | 0,154523 | 0,128777 | 0,006396 | 0,442991 | -0,00695 |
| ***Gna15*** | 0,11704 | 0,128682 | 0,186349 | -0,04242 | 0,334794 |
| ***Gng4*** | 0,161918 | 0,305121 | -0,01693 | 0,223903 | 0,033712 |
| ***Prkcd*** | 0,074277 | 0,348892 | 0,246503 | 0,028518 | 0,008762 |
| ***Ercc2*** | -0,02735 | 0,284122 | 0,059029 | 0,189092 | 0,184635 |
| ***Kat7*** | 0,30003 | 0,239376 | 0,057094 | 0,123526 | -0,03114 |
| ***Nfkb1*** | 0,158929 | 0,198966 | -0,05669 | 0,144947 | 0,239545 |
| ***Cacng2*** | 0,017724 | 0,198478 | 0,109997 | -0,0074 | 0,366088 |
| ***Cacng8*** | 0,008622 | 0,107768 | 0,287653 | 0,101776 | 0,176022 |
| ***Rela*** | 0,172055 | 0,224581 | 0,095794 | 0,07538 | 0,090153 |
| ***Gnaz*** | 0,193874 | 0,107859 | 0,148681 | 0,126389 | 0,058139 |
| ***Prkar1b*** | -0,0144 | 0,23196 | 0,228917 | 0,084269 | 0,096835 |
| ***Cacna1e*** | 0,129002 | -0,09285 | 0,004205 | 0,18798 | 0,395057 |
| ***Polr2i*** | 0,175542 | 0,025813 | 0,103586 | 0,199543 | 0,093275 |
| ***Hsp90aa1*** | 0,502608 | -0,32229 | 0,178781 | 0,094177 | 0,142744 |
| ***Cacng6*** | -0,03307 | 0,303831 | 0,07322 | -0,15503 | 0,37013 |
| ***Cacna1h*** | -0,19318 | 0,348514 | 0,102798 | -0,01861 | 0,304007 |
| ***Ercc3*** | 0,300378 | 0,049315 | -0,02093 | 0,25926 | -0,04932 |
| ***Gng5*** | 0,25983 | -0,08528 | 0,135085 | 0,151036 | 0,056332 |
| ***Cacng7*** | 0,149059 | 0,189583 | 0,235946 | -0,13443 | 0,039857 |
| ***Prkar2b*** | -0,27859 | 0,108796 | 0,194686 | 0,282446 | 0,160946 |
| ***Gtf2h5*** | 0,288639 | 0,127746 | -0,08328 | 0,034254 | 0,100136 |
| ***Cacna2d1*** | -0,17089 | 0,053001 | 0,239831 | 0,273729 | 0,056185 |
| ***Polr2h*** | 0,255908 | 0,197954 | 0,09597 | 0,060691 | -0,15887 |
| ***Cacnb2*** | -0,13529 | 0,271521 | 0,075619 | -0,00103 | 0,228861 |
| ***Crebbp*** | 0,186457 | 0,187408 | -0,11777 | 0,234118 | -0,05094 |
| ***Gtf2e2*** | 0,103661 | 0,09209 | 0,133753 | 0,130948 | -0,02324 |
| ***Cacna1f*** | 0,007547 | 0,242404 | -0,04017 | 0,137028 | 0,063614 |
| ***Gna14*** | 0,170907 | 0,213407 | -0,01014 | -0,10352 | 0,129124 |
| ***Polr2g*** | 0,155926 | 0,014632 | 0,061713 | -0,00169 | 0,149725 |
| ***Gnb2*** | 0,148041 | 0,151365 | -0,01693 | 0,063766 | 0,025134 |
| ***Gtf2b*** | 0,443335 | -0,27385 | 0,060709 | 0,085171 | 0,054977 |
| ***Shbg*** | -0,28459 | 0,198341 | 0,236826 | 0,217658 | -0,00519 |
| ***Cacng1*** | -0,133 | 0,222536 | 0,225011 | -0,01992 | 0,065548 |
| ***Gnb3*** | -0,03474 | 0,049647 | 0,092079 | 0,011347 | 0,238027 |
| ***Ep300*** | 0,223775 | -0,03702 | 0,045473 | 0,279534 | -0,16359 |
| ***Cacna2d4*** | 0,05694 | 0,342115 | -0,09898 | -0,03771 | 0,082057 |
| ***Camk4*** | 0,132041 | 0,0081 | 0,125959 | -0,00908 | 0,078172 |
| ***Gtf2h1*** | 0,053027 | -0,13832 | 0,205748 | 0,100247 | 0,106048 |
| ***Polr2c*** | 0,209964 | 0,021694 | 0,04099 | -0,02894 | 0,077666 |
| ***Polr2f*** | 0,16294 | -0,47465 | 0,281367 | 0,163597 | 0,182947 |
| ***Src*** | -0,27544 | 0,024078 | 0,202661 | 0,159584 | 0,199357 |
| ***Cacna2d2*** | 0,062898 | 0,064791 | 0,000827 | 0,058515 | 0,123115 |
| ***Prkch*** | 0,199389 | -0,01281 | 0,351147 | -0,02797 | -0,20844 |
| ***Gng7*** | -0,27504 | 0,192663 | 0,196458 | -0,16704 | 0,349811 |
| ***Calml5*** | -0,13791 | 0,271872 | -0,07333 | -0,03195 | 0,266241 |
| ***Gnas*** | 0,157345 | -0,00359 | 0,066071 | 0,077306 | -0,00319 |
| ***Gnb4*** | -0,03575 | 0,040054 | 0,183707 | 0,072581 | 0,026427 |
| ***Ncoa2*** | 0,086645 | 0,128211 | -0,00857 | 0,024341 | 0,049417 |
| ***Tgfb1i1*** | -0,18525 | 0,074049 | 0,218066 | 0,051482 | 0,070417 |
| ***Polr2d*** | -0,10217 | 0,10536 | -0,05706 | 0,189599 | 0,057752 |
| ***Prkcq*** | 0,10399 | -0,25314 | 0,039609 | 0,162102 | 0,11465 |
| ***Prkd1*** | -0,00044 | 0,078895 | -0,14158 | 0,042999 | 0,164733 |
| ***Ncoa1*** | 0,119747 | 0,011103 | -0,03914 | 0,078061 | -0,04566 |
| ***Cacna1c*** | -0,04337 | 0,022203 | 0,069706 | 0,034492 | 0,019935 |
| ***Cacna2d3*** | -0,02406 | -0,04318 | 0,034071 | -0,04901 | 0,16 |
| ***Gnat2*** | -0,21263 | 0,04314 | 0,139885 | 0,004939 | 0,09737 |
| ***Gtf2h2*** | 0,2036 | -0,11801 | 0,034466 | -0,04862 | -0,01215 |
| ***Cacng5*** | -0,24957 | 0,007045 | 0,194554 | 0,144425 | -0,05637 |
| ***Prkcb*** | -0,12369 | 0,164085 | -0,09661 | 0,022314 | 0,069768 |
| ***Gng2*** | -0,20107 | -0,08394 | 0,237358 | 0,002693 | 0,074449 |
| ***Cacna1g*** | -0,19433 | 0,273079 | 0,102597 | -0,11534 | -0,0667 |
| ***Gnb1l*** | -0,04641 | 0,105092 | -0,00544 | -0,07418 | 0,01498 |
| ***Gtf2f1*** | 0,06257 | -0,29206 | -0,03852 | 0,065163 | 0,196671 |
| ***Cacng4*** | -0,12011 | -0,20771 | 0,247698 | -0,13888 | 0,205221 |
| ***Ncoa4*** | -0,12644 | -0,14738 | 0,214951 | 0,048295 | -0,00323 |
| ***Gnat1*** | -0,35967 | -0,17673 | 0,272882 | 0,155199 | 0,074056 |
| ***Cacna1d*** | -0,16088 | 0,110339 | -0,03254 | -0,09703 | 0,138796 |
| ***Smad3*** | 0,136784 | 0,106913 | -0,0102 | -0,24429 | -0,04875 |
| ***Gnaq*** | -0,05399 | -0,01776 | 0,09043 | -0,1226 | 0,010019 |
| ***Prkci*** | 0,024551 | -0,43889 | 0,186525 | 0,082533 | 0,04227 |
| ***Polr2k*** | 0,06281 | -0,0837 | 0,155358 | -0,09311 | -0,15053 |
| ***Prkar2a*** | 0,387154 | -0,67441 | 0,07029 | 0,328312 | -0,22801 |
| ***Mapk1*** | 0,044576 | -0,16036 | 0,093211 | 0,032593 | -0,13623 |
| ***Hspa4*** | -0,01923 | -0,18069 | 0,073361 | 0,029925 | -0,0321 |
| ***Taf2*** | 0,337815 | -0,59419 | -0,13063 | 0,164712 | 0,08943 |
| ***Gng3*** | -0,03794 | -0,07169 | -0,00994 | -0,07621 | 0,030828 |
| ***Gna13*** | -0,13762 | 0,203253 | -0,0871 | -0,07987 | -0,06962 |
| ***Prkar1a*** | -0,19423 | -0,07836 | 0,018844 | 0,029118 | 0,03697 |
| ***Cacng3*** | -0,12755 | -0,11056 | 0,045502 | -0,0353 | -0,00726 |
| ***Gnb1*** | 0,00148 | -0,21466 | -0,05589 | -0,05873 | 0,075602 |
| ***Gnai3*** | -0,117 | 0,055301 | -0,06614 | -0,11181 | -0,02625 |
| ***Prkag1*** | 0,015082 | -0,1281 | -0,02855 | -0,07731 | -0,04722 |
| ***Gng12*** | -0,11917 | -0,24864 | -0,00652 | 0,053507 | 0,043562 |
| ***Gtf2h3*** | -0,09165 | -0,08701 | -0,15976 | 0,005181 | 0,041885 |
| ***Cacna1a*** | -0,13667 | 0,010883 | 0,016384 | -0,20037 | 0,014165 |
| ***Gng13*** | -0,412 | 0,419106 | -0,23984 | 0,029264 | -0,09515 |
| ***Polr2b*** | 0,017711 | -0,23013 | 0,029419 | -0,10407 | -0,02658 |
| ***Prkacb*** | -0,17137 | -0,11709 | 0,152978 | -0,07941 | -0,11836 |
| ***Gnal*** | 0,270315 | -0,25518 | -0,21388 | -0,06467 | -0,07254 |
| ***Gna11*** | 0,117817 | -0,38846 | -0,07016 | 0,053208 | -0,05876 |
| ***Prkce*** | 0,064813 | -0,4592 | -0,03295 | 0,190098 | -0,12429 |
| ***Cacna1b*** | -0,66315 | 0,077493 | 0,049701 | -0,02331 | 0,185651 |
| ***Prkaca*** | 0,087291 | -0,5 | -0,14718 | 0,014032 | 0,092124 |
| ***Gnai2*** | 0,048359 | -0,4014 | -0,14762 | -0,05658 | 0,091077 |
| ***Gtf2e1*** | -0,04778 | 0,064877 | -0,14084 | -0,2288 | -0,12571 |
| ***Gtf2h4*** | -0,17032 | -0,17835 | 0,035948 | -0,06562 | -0,1045 |
| ***Nfkb2*** | -0,12845 | -0,86625 | -0,02873 | 0,190012 | 0,325921 |
| ***Gng10*** | -0,19458 | 0,016107 | -0,21464 | -0,13453 | -0,01707 |
| ***Calr*** | 0,116609 | -0,34303 | -0,2809 | -0,0345 | -0,0133 |
| ***Cdk7*** | 0,085413 | -0,11919 | -0,05557 | -0,25004 | -0,21808 |
| ***Gtf2a1*** | -0,23657 | -0,04755 | -0,03433 | -0,15352 | -0,24815 |
| ***Kat2b*** | -0,0819 | -0,6162 | -0,13926 | 0,230563 | -0,12506 |
| ***Mapk3*** | -0,12178 | -0,2083 | -0,11816 | -0,24199 | -0,08334 |
| ***Calm1*** | -0,26531 | -0,54964 | -0,33072 | 0,183607 | 0,174919 |
| ***Polr2e*** | 0,003755 | -0,74755 | 0,071654 | -0,07255 | -0,04956 |
| ***Tbp*** | -0,22589 | 0,012902 | -0,06269 | -0,27582 | -0,25317 |
| ***Prkcz*** | 0,069884 | -0,36703 | -0,18978 | -0,14472 | -0,18557 |
| ***Shc1*** | -0,03727 | -0,27279 | -0,23371 | -0,16462 | -0,14019 |
| ***Cacnb3*** | -0,16395 | -0,08676 | -0,14855 | -0,52755 | -0,16726 |
| ***Ccnh*** | -0,34829 | -0,57279 | 0,00624 | -0,19423 | -0,26363 |
| ***Mnat1*** | 0,003048 | -0,30839 | -0,22958 | -0,43191 | -0,42815 |
| ***Prkag2*** | -0,50905 | -0,84728 | -0,13855 | -0,19606 | -0,26978 |
| ***Gng11*** | -0,49266 | -0,36549 | -0,49521 | -0,70903 | -0,48259 |
| ***Prkd3*** | -0,08954 | -0,27329 | -0,74934 | -0,84385 | -0,69256 |
| ***Prkca*** | -0,78907 | -1,45442 | -0,55646 | -0,30357 | -0,30056 |
| ***Ar*** | -1,20432 | -1,20092 | -1,31942 | -1,29383 | -1,37866 |
| ***Ccnd1*** | -2,06339 | -1,47819 | -1,8214 | -1,3163 | -0,59342 |

**Table S3:** Activation Z-score of genes involved in estrogen receptor signaling male vs. female. Activation Z-score was calculated with IPA software from Qiagen.

| **Pathway Summary** | -0,896 | -0,981 | -1,408 | 0,555 | -0,822 |
| --- | --- | --- | --- | --- | --- |
| **Genes in the estrogen receptor signaling network** | **0 h** | **24 h** | **48 h** | **72 h** | **96 h** |
| ***Fos*** | 0,477946 | 1,616629 | 1,925766 | 1,628887 | 1,742349 |
| ***Plcl1*** | -0,13783 | 0,992468 | 1,146341 | 1,245308 | 0,792632 |
| ***Pgf*** | 0,079831 | 0,30252 | 1,032182 | 1,148335 | 0,332997 |
| ***Jun*** | 0,719868 | 0,883187 | 0,549306 | 0,296255 | 0,375915 |
| ***Egfr*** | 1,511161 | 0,917405 | 0,196501 | 0,031613 | 0,151305 |
| ***Vegfa*** | -0,06545 | 0,80094 | 0,960118 | 0,861856 | 0,106438 |
| ***Cav1*** | 0,773773 | 0,658437 | 0,150822 | 0,097915 | 0,546004 |
| ***Igf1*** | -0,0711 | 0,534636 | 0,478904 | 0,342446 | 0,463394 |
| ***Prkab2*** | -0,04086 | 0,604595 | 0,730222 | 0,366204 | -0,11625 |
| ***Eif4ebp1*** | 0,246025 | 0,279086 | 0,437429 | 0,286157 | 0,203797 |
| ***Arg2*** | -0,01689 | -0,06842 | 0,759289 | 0,230589 | 0,399646 |
| ***Gnao1*** | 0,259911 | 0,201797 | 0,213923 | 0,232768 | 0,291226 |
| ***Gnai1*** | 0,24009 | 0,085879 | 0,3033 | 0,310778 | 0,25472 |
| ***Mmp9*** | 0,170831 | 0,469305 | 0,34465 | 0,001376 | 0,194084 |
| ***Creb5*** | -0,09158 | -0,29206 | 0,439232 | 0,749029 | 0,369094 |
| ***Gna12*** | 0,650449 | 0,30947 | 0,083986 | -0,01054 | 0,062736 |
| ***Mmp27*** | 0,154702 | 0,153958 | 0,388733 | 0,126761 | 0,264608 |
| ***Myl9*** | -0,19785 | 0,162461 | 0,185934 | 0,540821 | 0,3637 |
| ***Mras*** | 0,174646 | 1,003178 | -0,15429 | -0,08634 | 0,102783 |
| ***Myl7*** | -0,17735 | 0,395424 | 0,460227 | 0,328122 | 0,009238 |
| ***Prkcg*** | -0,04543 | 0,032092 | 0,447063 | 0,40637 | 0,164773 |
| ***Atf4*** | -0,07623 | 0,409375 | 0,25378 | 0,177375 | 0,221857 |
| ***Map2k1*** | 0,13442 | 0,207022 | 0,290068 | 0,177577 | 0,173668 |
| ***Map2k2*** | 0,575392 | -0,15963 | 0,11357 | 0,344424 | 0,071213 |
| ***Vegfc*** | 0,130776 | 0,258138 | 0,188709 | 0,082832 | 0,255641 |
| ***Plcb2*** | 0,164871 | 0,199211 | 0,118978 | 0,264859 | 0,1516 |
| ***Med31*** | 0,201209 | 0,811269 | 0,285495 | -0,19289 | -0,22687 |
| ***Plcd3*** | 0,154834 | 0,323924 | 0,119373 | 0,10714 | 0,169208 |
| ***Med13l*** | 0,10614 | 0,269185 | 0,221961 | 0,298574 | -0,02243 |
| ***Esr2*** | -0,00057 | 0,018629 | 0,301336 | 0,113546 | 0,433366 |
| ***Myc*** | 0,149033 | 0,077665 | 0,227367 | 0,168713 | 0,230961 |
| ***Mmp10*** | 0,143721 | -0,0084 | 0,263216 | 0,455295 | -0,03518 |
| ***Med13*** | 0,168915 | 0,092668 | 0,386759 | 0,324642 | -0,15625 |
| ***Mmp21*** | 0,160533 | 0,534537 | 0,103597 | -0,20013 | 0,18506 |
| ***Nr0b1*** | 0,245271 | -0,13649 | 0,204394 | 0,173183 | 0,290748 |
| ***Sos1*** | 0,264454 | 0,128647 | 0,077278 | 0,247009 | 0,056206 |
| ***Mmp24*** | 0,186939 | -0,01756 | 0,075556 | 0,147056 | 0,366768 |
| ***Nrf1*** | 0,26316 | 0,739826 | -0,055 | -0,01539 | -0,19097 |
| ***Cacna1s*** | 0,045355 | 0,098768 | 0,128293 | 0,065133 | 0,402048 |
| ***Ncor2*** | -0,03862 | 0,293661 | 0,141303 | 0,190168 | 0,142945 |
| ***She*** | 0,021152 | 0,025454 | 0,4008 | 0,069498 | 0,212083 |
| ***Gna15*** | 0,11704 | 0,128682 | 0,186349 | -0,04242 | 0,334794 |
| ***Eif2b3*** | 0,307242 | 0,022034 | 0,232106 | 0,138004 | 0,016692 |
| ***Eif2b4*** | 0,364693 | 0,271258 | 0,120752 | -0,04479 | -0,00174 |
| ***Prkcd*** | 0,074277 | 0,348892 | 0,246503 | 0,028518 | 0,008762 |
| ***Nfkb1*** | 0,158929 | 0,198966 | -0,05669 | 0,144947 | 0,239545 |
| ***Myl6b*** | 0,01091 | 0,284951 | 0,315639 | -0,05845 | 0,122485 |
| ***Sp1*** | 0,094922 | 0,310516 | 0,068808 | 0,047906 | 0,139302 |
| ***Rela*** | 0,172055 | 0,224581 | 0,095794 | 0,07538 | 0,090153 |
| ***Mdk*** | -0,02083 | 0,148577 | 0,207104 | 0,056588 | 0,246794 |
| ***Ganz*** | 0,193874 | 0,107859 | 0,148681 | 0,126389 | 0,058139 |
| ***Prkar1b*** | -0,0144 | 0,23196 | 0,228917 | 0,084269 | 0,096835 |
| ***Cacna1e*** | 0,129002 | -0,09285 | 0,004205 | 0,18798 | 0,395057 |
| ***Jak1*** | 0,143002 | 0,289012 | 0,123497 | -0,0766 | 0,130972 |
| ***Med12l*** | 0,243158 | 0,114068 | -0,01262 | 0,117037 | 0,142669 |
| ***Hsp90aa1*** | 0,502608 | -0,32229 | 0,178781 | 0,094177 | 0,142744 |
| ***Myl6*** | 0,26253 | -0,31051 | 0,040774 | 0,301868 | 0,286839 |
| ***Rala*** | 0,0355 | 0,107634 | 0,179678 | 0,155122 | 0,08952 |
| ***Rasd2*** | -0,06273 | 0,27061 | 0,160291 | -0,01052 | 0,20936 |
| ***Setd7*** | 0,01817 | 0,139424 | 0,451778 | 0,047215 | -0,0914 |
| ***Eif2b2*** | 0,046137 | -0,05748 | 0,297701 | 0,1082 | 0,168816 |
| ***Sod2*** | 0,112468 | 0,368751 | 0,010485 | 0,083511 | -0,01597 |
| ***Med21*** | 0,161681 | 0,086849 | 0,168733 | 0,050556 | 0,08612 |
| ***Med10*** | 0,122237 | 0,313462 | 0,12559 | -0,07374 | 0,053827 |
| ***Ralb*** | 0,014308 | 0,182199 | -0,06845 | 0,121461 | 0,291222 |
| ***Mmp17*** | 0,042256 | -0,06456 | 0,208697 | 0,070272 | 0,281833 |
| ***Snai1*** | 0,241185 | -0,03282 | 0,165691 | -0,1183 | 0,276763 |
| ***Ncoa3*** | 0,336911 | 0,236946 | -0,20161 | 0,16127 | -0,00466 |
| ***Foxg1*** | -0,03995 | 0,201586 | 0,164909 | 0,050022 | 0,150193 |
| ***Gng5*** | 0,25983 | -0,08528 | 0,135085 | 0,151036 | 0,056332 |
| ***Eif4e*** | 0,139717 | -0,22717 | 0,308365 | 0,238362 | 0,041298 |
| ***Gsk3b*** | 0,155658 | 0,198252 | 0,01772 | 0,058219 | 0,059877 |
| ***Adcy5*** | 0,074304 | 0,336537 | -0,07785 | -0,02083 | 0,177293 |
| ***Med4*** | 0,177843 | 0,456701 | 0,132076 | -0,09318 | -0,18433 |
| ***Pik3c2a*** | 0,005251 | -0,19112 | 0,220942 | 0,330514 | 0,110934 |
| ***Plcg1*** | 0,076618 | 0,17445 | 0,163278 | -0,05111 | 0,113243 |
| ***Runx2*** | -0,01584 | 0,087844 | 0,167739 | 0,045434 | 0,189704 |
| ***Prkar2b*** | -0,27859 | 0,108796 | 0,194686 | 0,282446 | 0,160946 |
| ***Adcy9*** | 0,130001 | 0,275745 | 0,114288 | 0,033052 | -0,09261 |
| ***Pik3r5*** | -0,01583 | 0,191325 | 0,133672 | -0,09727 | 0,247275 |
| ***Igf1r*** | -0,0981 | -0,0826 | 0,279362 | 0,110573 | 0,242523 |
| ***Adcy2*** | 0,06202 | -0,10133 | 0,210394 | 0,138832 | 0,132861 |
| ***Crebbp*** | 0,186457 | 0,187408 | -0,11777 | 0,234118 | -0,05094 |
| ***Mmp11*** | 0,111364 | 0,177752 | 0,023179 | 0,02362 | 0,099515 |
| ***Sra1*** | 0,225891 | 0,076737 | 0,150775 | -0,00428 | -0,01777 |
| ***Atp5f1d*** | 0,120078 | 0,12668 | 0,071705 | 0,042124 | 0,061662 |
| ***Limk2*** | -0,08704 | 0,181881 | 0,222302 | 0,056749 | 0,035087 |
| ***Adcy8*** | 0,231949 | 0,077836 | 0,007572 | 0,184924 | -0,09647 |
| ***Gna14*** | 0,170907 | 0,213407 | -0,01014 | -0,10352 | 0,129124 |
| ***Pik3c2b*** | 0,118141 | 0,169816 | 0,018153 | 0,04807 | 0,042186 |
| ***Eif2b5*** | 0,052381 | -0,13781 | 0,320581 | 0,041547 | 0,11536 |
| ***H2bw2*** | 0,021567 | 0,128356 | 0,049833 | 0,205873 | -0,01419 |
| ***Mmp8*** | 0,202417 | 0,023617 | 0,11484 | -0,13426 | 0,18465 |
| ***Prkag3*** | 0,069459 | 0,009306 | -0,05614 | 0,105657 | 0,258308 |
| ***Pik3r6*** | -0,14476 | 0,123504 | 0,067777 | 0,110262 | 0,229527 |
| ***Foxo3*** | 0,150165 | 0,280371 | 0,170242 | -0,06662 | -0,14944 |
| ***Med17*** | 0,17807 | -0,06838 | 0,148112 | 0,005444 | 0,120114 |
| ***Mmp1*** | 0,175626 | -0,07255 | 0,158068 | 0,024595 | 0,095549 |
| ***Gnb3*** | -0,03474 | 0,049647 | 0,092079 | 0,011347 | 0,238027 |
| ***Raf1*** | 0,095374 | 0,446542 | -0,06254 | 0,026048 | -0,14962 |
| ***Adcy1*** | -0,07322 | -0,12335 | 0,320176 | 0,346676 | -0,11667 |
| ***Ep300*** | 0,223775 | -0,03702 | 0,045473 | 0,279534 | -0,16359 |
| ***Mmp2*** | -0,14215 | 0,204659 | 0,059294 | 0,059904 | 0,162377 |
| ***Mmp3*** | -0,16124 | 0,212344 | 0,10715 | 0,13371 | 0,043673 |
| ***Mylpf*** | -0,08363 | -0,19368 | 0,162583 | 0,246486 | 0,201406 |
| ***Cdkn1a*** | -0,38236 | -0,08342 | 0,323505 | 0,230924 | 0,241859 |
| ***Prok1*** | -0,21473 | -0,00834 | 0,20743 | 0,054793 | 0,287334 |
| ***Atp5f1c*** | 0,168399 | 0,16927 | 0,075252 | -0,00083 | -0,08907 |
| ***Rps6kb2*** | -0,03822 | 0,022134 | -0,02301 | 0,169163 | 0,185489 |
| ***Src*** | -0,27544 | 0,024078 | 0,202661 | 0,159584 | 0,199357 |
| ***Hras*** | 0,40146 | -0,23578 | -0,11532 | 0,234695 | 0,023973 |
| ***Prkch*** | 0,199389 | -0,01281 | 0,351147 | -0,02797 | -0,20844 |
| ***Gng7*** | -0,27504 | 0,192663 | 0,196458 | -0,16704 | 0,349811 |
| ***Gnas*** | 0,157345 | -0,00359 | 0,066071 | 0,077306 | -0,00319 |
| ***Ncoa2*** | 0,086645 | 0,128211 | -0,00857 | 0,024341 | 0,049417 |
| ***Rasd1*** | 0,052517 | 0,084486 | 0,002894 | 0,137336 | -0,00633 |
| ***Mmp15*** | 0,690075 | 0,33487 | -0,71809 | -0,09848 | 0,028916 |
| ***Plcg2*** | 0,245478 | 0,197959 | -0,16078 | -0,02148 | -0,03416 |
| ***Pelp1*** | 0,159701 | 0,004144 | 0,0619 | 0,096605 | -0,10606 |
| ***Ppp1r12a*** | 0,098903 | -0,20144 | 0,063026 | 0,192264 | 0,039148 |
| ***Creb3l3*** | 0,384907 | 0,539266 | -0,04744 | -0,33531 | -0,35437 |
| ***Ddx5*** | -0,01649 | 0,094396 | 0,154655 | -0,12105 | 0,074741 |
| ***Gsk3a*** | 0,079988 | -0,12934 | -0,0048 | 0,275579 | -0,03598 |
| ***Cyc1*** | 0,081219 | 0,212579 | -0,13183 | -0,05768 | 0,074994 |
| ***Mmp16*** | 0,017706 | -0,01558 | 0,05142 | 0,093415 | 0,030836 |
| ***Mmp28*** | -0,34175 | 0,218897 | 0,123572 | 0,036782 | 0,137211 |
| ***Ncor1*** | 0,187013 | 0,035806 | -0,0549 | 0,083873 | -0,08182 |
| ***Prkcq*** | 0,10399 | -0,25314 | 0,039609 | 0,162102 | 0,11465 |
| ***Igf2*** | -0,21875 | 0,049443 | 0,216651 | 0,054757 | 0,062983 |
| ***Plcb3*** | 0,100853 | 0,006733 | -0,07192 | 0,02189 | 0,106291 |
| ***Myl2*** | 0,013482 | 0,080843 | -0,03369 | 0,05337 | 0,040183 |
| ***Lep*** | -0,21615 | 0,092485 | 0,031865 | -0,17527 | 0,419314 |
| ***Mmp25*** | 0,136796 | 0,001625 | -0,11193 | 0,118484 | 0,002082 |
| ***Uqcrfs1*** | 0,090576 | 0,109382 | -0,0161 | -0,09273 | 0,054135 |
| ***Prkd1*** | -0,00044 | 0,078895 | -0,14158 | 0,042999 | 0,164733 |
| ***Myl10*** | 0,329828 | -0,07041 | -0,20281 | -0,07408 | 0,158855 |
| ***Pik3r2*** | 0,25572 | -0,09114 | 0,075034 | -0,03299 | -0,0685 |
| ***Notum*** | -0,09845 | 0,247083 | -0,09462 | -0,04298 | 0,115692 |
| ***Shf*** | 0,070188 | 0,038728 | 0,169236 | 0,039846 | -0,19247 |
| ***Ncoa1*** | 0,119747 | 0,011103 | -0,03914 | 0,078061 | -0,04566 |
| ***Bad*** | 0,064966 | 0,013672 | 0,015555 | 0,017132 | 0,001594 |
| ***Nr3c1*** | -0,18234 | 0,22316 | 0,147401 | 0,048842 | -0,12813 |
| ***Pgr*** | -0,14119 | -0,02806 | -0,03265 | 0,179473 | 0,128728 |
| ***Akt3*** | -0,24716 | -0,00756 | 0,243473 | 0,466583 | -0,35147 |
| ***Cacna1c*** | -0,04337 | 0,022203 | 0,069706 | 0,034492 | 0,019935 |
| ***Adcy3*** | -0,0185 | -0,08074 | 0,055628 | -0,02652 | 0,157862 |
| ***Rock1*** | 0,046289 | 0,044236 | -0,11688 | 0,116376 | -0,00638 |
| ***Mmp13*** | -0,12614 | 0,14188 | -0,15341 | 0,18128 | 0,034376 |
| ***Nras*** | -0,01512 | -0,20668 | 0,095093 | 0,160952 | 0,042765 |
| ***Gnat2*** | -0,21263 | 0,04314 | 0,139885 | 0,004939 | 0,09737 |
| ***Notch1*** | -0,05018 | 0,291783 | -0,19438 | 0,021287 | -0,01234 |
| ***Gper1*** | 0,008833 | -0,02067 | 0,041111 | -0,15409 | 0,170554 |
| ***Plch2*** | -0,12351 | 0,028089 | 0,009241 | 0,052869 | 0,077138 |
| ***Plcd1*** | -0,22994 | 0,19987 | 0,134655 | -0,07614 | 0,012606 |
| ***Prkcb*** | -0,12369 | 0,164085 | -0,09661 | 0,022314 | 0,069768 |
| ***Hsp90ab1*** | 0,153701 | -0,42093 | 0,171052 | 0,082244 | 0,047582 |
| ***Foxo6*** | 0,045591 | -0,09656 | -0,10833 | 0,087444 | 0,101788 |
| ***Gng2*** | -0,20107 | -0,08394 | 0,237358 | 0,002693 | 0,074449 |
| ***Trim63*** | -0,30558 | -0,13588 | 0,367515 | 0,053345 | 0,044612 |
| ***Gps2*** | 0,059005 | 0,039593 | -0,00723 | -0,06048 | -0,01434 |
| ***Mmp23b*** | -0,09272 | 0,323873 | -0,0948 | -0,07264 | -0,05072 |
| ***Myl3*** | -0,11593 | -0,02584 | 0,02481 | -0,08367 | 0,203004 |
| ***Atf2*** | 0,06989 | 0,196257 | -0,04385 | -0,06945 | -0,15106 |
| ***Foxo1*** | -0,14653 | -0,02777 | 0,134011 | 0,162362 | -0,1237 |
| ***Hes1*** | 0,317611 | 0,027997 | -0,20315 | -0,19163 | 0,03863 |
| ***Sdhd*** | 0,008542 | 0,297357 | -0,06897 | -0,16143 | -0,09867 |
| ***Myl12b*** | 0,160155 | -0,27286 | -0,00157 | -0,0224 | 0,112726 |
| ***Rap1b*** | -0,10425 | 0,078944 | -0,00589 | 0,027729 | -0,0215 |
| ***Ctbp1*** | 0,168556 | -0,15709 | -0,17891 | 0,105041 | 0,033866 |
| ***Gnat1*** | -0,35967 | -0,17673 | 0,272882 | 0,155199 | 0,074056 |
| ***Plcb4*** | -0,04048 | 0,006694 | -0,11092 | -0,05022 | 0,159453 |
| ***Cacna1d*** | -0,16088 | 0,110339 | -0,03254 | -0,09703 | 0,138796 |
| ***Limk1*** | -0,20807 | 0,11117 | 0,037354 | 0,093216 | -0,08063 |
| ***Nrip1*** | 0,062801 | -0,10827 | 0,210467 | -0,17986 | -0,03364 |
| ***Pik3r3*** | 0,293126 | -0,01107 | -0,15181 | -0,15931 | -0,02902 |
| ***Rps6kb1*** | 0,108294 | -0,20059 | 0,049206 | 0,088938 | -0,10768 |
| ***Mmp7*** | -0,03684 | -0,08836 | -0,06039 | 0,172551 | -0,05283 |
| ***Mmp20*** | -0,1936 | -0,06276 | -0,16886 | 0,039217 | 0,318111 |
| ***Sos2*** | -0,26722 | 0,121053 | 0,169366 | 0,096972 | -0,19058 |
| ***Vegfb*** | -0,24608 | -0,57535 | 0,056054 | 0,403197 | 0,282737 |
| ***Mmp1b*** | 0,123068 | -0,00822 | -0,0512 | -0,05909 | -0,09565 |
| ***Gnaq*** | -0,05399 | -0,01776 | 0,09043 | -0,1226 | 0,010019 |
| ***Adcy7*** | 0,064468 | -0,03082 | -0,1425 | 0,061201 | -0,04773 |
| ***Trrap*** | 0,085796 | 9,18E-06 | -0,23682 | 0,047971 | 0,00075 |
| ***Prkci*** | 0,024551 | -0,43889 | 0,186525 | 0,082533 | 0,04227 |
| ***Egf*** | 0,126055 | -0,10657 | 0,172765 | -0,27676 | -0,02371 |
| ***Myl1*** | -0,24247 | -0,01812 | 0,04039 | 0,056037 | 0,051839 |
| ***Prkar2a*** | 0,387154 | -0,67441 | 0,07029 | 0,328312 | -0,22801 |
| ***Mapk1*** | 0,044576 | -0,16036 | 0,093211 | 0,032593 | -0,13623 |
| ***Pik3ca*** | 0,052469 | 0,079205 | -0,0719 | -0,11625 | -0,08746 |
| ***Rap2a*** | 0,51035 | -0,04705 | -0,10372 | -0,20105 | -0,30269 |
| ***Med15*** | 0,039891 | -0,01099 | -0,07611 | -0,10938 | 0,011848 |
| ***Uqcrc2*** | 0,01038 | 0,036687 | 0,067572 | -0,22193 | -0,04503 |
| ***Atp5f1a*** | -0,0042 | 0,071387 | -0,07363 | -0,07722 | -0,06885 |
| ***Shc3*** | -0,2422 | 0,020647 | 0,046693 | -0,00608 | 0,022539 |
| ***Cfl1*** | 0,097439 | -0,53466 | -0,00671 | 0,172816 | 0,111207 |
| ***Ctbp2*** | -0,19876 | 0,122534 | 0,114086 | -0,24674 | 0,04575 |
| ***Plcd4*** | 0,050075 | -0,09895 | 0,070874 | -0,36691 | 0,18131 |
| ***Atp5mc1*** | -0,17897 | 0,144758 | -0,07167 | 0,03197 | -0,09219 |
| ***Grb2*** | -0,06551 | 0,079172 | -0,07072 | -0,06606 | -0,04439 |
| ***Med16*** | 0,086434 | 0,066059 | -0,23461 | -0,04622 | -0,03926 |
| ***Med27*** | 0,108174 | -0,06303 | -0,00457 | -0,13606 | -0,07292 |
| ***Gna13*** | -0,13762 | 0,203253 | -0,0871 | -0,07987 | -0,06962 |
| ***Rock2*** | 0,295396 | -0,29271 | -0,18228 | -0,03032 | 0,026191 |
| ***Prkar1a*** | -0,19423 | -0,07836 | 0,018844 | 0,029118 | 0,03697 |
| ***Carm1*** | -0,019 | -0,18951 | -0,07254 | 0,041051 | 0,052087 |
| ***Pik3cd*** | -0,26532 | 0,047348 | 0,051176 | 0,075089 | -0,11023 |
| ***Jak3*** | -0,13502 | 0,15999 | -0,01889 | -0,13616 | -0,07921 |
| ***Foxo4*** | 0,125601 | -0,17031 | -0,18312 | 0,067529 | -0,07672 |
| ***Adcy10*** | -0,35885 | -0,06087 | 0,064495 | 0,079042 | 0,026704 |
| ***Gnb1*** | 0,00148 | -0,21466 | -0,05589 | -0,05873 | 0,075602 |
| ***Plcz1*** | -0,03042 | -0,04838 | -0,01187 | 0,080702 | -0,24691 |
| ***Hdac3*** | -0,11855 | 0,019597 | -0,11064 | -0,07235 | 0,019299 |
| ***Gnai3*** | -0,117 | 0,055301 | -0,06614 | -0,11181 | -0,02625 |
| ***Prkag1*** | 0,015082 | -0,1281 | -0,02855 | -0,07731 | -0,04722 |
| ***Cdk8*** | -0,04514 | -0,20221 | 0,085983 | 0,112521 | -0,22881 |
| ***Rhoa*** | -0,13676 | -0,1495 | 0,013621 | -0,03237 | 0,023586 |
| ***Cacna1a*** | -0,13667 | 0,010883 | 0,016384 | -0,20037 | 0,014165 |
| ***Rras*** | -0,04157 | -0,40855 | -0,11514 | 0,07881 | 0,186698 |
| ***Pcna*** | -0,07526 | -0,05098 | -0,20175 | 0,001387 | 0,01912 |
| ***Fbxo32*** | 0,176168 | -0,01185 | 0,10271 | -0,20768 | -0,36688 |
| ***Polr2b*** | 0,017711 | -0,23013 | 0,029419 | -0,10407 | -0,02658 |
| ***Pik3r1*** | 0,090286 | -0,57779 | -0,2358 | 0,144529 | 0,263983 |
| ***Atp5pb*** | -0,0982 | -0,08772 | 0,045613 | -0,05532 | -0,12723 |
| ***Prkacb*** | -0,17137 | -0,11709 | 0,152978 | -0,07941 | -0,11836 |
| ***Ccnc*** | 0,010763 | -0,31776 | 0,189612 | 0,074324 | -0,29186 |
| ***Gnal*** | 0,270315 | -0,25518 | -0,21388 | -0,06467 | -0,07254 |
| ***Gna11*** | 0,117817 | -0,38846 | -0,07016 | 0,053208 | -0,05876 |
| ***Thrap3*** | -0,28543 | 0,244072 | -0,21347 | -0,11569 | 0,021659 |
| ***Prkce*** | 0,064813 | -0,4592 | -0,03295 | 0,190098 | -0,12429 |
| ***Plch1*** | 0,099831 | -0,20385 | -0,23486 | -0,02397 | -0,00027 |
| ***Akt2*** | -0,09815 | -0,15659 | -0,00876 | -0,09686 | -0,01344 |
| ***Adcy4*** | -0,21526 | 0,114692 | -0,06 | -0,10196 | -0,1173 |
| ***Med12*** | 0,161727 | -0,32009 | -0,08208 | -0,0048 | -0,1346 |
| ***Pik3cg*** | -0,02541 | -0,17348 | -0,01282 | 0,058653 | -0,23801 |
| ***Eif2b1*** | 0,012587 | -0,27529 | 0,010685 | -0,07553 | -0,0732 |
| ***Pdia3*** | 0,289203 | -0,42388 | -0,27819 | 0,026608 | -0,02869 |
| ***Myl12a*** | 0,035807 | -0,36587 | -0,09805 | -0,13065 | 0,116878 |
| ***Nr0b2*** | -0,35622 | 0,233567 | 0,047325 | -0,39205 | 0,021575 |
| ***Pik3r4*** | -0,00762 | 0,057253 | -0,08123 | -0,32561 | -0,09624 |
| ***Prkaca*** | 0,087291 | -0,5 | -0,14718 | 0,014032 | 0,092124 |
| ***Med 30*** | -0,23184 | -0,04694 | -0,00355 | -0,11546 | -0,05761 |
| ***Gnai2*** | 0,048359 | -0,4014 | -0,14762 | -0,05658 | 0,091077 |
| ***Myl4*** | -0,25208 | 0,020341 | -0,16735 | -0,17303 | 0,096478 |
| ***Creb1*** | 0,006401 | -0,28437 | -0,02665 | 0,017619 | -0,19909 |
| ***Nos3*** | -0,44887 | 0,068903 | -0,05055 | -0,06996 | 0,013571 |
| ***Cfl2*** | -0,24406 | -0,24609 | -0,02235 | -0,01607 | 0,024846 |
| ***Pik3c3*** | -0,23607 | 0,073196 | -0,14118 | -0,14439 | -0,05641 |
| ***Med6*** | -0,11811 | -0,17516 | -0,0774 | -0,07797 | -0,05723 |
| ***Nfkb2*** | -0,12845 | -0,86625 | -0,02873 | 0,190012 | 0,325921 |
| ***Mprip*** | 0,219189 | -0,31521 | -0,24068 | -0,07014 | -0,10076 |
| ***Prkaa1*** | -0,18974 | -0,09698 | -0,11026 | -0,10454 | -0,04678 |
| ***Zdhhc21*** | -0,20251 | -0,53057 | 0,116844 | 0,069682 | -0,01325 |
| ***Prkaa2*** | 0,026958 | -0,16209 | -0,16135 | -0,10442 | -0,16972 |
| ***Pten*** | -0,06088 | -0,2313 | -0,00484 | -0,14557 | -0,13593 |
| ***Hif1a*** | -0,06375 | -0,27724 | -0,25817 | 0,067145 | -0,05719 |
| ***Med18*** | -0,21043 | 0,006515 | -0,16771 | -0,05061 | -0,16909 |
| ***Vegfd*** | 0,160988 | -0,32227 | -0,25565 | -0,13087 | -0,05376 |
| ***Creb3L4*** | -0,26597 | -0,33073 | -0,06454 | -0,03184 | 0,091149 |
| ***Akt1*** | -0,12742 | -0,47145 | -0,12531 | -0,0356 | 0,152021 |
| ***Med24*** | 0,041867 | -0,28144 | -0,08282 | -0,37162 | 0,063763 |
| ***Ppp1cb*** | -0,1511 | -0,07912 | -0,12337 | -0,16331 | -0,14493 |
| ***Plcl2*** | -0,14263 | 0,227116 | -0,1606 | -0,32853 | -0,25868 |
| ***Hnrnpd*** | -0,46242 | -0,39908 | 0,142871 | 0,119328 | -0,06451 |
| ***Rap2b*** | -0,53235 | 0,177452 | 0,098047 | -0,30301 | -0,10827 |
| ***Tyk2*** | 0,010038 | -0,2758 | -0,26825 | -0,17562 | 0,040721 |
| ***Shc2*** | -0,31489 | 0,003963 | -0,24513 | -0,04371 | -0,08298 |
| ***Med1*** | 0,053763 | -0,41944 | -0,10408 | -0,04778 | -0,19516 |
| ***Sdhc*** | -0,19668 | -0,06477 | -0,12149 | -0,13121 | -0,20328 |
| ***Tbl1xr1*** | -0,02301 | -0,42303 | -0,1496 | -0,08297 | -0,05581 |
| ***Jak2*** | -0,02781 | -0,30249 | -0,25278 | 0,041496 | -0,19887 |
| ***Hsp90b1*** | -0,02465 | -0,28963 | -0,1934 | -0,04917 | -0,19621 |
| ***Igf2r*** | 0,130989 | -0,77769 | -0,06123 | -0,1016 | 0,054202 |
| ***Creb3*** | 0,118507 | -0,56062 | -0,25304 | -0,11912 | 0,049329 |
| ***Plcb1*** | 0,41932 | -0,57034 | -0,37457 | -0,19463 | -0,05126 |
| ***Mapk3*** | -0,12178 | -0,2083 | -0,11816 | -0,24199 | -0,08334 |
| ***Prkcz*** | 0,069884 | -0,36703 | -0,18978 | -0,14472 | -0,18557 |
| ***Zdhhc7*** | 0,042912 | -0,3461 | -0,09452 | -0,20362 | -0,22119 |
| ***Plce1*** | -0,04291 | -0,48522 | -0,17329 | 0,048268 | -0,17428 |
| ***Shc1*** | -0,03727 | -0,27279 | -0,23371 | -0,16462 | -0,14019 |
| ***Adcy6*** | 0,222205 | -0,25295 | -0,48268 | -0,21092 | -0,13144 |
| ***Rap1a*** | -0,09409 | -0,14484 | -0,13954 | -0,2421 | -0,26123 |
| ***Dlg4*** | -0,50511 | -0,05985 | -0,35011 | 0,009915 | 0,019942 |
| ***Tfam*** | -0,20041 | -0,43833 | 0,040577 | 0,053657 | -0,34895 |
| ***Eras*** | -0,10103 | -0,09304 | -0,48438 | -0,19299 | -0,02703 |
| ***Med23*** | 0,00227 | -0,51316 | -0,11281 | -0,13799 | -0,14443 |
| ***Mmp19*** | -0,24902 | -0,38276 | -0,0365 | -0,15449 | -0,08493 |
| ***Ppp1r12b*** | 0,005695 | -0,33049 | -0,06602 | -0,15372 | -0,44367 |
| ***Mtor*** | 0,012855 | -0,15939 | -0,11537 | -0,44566 | -0,29787 |
| ***Kras*** | -0,26815 | -0,15401 | -0,19245 | -0,26561 | -0,23842 |
| ***Pik3c2g*** | -0,48782 | -0,08919 | -0,10013 | -0,11043 | -0,35399 |
| ***Prkab1*** | -0,21304 | 0,004241 | -0,24166 | -0,39474 | -0,30941 |
| ***Mcu*** | -0,44396 | -0,53601 | -0,26586 | -0,09492 | 0,129141 |
| ***Med14*** | -0,17404 | -0,72364 | -0,36392 | 0,035374 | -0,04465 |
| ***Mmp12*** | -0,17724 | -0,01985 | 0,177688 | -0,42788 | -0,84604 |
| ***Pik3cb*** | -0,21367 | -0,8372 | -0,21036 | -0,02392 | -0,02048 |
| ***Agt*** | -0,32361 | 0,085077 | -0,52515 | -0,41596 | -0,29566 |
| ***Foxa1*** | 0,558808 | -0,8413 | -0,40635 | -0,48428 | -0,32452 |
| ***Tp53*** | -0,03313 | -0,694 | -0,51593 | -0,13647 | -0,15904 |
| ***Rbfox2*** | -0,47246 | -0,39998 | -0,196 | -0,39742 | -0,0785 |
| ***Prkdc*** | -0,00705 | -0,58037 | -0,49181 | -0,35542 | -0,12027 |
| ***Esr1*** | -0,8671 | 0,042478 | -0,57455 | -0,17213 | -0,09752 |
| ***Rras2*** | -0,07237 | -0,80744 | -0,56238 | -0,24113 | -0,04987 |
| ***Bcl2*** | -0,06053 | -0,19298 | -1,1254 | -0,45546 | 0,040528 |
| ***Mmp14*** | -0,92601 | -0,38032 | -0,41517 | -0,10832 | -0,07255 |
| ***Prkag2*** | -0,50905 | -0,84728 | -0,13855 | -0,19606 | -0,26978 |
| ***Gng11*** | -0,49266 | -0,36549 | -0,49521 | -0,70903 | -0,48259 |
| ***Prkd3*** | -0,08954 | -0,27329 | -0,74934 | -0,84385 | -0,69256 |
| ***Ppargc1a*** | -0,79412 | -0,38501 | -0,49487 | -0,42794 | -0,66493 |
| ***Prkca*** | -0,78907 | -1,45442 | -0,55646 | -0,30357 | -0,30056 |
| ***Pdgfc*** | -0,24588 | -0,79194 | -0,87006 | -1,05117 | -0,55016 |
| ***Pak1*** | -0,58386 | -1,52828 | -1,52914 | -0,77541 | -0,55814 |
| ***Lepr*** | -1,18575 | -0,92697 | -0,63828 | -1,08262 | -1,81091 |
| ***Ccnd1*** | -2,06339 | -1,47819 | -1,8214 | -1,3163 | -0,59342 |

**Table S4:** Activation Z-score of genes involved in noradrenaline and adrenaline degradation male vs. female. Activation Z-score was calculated with IPA software from Qiagen.

| **Pathway Summary** | -0,905 | 1,387 | -2,138 | -2,828 | -2,121 |
| --- | --- | --- | --- | --- | --- |
| **genes in the noradrenaline and adrenaline degradation network** | **0 h** | **24 h** | **48 h** | **72 h** | **96 h** |
| ***Adh4*** | 0,843657 | 1,099951 | -0,02072 | -0,12201 | -0,06158 |
| ***Aldh4a1*** | -0,18994 | 1,145047 | 0,371212 | 0,082255 | -0,3707 |
| ***Smox*** | -0,38514 | 0,265107 | 0,58581 | 0,252859 | 0,094358 |
| ***Adh5*** | 0,051015 | 0,302692 | 0,253513 | 0,082737 | 0,112555 |
| ***Akr1a1*** | 0,332914 | 0,263026 | -0,02438 | -0,09479 | -0,01213 |
| ***Aldh3a1*** | -0,01797 | 0,141252 | 0,02739 | 0,166023 | 0,14445 |
| ***Aldh7a1*** | 0,220354 | 0,819824 | -0,32258 | -0,21583 | -0,08076 |
| ***Adhfe1*** | 0,120446 | 0,657312 | 0,167675 | -0,18753 | -0,33779 |
| ***Adh6a*** | -0,09923 | 0,191731 | -0,05508 | 0,066197 | 0,147457 |
| ***Aldh1a2*** | -0,04424 | 0,074551 | -0,0644 | 0,130846 | 0,115865 |
| ***Aldh3b2*** | -0,23295 | 0,191515 | 0,047374 | -0,23983 | 0,346124 |
| ***Pnmt*** | -0,03446 | -0,21127 | 0,091832 | 0,074225 | 0,083498 |
| ***Lrtomt*** | -0,40908 | 0,276152 | -0,00819 | -0,01883 | 0,160188 |
| ***Comt*** | 0,338135 | -0,06313 | -0,25532 | -0,2088 | -0,03251 |
| ***Adh7*** | -0,01942 | 0,083544 | -0,20337 | 0,123429 | -0,29415 |
| ***Aldh2*** | -0,05226 | 0,209599 | -0,10349 | -0,25722 | -0,14803 |
| ***Dhrs4*** | 0,191709 | 0,457548 | -0,36069 | -0,35123 | -0,30604 |
| ***Adh1c*** | -0,16409 | 0,881367 | -0,32695 | -0,40502 | -0,41997 |
| ***Aldh3b1*** | -0,26231 | 0,03008 | -0,35622 | 0,084234 | 0,031547 |
| ***Aldh3b3*** | -0,98556 | 0,053856 | 0,106851 | 0,059383 | 0,170798 |
| ***Aldh1a3*** | -0,15936 | -0,26665 | -0,27743 | -0,07322 | 0,015636 |
| ***Aldh9a1*** | -0,30241 | 0,092659 | -0,28733 | -0,27964 | -0,1646 |
| ***Aldh1a1*** | 0,149176 | -0,68979 | -0,46523 | -0,18232 | 0,010048 |
| ***Aldh1b1*** | -0,4054 | -0,14953 | -0,38866 | -0,21044 | -0,2086 |
| ***Dhrs9*** | -0,26926 | -0,47193 | -0,06055 | -0,13292 | -0,43896 |
| ***Aldh3a2*** | -0,07275 | -0,76701 | -0,39468 | -0,41599 | -0,25463 |
| ***Maob*** | -0,68574 | -0,33792 | -0,72165 | -0,47743 | -0,326 |
| ***Maoa*** | -0,70329 | -0,4951 | -1,32169 | -1,29354 | -0,54919 |

**Table S5:** Activation Z-score of genes involved in melatonin degradation male vs. female. Activation Z-score was calculated with IPA software from Qiagen.

| **Pathway Summary** | -1,5 | 0 | 0,5 | -1,606 | -3,357 |
| --- | --- | --- | --- | --- | --- |
| **genes in the melatonin degradation I network** | **0 h** | **24 h** | **48 h** | **72 h** | **96 h** |
| ***Ugt2b17*** | 0,557028 | 2,213161 | 1,292475 | 0,591189 | -0,39135 |
| ***Cyp2e1*** | 0,155034 | 0,940182 | 2,273941 | 0,984777 | -0,50221 |
| ***Cyp2u1*** | 1,499901 | 0,704252 | 0,276107 | 0,353724 | 0,40437 |
| ***Cyp1a2*** | 0,066898 | 1,509168 | 1,215758 | 0,619908 | -1,80321 |
| ***Ugt2b28*** | 0,774388 | 0,659801 | 0,273055 | -0,19352 | -0,16884 |
| ***Cyp2s1*** | -0,07623 | -0,17694 | 0,083406 | 0,723848 | 0,266478 |
| ***Cyp1a1*** | -0,0377 | 0,221035 | 0,38464 | 0,078008 | 0,130361 |
| ***Por*** | -0,22385 | 0,116112 | 0,419562 | 0,346398 | -0,05581 |
| ***Ugt2b7*** | 0,55257 | -0,32078 | 0,463908 | -0,14661 | -0,11281 |
| ***Cyp2f1*** | 0,118274 | 1,192971 | 0,421422 | -0,61217 | -0,78225 |
| ***Cyp19a1*** | -0,01045 | 0,047247 | 0,095659 | -0,00655 | 0,153157 |
| ***B3gat3*** | 0,044436 | -0,0449 | -0,00396 | 0,117245 | 0,087244 |
| ***Sult1b1*** | -0,06713 | 0,322959 | 0,273638 | -0,20484 | -0,14874 |
| ***Cyp1b1*** | -0,2066 | 0,193461 | -0,00119 | -0,04376 | 0,197928 |
| ***Large2*** | 0,198625 | 0,126268 | -0,19906 | -0,11465 | 0,127858 |
| ***B4gat1*** | 0,028608 | 0,044543 | 0,062539 | -0,05311 | -0,00796 |
| ***Ugt2a1*** | -0,01249 | 0,005613 | 0,013367 | 0,025455 | 0,025249 |
| ***Ext2*** | 0,055656 | 0,027359 | -0,09587 | -0,06187 | 0,062621 |
| ***Cyp4x1*** | -0,25655 | 0,308355 | -0,02177 | -0,2414 | 0,162979 |
| ***Sult1c3*** | -0,34907 | 0,08808 | 0,041571 | -0,07432 | 0,237151 |
| ***Ugt2a3*** | 0,006423 | 1,216072 | -0,00087 | -0,60617 | -0,79121 |
| ***Csgalnact1*** | 0,013946 | -0,10522 | 0,066224 | -0,03088 | -0,17641 |
| ***Large1*** | 0,509803 | -1,06261 | -0,15702 | 0,061787 | 0,240405 |
| ***Cyp51a1*** | -0,32394 | -0,46856 | 0,011095 | 0,040245 | 0,26191 |
| ***Cyp2c18*** | -0,2453 | 0,259722 | 0,049139 | -0,39986 | -0,29794 |
| ***Ugt2b10*** | -0,06221 | -0,04937 | 0,124047 | -0,33365 | -0,35 |
| ***Cyp2j2*** | 0,072261 | 0,23827 | -0,40419 | -0,37816 | -0,2528 |
| ***Cyp3a7*** | -0,36401 | -0,43583 | -0,05304 | 0,112209 | 0,001546 |
| ***Cyp4f8*** | -0,05355 | 1,32477 | -0,16482 | -1,06064 | -0,8168 |
| ***Cyp2c9*** | -0,67462 | 0,257784 | 0,167694 | -0,2441 | -0,31643 |
| ***Cyp4b1*** | 0,042087 | 0,213902 | -0,018 | -0,61088 | -0,66467 |
| ***Ugt3a1*** | -0,17178 | 1,538057 | 0,565115 | -1,43647 | -1,80589 |
| ***Sult1d1*** | -0,75893 | -0,67835 | -0,02509 | -0,09733 | -0,65697 |
| ***Sult1a1*** | -0,73322 | -1,18578 | -0,9819 | -0,42152 | -0,10652 |
| ***Cyp2a6*** | -1,61421 | -1,43386 | -1,73229 | -1,86785 | -2,32511 |
| ***Cyp2b6*** | -4,87545 | -1,58102 | -1,3552 | -1,03239 | -1,02875 |
| ***Cyp3a5*** | -4,0533 | -3,72033 | -2,12901 | 0,626905 | -0,99918 |
| ***Cyp2c8*** | -3,0275 | -2,40431 | -1,64767 | -1,56727 | -2,10398 |
| ***Cyp2c40*** | -3,13872 | -2,61072 | -3,0842 | -2,64018 | -2,58465 |

**Table S6:** Activation Z-score of genes involved in the superpathway of melatonin degradation male vs. female. Activation Z-score was calculated with IPA software from Qiagen.

| **Pathway Summary** | -2,065 | -0,408 | 0,229 | -1,964 | -3,638 |
| --- | --- | --- | --- | --- | --- |
| **genes in the superpathway of melatonin degradation network** | **0 h** | **24 h** | **48 h** | **72 h** | **96 h** |
| ***Ugt2b17*** | 0,557028 | 2,213161 | 1,292475 | 0,591189 | -0,39135 |
| ***Cyp2e1*** | 0,155034 | 0,940182 | 2,273941 | 0,984777 | -0,50221 |
| ***Cyp2u1*** | 1,499901 | 0,704252 | 0,276107 | 0,353724 | 0,40437 |
| ***Cyp1a2*** | 0,066898 | 1,509168 | 1,215758 | 0,619908 | -1,80321 |
| ***Ugt2b28*** | 0,774388 | 0,659801 | 0,273055 | -0,19352 | -0,16884 |
| ***Cyp2s1*** | -0,07623 | -0,17694 | 0,083406 | 0,723848 | 0,266478 |
| ***Smox*** | -0,38514 | 0,265107 | 0,58581 | 0,252859 | 0,094358 |
| ***Cyp1a1*** | -0,0377 | 0,221035 | 0,38464 | 0,078008 | 0,130361 |
| ***Por*** | -0,22385 | 0,116112 | 0,419562 | 0,346398 | -0,05581 |
| ***Mpo*** | 0,058613 | 0,1117 | 0,02257 | 0,230348 | 0,084871 |
| ***Ugt2b7*** | 0,55257 | -0,32078 | 0,463908 | -0,14661 | -0,11281 |
| ***Cyp2f1*** | 0,118274 | 1,192971 | 0,421422 | -0,61217 | -0,78225 |
| ***Cyp19a1*** | -0,01045 | 0,047247 | 0,095659 | -0,00655 | 0,153157 |
| ***B3gat3*** | 0,044436 | -0,0449 | -0,00396 | 0,117245 | 0,087244 |
| ***Sult1b1*** | -0,06713 | 0,322959 | 0,273638 | -0,20484 | -0,14874 |
| ***Cyp1b1*** | -0,2066 | 0,193461 | -0,00119 | -0,04376 | 0,197928 |
| ***Large2*** | 0,198625 | 0,126268 | -0,19906 | -0,11465 | 0,127858 |
| ***B4gat1*** | 0,028608 | 0,044543 | 0,062539 | -0,05311 | -0,00796 |
| ***Ugt2a1*** | -0,01249 | 0,005613 | 0,013367 | 0,025455 | 0,025249 |
| ***Ext2*** | 0,055656 | 0,027359 | -0,09587 | -0,06187 | 0,062621 |
| ***Cyp4x1*** | -0,25655 | 0,308355 | -0,02177 | -0,2414 | 0,162979 |
| ***Sult1c3*** | -0,34907 | 0,08808 | 0,041571 | -0,07432 | 0,237151 |
| ***Ugt2a3*** | 0,006423 | 1,216072 | -0,00087 | -0,60617 | -0,79121 |
| ***Csgalnact1*** | 0,013946 | -0,10522 | 0,066224 | -0,03088 | -0,17641 |
| ***Large1*** | 0,509803 | -1,06261 | -0,15702 | 0,061787 | 0,240405 |
| ***Cyp51a1*** | -0,32394 | -0,46856 | 0,011095 | 0,040245 | 0,26191 |
| ***Cyp2c18*** | -0,2453 | 0,259722 | 0,049139 | -0,39986 | -0,29794 |
| ***Ugt2b10*** | -0,06221 | -0,04937 | 0,124047 | -0,33365 | -0,35 |
| ***Cyp2j2*** | 0,072261 | 0,23827 | -0,40419 | -0,37816 | -0,2528 |
| ***Cyp3a7*** | -0,36401 | -0,43583 | -0,05304 | 0,112209 | 0,001546 |
| ***Cyp4f8*** | -0,05355 | 1,32477 | -0,16482 | -1,06064 | -0,8168 |
| ***Cyp2c9*** | -0,67462 | 0,257784 | 0,167694 | -0,2441 | -0,31643 |
| ***Cyp4b1*** | 0,042087 | 0,213902 | -0,018 | -0,61088 | -0,66467 |
| ***Ugt3a1*** | -0,17178 | 1,538057 | 0,565115 | -1,43647 | -1,80589 |
| ***Sult1d1*** | -0,75893 | -0,67835 | -0,02509 | -0,09733 | -0,65697 |
| ***Maob*** | -0,68574 | -0,33792 | -0,72165 | -0,47743 | -0,326 |
| ***Sult1a1*** | -0,73322 | -1,18578 | -0,9819 | -0,42152 | -0,10652 |
| ***Maoa*** | -0,70329 | -0,4951 | -1,32169 | -1,29354 | -0,54919 |
| ***Cyp2a6*** | -1,61421 | -1,43386 | -1,73229 | -1,86785 | -2,32511 |
| ***Cyp2b6*** | -4,87545 | -1,58102 | -1,3552 | -1,03239 | -1,02875 |
| ***Cyp3a5*** | -4,0533 | -3,72033 | -2,12901 | 0,626905 | -0,99918 |
| ***Cyp2c8*** | -3,0275 | -2,40431 | -1,64767 | -1,56727 | -2,10398 |
| ***Cyp2c40*** | -3,13872 | -2,61072 | -3,0842 | -2,64018 | -2,58465 |

**Table S7:** Activation Z-score of genes involved in IGF-1 signaling male vs. female. Activation Z-score was calculated with IPA software from Qiagen.

| **Pathway Summary** | -0,853 | -2,117 | 0,535 | 0,894 | -1,414 |
| --- | --- | --- | --- | --- | --- |
| **genes in the IGF-1 signaling network** | **0 h** | **24 h** | **48 h** | **72 h** | **96 h** |
| ***Fos*** | 0,477946 | 1,616629 | 1,925766 | 1,628887 | 1,742349 |
| ***Jun*** | 0,719868 | 0,883187 | 0,549306 | 0,296255 | 0,375915 |
| ***Srf*** | 0,166654 | 0,267418 | 0,768951 | 0,658099 | 0,255454 |
| ***Igfbp3*** | 0,154127 | 0,129484 | -0,25423 | 0,6088 | 1,466355 |
| ***Socs7*** | 0,671739 | 0,832915 | 0,231459 | 0,372671 | -0,19324 |
| ***Igf1*** | -0,0711 | 0,534636 | 0,478904 | 0,342446 | 0,463394 |
| ***Socs3*** | 0,648692 | 0,44729 | 0,310238 | 0,230759 | 0,070863 |
| ***Ccn1*** | 0,813415 | -0,28874 | 0,297317 | 0,371764 | 0,414685 |
| ***Igfbp2*** | 0,031061 | 0,922081 | 0,547374 | -0,04514 | -0,18594 |
| ***Ccn2*** | -0,15655 | -0,05686 | 0,333359 | 0,426881 | 0,618521 |
| ***Csnk2a2*** | 0,144079 | 0,411782 | 0,260595 | 0,257485 | 0,005596 |
| ***Mras*** | 0,174646 | 1,003178 | -0,15429 | -0,08634 | 0,102783 |
| ***Ywhag*** | 0,339208 | 0,21306 | 0,216996 | 0,046067 | 0,21918 |
| ***Map2k1*** | 0,13442 | 0,207022 | 0,290068 | 0,177577 | 0,173668 |
| ***Map2k2*** | 0,575392 | -0,15963 | 0,11357 | 0,344424 | 0,071213 |
| ***Sfn*** | -0,18258 | -0,13214 | 0,5573 | 0,359966 | 0,265365 |
| ***Sos1*** | 0,264454 | 0,128647 | 0,077278 | 0,247009 | 0,056206 |
| ***Prkar1b*** | -0,0144 | 0,23196 | 0,228917 | 0,084269 | 0,096835 |
| ***Jak1*** | 0,143002 | 0,289012 | 0,123497 | -0,0766 | 0,130972 |
| ***Rala*** | 0,0355 | 0,107634 | 0,179678 | 0,155122 | 0,08952 |
| ***Rasd2*** | -0,06273 | 0,27061 | 0,160291 | -0,01052 | 0,20936 |
| ***Ralb*** | 0,014308 | 0,182199 | -0,06845 | 0,121461 | 0,291222 |
| ***Socs1*** | -0,02489 | 0,159075 | 0,008826 | -0,02151 | 0,377607 |
| ***Pik3c2a*** | 0,005251 | -0,19112 | 0,220942 | 0,330514 | 0,110934 |
| ***Prkar2b*** | -0,27859 | 0,108796 | 0,194686 | 0,282446 | 0,160946 |
| ***Pik3r5*** | -0,01583 | 0,191325 | 0,133672 | -0,09727 | 0,247275 |
| ***Csnk2b*** | 0,07672 | 0,050343 | 0,09571 | 0,184862 | 0,044891 |
| ***Igf1r*** | -0,0981 | -0,0826 | 0,279362 | 0,110573 | 0,242523 |
| ***Pik3c2b*** | 0,118141 | 0,169816 | 0,018153 | 0,04807 | 0,042186 |
| ***Mapk8*** | -0,004 | 0,261262 | 0,311823 | 0,025523 | -0,20447 |
| ***Pik3r6*** | -0,14476 | 0,123504 | 0,067777 | 0,110262 | 0,229527 |
| ***Foxo3*** | 0,150165 | 0,280371 | 0,170242 | -0,06662 | -0,14944 |
| ***Elk1*** | 0,046732 | 0,036118 | 0,121456 | 0,194401 | -0,02615 |
| ***Raf1*** | 0,095374 | 0,446542 | -0,06254 | 0,026048 | -0,14962 |
| ***Igfbp7*** | 0,097491 | 0,114347 | -0,10688 | 0,093652 | 0,142331 |
| ***Igfbp1*** | -0,23076 | 0,167957 | 0,213571 | 0,25428 | -0,07858 |
| ***Rps6kb2*** | -0,03822 | 0,022134 | -0,02301 | 0,169163 | 0,185489 |
| ***Stat3*** | 0,030441 | 0,050623 | 0,152673 | 0,083143 | -0,0019 |
| ***Hras*** | 0,40146 | -0,23578 | -0,11532 | 0,234695 | 0,023973 |
| ***Rasd1*** | 0,052517 | 0,084486 | 0,002894 | 0,137336 | -0,00633 |
| ***Ccn3*** | -0,0378 | 0,039172 | 0,057329 | 0,067361 | 0,10377 |
| ***Csnk2a1*** | 0,043272 | 0,129717 | 0,06803 | -0,11024 | 0,021634 |
| ***Casp9*** | 0,036725 | -0,07209 | 0,046627 | 0,030927 | 0,095945 |
| ***Pik3r2*** | 0,25572 | -0,09114 | 0,075034 | -0,03299 | -0,0685 |
| ***Bad*** | 0,064966 | 0,013672 | 0,015555 | 0,017132 | 0,001594 |
| ***Akt3*** | -0,24716 | -0,00756 | 0,243473 | 0,466583 | -0,35147 |
| ***Igfbp6*** | 0,169145 | 0,175452 | 0,039918 | -0,2352 | -0,05515 |
| ***Ptpn11*** | 0,277848 | -0,11732 | -0,15029 | 0,091185 | -0,0196 |
| ***Nras*** | -0,01512 | -0,20668 | 0,095093 | 0,160952 | 0,042765 |
| ***Igfbp4*** | 0,023353 | 0,525892 | -0,55693 | 0,084373 | -0,00613 |
| ***Foxo1*** | -0,14653 | -0,02777 | 0,134011 | 0,162362 | -0,1237 |
| ***Rap1b*** | -0,10425 | 0,078944 | -0,00589 | 0,027729 | -0,0215 |
| ***Socs4*** | 0,263553 | -0,08164 | -0,11732 | 0,048054 | -0,14043 |
| ***Socs6*** | 0,135443 | -0,05049 | 0,113252 | 0,007963 | -0,24327 |
| ***Pik3r3*** | 0,293126 | -0,01107 | -0,15181 | -0,15931 | -0,02902 |
| ***Rps6kb1*** | 0,108294 | -0,20059 | 0,049206 | 0,088938 | -0,10768 |
| ***Sos2*** | -0,26722 | 0,121053 | 0,169366 | 0,096972 | -0,19058 |
| ***Prkci*** | 0,024551 | -0,43889 | 0,186525 | 0,082533 | 0,04227 |
| ***Prkar2a*** | 0,387154 | -0,67441 | 0,07029 | 0,328312 | -0,22801 |
| ***Mapk1*** | 0,044576 | -0,16036 | 0,093211 | 0,032593 | -0,13623 |
| ***Pik3ca*** | 0,052469 | 0,079205 | -0,0719 | -0,11625 | -0,08746 |
| ***Rap2a*** | 0,51035 | -0,04705 | -0,10372 | -0,20105 | -0,30269 |
| ***Grb2*** | -0,06551 | 0,079172 | -0,07072 | -0,06606 | -0,04439 |
| ***Prkar1a*** | -0,19423 | -0,07836 | 0,018844 | 0,029118 | 0,03697 |
| ***Pik3cd*** | -0,26532 | 0,047348 | 0,051176 | 0,075089 | -0,11023 |
| ***Ywhaz*** | 0,050604 | -0,27762 | -0,04673 | -0,00397 | 0,054837 |
| ***Rasa1*** | 0,043313 | -0,05235 | -0,04907 | -0,01759 | -0,16055 |
| ***Ywhae*** | 0,043221 | -0,29075 | -0,0455 | 0,067446 | -0,0156 |
| ***Prkag1*** | 0,015082 | -0,1281 | -0,02855 | -0,07731 | -0,04722 |
| ***Pdpk1*** | 0,193546 | -0,2998 | -0,05357 | -0,05531 | -0,06789 |
| ***Socs5*** | -0,18997 | -0,26372 | -0,11039 | 0,044506 | 0,22789 |
| ***Rras*** | -0,04157 | -0,40855 | -0,11514 | 0,07881 | 0,186698 |
| ***Pik3r1*** | 0,090286 | -0,57779 | -0,2358 | 0,144529 | 0,263983 |
| ***Prkacb*** | -0,17137 | -0,11709 | 0,152978 | -0,07941 | -0,11836 |
| ***Akt2*** | -0,09815 | -0,15659 | -0,00876 | -0,09686 | -0,01344 |
| ***Irs2*** | -0,09888 | -0,29547 | 0,072083 | 0,102651 | -0,16769 |
| ***Pik3cg*** | -0,02541 | -0,17348 | -0,01282 | 0,058653 | -0,23801 |
| ***Ptk2*** | 0,271355 | -0,35804 | -0,05002 | -0,10527 | -0,1699 |
| ***Ywhaq*** | 0,008256 | -0,30748 | -0,12755 | -0,14908 | 0,138903 |
| ***Pik3r4*** | -0,00762 | 0,057253 | -0,08123 | -0,32561 | -0,09624 |
| ***Prkaca*** | 0,087291 | -0,5 | -0,14718 | 0,014032 | 0,092124 |
| ***Pik3c3*** | -0,23607 | 0,073196 | -0,14118 | -0,14439 | -0,05641 |
| ***Ywhah*** | 0,047272 | -0,40727 | -0,13391 | -0,07661 | 0,042428 |
| ***Igfbp5*** | -0,74584 | 0,223234 | -0,01959 | -0,0685 | 0,009942 |
| ***Akt1*** | -0,12742 | -0,47145 | -0,12531 | -0,0356 | 0,152021 |
| ***Rap2b*** | -0,53235 | 0,177452 | 0,098047 | -0,30301 | -0,10827 |
| ***Pxn*** | -0,20978 | -0,01424 | -0,24803 | -0,19874 | -0,03971 |
| ***Ywhab*** | -0,03271 | -0,63669 | -0,18525 | 0,093508 | 0,033802 |
| ***Jak2*** | -0,02781 | -0,30249 | -0,25278 | 0,041496 | -0,19887 |
| ***Mapk3*** | -0,12178 | -0,2083 | -0,11816 | -0,24199 | -0,08334 |
| ***Prkcz*** | 0,069884 | -0,36703 | -0,18978 | -0,14472 | -0,18557 |
| ***Shc1*** | -0,03727 | -0,27279 | -0,23371 | -0,16462 | -0,14019 |
| ***Rap1a*** | -0,09409 | -0,14484 | -0,13954 | -0,2421 | -0,26123 |
| ***Eras*** | -0,10103 | -0,09304 | -0,48438 | -0,19299 | -0,02703 |
| ***Kras*** | -0,26815 | -0,15401 | -0,19245 | -0,26561 | -0,23842 |
| ***Pik3c2g*** | -0,48782 | -0,08919 | -0,10013 | -0,11043 | -0,35399 |
| ***Pik3cb*** | -0,21367 | -0,8372 | -0,21036 | -0,02392 | -0,02048 |
| ***Rras2*** | -0,07237 | -0,80744 | -0,56238 | -0,24113 | -0,04987 |
| ***Socs2*** | 0,887957 | -0,6283 | -1,35676 | -0,69105 | 0,046374 |
| ***Irs1*** | -0,02384 | -0,72107 | -0,35943 | -0,17462 | -0,53636 |
| ***Prkag2*** | -0,50905 | -0,84728 | -0,13855 | -0,19606 | -0,26978 |
| ***Grb10*** | -0,41701 | -0,3467 | -0,27657 | -0,92674 | -1,09772 |

**Table S8:** Activation Z-score of genes involved in growth hormone signaling male vs. female. Activation Z-score was calculated with IPA software from Qiagen.

| **Pathway summary** | -1,387 | -2,041 | 1 | 0,258 | -1,265 |
| --- | --- | --- | --- | --- | --- |
| **genes in the growth hormone signaling network** | **0 h** | **24 h** | **48 h** | **72 h** | **96 h** |
| ***Fos*** | 0,477946 | 1,616629 | 1,925766 | 1,628887 | 1,742349 |
| ***Srf*** | 0,166654 | 0,267418 | 0,768951 | 0,658099 | 0,255454 |
| ***Igfbp3*** | 0,154127 | 0,129484 | -0,25423 | 0,6088 | 1,466355 |
| ***Socs7*** | 0,671739 | 0,832915 | 0,231459 | 0,372671 | -0,19324 |
| ***Igf1*** | -0,0711 | 0,534636 | 0,478904 | 0,342446 | 0,463394 |
| ***Socs3*** | 0,648692 | 0,44729 | 0,310238 | 0,230759 | 0,070863 |
| ***Cebpa*** | 0,565119 | 1,128915 | 0,105326 | -0,23774 | -0,37059 |
| ***Rps6ka1*** | 0,356569 | -0,42647 | 0,267063 | 0,635706 | 0,32401 |
| ***Igfals*** | -0,13726 | 0,154454 | 0,171826 | 0,3672 | 0,465491 |
| ***Prkcg*** | -0,04543 | 0,032092 | 0,447063 | 0,40637 | 0,164773 |
| ***Prkcd*** | 0,074277 | 0,348892 | 0,246503 | 0,028518 | 0,008762 |
| ***Rps6ka2*** | 0,062066 | 0,063265 | 0,385395 | 0,174028 | -0,04269 |
| ***Cshl1*** | 0,149458 | 0,180954 | 0,157992 | -0,03499 | 0,175119 |
| ***Rps6ka4*** | -0,11524 | 0,052492 | 0,24968 | 0,263589 | 0,171066 |
| ***Socs1*** | -0,02489 | 0,159075 | 0,008826 | -0,02151 | 0,377607 |
| ***Pik3c2a*** | 0,005251 | -0,19112 | 0,220942 | 0,330514 | 0,110934 |
| ***Plcg1*** | 0,076618 | 0,17445 | 0,163278 | -0,05111 | 0,113243 |
| ***Pik3r5*** | -0,01583 | 0,191325 | 0,133672 | -0,09727 | 0,247275 |
| ***Igf1r*** | -0,0981 | -0,0826 | 0,279362 | 0,110573 | 0,242523 |
| ***Pik3c2b*** | 0,118141 | 0,169816 | 0,018153 | 0,04807 | 0,042186 |
| ***Slc2a4*** | 0,021205 | 0,073454 | 0,148644 | 0,144957 | 0,005989 |
| ***Pik3r6*** | -0,14476 | 0,123504 | 0,067777 | 0,110262 | 0,229527 |
| ***Elk1*** | 0,046732 | 0,036118 | 0,121456 | 0,194401 | -0,02615 |
| ***Rps6kb2*** | -0,03822 | 0,022134 | -0,02301 | 0,169163 | 0,185489 |
| ***Stat3*** | 0,030441 | 0,050623 | 0,152673 | 0,083143 | -0,0019 |
| ***Prkch*** | 0,199389 | -0,01281 | 0,351147 | -0,02797 | -0,20844 |
| ***A2m*** | -0,03041 | -0,01181 | 0,077727 | 0,101642 | 0,150715 |
| ***Stat5b*** | -0,23896 | 0,314471 | 0,099072 | 0,045493 | 0,011286 |
| ***Plcg2*** | 0,245478 | 0,197959 | -0,16078 | -0,02148 | -0,03416 |
| ***Rps6ka6*** | -0,15181 | 0,000924 | 0,171155 | 0,193801 | -0,0119 |
| ***Ghr*** | 0,020345 | 0,862462 | -0,07016 | -0,35706 | -0,26179 |
| ***Prkcq*** | 0,10399 | -0,25314 | 0,039609 | 0,162102 | 0,11465 |
| ***Igf2*** | -0,21875 | 0,049443 | 0,216651 | 0,054757 | 0,062983 |
| ***Stat5a*** | -0,24017 | 0,286443 | 0,032579 | -0,0361 | 0,110802 |
| ***Prkd1*** | -0,00044 | 0,078895 | -0,14158 | 0,042999 | 0,164733 |
| ***Pik3r2*** | 0,25572 | -0,09114 | 0,075034 | -0,03299 | -0,0685 |
| ***Prkcb*** | -0,12369 | 0,164085 | -0,09661 | 0,022314 | 0,069768 |
| ***Socs4*** | 0,263553 | -0,08164 | -0,11732 | 0,048054 | -0,14043 |
| ***Socs6*** | 0,135443 | -0,05049 | 0,113252 | 0,007963 | -0,24327 |
| ***Pik3r3*** | 0,293126 | -0,01107 | -0,15181 | -0,15931 | -0,02902 |
| ***Rps6kb1*** | 0,108294 | -0,20059 | 0,049206 | 0,088938 | -0,10768 |
| ***Rps6kc1*** | 0,201055 | -0,36091 | -0,10481 | 0,205575 | -0,03476 |
| ***Prl*** | -0,0813 | 0,107393 | -0,02015 | -0,16834 | 0,066114 |
| ***Prkci*** | 0,024551 | -0,43889 | 0,186525 | 0,082533 | 0,04227 |
| ***Mapk1*** | 0,044576 | -0,16036 | 0,093211 | 0,032593 | -0,13623 |
| ***Pik3ca*** | 0,052469 | 0,079205 | -0,0719 | -0,11625 | -0,08746 |
| ***Pik3cd*** | -0,26532 | 0,047348 | 0,051176 | 0,075089 | -0,11023 |
| ***Pdpk1*** | 0,193546 | -0,2998 | -0,05357 | -0,05531 | -0,06789 |
| ***Socs5*** | -0,18997 | -0,26372 | -0,11039 | 0,044506 | 0,22789 |
| ***Pik3r1*** | 0,090286 | -0,57779 | -0,2358 | 0,144529 | 0,263983 |
| ***Prkce*** | 0,064813 | -0,4592 | -0,03295 | 0,190098 | -0,12429 |
| ***Rps6ka5*** | 0,176684 | -0,0003 | -0,19175 | -0,28546 | -0,06267 |
| ***Pik3cg*** | -0,02541 | -0,17348 | -0,01282 | 0,058653 | -0,23801 |
| ***Pik3r4*** | -0,00762 | 0,057253 | -0,08123 | -0,32561 | -0,09624 |
| ***Ptpn6*** | -0,15087 | -0,10891 | -0,30655 | -0,02284 | 0,128052 |
| ***Pik3c3*** | -0,23607 | 0,073196 | -0,14118 | -0,14439 | -0,05641 |
| ***Onecut1*** | 0,673633 | -0,44932 | -0,54448 | 0,010427 | -0,21462 |
| ***Jak2*** | -0,02781 | -0,30249 | -0,25278 | 0,041496 | -0,19887 |
| ***Mapk3*** | -0,12178 | -0,2083 | -0,11816 | -0,24199 | -0,08334 |
| ***Prkcz*** | 0,069884 | -0,36703 | -0,18978 | -0,14472 | -0,18557 |
| ***Rps6ka3*** | -0,05131 | -0,42525 | -0,11463 | -0,30208 | -0,12955 |
| ***Pik3c2g*** | -0,48782 | -0,08919 | -0,10013 | -0,11043 | -0,35399 |
| ***Pik3cb*** | -0,21367 | -0,8372 | -0,21036 | -0,02392 | -0,02048 |
| ***Socs2*** | 0,887957 | -0,6283 | -1,35676 | -0,69105 | 0,046374 |
| ***Irs1*** | -0,02384 | -0,72107 | -0,35943 | -0,17462 | -0,53636 |
| ***Stat1*** | -0,22362 | -0,63717 | -0,45049 | -0,29489 | -0,31201 |
| ***Prkd3*** | -0,08954 | -0,27329 | -0,74934 | -0,84385 | -0,69256 |
| ***Prkca*** | -0,78907 | -1,45442 | -0,55646 | -0,30357 | -0,30056 |

**Table S9:** Activation Z-score of genes involved in aryl hydrocarbon receptor signaling male vs. female. Activation Z-score was calculated with IPA software from Qiagen.

| **Pathway Summary** | 1,46 | -0,16 | -2,2 | -1 | 0,775 |
| --- | --- | --- | --- | --- | --- |
| **genes in the aryl hydrocarbon receptor signaling network** | **0 h** | **24 h** | **48 h** | **72 h** | **96 h** |
| ***Fos*** | 0,477946 | 1,616629 | 1,925766 | 1,628887 | 1,742349 |
| ***Gstp1*** | 1,882254 | 0,337857 | 0,177296 | 0,244776 | 0,194589 |
| ***Jun*** | 0,719868 | 0,883187 | 0,549306 | 0,296255 | 0,375915 |
| ***Hspb1*** | 0,840589 | -0,25501 | 0,693456 | 0,618116 | 0,540379 |
| ***Tgm2*** | -0,0872 | 0,169539 | 0,582522 | 0,639144 | 0,547246 |
| ***Cyp1a2*** | 0,066898 | 1,509168 | 1,215758 | 0,619908 | -1,80321 |
| ***Aldh4a1*** | -0,18994 | 1,145047 | 0,371212 | 0,082255 | -0,3707 |
| ***Hspb3*** | 0,070333 | 0,042504 | 0,345678 | 0,089364 | 0,353021 |
| ***Esr2*** | -0,00057 | 0,018629 | 0,301336 | 0,113546 | 0,433366 |
| ***Tyr*** | 0,328416 | 0,102677 | 0,150013 | 0,054926 | 0,220609 |
| ***Myc*** | 0,149033 | 0,077665 | 0,227367 | 0,168713 | 0,230961 |
| ***Cyp1a1*** | -0,0377 | 0,221035 | 0,38464 | 0,078008 | 0,130361 |
| ***Aldh1l1*** | -0,0806 | 1,174822 | 0,301611 | -0,27572 | -0,37022 |
| ***Ccne2*** | 0,003399 | 0,667724 | 0,064122 | 0,251056 | -0,25375 |
| ***Ncor2*** | -0,03862 | 0,293661 | 0,141303 | 0,190168 | 0,142945 |
| ***Il1b*** | 0,263769 | 0,310882 | -0,03707 | 0,045763 | 0,139904 |
| ***Nfkb1*** | 0,158929 | 0,198966 | -0,05669 | 0,144947 | 0,239545 |
| ***Mdm2*** | 0,19264 | 0,459077 | -0,14754 | -0,09064 | 0,253251 |
| ***Ccna1*** | 0,212854 | 0,166242 | -0,14205 | 0,142584 | 0,286707 |
| ***Sp1*** | 0,094922 | 0,310516 | 0,068808 | 0,047906 | 0,139302 |
| ***Rela*** | 0,172055 | 0,224581 | 0,095794 | 0,07538 | 0,090153 |
| ***Hsp90aa1*** | 0,502608 | -0,32229 | 0,178781 | 0,094177 | 0,142744 |
| ***Ncoa3*** | 0,336911 | 0,236946 | -0,20161 | 0,16127 | -0,00466 |
| ***Tgfb2*** | -0,09135 | 0,29691 | -0,22754 | 0,184326 | 0,361922 |
| ***Smarca4*** | 0,10746 | 0,020047 | 0,021567 | 0,237996 | 0,101907 |
| ***Rxrb*** | 0,457257 | 0,219044 | 0,120287 | -0,14449 | -0,17246 |
| ***Aldh3a1*** | -0,01797 | 0,141252 | 0,02739 | 0,166023 | 0,14445 |
| ***Aldh1l2*** | -0,09203 | -0,04787 | 0,308585 | 0,377108 | -0,09043 |
| ***Aldh7a1*** | 0,220354 | 0,819824 | -0,32258 | -0,21583 | -0,08076 |
| ***E2f1*** | 0,383477 | 0,036203 | -0,14638 | -0,01541 | 0,148558 |
| ***Ccne1*** | 0,063307 | -0,09174 | -0,12477 | 0,147856 | 0,410568 |
| ***Tfdp1*** | 0,479729 | -0,24715 | -0,05037 | 0,055696 | 0,16705 |
| ***Mapk8*** | -0,004 | 0,261262 | 0,311823 | 0,025523 | -0,20447 |
| ***Aldh16a1*** | 0,101859 | 0,218341 | 0,078239 | -0,04107 | 0,029463 |
| ***Ahrr*** | 0,068564 | 0,047845 | 0,055737 | 0,132749 | 0,055582 |
| ***Ep300*** | 0,223775 | -0,03702 | 0,045473 | 0,279534 | -0,16359 |
| ***Cdkn1a*** | -0,38236 | -0,08342 | 0,323505 | 0,230924 | 0,241859 |
| ***Src*** | -0,27544 | 0,024078 | 0,202661 | 0,159584 | 0,199357 |
| ***Ncoa2*** | 0,086645 | 0,128211 | -0,00857 | 0,024341 | 0,049417 |
| ***Atr*** | 0,206521 | -0,08781 | 0,175079 | -0,09869 | 0,035034 |
| ***Rbl2*** | 0,286966 | 0,070377 | -0,02969 | 0,104901 | -0,21339 |
| ***Trip11*** | -0,01402 | -0,2151 | 0,127259 | 0,166191 | 0,154065 |
| ***Aldh1a2*** | -0,04424 | 0,074551 | -0,0644 | 0,130846 | 0,115865 |
| ***Rarg*** | -0,04255 | 0,066603 | -0,01461 | 0,039029 | 0,145206 |
| ***Arnt*** | -0,02327 | 0,187907 | -0,00335 | -0,04279 | 0,055936 |
| ***Tnf*** | -0,18177 | -0,4173 | 0,296697 | 0,27893 | 0,183334 |
| ***Nedd8*** | 0,193552 | -0,17364 | 0,03783 | 0,081964 | 0,012412 |
| ***Cyp1b1*** | -0,2066 | 0,193461 | -0,00119 | -0,04376 | 0,197928 |
| ***Hspb2*** | -0,1899 | -0,04605 | 0,095898 | 0,031126 | 0,238688 |
| ***Rxra*** | 0,193827 | 0,169862 | 0,015074 | 0,019906 | -0,27327 |
| ***Aip*** | 0,258088 | 0,093464 | -0,00095 | -0,20454 | -0,03918 |
| ***Tgfb1*** | -0,07187 | 0,048726 | -0,04699 | 0,189121 | -0,01543 |
| ***Nfic*** | 0,317765 | -0,24542 | -0,08813 | 0,13687 | -0,02143 |
| ***Ccnd3*** | 0,053045 | -0,24094 | 0,075726 | 0,141443 | 0,052609 |
| ***Ptges3*** | -0,09485 | -0,13093 | 0,394845 | 0,005238 | -0,10124 |
| ***Cdkn2a*** | -0,08573 | 0,201236 | -0,30213 | 0,013845 | 0,22452 |
| ***Hsp90ab1*** | 0,153701 | -0,42093 | 0,171052 | 0,082244 | 0,047582 |
| ***Tp73*** | -0,20001 | 0,097373 | 0,08042 | -0,03376 | 0,086225 |
| ***Ahr*** | -0,14658 | 0,313906 | -0,04025 | -0,03685 | -0,07687 |
| ***Mgst2*** | 0,091526 | -0,15884 | 0,018484 | -0,07328 | 0,127777 |
| ***Il1a*** | -0,31893 | -0,09356 | 0,255765 | 0,197994 | -0,07798 |
| ***Nrip1*** | 0,062801 | -0,10827 | 0,210467 | -0,17986 | -0,03364 |
| ***Hspb7*** | -0,28074 | -0,01222 | 0,044173 | 0,147622 | 0,050885 |
| ***Ncoa7*** | 0,185997 | -0,00132 | 0,066625 | -0,29896 | -0,02679 |
| ***Mgst1*** | 0,106844 | 0,117306 | -0,03641 | -0,20413 | -0,0961 |
| ***Mapk1*** | 0,044576 | -0,16036 | 0,093211 | 0,032593 | -0,13623 |
| ***Gstm3*** | -0,14577 | -0,12861 | 0,088728 | 0,337619 | -0,29394 |
| ***Bax*** | 0,015485 | -0,26442 | 0,05446 | -0,0615 | 0,110614 |
| ***Cdkn1b*** | -0,07612 | 0,169467 | 0,093087 | -0,16706 | -0,17122 |
| ***Il6*** | -0,00664 | -0,10692 | 0,07348 | -0,05884 | -0,06107 |
| ***Nfix*** | 0,302124 | -0,09523 | -0,18531 | -0,07514 | -0,12294 |
| ***Gsto2*** | -0,2979 | -0,13784 | -0,0402 | 0,048601 | 0,235744 |
| ***Nr2f1*** | -0,13963 | -0,01326 | 0,180148 | -0,13477 | -0,11356 |
| ***Rara*** | 0,118619 | -0,32306 | -0,15279 | 0,129174 | -0,01458 |
| ***Nfe2l2*** | 0,237238 | 0,092282 | -0,35333 | -0,29459 | 0,071303 |
| ***Aldh18a1*** | 0,204397 | -0,38172 | -0,32766 | -0,0109 | 0,227284 |
| ***Aldh2*** | -0,05226 | 0,209599 | -0,10349 | -0,25722 | -0,14803 |
| ***Gstz1*** | -0,02332 | 0,812286 | -0,42883 | -0,45182 | -0,28936 |
| ***Faslg*** | -0,00086 | -0,10271 | -0,24749 | -0,2074 | 0,158512 |
| ***Aldh6a1*** | -0,12568 | 0,70767 | -0,35314 | -0,33851 | -0,32771 |
| ***Gstm6*** | -0,17145 | 0,278406 | -0,21578 | -0,06966 | -0,26243 |
| ***Nr0b2*** | -0,35622 | 0,233567 | 0,047325 | -0,39205 | 0,021575 |
| ***Gstm5*** | -0,13549 | -0,09147 | -0,00525 | -0,06958 | -0,14526 |
| ***Gsta3*** | 0,082941 | 0,207171 | -0,25599 | -0,34971 | -0,13187 |
| ***Aldh3b1*** | -0,26231 | 0,03008 | -0,35622 | 0,084234 | 0,031547 |
| ***Nfkb2*** | -0,12845 | -0,86625 | -0,02873 | 0,190012 | 0,325921 |
| ***Ctsd*** | -0,22888 | -0,15331 | -0,0349 | -0,05525 | -0,04359 |
| ***Gsta5*** | -0,26097 | -0,83708 | 0,265944 | 0,378938 | -0,0793 |
| ***Gstk1*** | -0,18235 | 0,980101 | -0,47865 | -0,43221 | -0,41973 |
| ***Gstm4*** | -0,10794 | -0,03032 | 0,081005 | -0,06848 | -0,40839 |
| ***Aldh5a1*** | -0,01626 | 0,086619 | -0,35096 | -0,1413 | -0,15137 |
| ***Chek2*** | 0,368428 | -0,01923 | -0,35414 | -0,25727 | -0,36468 |
| ***Ccnd2*** | -0,4796 | 0,051448 | -0,06473 | -0,13573 | -0,03335 |
| ***Dct*** | -0,20203 | -0,06099 | -0,19029 | 0,023291 | -0,25082 |
| ***Med1*** | 0,053763 | -0,41944 | -0,10408 | -0,04778 | -0,19516 |
| ***Rb1*** | 0,043385 | -0,50635 | -0,07898 | 0,076591 | -0,27296 |
| ***Hsp90b1*** | -0,02465 | -0,28963 | -0,1934 | -0,04917 | -0,19621 |
| ***Aldh1a3*** | -0,15936 | -0,26665 | -0,27743 | -0,07322 | 0,015636 |
| ***Mapk3*** | -0,12178 | -0,2083 | -0,11816 | -0,24199 | -0,08334 |
| ***Mcm7*** | 0,340481 | 0,024752 | -0,54627 | -0,39067 | -0,2028 |
| ***Gstm3*** | -0,08747 | -0,54621 | -0,07372 | 0,03858 | -0,15077 |
| ***Tgfb3*** | -0,19636 | 0,006423 | 0,064986 | -0,53177 | -0,25287 |
| ***Aldh9a1*** | -0,30241 | 0,092659 | -0,28733 | -0,27964 | -0,1646 |
| ***Cdk2*** | -0,00943 | -0,50122 | -0,22533 | -0,18658 | -0,01907 |
| ***Cdk4*** | -0,10627 | -0,41942 | -0,26368 | -0,14062 | -0,1066 |
| ***Cdk6*** | 0,062813 | -0,95762 | -0,29683 | -0,04338 | 0,167408 |
| ***Gsto1*** | -0,17536 | -0,37914 | -0,10067 | -0,17171 | -0,25074 |
| ***Gstm2*** | -0,39018 | 0,482035 | -0,48423 | -0,34486 | -0,40086 |
| ***Aldh1a1*** | 0,149176 | -0,68979 | -0,46523 | -0,18232 | 0,010048 |
| ***Tff1*** | -0,09906 | -0,22898 | -0,79619 | -0,38787 | 0,279885 |
| ***Chek1*** | -0,182 | -0,19946 | -0,78685 | -0,21979 | 0,141936 |
| ***Aldh1b1*** | -0,4054 | -0,14953 | -0,38866 | -0,21044 | -0,2086 |
| ***Pola1*** | -0,20212 | -0,43305 | -0,60189 | -0,36994 | 0,12439 |
| ***Gstt2/gstt2b*** | -0,15546 | -0,17558 | -0,17329 | -0,54045 | -0,45832 |
| ***Tp53*** | -0,03313 | -0,694 | -0,51593 | -0,13647 | -0,15904 |
| ***Gstm1*** | -0,39472 | -0,33612 | -0,22027 | -0,25434 | -0,4292 |
| ***Esr1*** | -0,8671 | 0,042478 | -0,57455 | -0,17213 | -0,09752 |
| ***Apaf1*** | -0,37001 | -0,05315 | -0,54746 | -0,3873 | -0,39245 |
| ***Nfib*** | -0,15273 | -0,73237 | -0,48208 | -0,16453 | -0,2606 |
| ***Aldh3a2*** | -0,07275 | -0,76701 | -0,39468 | -0,41599 | -0,25463 |
| ***Nqo2*** | -0,46226 | -0,5755 | -0,58456 | -0,18769 | -0,16149 |
| ***Nfia*** | -0,23398 | -1,26555 | -0,42493 | -0,04115 | -0,10567 |
| ***Atm*** | 0,040639 | -0,85815 | -0,47161 | -0,46039 | -0,36904 |
| ***Fas*** | -0,26124 | -0,37775 | -0,62657 | -0,563 | -0,29446 |
| ***Rarb*** | -0,53431 | -0,65308 | -0,60431 | -0,37019 | -0,04105 |
| ***Aldh8a1*** | -0,129 | 0,635762 | -0,37848 | -0,93457 | -1,40599 |
| ***Dhfr*** | -0,07265 | -0,81539 | -0,74403 | -0,54655 | -0,57047 |
| ***Ccna2*** | -0,31525 | 0,008913 | -1,83718 | -0,40253 | -0,31208 |
| ***Rxrg*** | -0,82884 | -0,95299 | -0,79999 | -0,3721 | -0,44445 |
| ***Mgst3*** | -0,9771 | -0,44419 | -1,12997 | -0,54633 | -0,3664 |
| ***Rbl1*** | -0,58928 | -0,91153 | -1,10301 | -0,54222 | -0,41017 |
| ***Nqo1*** | -1,16194 | -0,75202 | -0,53121 | -0,54596 | -0,73791 |
| ***Ccnd1*** | -2,06339 | -1,47819 | -1,8214 | -1,3163 | -0,59342 |

**Table S10:** Activation Z-score of genes involved in xenobiotic metabolism general signaling pathway male vs. female. Activation Z-score was calculated with IPA software from Qiagen.

| **Pathway Summary** | 0 | -1,342 | -1,134 | -1,219 | -2,2 |
| --- | --- | --- | --- | --- | --- |
| **genes in the xenobiotic metabolism general signaling pathway network** | **0 h** | **24 h** | **48 h** | **72 h** | **96 h** |
| ***Ugt2b17*** | 0,557028 | 2,213161 | 1,292475 | 0,591189 | -0,39135 |
| ***Hmox1*** | 0,452339 | 0,564182 | 1,329974 | 0,634012 | 0,537807 |
| ***Gstp1*** | 1,882254 | 0,337857 | 0,177296 | 0,244776 | 0,194589 |
| ***Map3k6*** | 0,451248 | 0,128543 | 0,407265 | 0,695676 | -0,01789 |
| ***Ugt2b28*** | 0,774388 | 0,659801 | 0,273055 | -0,19352 | -0,16884 |
| ***Mras*** | 0,174646 | 1,003178 | -0,15429 | -0,08634 | 0,102783 |
| ***Prkcg*** | -0,04543 | 0,032092 | 0,447063 | 0,40637 | 0,164773 |
| ***Map2k1*** | 0,13442 | 0,207022 | 0,290068 | 0,177577 | 0,173668 |
| ***Map2k2*** | 0,575392 | -0,15963 | 0,11357 | 0,344424 | 0,071213 |
| ***Map3k3*** | 0,286673 | 0,292287 | 0,040959 | 0,085609 | 0,119859 |
| ***Map3k12*** | -0,03199 | 0,362781 | 0,120343 | 0,113524 | 0,199336 |
| ***Abca4*** | -0,22516 | 0,274006 | 0,336817 | 0,065528 | 0,289708 |
| ***Prkcd*** | 0,074277 | 0,348892 | 0,246503 | 0,028518 | 0,008762 |
| ***Mapk11*** | 0,344524 | 0,124728 | -0,13352 | 0,077608 | 0,278353 |
| ***Mapk7*** | 0,108125 | 0,270136 | 0,024832 | 0,058539 | 0,205462 |
| ***Map2k3*** | 0,106438 | 0,031734 | 0,048844 | 0,2398 | 0,194726 |
| ***Map2k4*** | 0,061557 | 0,024216 | 0,304808 | 0,244426 | -0,0187 |
| ***Rala*** | 0,0355 | 0,107634 | 0,179678 | 0,155122 | 0,08952 |
| ***Rasd2*** | -0,06273 | 0,27061 | 0,160291 | -0,01052 | 0,20936 |
| ***Ralb*** | 0,014308 | 0,182199 | -0,06845 | 0,121461 | 0,291222 |
| ***Map3k8*** | 0,086216 | 0,168118 | -0,02436 | 0,13463 | 0,117251 |
| ***Pik3c2a*** | 0,005251 | -0,19112 | 0,220942 | 0,330514 | 0,110934 |
| ***Map3k2*** | 0,274096 | 0,2759 | 0,178762 | -0,03937 | -0,21503 |
| ***Ftl*** | -0,00938 | 0,144546 | 0,162584 | 0,087036 | 0,082192 |
| ***Slc51a*** | -0,06567 | 0,345798 | 0,206038 | -0,09768 | 0,074967 |
| ***Pik3r5*** | -0,01583 | 0,191325 | 0,133672 | -0,09727 | 0,247275 |
| ***Crebbp*** | 0,186457 | 0,187408 | -0,11777 | 0,234118 | -0,05094 |
| ***Ugt2b7*** | 0,55257 | -0,32078 | 0,463908 | -0,14661 | -0,11281 |
| ***Mapk12*** | 0,137599 | 0,207914 | 0,030111 | -0,05141 | 0,079893 |
| ***Pik3c2b*** | 0,118141 | 0,169816 | 0,018153 | 0,04807 | 0,042186 |
| ***Mapk8*** | -0,004 | 0,261262 | 0,311823 | 0,025523 | -0,20447 |
| ***Pik3r6*** | -0,14476 | 0,123504 | 0,067777 | 0,110262 | 0,229527 |
| ***Slc9c1*** | 0,096478 | -0,01792 | 0,199211 | 0,182054 | -0,07693 |
| ***Gclc*** | 0,079908 | -0,1071 | 0,082775 | 0,102428 | 0,214729 |
| ***Raf1*** | 0,095374 | 0,446542 | -0,06254 | 0,026048 | -0,14962 |
| ***Ep300*** | 0,223775 | -0,03702 | 0,045473 | 0,279534 | -0,16359 |
| ***Map3k1*** | -0,22776 | 0,608811 | 0,12805 | -0,11516 | -0,05375 |
| ***Hras*** | 0,40146 | -0,23578 | -0,11532 | 0,234695 | 0,023973 |
| ***Map3k9*** | -0,34736 | 0,368542 | -0,00779 | 0,055689 | 0,234789 |
| ***Prkch*** | 0,199389 | -0,01281 | 0,351147 | -0,02797 | -0,20844 |
| ***Abca2*** | -0,09513 | 0,107184 | 0,078279 | 0,259134 | -0,06714 |
| ***Cul3*** | 0,020882 | 0,207118 | 0,034457 | 0,08637 | -0,07733 |
| ***Rasd1*** | 0,052517 | 0,084486 | 0,002894 | 0,137336 | -0,00633 |
| ***Map3k14*** | 0,141119 | -0,18086 | -0,05305 | 0,247592 | 0,099534 |
| ***Mapk13*** | -0,10682 | 0,118433 | 0,036508 | -0,03219 | 0,2349 |
| ***Ugt8*** | 0,044986 | 0,16308 | 0,036966 | 0,052765 | -0,06637 |
| ***Eif2ak3*** | 0,08824 | -0,08293 | 0,027969 | 0,097027 | 0,078312 |
| ***Map3k7*** | 0,331366 | -0,11687 | 0,027811 | 0,048484 | -0,09728 |
| ***Arnt*** | -0,02327 | 0,187907 | -0,00335 | -0,04279 | 0,055936 |
| ***Prkcq*** | 0,10399 | -0,25314 | 0,039609 | 0,162102 | 0,11465 |
| ***Prkd1*** | -0,00044 | 0,078895 | -0,14158 | 0,042999 | 0,164733 |
| ***Map3k4*** | 0,053287 | -0,08844 | 0,127158 | 0,081906 | -0,0294 |
| ***Pik3r2*** | 0,25572 | -0,09114 | 0,075034 | -0,03299 | -0,0685 |
| ***Nr1h2*** | -0,10864 | 0,132925 | 0,225347 | -0,03865 | -0,07735 |
| ***Rxra*** | 0,193827 | 0,169862 | 0,015074 | 0,019906 | -0,27327 |
| ***Akt3*** | -0,24716 | -0,00756 | 0,243473 | 0,466583 | -0,35147 |
| ***Keap1*** | 0,115311 | 0,018182 | 0,044694 | 0,072474 | -0,14782 |
| ***Maf*** | -0,23297 | 0,124636 | 0,049369 | 0,069298 | 0,088888 |
| ***Nras*** | -0,01512 | -0,20668 | 0,095093 | 0,160952 | 0,042765 |
| ***Map3k11*** | 0,051429 | -0,139 | 0,06189 | 0,035027 | 0,056671 |
| ***Ugt2a1*** | -0,01249 | 0,005613 | 0,013367 | 0,025455 | 0,025249 |
| ***Map3k5*** | 0,220329 | -0,46408 | -0,06448 | 0,353496 | -0,00104 |
| ***Prkcb*** | -0,12369 | 0,164085 | -0,09661 | 0,022314 | 0,069768 |
| ***Map3k15*** | -0,3586 | 0,142139 | 0,032649 | -0,0291 | 0,238245 |
| ***Ahr*** | -0,14658 | 0,313906 | -0,04025 | -0,03685 | -0,07687 |
| ***Mgst2*** | 0,091526 | -0,15884 | 0,018484 | -0,07328 | 0,127777 |
| ***Rap1b*** | -0,10425 | 0,078944 | -0,00589 | 0,027729 | -0,0215 |
| ***Map2k7*** | 0,19363 | -0,09578 | -0,15608 | 0,019885 | -0,01475 |
| ***Pik3r3*** | 0,293126 | -0,01107 | -0,15181 | -0,15931 | -0,02902 |
| ***Prkci*** | 0,024551 | -0,43889 | 0,186525 | 0,082533 | 0,04227 |
| ***Mgst1*** | 0,106844 | 0,117306 | -0,03641 | -0,20413 | -0,0961 |
| ***Mapk1*** | 0,044576 | -0,16036 | 0,093211 | 0,032593 | -0,13623 |
| ***Gstm3*** | -0,14577 | -0,12861 | 0,088728 | 0,337619 | -0,29394 |
| ***Pik3ca*** | 0,052469 | 0,079205 | -0,0719 | -0,11625 | -0,08746 |
| ***Rap2a*** | 0,51035 | -0,04705 | -0,10372 | -0,20105 | -0,30269 |
| ***Mapk14*** | 0,220987 | -0,26318 | 0,014724 | -0,02297 | -0,09842 |
| ***Gsto2*** | -0,2979 | -0,13784 | -0,0402 | 0,048601 | 0,235744 |
| ***Pik3cd*** | -0,26532 | 0,047348 | 0,051176 | 0,075089 | -0,11023 |
| ***Nfe2l2*** | 0,237238 | 0,092282 | -0,35333 | -0,29459 | 0,071303 |
| ***Rras*** | -0,04157 | -0,40855 | -0,11514 | 0,07881 | 0,186698 |
| ***Pik3r1*** | 0,090286 | -0,57779 | -0,2358 | 0,144529 | 0,263983 |
| ***Ppara*** | 0,284157 | 0,016818 | -0,01524 | -0,10652 | -0,51136 |
| ***Prkce*** | 0,064813 | -0,4592 | -0,03295 | 0,190098 | -0,12429 |
| ***Akt2*** | -0,09815 | -0,15659 | -0,00876 | -0,09686 | -0,01344 |
| ***Gstz1*** | -0,02332 | 0,812286 | -0,42883 | -0,45182 | -0,28936 |
| ***Pik3cg*** | -0,02541 | -0,17348 | -0,01282 | 0,058653 | -0,23801 |
| ***Gstm6*** | -0,17145 | 0,278406 | -0,21578 | -0,06966 | -0,26243 |
| ***Gstm5*** | -0,13549 | -0,09147 | -0,00525 | -0,06958 | -0,14526 |
| ***Gsta3*** | 0,082941 | 0,207171 | -0,25599 | -0,34971 | -0,13187 |
| ***Pik3r4*** | -0,00762 | 0,057253 | -0,08123 | -0,32561 | -0,09624 |
| ***Map3k10*** | -0,09285 | -0,20732 | 0,037417 | -0,1273 | -0,10611 |
| ***Pik3c3*** | -0,23607 | 0,073196 | -0,14118 | -0,14439 | -0,05641 |
| ***Gsta5*** | -0,26097 | -0,83708 | 0,265944 | 0,378938 | -0,0793 |
| ***Gstk1*** | -0,18235 | 0,980101 | -0,47865 | -0,43221 | -0,41973 |
| ***Gstm4*** | -0,10794 | -0,03032 | 0,081005 | -0,06848 | -0,40839 |
| ***Akt1*** | -0,12742 | -0,47145 | -0,12531 | -0,0356 | 0,152021 |
| ***Rap2b*** | -0,53235 | 0,177452 | 0,098047 | -0,30301 | -0,10827 |
| ***Ugt2b10*** | -0,06221 | -0,04937 | 0,124047 | -0,33365 | -0,35 |
| ***Mapk3*** | -0,12178 | -0,2083 | -0,11816 | -0,24199 | -0,08334 |
| ***Prkcz*** | 0,069884 | -0,36703 | -0,18978 | -0,14472 | -0,18557 |
| ***Gstm3*** | -0,08747 | -0,54621 | -0,07372 | 0,03858 | -0,15077 |
| ***Rap1a*** | -0,09409 | -0,14484 | -0,13954 | -0,2421 | -0,26123 |
| ***Mapk9*** | -0,00623 | -0,34585 | -0,12821 | -0,25198 | -0,15795 |
| ***Eras*** | -0,10103 | -0,09304 | -0,48438 | -0,19299 | -0,02703 |
| ***Nr1h3*** | 0,045567 | -0,18155 | -0,28072 | -0,24717 | -0,25165 |
| ***Map2k6*** | -0,49451 | 0,296523 | -0,02225 | -0,42464 | -0,38477 |
| ***Map2k5*** | 0,011184 | -0,51421 | -0,24865 | -0,09975 | -0,20663 |
| ***Gsto1*** | -0,17536 | -0,37914 | -0,10067 | -0,17171 | -0,25074 |
| ***Kras*** | -0,26815 | -0,15401 | -0,19245 | -0,26561 | -0,23842 |
| ***Gstm2*** | -0,39018 | 0,482035 | -0,48423 | -0,34486 | -0,40086 |
| ***Pik3c2g*** | -0,48782 | -0,08919 | -0,10013 | -0,11043 | -0,35399 |
| ***Pik3cb*** | -0,21367 | -0,8372 | -0,21036 | -0,02392 | -0,02048 |
| ***Gstt2/gstt2b*** | -0,15546 | -0,17558 | -0,17329 | -0,54045 | -0,45832 |
| ***Nr1i2*** | -0,21815 | -0,37089 | -0,35606 | -0,21664 | -0,3979 |
| ***Gstm1*** | -0,39472 | -0,33612 | -0,22027 | -0,25434 | -0,4292 |
| ***Rras2*** | -0,07237 | -0,80744 | -0,56238 | -0,24113 | -0,04987 |
| ***Nr1h4*** | 0,255714 | -0,60739 | -0,50502 | -0,55488 | -0,43199 |
| ***Slc51b*** | -0,94048 | -0,25946 | -0,43251 | -0,19989 | -0,11032 |
| ***Nqo2*** | -0,46226 | -0,5755 | -0,58456 | -0,18769 | -0,16149 |
| ***Map3k13*** | -0,14057 | -1,35639 | -0,58339 | 0,046662 | -0,05362 |
| ***Nr1i3*** | -0,76069 | 0,127178 | -0,24139 | -0,64651 | -0,60801 |
| ***Prkd3*** | -0,08954 | -0,27329 | -0,74934 | -0,84385 | -0,69256 |
| ***Prkca*** | -0,78907 | -1,45442 | -0,55646 | -0,30357 | -0,30056 |
| ***Mgst3*** | -0,9771 | -0,44419 | -1,12997 | -0,54633 | -0,3664 |
| ***Nqo1*** | -1,16194 | -0,75202 | -0,53121 | -0,54596 | -0,73791 |

**Table S11:** Activation Z-score of genes involved in the xenobiotic metabolism PXR signaling male vs. female. Activation Z-score was calculated with IPA software from Qiagen.

| **Pathway Summary** | -3,571 | -0,246 | -2,469 | -1,732 | -3,727 |
| --- | --- | --- | --- | --- | --- |
| **genes in the xenobiotic metabolism PXR signaling pathway network** | **0 h** | **24 h** | **48 h** | **72 h** | **96 h** |
| ***Ces3*** | 0,72206 | 1,805008 | 2,492906 | 2,115416 | 1,127898 |
| ***Ugt2b17*** | 0,557028 | 2,213161 | 1,292475 | 0,591189 | -0,39135 |
| ***Gstp1*** | 1,882254 | 0,337857 | 0,177296 | 0,244776 | 0,194589 |
| ***Ces4a*** | 1,213557 | 0,877347 | 0,104064 | -0,06546 | -0,01421 |
| ***Ust*** | -0,16453 | -0,06222 | 0,274577 | 0,854592 | 0,955727 |
| ***Scand1*** | 0,32096 | 0,306684 | 0,417708 | 0,224246 | 0,219998 |
| ***Ugt2b28*** | 0,774388 | 0,659801 | 0,273055 | -0,19352 | -0,16884 |
| ***Hs6st2*** | 0,161186 | 0,281571 | 0,278357 | 0,174943 | 0,175664 |
| ***Aldh4a1*** | -0,18994 | 1,145047 | 0,371212 | 0,082255 | -0,3707 |
| ***Prkcg*** | -0,04543 | 0,032092 | 0,447063 | 0,40637 | 0,164773 |
| ***Hs3st2*** | -0,05167 | 0,29722 | 0,192894 | 0,118949 | 0,392014 |
| ***Ppp1r14a*** | 0,169126 | 0,486704 | 0,296002 | -0,20894 | 0,158245 |
| ***Ppp1r14c*** | 0,085778 | 0,27538 | 0,221505 | 0,184427 | 0,07246 |
| ***Smox*** | -0,38514 | 0,265107 | 0,58581 | 0,252859 | 0,094358 |
| ***Grip1*** | 0,029567 | 0,205048 | 0,124745 | 0,268464 | 0,161213 |
| ***Aldh1l1*** | -0,0806 | 1,174822 | 0,301611 | -0,27572 | -0,37022 |
| ***Ndst3*** | 0,133059 | 0,19889 | 0,028859 | 0,32905 | 0,043503 |
| ***Ncor2*** | -0,03862 | 0,293661 | 0,141303 | 0,190168 | 0,142945 |
| ***Prkcd*** | 0,074277 | 0,348892 | 0,246503 | 0,028518 | 0,008762 |
| ***Prkar1b*** | -0,0144 | 0,23196 | 0,228917 | 0,084269 | 0,096835 |
| ***Hs3st5*** | -0,0727 | 0,050861 | 0,167011 | 0,150199 | 0,30591 |
| ***Hsp90aa1*** | 0,502608 | -0,32229 | 0,178781 | 0,094177 | 0,142744 |
| ***Ces1e*** | 0,227733 | 1,355056 | 0,162471 | -0,33273 | -0,8325 |
| ***Cited2*** | 0,380948 | -0,21694 | 0,046022 | 0,113582 | 0,256108 |
| ***Ppp1r11*** | 0,197113 | -0,14215 | 0,247016 | 0,22562 | 0,042782 |
| ***Hs6st1*** | 0,087558 | -0,02053 | 0,183297 | 0,348937 | -0,04189 |
| ***Chst3*** | 0,206098 | 0,116151 | 0,150104 | 0,04956 | 0,016728 |
| ***Chst11*** | 0,105503 | -0,00788 | -0,07606 | 0,405144 | 0,056785 |
| ***Prkar2b*** | -0,27859 | 0,108796 | 0,194686 | 0,282446 | 0,160946 |
| ***Ppp1r3d*** | -0,09352 | 0,057046 | 0,103671 | -0,0037 | 0,404696 |
| ***Aldh3a1*** | -0,01797 | 0,141252 | 0,02739 | 0,166023 | 0,14445 |
| ***Hs3st6*** | -0,00883 | 0,312148 | 0,070264 | 0,049472 | 0,037658 |
| ***Aldh1l2*** | -0,09203 | -0,04787 | 0,308585 | 0,377108 | -0,09043 |
| ***Hs6st3*** | 0,002158 | 0,135798 | 0,279591 | -0,05443 | 0,087057 |
| ***Crebbp*** | 0,186457 | 0,187408 | -0,11777 | 0,234118 | -0,05094 |
| ***Ugt2b7*** | 0,55257 | -0,32078 | 0,463908 | -0,14661 | -0,11281 |
| ***Sra1*** | 0,225891 | 0,076737 | 0,150775 | -0,00428 | -0,01777 |
| ***Esd*** | 0,071244 | -0,29248 | 0,325026 | 0,316411 | 0,004847 |
| ***Aldh7a1*** | 0,220354 | 0,819824 | -0,32258 | -0,21583 | -0,08076 |
| ***Ppp1r10*** | -0,17764 | 0,03657 | 0,285491 | 0,116294 | 0,137999 |
| ***Aldh16a1*** | 0,101859 | 0,218341 | 0,078239 | -0,04107 | 0,029463 |
| ***Hs3st1*** | -0,32496 | 0,260942 | 0,165804 | -0,06383 | 0,345892 |
| ***Ep300*** | 0,223775 | -0,03702 | 0,045473 | 0,279534 | -0,16359 |
| ***Nos2*** | 0,047766 | 0,196393 | 0,014087 | -0,16033 | 0,249916 |
| ***Chst4*** | 0,034536 | 0,158027 | 0,089642 | -0,26086 | 0,3187 |
| ***Ces5a*** | -0,0177 | 0,034085 | 0,112758 | 0,109323 | 0,063639 |
| ***Prkch*** | 0,199389 | -0,01281 | 0,351147 | -0,02797 | -0,20844 |
| ***Chst12*** | -0,21381 | 0,102366 | 0,163994 | 0,073405 | 0,169455 |
| ***Ces2*** | 0,229821 | 0,155655 | 0,105746 | 0,065959 | -0,28689 |
| ***Ugt8*** | 0,044986 | 0,16308 | 0,036966 | 0,052765 | -0,06637 |
| ***Aldh1a2*** | -0,04424 | 0,074551 | -0,0644 | 0,130846 | 0,115865 |
| ***Hs3st4*** | -0,30244 | 0,086334 | 0,216179 | 0,028372 | 0,17809 |
| ***Ppp1r12a*** | 0,098903 | -0,20144 | 0,063026 | 0,192264 | 0,039148 |
| ***Sult1b1*** | -0,06713 | 0,322959 | 0,273638 | -0,20484 | -0,14874 |
| ***Ncor1*** | 0,187013 | 0,035806 | -0,0549 | 0,083873 | -0,08182 |
| ***Prkcq*** | 0,10399 | -0,25314 | 0,039609 | 0,162102 | 0,11465 |
| ***Hs3st3b1*** | 0,057123 | -0,06301 | -0,07265 | 0,12012 | 0,113977 |
| ***Prkd1*** | -0,00044 | 0,078895 | -0,14158 | 0,042999 | 0,164733 |
| ***Rxra*** | 0,193827 | 0,169862 | 0,015074 | 0,019906 | -0,27327 |
| ***Ncoa1*** | 0,119747 | 0,011103 | -0,03914 | 0,078061 | -0,04566 |
| ***Ppp1r3a*** | -0,06288 | 0,330007 | 0,081864 | 0,13826 | -0,36336 |
| ***Ugt2a1*** | -0,01249 | 0,005613 | 0,013367 | 0,025455 | 0,025249 |
| ***Cat*** | 0,079423 | 0,133675 | -0,05435 | -0,08199 | -0,03185 |
| ***Prkcb*** | -0,12369 | 0,164085 | -0,09661 | 0,022314 | 0,069768 |
| ***Chst2*** | -0,05745 | 0,132523 | -0,02665 | 0,049296 | -0,06265 |
| ***Hsp90ab1*** | 0,153701 | -0,42093 | 0,171052 | 0,082244 | 0,047582 |
| ***Mgst2*** | 0,091526 | -0,15884 | 0,018484 | -0,07328 | 0,127777 |
| ***Snw1*** | -0,03129 | 0,078455 | -0,02802 | 0,012924 | -0,07194 |
| ***Nrip1*** | 0,062801 | -0,10827 | 0,210467 | -0,17986 | -0,03364 |
| ***Sult1c3*** | -0,34907 | 0,08808 | 0,041571 | -0,07432 | 0,237151 |
| ***Ppp1r14b*** | 0,142972 | -0,39431 | 0,030588 | 0,043426 | 0,116188 |
| ***Sult2b1*** | -0,01121 | 0,160563 | -0,1065 | -0,12214 | 0,008768 |
| ***Ppp1r14d*** | -0,22588 | 0,094183 | -0,09657 | -0,14416 | 0,286068 |
| ***Chst7*** | 0,044349 | 0,033325 | 0,080503 | -0,22675 | -0,02035 |
| ***Chst13*** | -0,00786 | -0,2197 | 0,066671 | 0,037978 | 0,02233 |
| ***Camk2d*** | -0,18448 | -0,3164 | 0,239786 | 0,235981 | -0,07561 |
| ***Prkci*** | 0,024551 | -0,43889 | 0,186525 | 0,082533 | 0,04227 |
| ***Mgst1*** | 0,106844 | 0,117306 | -0,03641 | -0,20413 | -0,0961 |
| ***Prkar2a*** | 0,387154 | -0,67441 | 0,07029 | 0,328312 | -0,22801 |
| ***Ppm1a*** | -0,02629 | 0,017805 | 0,02218 | -0,05256 | -0,10155 |
| ***Gstm3*** | -0,14577 | -0,12861 | 0,088728 | 0,337619 | -0,29394 |
| ***Ndst2*** | -0,20442 | -0,07856 | 0,042657 | 0,150329 | -0,05868 |
| ***Ppp1ca*** | -0,23883 | -0,18626 | 0,072024 | 0,083722 | 0,101551 |
| ***Camk2g*** | 0,031426 | 0,033648 | -0,06763 | -0,10459 | -0,07219 |
| ***Ces1*** | -0,12385 | 0,340526 | 0,219336 | 0,011072 | -0,6343 |
| ***Prkar1a*** | -0,19423 | -0,07836 | 0,018844 | 0,029118 | 0,03697 |
| ***Gsto2*** | -0,2979 | -0,13784 | -0,0402 | 0,048601 | 0,235744 |
| ***Chst10*** | -0,37018 | -0,01537 | -0,1227 | 0,059074 | 0,20577 |
| ***Chst15*** | -0,2913 | -0,03826 | 0,432391 | 0,094591 | -0,4521 |
| ***Prkag1*** | 0,015082 | -0,1281 | -0,02855 | -0,07731 | -0,04722 |
| ***Aldh18a1*** | 0,204397 | -0,38172 | -0,32766 | -0,0109 | 0,227284 |
| ***Hs3st3a1*** | -0,31821 | -0,09785 | -0,1328 | 0,081268 | 0,162928 |
| ***Prkacb*** | -0,17137 | -0,11709 | 0,152978 | -0,07941 | -0,11836 |
| ***Aldh2*** | -0,05226 | 0,209599 | -0,10349 | -0,25722 | -0,14803 |
| ***Gal3st2*** | -0,28091 | -0,11758 | 0,0691 | -0,27645 | 0,248223 |
| ***Prkce*** | 0,064813 | -0,4592 | -0,03295 | 0,190098 | -0,12429 |
| ***Gstz1*** | -0,02332 | 0,812286 | -0,42883 | -0,45182 | -0,28936 |
| ***Camk2a*** | -0,22312 | -0,29265 | 0,211607 | -0,0604 | -0,0165 |
| ***Cdk5*** | 0,164429 | -0,25828 | -0,18123 | -0,00867 | -0,10523 |
| ***Chst1*** | -0,25252 | 0,036369 | 0,10231 | -0,25253 | -0,04909 |
| ***Aldh6a1*** | -0,12568 | 0,70767 | -0,35314 | -0,33851 | -0,32771 |
| ***Gstm6*** | -0,17145 | 0,278406 | -0,21578 | -0,06966 | -0,26243 |
| ***Gstm5*** | -0,13549 | -0,09147 | -0,00525 | -0,06958 | -0,14526 |
| ***Gsta3*** | 0,082941 | 0,207171 | -0,25599 | -0,34971 | -0,13187 |
| ***Prkaca*** | 0,087291 | -0,5 | -0,14718 | 0,014032 | 0,092124 |
| ***Aldh3b1*** | -0,26231 | 0,03008 | -0,35622 | 0,084234 | 0,031547 |
| ***Gsta5*** | -0,26097 | -0,83708 | 0,265944 | 0,378938 | -0,0793 |
| ***Gstk1*** | -0,18235 | 0,980101 | -0,47865 | -0,43221 | -0,41973 |
| ***Gstm4*** | -0,10794 | -0,03032 | 0,081005 | -0,06848 | -0,40839 |
| ***Aldh5a1*** | -0,01626 | 0,086619 | -0,35096 | -0,1413 | -0,15137 |
| ***Hs2st1*** | -0,17552 | -0,37367 | -0,01396 | -0,06005 | 0,047499 |
| ***Camk2b*** | -0,63734 | -0,09794 | 0,063998 | 0,042174 | 0,037704 |
| ***Ndst1*** | -0,12359 | -0,08368 | -0,05185 | -0,22874 | -0,16588 |
| ***Ppp1cb*** | -0,1511 | -0,07912 | -0,12337 | -0,16331 | -0,14493 |
| ***Ugt2b10*** | -0,06221 | -0,04937 | 0,124047 | -0,33365 | -0,35 |
| ***Ndst4*** | -0,46189 | -0,18419 | -0,0894 | 0,068262 | -0,03833 |
| ***Ppp1r7*** | -0,21864 | -0,34749 | -0,07285 | -0,08013 | 0,007066 |
| ***Med1*** | 0,053763 | -0,41944 | -0,10408 | -0,04778 | -0,19516 |
| ***Cyp3a7*** | -0,36401 | -0,43583 | -0,05304 | 0,112209 | 0,001546 |
| ***Sult4a1*** | -0,03304 | 0,183221 | -0,20444 | -0,4542 | -0,2331 |
| ***Hsp90b1*** | -0,02465 | -0,28963 | -0,1934 | -0,04917 | -0,19621 |
| ***Aldh1a3*** | -0,15936 | -0,26665 | -0,27743 | -0,07322 | 0,015636 |
| ***Cyp2c9*** | -0,67462 | 0,257784 | 0,167694 | -0,2441 | -0,31643 |
| ***Prkcz*** | 0,069884 | -0,36703 | -0,18978 | -0,14472 | -0,18557 |
| ***Gstm3*** | -0,08747 | -0,54621 | -0,07372 | 0,03858 | -0,15077 |
| ***Ppp1r3c*** | -0,20615 | 0,757792 | 0,253787 | -0,85739 | -0,80804 |
| ***Aldh9a1*** | -0,30241 | 0,092659 | -0,28733 | -0,27964 | -0,1646 |
| ***Cdk2*** | -0,00943 | -0,50122 | -0,22533 | -0,18658 | -0,01907 |
| ***Dnajc7*** | -0,04284 | -0,5412 | 0,021931 | -0,29237 | -0,12199 |
| ***Ppp1cc*** | -0,26975 | -0,30838 | -0,01636 | -0,13976 | -0,32732 |
| ***Gsto1*** | -0,17536 | -0,37914 | -0,10067 | -0,17171 | -0,25074 |
| ***Gstm2*** | -0,39018 | 0,482035 | -0,48423 | -0,34486 | -0,40086 |
| ***Abcc3*** | -0,1613 | -0,49806 | -0,1044 | -0,23767 | -0,1381 |
| ***Abcc2*** | 0,030509 | -0,50224 | -0,16845 | -0,25012 | -0,25719 |
| ***Aldh1a1*** | 0,149176 | -0,68979 | -0,46523 | -0,18232 | 0,010048 |
| ***Aldh1b1*** | -0,4054 | -0,14953 | -0,38866 | -0,21044 | -0,2086 |
| ***Sult1e1*** | -0,77274 | 0,088906 | 0,193589 | -0,32624 | -0,62783 |
| ***Gstt2/gstt2b*** | -0,15546 | -0,17558 | -0,17329 | -0,54045 | -0,45832 |
| ***Nr1i2*** | -0,21815 | -0,37089 | -0,35606 | -0,21664 | -0,3979 |
| ***Gstm1*** | -0,39472 | -0,33612 | -0,22027 | -0,25434 | -0,4292 |
| ***Aldh3a2*** | -0,07275 | -0,76701 | -0,39468 | -0,41599 | -0,25463 |
| ***Prkag2*** | -0,50905 | -0,84728 | -0,13855 | -0,19606 | -0,26978 |
| ***Aldh8a1*** | -0,129 | 0,635762 | -0,37848 | -0,93457 | -1,40599 |
| ***Sult1d1*** | -0,75893 | -0,67835 | -0,02509 | -0,09733 | -0,65697 |
| ***Maob*** | -0,68574 | -0,33792 | -0,72165 | -0,47743 | -0,326 |
| ***Prkd3*** | -0,08954 | -0,27329 | -0,74934 | -0,84385 | -0,69256 |
| ***Ppargc1a*** | -0,79412 | -0,38501 | -0,49487 | -0,42794 | -0,66493 |
| ***Prkca*** | -0,78907 | -1,45442 | -0,55646 | -0,30357 | -0,30056 |
| ***Mgst3*** | -0,9771 | -0,44419 | -1,12997 | -0,54633 | -0,3664 |
| ***Ces1g*** | -0,50341 | 0,343339 | -0,57869 | -1,01469 | -1,90591 |
| ***Maoa*** | -0,70329 | -0,4951 | -1,32169 | -1,29354 | -0,54919 |
| ***Sult1c2*** | -1,32587 | -0,66 | -1,16059 | -0,84454 | -0,46645 |
| ***Abcb1*** | -3,15988 | -0,99045 | -1,36827 | -1,08103 | -0,80522 |
| ***Sult2a1*** | -3,65458 | -0,33344 | -1,47038 | -1,9286 | -2,14337 |
| ***Cyp2b6*** | -4,87545 | -1,58102 | -1,3552 | -1,03239 | -1,02875 |
| ***Cyp3a5*** | -4,0533 | -3,72033 | -2,12901 | 0,626905 | -0,99918 |
| ***Cyp2c8*** | -3,0275 | -2,40431 | -1,64767 | -1,56727 | -2,10398 |
| ***Sult3a1/sult3a2*** | -7,11037 | -3,91233 | -0,98212 | -0,60393 | -0,52936 |

**Table S12:** Activation Z-score of genes involved in the xenobiotic metabolism CAR signaling male vs. female. Activation Z-score was calculated with IPA software from Qiagen.

| **Pathway Summary** | -3,677 | -1,921 | -3,175 | -2,885 | -4,422 |
| --- | --- | --- | --- | --- | --- |
| **genes in the xenobiotic metabolism CAR signaling pathway network** | **0 h** | **24 h** | **48 h** | **72 h** | **96 h** |
| ***Ugt2b17*** | 0,557028 | 2,213161 | 1,292475 | 0,591189 | -0,39135 |
| ***Gstp1*** | 1,882254 | 0,337857 | 0,177296 | 0,244776 | 0,194589 |
| ***Egfr*** | 1,511161 | 0,917405 | 0,196501 | 0,031613 | 0,151305 |
| ***Ust*** | -0,16453 | -0,06222 | 0,274577 | 0,854592 | 0,955727 |
| ***Abcc1*** | -0,32994 | 0,570196 | 0,756808 | 0,448079 | 0,320496 |
| ***Cyp1a2*** | 0,066898 | 1,509168 | 1,215758 | 0,619908 | -1,80321 |
| ***Scand1*** | 0,32096 | 0,306684 | 0,417708 | 0,224246 | 0,219998 |
| ***Ppp2r5b*** | 0,54375 | 0,20016 | 0,209439 | 0,266752 | 0,210274 |
| ***Sod3*** | 0,25318 | 0,202585 | 0,439863 | 0,205194 | 0,278381 |
| ***Ugt2b28*** | 0,774388 | 0,659801 | 0,273055 | -0,19352 | -0,16884 |
| ***Hs6st2*** | 0,161186 | 0,281571 | 0,278357 | 0,174943 | 0,175664 |
| ***Aldh4a1*** | -0,18994 | 1,145047 | 0,371212 | 0,082255 | -0,3707 |
| ***Prkcg*** | -0,04543 | 0,032092 | 0,447063 | 0,40637 | 0,164773 |
| ***Map2k1*** | 0,13442 | 0,207022 | 0,290068 | 0,177577 | 0,173668 |
| ***Hs3st2*** | -0,05167 | 0,29722 | 0,192894 | 0,118949 | 0,392014 |
| ***Map2k2*** | 0,575392 | -0,15963 | 0,11357 | 0,344424 | 0,071213 |
| ***Grip1*** | 0,029567 | 0,205048 | 0,124745 | 0,268464 | 0,161213 |
| ***Cyp1a1*** | -0,0377 | 0,221035 | 0,38464 | 0,078008 | 0,130361 |
| ***Aldh1l1*** | -0,0806 | 1,174822 | 0,301611 | -0,27572 | -0,37022 |
| ***Ndst3*** | 0,133059 | 0,19889 | 0,028859 | 0,32905 | 0,043503 |
| ***Prkcd*** | 0,074277 | 0,348892 | 0,246503 | 0,028518 | 0,008762 |
| ***Fmo5*** | 0,273567 | 0,20543 | -0,03977 | 0,199263 | 0,02579 |
| ***Map2k3*** | 0,106438 | 0,031734 | 0,048844 | 0,2398 | 0,194726 |
| ***Rack1*** | 0,267469 | -0,30304 | 0,11933 | 0,380574 | 0,152543 |
| ***Map2k4*** | 0,061557 | 0,024216 | 0,304808 | 0,244426 | -0,0187 |
| ***Hs3st5*** | -0,0727 | 0,050861 | 0,167011 | 0,150199 | 0,30591 |
| ***Hsp90aa1*** | 0,502608 | -0,32229 | 0,178781 | 0,094177 | 0,142744 |
| ***Cited2*** | 0,380948 | -0,21694 | 0,046022 | 0,113582 | 0,256108 |
| ***Hs6st1*** | 0,087558 | -0,02053 | 0,183297 | 0,348937 | -0,04189 |
| ***Chst3*** | 0,206098 | 0,116151 | 0,150104 | 0,04956 | 0,016728 |
| ***Chst11*** | 0,105503 | -0,00788 | -0,07606 | 0,405144 | 0,056785 |
| ***Aldh3a1*** | -0,01797 | 0,141252 | 0,02739 | 0,166023 | 0,14445 |
| ***Hs3st6*** | -0,00883 | 0,312148 | 0,070264 | 0,049472 | 0,037658 |
| ***Aldh1l2*** | -0,09203 | -0,04787 | 0,308585 | 0,377108 | -0,09043 |
| ***Hs6st3*** | 0,002158 | 0,135798 | 0,279591 | -0,05443 | 0,087057 |
| ***Crebbp*** | 0,186457 | 0,187408 | -0,11777 | 0,234118 | -0,05094 |
| ***Ugt2b7*** | 0,55257 | -0,32078 | 0,463908 | -0,14661 | -0,11281 |
| ***Sra1*** | 0,225891 | 0,076737 | 0,150775 | -0,00428 | -0,01777 |
| ***Fmo9*** | 0,00859 | 0,002303 | 0,122003 | 0,195754 | 0,095376 |
| ***Aldh7a1*** | 0,220354 | 0,819824 | -0,32258 | -0,21583 | -0,08076 |
| ***Aldh16a1*** | 0,101859 | 0,218341 | 0,078239 | -0,04107 | 0,029463 |
| ***Hs3st1*** | -0,32496 | 0,260942 | 0,165804 | -0,06383 | 0,345892 |
| ***Ep300*** | 0,223775 | -0,03702 | 0,045473 | 0,279534 | -0,16359 |
| ***Nos2*** | 0,047766 | 0,196393 | 0,014087 | -0,16033 | 0,249916 |
| ***Chst4*** | 0,034536 | 0,158027 | 0,089642 | -0,26086 | 0,3187 |
| ***Src*** | -0,27544 | 0,024078 | 0,202661 | 0,159584 | 0,199357 |
| ***Prkch*** | 0,199389 | -0,01281 | 0,351147 | -0,02797 | -0,20844 |
| ***Chst12*** | -0,21381 | 0,102366 | 0,163994 | 0,073405 | 0,169455 |
| ***Ppp2r2c*** | -0,1749 | 0,026287 | 0,217966 | 0,089528 | 0,134401 |
| ***Ugt8*** | 0,044986 | 0,16308 | 0,036966 | 0,052765 | -0,06637 |
| ***Aldh1a2*** | -0,04424 | 0,074551 | -0,0644 | 0,130846 | 0,115865 |
| ***Hs3st4*** | -0,30244 | 0,086334 | 0,216179 | 0,028372 | 0,17809 |
| ***Sult1b1*** | -0,06713 | 0,322959 | 0,273638 | -0,20484 | -0,14874 |
| ***Prkcq*** | 0,10399 | -0,25314 | 0,039609 | 0,162102 | 0,11465 |
| ***Hs3st3b1*** | 0,057123 | -0,06301 | -0,07265 | 0,12012 | 0,113977 |
| ***Ppp2r2a*** | 0,092768 | -0,01173 | -0,03884 | 0,074772 | 0,031201 |
| ***Prkd1*** | -0,00044 | 0,078895 | -0,14158 | 0,042999 | 0,164733 |
| ***Rxra*** | 0,193827 | 0,169862 | 0,015074 | 0,019906 | -0,27327 |
| ***Ncoa1*** | 0,119747 | 0,011103 | -0,03914 | 0,078061 | -0,04566 |
| ***Ppp2r1b*** | -0,21429 | 0,114855 | -0,01451 | 0,153858 | 0,050516 |
| ***Ugt2a1*** | -0,01249 | 0,005613 | 0,013367 | 0,025455 | 0,025249 |
| ***Ppp2cb*** | -0,17743 | 0,017874 | 0,059428 | 0,076909 | 0,074506 |
| ***Prkcb*** | -0,12369 | 0,164085 | -0,09661 | 0,022314 | 0,069768 |
| ***Chst2*** | -0,05745 | 0,132523 | -0,02665 | 0,049296 | -0,06265 |
| ***Hsp90ab1*** | 0,153701 | -0,42093 | 0,171052 | 0,082244 | 0,047582 |
| ***Mgst2*** | 0,091526 | -0,15884 | 0,018484 | -0,07328 | 0,127777 |
| ***Ppp2r1a*** | 0,042315 | 0,05266 | -0,10248 | 0,039151 | -0,02811 |
| ***Gm4846*** | -0,29505 | 0,226148 | -0,02433 | 0,018699 | 0,071374 |
| ***Ppp2ca*** | -0,03232 | 0,011038 | -0,00242 | 0,027061 | -0,0107 |
| ***Snw1*** | -0,03129 | 0,078455 | -0,02802 | 0,012924 | -0,07194 |
| ***Nrip1*** | 0,062801 | -0,10827 | 0,210467 | -0,17986 | -0,03364 |
| ***Map2k7*** | 0,19363 | -0,09578 | -0,15608 | 0,019885 | -0,01475 |
| ***Sult1c3*** | -0,34907 | 0,08808 | 0,041571 | -0,07432 | 0,237151 |
| ***Sult2b1*** | -0,01121 | 0,160563 | -0,1065 | -0,12214 | 0,008768 |
| ***Chst7*** | 0,044349 | 0,033325 | 0,080503 | -0,22675 | -0,02035 |
| ***Chst13*** | -0,00786 | -0,2197 | 0,066671 | 0,037978 | 0,02233 |
| ***Prkci*** | 0,024551 | -0,43889 | 0,186525 | 0,082533 | 0,04227 |
| ***Mgst1*** | 0,106844 | 0,117306 | -0,03641 | -0,20413 | -0,0961 |
| ***Ppp2r5d*** | 0,002409 | 0,042909 | -0,12701 | -0,10047 | 0,063347 |
| ***Mapk1*** | 0,044576 | -0,16036 | 0,093211 | 0,032593 | -0,13623 |
| ***Ppp2r2b*** | -0,03614 | 0,085198 | -0,06589 | -0,10135 | -0,01374 |
| ***Gstm3*** | -0,14577 | -0,12861 | 0,088728 | 0,337619 | -0,29394 |
| ***Ndst2*** | -0,20442 | -0,07856 | 0,042657 | 0,150329 | -0,05868 |
| ***Ppm1j*** | -0,06293 | 0,166445 | -0,12194 | -0,08866 | -0,07174 |
| ***Gsto2*** | -0,2979 | -0,13784 | -0,0402 | 0,048601 | 0,235744 |
| ***Chst10*** | -0,37018 | -0,01537 | -0,1227 | 0,059074 | 0,20577 |
| ***Chst15*** | -0,2913 | -0,03826 | 0,432391 | 0,094591 | -0,4521 |
| ***Ptpa*** | 0,113733 | -0,34736 | -0,21522 | 0,135324 | 0,051821 |
| ***Aldh18a1*** | 0,204397 | -0,38172 | -0,32766 | -0,0109 | 0,227284 |
| ***Hs3st3a1*** | -0,31821 | -0,09785 | -0,1328 | 0,081268 | 0,162928 |
| ***Ppp2r3a*** | -0,2392 | -0,06188 | 0,091781 | 0,069591 | -0,17016 |
| ***Aldh2*** | -0,05226 | 0,209599 | -0,10349 | -0,25722 | -0,14803 |
| ***Ppp2r5e*** | 0,130195 | 0,014167 | -0,20408 | -0,17612 | -0,11958 |
| ***Gal3st2*** | -0,28091 | -0,11758 | 0,0691 | -0,27645 | 0,248223 |
| ***Prkce*** | 0,064813 | -0,4592 | -0,03295 | 0,190098 | -0,12429 |
| ***Gstz1*** | -0,02332 | 0,812286 | -0,42883 | -0,45182 | -0,28936 |
| ***Chst1*** | -0,25252 | 0,036369 | 0,10231 | -0,25253 | -0,04909 |
| ***Aldh6a1*** | -0,12568 | 0,70767 | -0,35314 | -0,33851 | -0,32771 |
| ***Gstm6*** | -0,17145 | 0,278406 | -0,21578 | -0,06966 | -0,26243 |
| ***Gstm5*** | -0,13549 | -0,09147 | -0,00525 | -0,06958 | -0,14526 |
| ***Gsta3*** | 0,082941 | 0,207171 | -0,25599 | -0,34971 | -0,13187 |
| ***Aldh3b1*** | -0,26231 | 0,03008 | -0,35622 | 0,084234 | 0,031547 |
| ***Ppm1l*** | -0,13908 | -0,07156 | -0,04962 | -0,14165 | -0,08811 |
| ***Gsta5*** | -0,26097 | -0,83708 | 0,265944 | 0,378938 | -0,0793 |
| ***Gstk1*** | -0,18235 | 0,980101 | -0,47865 | -0,43221 | -0,41973 |
| ***Gstm4*** | -0,10794 | -0,03032 | 0,081005 | -0,06848 | -0,40839 |
| ***Aldh5a1*** | -0,01626 | 0,086619 | -0,35096 | -0,1413 | -0,15137 |
| ***Hs2st1*** | -0,17552 | -0,37367 | -0,01396 | -0,06005 | 0,047499 |
| ***Ndst1*** | -0,12359 | -0,08368 | -0,05185 | -0,22874 | -0,16588 |
| ***Ugt2b10*** | -0,06221 | -0,04937 | 0,124047 | -0,33365 | -0,35 |
| ***Ndst4*** | -0,46189 | -0,18419 | -0,0894 | 0,068262 | -0,03833 |
| ***Med1*** | 0,053763 | -0,41944 | -0,10408 | -0,04778 | -0,19516 |
| ***Cyp3a7*** | -0,36401 | -0,43583 | -0,05304 | 0,112209 | 0,001546 |
| ***Sult4a1*** | -0,03304 | 0,183221 | -0,20444 | -0,4542 | -0,2331 |
| ***Hsp90b1*** | -0,02465 | -0,28963 | -0,1934 | -0,04917 | -0,19621 |
| ***Aldh1a3*** | -0,15936 | -0,26665 | -0,27743 | -0,07322 | 0,015636 |
| ***Mapk3*** | -0,12178 | -0,2083 | -0,11816 | -0,24199 | -0,08334 |
| ***Fmo6*** | -0,23521 | -0,20859 | -0,15424 | -0,06636 | -0,12892 |
| ***Cyp2c9*** | -0,67462 | 0,257784 | 0,167694 | -0,2441 | -0,31643 |
| ***Prkcz*** | 0,069884 | -0,36703 | -0,18978 | -0,14472 | -0,18557 |
| ***Gstm3*** | -0,08747 | -0,54621 | -0,07372 | 0,03858 | -0,15077 |
| ***Aldh9a1*** | -0,30241 | 0,092659 | -0,28733 | -0,27964 | -0,1646 |
| ***Dnajc7*** | -0,04284 | -0,5412 | 0,021931 | -0,29237 | -0,12199 |
| ***Map2k6*** | -0,49451 | 0,296523 | -0,02225 | -0,42464 | -0,38477 |
| ***Ppp2r5a*** | -0,05414 | -0,4049 | -0,15871 | -0,16977 | -0,24948 |
| ***Map2k5*** | 0,011184 | -0,51421 | -0,24865 | -0,09975 | -0,20663 |
| ***Gsto1*** | -0,17536 | -0,37914 | -0,10067 | -0,17171 | -0,25074 |
| ***Gstm2*** | -0,39018 | 0,482035 | -0,48423 | -0,34486 | -0,40086 |
| ***Abcc3*** | -0,1613 | -0,49806 | -0,1044 | -0,23767 | -0,1381 |
| ***Abcc2*** | 0,030509 | -0,50224 | -0,16845 | -0,25012 | -0,25719 |
| ***Aldh1a1*** | 0,149176 | -0,68979 | -0,46523 | -0,18232 | 0,010048 |
| ***Aldh1b1*** | -0,4054 | -0,14953 | -0,38866 | -0,21044 | -0,2086 |
| ***Sult1e1*** | -0,77274 | 0,088906 | 0,193589 | -0,32624 | -0,62783 |
| ***Gstt2/gstt2b*** | -0,15546 | -0,17558 | -0,17329 | -0,54045 | -0,45832 |
| ***Gstm1*** | -0,39472 | -0,33612 | -0,22027 | -0,25434 | -0,4292 |
| ***Aldh3a2*** | -0,07275 | -0,76701 | -0,39468 | -0,41599 | -0,25463 |
| ***Nr1i3*** | -0,76069 | 0,127178 | -0,24139 | -0,64651 | -0,60801 |
| ***Aldh8a1*** | -0,129 | 0,635762 | -0,37848 | -0,93457 | -1,40599 |
| ***Sult1d1*** | -0,75893 | -0,67835 | -0,02509 | -0,09733 | -0,65697 |
| ***Prkd3*** | -0,08954 | -0,27329 | -0,74934 | -0,84385 | -0,69256 |
| ***Ppargc1a*** | -0,79412 | -0,38501 | -0,49487 | -0,42794 | -0,66493 |
| ***Cyp2b19*** | -0,74333 | -0,60669 | -0,53875 | -0,34149 | -0,67054 |
| ***Prkca*** | -0,78907 | -1,45442 | -0,55646 | -0,30357 | -0,30056 |
| ***Mgst3*** | -0,9771 | -0,44419 | -1,12997 | -0,54633 | -0,3664 |
| ***Fmo1*** | -0,63676 | -0,10164 | -0,6546 | -1,21774 | -0,92992 |
| ***Fmo2*** | -1,09771 | -0,29407 | -0,97409 | -0,98823 | -0,54841 |
| ***Sult1c2*** | -1,32587 | -0,66 | -1,16059 | -0,84454 | -0,46645 |
| ***Fmo4*** | -1,14351 | -1,04934 | -0,89112 | -0,78921 | -0,65782 |
| ***Cyp2b23*** | -1,71292 | -0,97791 | -0,64935 | -0,72848 | -0,86702 |
| ***Abcb1*** | -3,15988 | -0,99045 | -1,36827 | -1,08103 | -0,80522 |
| ***Sult2a1*** | -3,65458 | -0,33344 | -1,47038 | -1,9286 | -2,14337 |
| ***Cyp2b6*** | -4,87545 | -1,58102 | -1,3552 | -1,03239 | -1,02875 |
| ***Cyp3a5*** | -4,0533 | -3,72033 | -2,12901 | 0,626905 | -0,99918 |
| ***Cyp2c8*** | -3,0275 | -2,40431 | -1,64767 | -1,56727 | -2,10398 |
| ***Sult3a1/sult3a2*** | -7,11037 | -3,91233 | -0,98212 | -0,60393 | -0,52936 |
| ***Cyp2b13/cyp2b9*** | -7,26718 | -4,61594 | -3,54464 | -3,04065 | -2,84884 |
| ***Fmo3*** | -5,79658 | -3,84383 | -4,39118 | -4,23965 | -3,20229 |

**Table S13:** Activation Z-score of genes involved in fatty acid metabolism male vs. female. Activation Z-score was calculated with IPA software from Qiagen.

| **Pathway Summary** | -3,602 | -0,087 | -2,214 | -2,961 | -3,154 |
| --- | --- | --- | --- | --- | --- |
| **genes in the fatty acid metabolism network** | **0 h** | **24 h** | **48 h** | **72 h** | **96 h** |
| ***Cyp4a22*** | 7,503465 | 6,92967 | 5,352887 | 3,397882 | 1,755223 |
| ***Elovl3*** | 5,046876 | 6,186568 | 2,899638 | 1,088746 | 0,664992 |
| ***Slco1a1*** | 3,103735 | 4,931852 | 3,018541 | 2,176901 | 1,075615 |
| ***Saa1*** | -0,22883 | 0,807029 | 1,679516 | 1,794978 | 0,628602 |
| ***Scp2*** | 0,905224 | 2,031537 | 0,985545 | 0,350656 | 0,253287 |
| ***Egr1*** | 0,200368 | 0,967559 | 1,098924 | 1,112761 | 1,004121 |
| ***F2r*** | 1,338302 | 0,862247 | 0,770082 | 0,465858 | 0,599782 |
| ***Gdf15*** | 0,203624 | 1,295703 | 0,569867 | 0,822218 | 1,120081 |
| ***Slc10a2*** | 0,620419 | 0,93608 | 0,591403 | 0,981331 | 0,854708 |
| ***Cyp2e1*** | 0,155034 | 0,940182 | 2,273941 | 0,984777 | -0,50221 |
| ***Hmox1*** | 0,452339 | 0,564182 | 1,329974 | 0,634012 | 0,537807 |
| ***Fabp4*** | -0,12234 | 1,045039 | 1,446707 | 0,929734 | 0,149975 |
| ***Pkm*** | -0,08209 | 0,184047 | 0,898776 | 1,488772 | 0,741155 |
| ***Gpc1*** | 0,663466 | 0,725001 | 0,593157 | 0,60759 | 0,525036 |
| ***Apobec1*** | 1,068508 | 0,595942 | 0,522453 | 0,411619 | 0,364742 |
| ***Fabp1*** | 0,377904 | 2,480121 | 1,289524 | -0,39195 | -0,87061 |
| ***Egfr*** | 1,511161 | 0,917405 | 0,196501 | 0,031613 | 0,151305 |
| ***Areg*** | -0,16675 | 1,281483 | 0,731572 | 0,577384 | 0,288823 |
| ***Vegfa*** | -0,06545 | 0,80094 | 0,960118 | 0,861856 | 0,106438 |
| ***Slco3a1*** | 0,218247 | 0,035296 | 0,407058 | 0,749166 | 1,112706 |
| ***Cxcl2*** | 0,96606 | 0,145288 | 0,10557 | 0,506642 | 0,672174 |
| ***Nudt7*** | 2,278704 | 1,573846 | -0,43075 | -0,47224 | -0,68295 |
| ***Ednrb*** | 0,011695 | 1,038317 | 0,813442 | 0,432524 | -0,0453 |
| ***Cav1*** | 0,773773 | 0,658437 | 0,150822 | 0,097915 | 0,546004 |
| ***Msn*** | -0,30299 | -0,12472 | 0,895414 | 0,692852 | 0,991822 |
| ***Cebpb*** | 0,197873 | 0,933786 | 0,337535 | 0,344411 | 0,258395 |
| ***B3galt1*** | 0,823736 | 1,057828 | 0,130182 | 0,008273 | -0,03934 |
| ***Arsa*** | 1,038985 | 0,228933 | 0,338002 | 0,204897 | 0,008078 |
| ***Hcar2*** | 0,07692 | 1,692572 | 0,145806 | -0,05036 | -0,05117 |
| ***Atp11a*** | 0,228036 | 0,308245 | 0,417813 | 0,410396 | 0,403075 |
| ***Abcc1*** | -0,32994 | 0,570196 | 0,756808 | 0,448079 | 0,320496 |
| ***Ptges*** | -0,40226 | 0,469452 | 1,145467 | 0,366267 | 0,178699 |
| ***St8sia3*** | 0,202583 | 0,451822 | 0,988341 | 0,357582 | -0,2478 |
| ***Igf1*** | -0,0711 | 0,534636 | 0,478904 | 0,342446 | 0,463394 |
| ***Socs3*** | 0,648692 | 0,44729 | 0,310238 | 0,230759 | 0,070863 |
| ***Tac1*** | 0,27023 | 0,636432 | 0,47235 | 0,056696 | 0,253429 |
| ***Dbp*** | 0,528163 | 1,06269 | 0,71036 | -0,09931 | -0,5649 |
| ***Cyp1a2*** | 0,066898 | 1,509168 | 1,215758 | 0,619908 | -1,80321 |
| ***Ccn1*** | 0,813415 | -0,28874 | 0,297317 | 0,371764 | 0,414685 |
| ***Tlr4*** | -0,14703 | -0,00808 | 0,78129 | 0,494066 | 0,451944 |
| ***Abcg2*** | 1,022892 | -0,17177 | -0,15845 | 0,248904 | 0,622251 |
| ***Kitlg*** | -0,02606 | 1,06252 | 0,234838 | 0,176335 | 0,112236 |
| ***Il22*** | 0,257143 | 0,221181 | 0,380083 | 0,32858 | 0,361546 |
| ***Prkab2*** | -0,04086 | 0,604595 | 0,730222 | 0,366204 | -0,11625 |
| ***Fabp2*** | 0,430475 | 1,37847 | -0,22077 | -0,13115 | 0,084636 |
| ***Bdnf*** | 0,244529 | 0,182207 | 0,014395 | 0,402227 | 0,692226 |
| ***Mitf*** | 0,478192 | -0,53408 | 0,256864 | 0,645143 | 0,677926 |
| ***Cyp2c23*** | 0,19853 | 1,306753 | 0,733897 | -0,19629 | -0,54483 |
| ***Eif4ebp1*** | 0,246025 | 0,279086 | 0,437429 | 0,286157 | 0,203797 |
| ***Kit*** | 0,288402 | 0,247651 | 0,559729 | 0,24703 | 0,108773 |
| ***Tgfbr2*** | 0,22758 | 0,159388 | 0,48169 | 0,38976 | 0,164781 |
| ***Apoa5*** | -0,00171 | 1,200094 | 0,038027 | 0,117437 | 0,054655 |
| ***S100a8*** | 0,316813 | 0,389164 | 0,164116 | 0,207476 | 0,302258 |
| ***Cyp27a1*** | 0,099837 | 0,68324 | 0,612828 | 0,138729 | -0,20747 |
| ***Mapkapk2*** | 0,422276 | 0,184214 | 0,140233 | 0,328419 | 0,195745 |
| ***Ppard*** | 0,035931 | 0,504734 | 0,246923 | 0,193196 | 0,287906 |
| ***F2rl1*** | 0,068704 | -0,13066 | 0,236344 | 0,442689 | 0,606844 |
| ***Sirt1*** | -0,0669 | 0,763695 | 0,318872 | 0,227024 | -0,0192 |
| ***Avp*** | 0,105017 | 0,100225 | 0,215471 | 0,348399 | 0,448057 |
| ***Ccl3l3*** | 0,240281 | 0,444464 | 0,384787 | 0,111675 | 0,025613 |
| ***Hsd17b8*** | 0,523038 | 0,646799 | 0,059485 | 0,102213 | -0,12863 |
| ***Mif*** | 0,042114 | 0,031338 | 0,387972 | 0,552037 | 0,186744 |
| ***Acsl1*** | 0,275928 | 1,416515 | 0,136382 | -0,25224 | -0,37723 |
| ***Pla2g5*** | 0,00819 | 0,536913 | 0,324362 | 0,118147 | 0,210228 |
| ***Acaa1*** | 0,400371 | 0,929744 | 0,067515 | -0,18491 | -0,03445 |
| ***Cotl1*** | 0,14868 | 0,128827 | 0,200416 | 0,450562 | 0,248541 |
| ***Abcg8*** | -0,39334 | 0,727529 | 0,795213 | 0,269695 | -0,23699 |
| ***Acsf2*** | 0,476563 | 0,584399 | 0,044442 | 0,035219 | 0,012726 |
| ***Runx1*** | -0,19519 | 0,095331 | 0,451294 | 0,511187 | 0,289887 |
| ***Anxa1*** | -0,06229 | -0,012 | 0,251502 | 0,404198 | 0,569906 |
| ***Lpl*** | 0,511842 | 0,147046 | 0,090463 | 0,161693 | 0,189924 |
| ***Btc*** | -0,11052 | 0,476393 | 0,300789 | 0,370847 | 0,063309 |
| ***Gpihbp1*** | 0,125463 | 0,410104 | 0,52057 | -0,14343 | 0,181107 |
| ***Ffar4*** | -0,31033 | 0,062398 | 0,941238 | 0,43388 | -0,07842 |
| ***C5*** | 0,293912 | 1,290041 | -0,01347 | -0,21699 | -0,30486 |
| ***Tgfa*** | 0,387056 | 0,210526 | 0,146732 | 0,097411 | 0,206395 |
| ***Lcat*** | -0,17959 | 0,579446 | 0,584129 | 0,271463 | -0,21531 |
| ***Ano6*** | 0,182379 | -0,01837 | 0,248597 | 0,39548 | 0,231441 |
| ***Ephx2*** | 0,424815 | 0,609694 | -0,06428 | 0,03513 | 0,023852 |
| ***Star*** | -0,06788 | 0,067333 | 0,275318 | 0,342523 | 0,407752 |
| ***Thrsp*** | -0,07521 | 1,394794 | 0,232433 | -0,05143 | -0,476 |
| ***Hacd4*** | 0,159192 | 0,10177 | 0,42503 | 0,169659 | 0,164038 |
| ***Chka*** | 0,426351 | 0,16697 | -0,22775 | 0,077874 | 0,541396 |
| ***Map2k1*** | 0,13442 | 0,207022 | 0,290068 | 0,177577 | 0,173668 |
| ***Pam*** | -0,07714 | -0,59335 | -0,05769 | 0,912832 | 0,784352 |
| ***Ins1*** | -0,15546 | 0,311846 | 0,152701 | 0,398085 | 0,245218 |
| ***Alox5*** | 0,097119 | 0,514201 | -0,04708 | 0,266093 | 0,107334 |
| ***Reln*** | -0,11612 | 0,046375 | 0,407064 | 0,275971 | 0,323788 |
| ***Ltf*** | 0,176336 | 0,19017 | 0,361557 | 0,134003 | 0,049525 |
| ***Ptgis*** | -5E-06 | 0,265702 | 0,309336 | 0,12827 | 0,199699 |
| ***Lrat*** | -0,06049 | 0,208794 | 0,084343 | 0,359785 | 0,303068 |
| ***Oxt*** | 0,135888 | 0,12567 | 0,109471 | 0,174866 | 0,337703 |
| ***Il17a*** | -0,14899 | 0,064948 | 0,589141 | 0,131666 | 0,233734 |
| ***Esr2*** | -0,00057 | 0,018629 | 0,301336 | 0,113546 | 0,433366 |
| ***Slc13a5*** | -0,21528 | 0,850017 | 0,396288 | -0,07046 | -0,09864 |
| ***Mfsd2b*** | 0,00675 | 0,169819 | 0,069104 | 0,278894 | 0,328563 |
| ***Ednra*** | 0,005759 | 0,234124 | 0,26893 | -0,09709 | 0,414275 |
| ***Cyp2s1*** | -0,07623 | -0,17694 | 0,083406 | 0,723848 | 0,266478 |
| ***Calb1*** | 0,322164 | 0,059735 | 0,135254 | -0,02045 | 0,317934 |
| ***Npc1l1*** | 0,22953 | 0,339241 | 0,066838 | 0,126331 | 0,037662 |
| ***Bmp2*** | 0,045494 | -0,21566 | 0,359039 | 0,444609 | 0,157484 |
| ***Spns2*** | -0,53938 | -0,1185 | 0,477213 | 0,765442 | 0,198112 |
| ***Cyp1a1*** | -0,0377 | 0,221035 | 0,38464 | 0,078008 | 0,130361 |
| ***F2*** | 0,060368 | 0,238874 | 0,224434 | 0,151489 | 0,097842 |
| ***Cd4*** | 0,426659 | 0,284947 | -0,12784 | -0,09186 | 0,280133 |
| ***B4galt1*** | 0,149573 | 0,309996 | 0,244133 | 0,099127 | -0,04937 |
| ***Abca4*** | -0,22516 | 0,274006 | 0,336817 | 0,065528 | 0,289708 |
| ***Sod1*** | -0,00286 | 0,235516 | 0,313475 | 0,189561 | -0,0042 |
| ***Il1b*** | 0,263769 | 0,310882 | -0,03707 | 0,045763 | 0,139904 |
| ***Kcnn4*** | 0,259664 | -0,08772 | 0,175254 | 0,194479 | 0,178763 |
| ***Prkcd*** | 0,074277 | 0,348892 | 0,246503 | 0,028518 | 0,008762 |
| ***Sphk1*** | 0,142443 | -0,11255 | 0,202839 | 0,136431 | 0,33292 |
| ***Nfkb1*** | 0,158929 | 0,198966 | -0,05669 | 0,144947 | 0,239545 |
| ***Pex5*** | 0,358129 | 0,184889 | 0,389115 | -0,02045 | -0,23866 |
| ***Lpin1*** | 0,304783 | 0,830156 | 0,490199 | -0,31185 | -0,64224 |
| ***Slc1a3*** | 0,092452 | 0,317137 | 0,003587 | 0,099027 | 0,15749 |
| ***Stx1a*** | 0,114638 | 0,434371 | 0,114831 | 0,064172 | -0,0609 |
| ***Sp1*** | 0,094922 | 0,310516 | 0,068808 | 0,047906 | 0,139302 |
| ***Rela*** | 0,172055 | 0,224581 | 0,095794 | 0,07538 | 0,090153 |
| ***Cntf*** | 0,152345 | 0,005822 | 0,321655 | 0,103383 | 0,070471 |
| ***Lrp10*** | 0,218111 | -0,32731 | 0,238422 | 0,311185 | 0,212797 |
| ***Hmgcl*** | -0,00448 | 0,219162 | 0,532861 | 0,084145 | -0,18872 |
| ***Fh*** | 0,289439 | 0,200237 | 0,097722 | 0,046696 | 0,005214 |
| ***Lipg*** | -0,59591 | 0,579194 | 0,290071 | 0,226069 | 0,131944 |
| ***Gnrh1*** | 0,198382 | -0,29813 | 0,317144 | 0,157381 | 0,256163 |
| ***Map2k3*** | 0,106438 | 0,031734 | 0,048844 | 0,2398 | 0,194726 |
| ***Fyn*** | 0,033787 | 0,57696 | 0,230998 | -0,11383 | -0,11226 |
| ***Sucla2*** | -0,07365 | 0,238709 | 0,103742 | 0,058305 | 0,280024 |
| ***Por*** | -0,22385 | 0,116112 | 0,419562 | 0,346398 | -0,05581 |
| ***Abca1*** | 0,121641 | 0,292213 | 0,159397 | 0,278491 | -0,26018 |
| ***Lamtor1*** | 0,079259 | -0,06169 | 0,228716 | 0,204765 | 0,136131 |
| ***Akr1c4*** | 0,140055 | 2,312705 | 0,392458 | -1,04147 | -1,21843 |
| ***Atp11b*** | 0,200468 | 0,064039 | 0,30509 | 0,085004 | -0,08501 |
| ***Pla2g10*** | -0,24089 | 0,31235 | 0,127281 | 0,119424 | 0,250417 |
| ***Lipc*** | -0,33561 | 0,056819 | 0,199002 | 0,359127 | 0,275048 |
| ***Vwf*** | -0,04866 | 0,054573 | 0,400477 | 0,043841 | 0,101266 |
| ***Sgpp1*** | 0,166081 | 0,115594 | 0,221875 | 0,071908 | -0,02587 |
| ***Mcat*** | 0,160198 | 0,294337 | -0,05166 | 0,122705 | 0,014659 |
| ***Hmox2*** | 0,173071 | 0,017732 | 0,196636 | 0,067259 | 0,077508 |
| ***Scd4*** | 0,10853 | -0,07341 | 0,032148 | 0,085914 | 0,375685 |
| ***Acadsb*** | 0,162206 | 0,336091 | -0,06282 | -0,0483 | 0,12505 |
| ***Snca*** | -0,42627 | 0,151173 | 0,30492 | 0,166235 | 0,313848 |
| ***Acbd6*** | 0,119008 | 0,093755 | 0,280415 | 0,085779 | -0,07518 |
| ***Tbxas1*** | -0,00737 | 0,224823 | 0,283397 | -0,15924 | 0,16072 |
| ***Itgav*** | 0,136453 | -0,41853 | 0,320676 | 0,1807 | 0,281092 |
| ***Irak1*** | 0,213248 | -0,22601 | 0,158575 | 0,292531 | 0,061371 |
| ***Socs1*** | -0,02489 | 0,159075 | 0,008826 | -0,02151 | 0,377607 |
| ***Gpat4*** | 0,440138 | 0,220668 | -0,1771 | 0,012453 | -0,00366 |
| ***Scap*** | -0,00978 | 0,275538 | 0,275419 | 0,015266 | -0,06669 |
| ***Ins*** | -0,33103 | 0,458564 | 0,005813 | -0,03666 | 0,391596 |
| ***Cd209d*** | 0,091387 | 0,313581 | 0,071771 | -0,02437 | 0,034695 |
| ***B3galt2*** | 0,497177 | 0,059779 | -0,05838 | -0,03372 | 0,019352 |
| ***Stard5*** | 0,083151 | 0,345222 | 0,468553 | -0,09486 | -0,31932 |
| ***Oxct2a/oxct2b*** | 0,116667 | -0,18203 | 0,104001 | 0,157922 | 0,285613 |
| ***Rxrb*** | 0,457257 | 0,219044 | 0,120287 | -0,14449 | -0,17246 |
| ***Prkar2b*** | -0,27859 | 0,108796 | 0,194686 | 0,282446 | 0,160946 |
| ***Slc51a*** | -0,06567 | 0,345798 | 0,206038 | -0,09768 | 0,074967 |
| ***Pla2g1b*** | 0,262145 | -0,0239 | -0,01338 | 0,010848 | 0,225401 |
| ***Angptl4*** | 0,581837 | 0,508041 | -0,05698 | -0,22451 | -0,35668 |
| ***Plaat1*** | -0,0946 | 0,479179 | 0,135682 | -0,06 | -0,01163 |
| ***Nppa*** | 0,034337 | 0,318987 | 0,116168 | -0,02375 | -0,01468 |
| ***Acox1*** | 0,201106 | 0,527651 | 0,197574 | -0,20825 | -0,29447 |
| ***Adcyap1*** | -0,1195 | 0,290491 | -0,08756 | 0,037486 | 0,296229 |
| ***Slc27a4*** | 0,152291 | -0,23916 | 0,166582 | 0,259891 | 0,071442 |
| ***Adipor1*** | 0,056755 | -0,02311 | 0,123791 | 0,204018 | 0,041937 |
| ***Acaa2*** | 0,088348 | 1,205352 | -0,2018 | -0,36845 | -0,3216 |
| ***Ada*** | -0,15487 | 0,116629 | 0,033557 | 0,081439 | 0,323756 |
| ***Foxo3*** | 0,150165 | 0,280371 | 0,170242 | -0,06662 | -0,14944 |
| ***Wnt1*** | 0,033599 | 0,388916 | 0,293452 | -0,19901 | -0,13614 |
| ***Tnxb*** | -0,01833 | 0,254863 | 0,134587 | -0,08781 | 0,08813 |
| ***Fcer1g*** | 0,466088 | -0,02297 | 0,085672 | -0,05944 | -0,11566 |
| ***Xbp1*** | 0,283165 | 0,006163 | 0,028796 | 0,000284 | 0,033335 |
| ***Nos2*** | 0,047766 | 0,196393 | 0,014087 | -0,16033 | 0,249916 |
| ***Map3k1*** | -0,22776 | 0,608811 | 0,12805 | -0,11516 | -0,05375 |
| ***Triap1*** | 0,156075 | 0,396196 | 0,141518 | -0,03061 | -0,32942 |
| ***Sspn*** | -0,39404 | -0,1089 | 0,475158 | 0,230308 | 0,12567 |
| ***Sgpl1*** | -0,17167 | 0,147311 | 0,181794 | -0,02678 | 0,196809 |
| ***Slc1a1*** | -0,25939 | 0,162354 | 0,024637 | 0,197187 | 0,201651 |
| ***Gpx4*** | 0,43353 | -0,0627 | -0,1227 | 0,010968 | 0,065181 |
| ***Clu*** | 0,169921 | 0,158111 | -0,1104 | -0,01523 | 0,119884 |
| ***Il16*** | -0,39255 | 0,136877 | 0,20861 | 0,112644 | 0,249318 |
| ***Hras*** | 0,40146 | -0,23578 | -0,11532 | 0,234695 | 0,023973 |
| ***Rgn*** | -0,25904 | 1,131845 | 0,357521 | -0,2515 | -0,68179 |
| ***Cyp4f11*** | -0,08725 | 0,372413 | 0,095964 | -0,08549 | -0,00129 |
| ***Cul3*** | 0,020882 | 0,207118 | 0,034457 | 0,08637 | -0,07733 |
| ***Ces2*** | 0,229821 | 0,155655 | 0,105746 | 0,065959 | -0,28689 |
| ***Slc27a2*** | 0,179649 | 1,385061 | -0,21278 | -0,39159 | -0,7137 |
| ***Suclg1*** | 0,261195 | 0,137212 | -0,00545 | -0,0765 | -0,07355 |
| ***Aacs*** | -0,2629 | 0,407739 | 0,053999 | 0,033047 | 0,006437 |
| ***Stat5b*** | -0,23896 | 0,314471 | 0,099072 | 0,045493 | 0,011286 |
| ***A3galt2*** | -0,23166 | 0,394605 | 0,134691 | 0,009511 | -0,07663 |
| ***Abca12*** | -0,1599 | 0,141797 | 0,32541 | -0,11128 | 0,029805 |
| ***Slc9a3r2*** | 0,275201 | 0,14752 | -0,19208 | -0,07088 | 0,061981 |
| ***Ghrl*** | -0,25736 | 0,238318 | 0,189848 | 0,054328 | -0,0071 |
| ***Elovl4*** | 0,354012 | -0,08627 | 0,221409 | -0,17037 | -0,10198 |
| ***Klf11*** | 0,263954 | 0,461579 | 0,044926 | -0,37353 | -0,20067 |
| ***Prxl2b*** | 0,527311 | 0,104619 | -0,21713 | -0,00462 | -0,21587 |
| ***Ghr*** | 0,020345 | 0,862462 | -0,07016 | -0,35706 | -0,26179 |
| ***Sdhb*** | 0,088212 | 0,221074 | -0,02752 | -0,01215 | -0,07708 |
| ***Ldlr*** | -0,19533 | -0,23531 | 0,05352 | 0,181182 | 0,380566 |
| ***Fads2*** | -0,2342 | 0,280332 | 0,08521 | 0,085893 | -0,03826 |
| ***Arnt*** | -0,02327 | 0,187907 | -0,00335 | -0,04279 | 0,055936 |
| ***Slc27a5*** | -0,19069 | 0,503285 | 0,492936 | -0,04094 | -0,59322 |
| ***Phgdh*** | -0,37389 | -0,2253 | -0,11459 | 0,328638 | 0,556468 |
| ***Nfkbia*** | 0,010711 | 0,285474 | -0,04494 | -0,06765 | -0,0162 |
| ***Abat*** | -0,04001 | 0,729514 | -0,084 | -0,23929 | -0,20142 |
| ***Tnf*** | -0,18177 | -0,4173 | 0,296697 | 0,27893 | 0,183334 |
| ***Apoa2*** | -0,17361 | 0,299471 | 0,622124 | -0,05897 | -0,53274 |
| ***Stat5a*** | -0,24017 | 0,286443 | 0,032579 | -0,0361 | 0,110802 |
| ***Cd83*** | -0,12075 | 0,344129 | 0,193813 | -0,10301 | -0,16707 |
| ***Smpd2*** | 0,373988 | 0,183463 | -0,043 | -0,20745 | -0,16175 |
| ***Cerk*** | 0,37678 | -0,40871 | 0,24643 | -0,13216 | 0,053874 |
| ***Ncam1*** | -0,11443 | 0,012297 | 0,347844 | 0,266199 | -0,3835 |
| ***Acadvl*** | 0,04424 | 0,257297 | 0,054568 | -0,1052 | -0,12314 |
| ***Gba*** | -0,08949 | -0,30691 | 0,002287 | 0,281243 | 0,239962 |
| ***Hacd1*** | -0,08739 | -0,47534 | 0,207957 | 0,271649 | 0,208672 |
| ***Rxra*** | 0,193827 | 0,169862 | 0,015074 | 0,019906 | -0,27327 |
| ***Hacd3*** | -0,11534 | 0,128228 | 0,462576 | -0,04605 | -0,30471 |
| ***Scarb1*** | -0,17737 | 0,467088 | 0,007424 | 0,012039 | -0,19073 |
| ***Mdh1*** | -0,06641 | 0,609027 | -0,05464 | -0,16995 | -0,20111 |
| ***Fdx1*** | 0,246646 | 0,014324 | -0,06385 | -0,02432 | -0,06093 |
| ***Tmem30a*** | -0,04702 | 0,283345 | -0,00767 | -0,05335 | -0,06974 |
| ***Abcc6*** | -0,08474 | 0,294524 | 0,027542 | -0,08805 | -0,04459 |
| ***Slc25a13*** | -0,13467 | -0,22342 | 0,44586 | 0,19267 | -0,18293 |
| ***Rasgrp4*** | 0,067573 | 0,250997 | 0,103654 | -0,35031 | 0,025117 |
| ***Ptk2b*** | 0,292783 | -0,40693 | 0,011903 | 0,069851 | 0,123411 |
| ***Pla2g6*** | 0,554899 | -0,63084 | 0,04968 | -0,0147 | 0,130395 |
| ***Scarb2*** | 0,203062 | -0,10702 | -0,06929 | 0,062819 | -0,00247 |
| ***Tspo*** | 0,199444 | -0,10564 | -0,08693 | -0,0227 | 0,093259 |
| ***Acot8*** | 0,294234 | -0,20949 | 0,082232 | -0,05299 | -0,03962 |
| ***Ptges3*** | -0,09485 | -0,13093 | 0,394845 | 0,005238 | -0,10124 |
| ***Smpd1*** | 0,298504 | -0,25833 | -0,03771 | 0,003571 | 0,063222 |
| ***Txn*** | 0,059856 | -0,18666 | -0,03048 | 0,111522 | 0,10477 |
| ***Htt*** | -0,11077 | -0,14556 | 0,015363 | 0,065637 | 0,222904 |
| ***Acot7*** | 0,136012 | -0,45218 | -0,0005 | 0,096697 | 0,264942 |
| ***Map3k5*** | 0,220329 | -0,46408 | -0,06448 | 0,353496 | -0,00104 |
| ***Oprl1*** | -0,23733 | 0,35561 | -0,09612 | -0,05376 | 0,05949 |
| ***Fads1*** | -0,21349 | 0,219678 | 0,020275 | -0,02971 | -0,01415 |
| ***Akap13*** | 0,371115 | -0,58444 | 0,023724 | 0,158101 | 0,00517 |
| ***Msmo1*** | -0,14019 | -0,50058 | 0,219667 | 0,165779 | 0,220969 |
| ***Slc25a10*** | 0,11865 | 0,696297 | -0,35526 | -0,25848 | -0,2367 |
| ***Wdtc1*** | 0,195379 | 0,166197 | -0,05373 | -0,10326 | -0,25719 |
| ***Smad3*** | 0,136784 | 0,106913 | -0,0102 | -0,24429 | -0,04875 |
| ***Eif6*** | 0,027371 | -0,26231 | -0,00343 | -0,01225 | 0,190126 |
| ***Gcdh*** | 0,101655 | 0,665216 | -0,28731 | -0,28521 | -0,27466 |
| ***Camp*** | -0,3413 | 0,170226 | 0,074399 | 0,066094 | -0,05043 |
| ***Acot9*** | -0,201 | -0,479 | -0,03022 | 0,290388 | 0,33654 |
| ***Ptafr*** | -0,43369 | 0,309562 | 0,048657 | -0,13854 | 0,121557 |
| ***Prkci*** | 0,024551 | -0,43889 | 0,186525 | 0,082533 | 0,04227 |
| ***Slc6a11*** | -0,24472 | -0,0248 | -0,00414 | -0,11504 | 0,282429 |
| ***Arhgef11*** | 0,119541 | -0,26957 | -0,01881 | 0,049776 | 0,002864 |
| ***Srebf1*** | -0,75619 | 0,685746 | 0,064183 | -0,03825 | -0,08533 |
| ***Vps4b*** | -0,15861 | 0,143235 | -0,01297 | -0,07382 | -0,02915 |
| ***Pemt*** | -0,23697 | 0,989664 | 0,319489 | -0,40521 | -0,8003 |
| ***Pias1*** | -0,01999 | -0,31838 | 0,043718 | 0,17472 | -0,01429 |
| ***Cpt1b*** | 0,439677 | -0,44974 | 0,025189 | -0,08737 | -0,06385 |
| ***Angptl3*** | -0,01735 | 0,479563 | 0,012199 | 0,059137 | -0,67252 |
| ***Mapk14*** | 0,220987 | -0,26318 | 0,014724 | -0,02297 | -0,09842 |
| ***Eif4ebp2*** | 0,21679 | -0,38658 | -0,10803 | 0,188426 | -0,06668 |
| ***Apof*** | -0,10418 | 0,377569 | -0,25427 | -0,25009 | 0,07323 |
| ***Mecr*** | 0,338302 | -0,05856 | -0,26117 | -0,08225 | -0,11077 |
| ***Fn1*** | -0,17803 | 0,115056 | -0,16879 | -0,00665 | 0,063373 |
| ***Ces1*** | -0,12385 | 0,340526 | 0,219336 | 0,011072 | -0,6343 |
| ***Sdha*** | -0,00492 | 0,116038 | -0,02557 | -0,20757 | -0,0694 |
| ***Orai1*** | -0,09673 | -0,61993 | 0,181397 | 0,12278 | 0,214529 |
| ***Ttc39b*** | -0,21905 | 0,019599 | -0,13306 | 0,025097 | 0,10313 |
| ***Apoa1*** | 0,200219 | -0,07687 | -0,25979 | -0,08649 | 0,018263 |
| ***Them4*** | 0,330806 | -0,31639 | -0,05956 | 0,065823 | -0,22931 |
| ***Serpinc1*** | 0,000837 | 0,419675 | 0,144461 | -0,24383 | -0,53269 |
| ***Pla2g4f*** | -0,328 | -0,04694 | 0,123387 | -0,09503 | 0,132622 |
| ***Hbp1*** | 0,182819 | 0,246372 | -0,08863 | -0,37777 | -0,18233 |
| ***Hnf1a*** | 0,141772 | -0,46934 | 0,133149 | 0,073791 | -0,10086 |
| ***Atp8b1*** | -0,01227 | -0,40007 | -0,09746 | 0,211675 | 0,070155 |
| ***Rac1*** | 0,004156 | -0,36322 | 0,012891 | 0,016666 | 0,099646 |
| ***Ptger2*** | -0,09062 | -0,20236 | -0,19651 | 0,448995 | -0,19034 |
| ***Hpgds*** | -0,43071 | 0,138671 | 0,02627 | 0,073397 | -0,04638 |
| ***Hspa8*** | -0,01242 | -0,20995 | -0,02698 | -0,01897 | 0,028543 |
| ***Pdhb*** | 0,06376 | 0,009939 | 0,002782 | -0,12147 | -0,19501 |
| ***Nfe2l2*** | 0,237238 | 0,092282 | -0,35333 | -0,29459 | 0,071303 |
| ***Aqp8*** | -0,71873 | 0,170539 | 0,222727 | 0,155749 | -0,08264 |
| ***Plscr1*** | 0,120276 | -0,77387 | -0,3144 | 0,258643 | 0,453488 |
| ***Ager*** | -0,37751 | 0,010039 | -0,07772 | -0,07408 | 0,26053 |
| ***Pccb*** | -0,01837 | 0,691332 | -0,21995 | -0,44752 | -0,27311 |
| ***Ogdh*** | 0,242025 | -0,325 | -0,17389 | -0,02301 | 0,010489 |
| ***Dbi*** | 0,109036 | 0,036403 | -0,24376 | -0,13536 | -0,03999 |
| ***Pex2*** | 0,068347 | -0,15174 | 0,080045 | -0,24601 | -0,03156 |
| ***Rhoa*** | -0,13676 | -0,1495 | 0,013621 | -0,03237 | 0,023586 |
| ***Slc25a17*** | 0,163416 | -0,30211 | -0,15694 | -0,05449 | 0,064203 |
| ***Ppt2*** | 0,170545 | -0,58907 | -0,04 | 0,130633 | 0,022986 |
| ***Cs*** | -0,10216 | -0,26461 | -0,08707 | 0,118318 | 0,028027 |
| ***Alkbh7*** | -0,24204 | -0,14199 | -0,09804 | 0,188924 | -0,01551 |
| ***Sptlc2*** | -0,31244 | -0,16422 | -0,25597 | 0,165366 | 0,255729 |
| ***Scd2*** | -0,43642 | -0,0554 | -0,01182 | -0,0192 | 0,20793 |
| ***Ppara*** | 0,284157 | 0,016818 | -0,01524 | -0,10652 | -0,51136 |
| ***Cpt2*** | 0,166767 | -0,26646 | 0,024149 | -0,12539 | -0,15092 |
| ***Park7*** | 0,073974 | -0,24848 | -0,13259 | 0,041246 | -0,08749 |
| ***P2rx7*** | -0,39759 | -0,02834 | -0,0222 | 0,050312 | 0,038123 |
| ***Abca5*** | 0,090601 | -0,44144 | -0,15628 | -0,00613 | 0,151655 |
| ***Apoe*** | 0,00429 | 0,122289 | -0,03966 | -0,30261 | -0,15475 |
| ***Erlin2*** | -0,22985 | -0,51883 | 0,156051 | 0,027061 | 0,189336 |
| ***Got2*** | -0,08117 | -0,21755 | -0,02099 | 0,011125 | -0,07308 |
| ***Pla2g15*** | -0,18275 | -0,48308 | 0,281231 | -0,05829 | 0,056042 |
| ***Acsm1*** | -0,06478 | 1,698055 | 0,122459 | -1,0631 | -1,07973 |
| ***Mapk10*** | -0,29999 | 0,071825 | -0,06007 | -0,18061 | 0,078565 |
| ***Lss*** | -0,37367 | -0,13491 | 0,009673 | -0,05388 | 0,161206 |
| ***Me1*** | -0,27869 | 0,030381 | -0,09373 | -0,02535 | -0,02935 |
| ***B4galnt1*** | -0,22002 | -0,27144 | -0,01329 | 0,109422 | -0,00439 |
| ***Canx*** | 0,013316 | -0,27514 | -0,10962 | 0,000647 | -0,02993 |
| ***Mbtps1*** | 0,062942 | 0,065451 | -0,41234 | -0,03739 | -0,08008 |
| ***Cd9*** | -0,24868 | 0,058871 | -0,30968 | 0,046527 | 0,047127 |
| ***Large1*** | 0,509803 | -1,06261 | -0,15702 | 0,061787 | 0,240405 |
| ***Serpina6*** | -0,73851 | 0,531935 | 0,070508 | -0,01966 | -0,2586 |
| ***Suclg2*** | -0,01446 | -0,01453 | 0,091296 | -0,31125 | -0,16605 |
| ***Elovl5*** | -0,2241 | 0,445751 | -0,17497 | -0,27147 | -0,19038 |
| ***Pnpla8*** | -0,02232 | -0,0185 | 0,08681 | -0,16123 | -0,30148 |
| ***Sgms2*** | -0,12935 | -0,39279 | -0,04223 | 0,094152 | 0,030405 |
| ***Decr2*** | -0,17105 | 0,40993 | -0,12647 | -0,32775 | -0,22645 |
| ***Slc27a1*** | -0,41268 | 0,148235 | 0,088034 | -0,06771 | -0,20017 |
| ***Slc6a13*** | -0,13277 | 0,569141 | -0,40062 | -0,28111 | -0,20817 |
| ***Naaa*** | -0,04221 | -0,2246 | -0,09696 | 0,172792 | -0,2631 |
| ***Acot5*** | 0,028035 | -0,31003 | -0,05441 | -0,06459 | -0,06322 |
| ***Acss2*** | -0,47749 | 0,754739 | -0,27084 | -0,25899 | -0,21873 |
| ***Me2*** | 0,17193 | 0,088743 | 0,326317 | -0,41003 | -0,64892 |
| ***Acsm3*** | 0,104651 | 1,190503 | -0,61333 | -0,63344 | -0,52236 |
| ***Sphk2*** | 0,053698 | -0,31016 | -0,07302 | -0,01502 | -0,13099 |
| ***Psap*** | -0,19939 | 0,011129 | -0,06714 | -0,18966 | -0,03282 |
| ***Pitpna*** | -0,04177 | -0,55117 | 0,005303 | 0,079987 | 0,023119 |
| ***Ptch1*** | 0,262288 | -0,12041 | 0,031938 | -0,26919 | -0,39448 |
| ***Vtn*** | -0,07626 | 0,194188 | 0,082433 | -0,44226 | -0,25958 |
| ***Sptlc1*** | -0,01337 | -0,45987 | 0,132835 | -0,10487 | -0,07282 |
| ***Mknk1*** | 0,178907 | -0,27213 | -0,1018 | -0,19094 | -0,13346 |
| ***Ephx1*** | 0,253109 | -0,37561 | -0,06008 | -0,12743 | -0,2109 |
| ***Apoh*** | -0,13351 | 0,128208 | -0,03296 | -0,26651 | -0,22174 |
| ***Degs1*** | -0,12657 | -0,18738 | -0,01994 | -0,06146 | -0,13375 |
| ***Gstm4*** | -0,10794 | -0,03032 | 0,081005 | -0,06848 | -0,40839 |
| ***Eci2*** | 0,191046 | 0,091902 | -0,17084 | -0,39478 | -0,26362 |
| ***Slco2a1*** | 0,046009 | -1,01397 | -0,20624 | 0,30514 | 0,313243 |
| ***Prkaa2*** | 0,026958 | -0,16209 | -0,16135 | -0,10442 | -0,16972 |
| ***Aldh5a1*** | -0,01626 | 0,086619 | -0,35096 | -0,1413 | -0,15137 |
| ***Pitpnb*** | -0,08261 | -0,15436 | -0,09378 | -0,15169 | -0,0915 |
| ***Cyp4f16/cyp4f37*** | 0,273198 | -1,08066 | 0,204109 | 0,167198 | -0,15048 |
| ***Hif1a*** | -0,06375 | -0,27724 | -0,25817 | 0,067145 | -0,05719 |
| ***Slc36a1*** | -0,05822 | -0,34103 | -0,16696 | 0,073714 | -0,10515 |
| ***Ppcs*** | -0,06869 | -0,20115 | -0,10706 | -0,24008 | 0,015927 |
| ***Osbp*** | 0,216893 | -0,62377 | -0,11025 | -0,01803 | -0,07237 |
| ***Sc5d*** | -0,31835 | 0,126571 | -0,17349 | -0,20497 | -0,04536 |
| ***Hnf4a*** | 0,050151 | -0,0984 | -0,05882 | -0,18117 | -0,33054 |
| ***Hacd2*** | -0,20426 | -0,25938 | -0,05941 | -0,02494 | -0,08535 |
| ***Cyp2c18*** | -0,2453 | 0,259722 | 0,049139 | -0,39986 | -0,29794 |
| ***Acbd5*** | 0,036182 | 0,094417 | -0,17579 | -0,28295 | -0,30968 |
| ***Fadd*** | 0,106753 | -0,48414 | -0,24516 | -0,03754 | 0,013868 |
| ***Hrh1*** | -0,34244 | -0,13853 | 0,104488 | -0,22612 | -0,04703 |
| ***Lpgat1*** | -0,05591 | -0,44222 | -0,20386 | -0,01972 | 0,067357 |
| ***Ankrd23*** | -0,23414 | -0,62813 | -0,09998 | 0,190177 | 0,112685 |
| ***Tnfrsf1a*** | -0,28457 | -0,31858 | 0,02452 | 0,128835 | -0,232 |
| ***Cyp4f3*** | -0,09517 | 0,077374 | -0,09012 | -0,36049 | -0,21465 |
| ***Acly*** | -0,53178 | -0,15754 | -0,04428 | 0,10239 | -0,05604 |
| ***Acsl4*** | 0,022254 | -0,66087 | -0,26081 | -0,00454 | 0,213346 |
| ***Lbp*** | -0,33897 | -0,19207 | -0,26068 | -0,01096 | 0,109726 |
| ***Aqp9*** | -0,00116 | 0,275407 | -0,33264 | -0,34037 | -0,29478 |
| ***Stard3*** | -0,19645 | -0,28329 | -0,0961 | -0,05702 | -0,06698 |
| ***Plekha8*** | -0,18055 | -0,1673 | -0,20704 | 0,063617 | -0,20944 |
| ***Ankrd26*** | -0,11365 | -0,35643 | -0,12938 | -0,00549 | -0,10092 |
| ***Abcc10*** | 0,146101 | -0,29311 | -0,29604 | -0,09186 | -0,17382 |
| ***Dlat*** | -0,3087 | -0,2988 | -0,04747 | -0,01842 | -0,04631 |
| ***Tjp2*** | -0,26705 | -0,39514 | -0,14166 | -0,07741 | 0,158415 |
| ***Cyp2j2*** | 0,072261 | 0,23827 | -0,40419 | -0,37816 | -0,2528 |
| ***Myd88*** | 0,190685 | -0,79805 | -0,19427 | 0,007765 | 0,069196 |
| ***Awat1*** | 0,015113 | -0,12656 | -0,21645 | -0,46849 | 0,065396 |
| ***Jak2*** | -0,02781 | -0,30249 | -0,25278 | 0,041496 | -0,19887 |
| ***Atp11c*** | 0,121907 | -0,51146 | -0,07754 | 0,001083 | -0,28015 |
| ***Plg*** | -0,03442 | 0,501651 | -0,11731 | -0,58013 | -0,52523 |
| ***Slc16a1*** | -0,4491 | 0,02104 | -0,20466 | -0,24386 | 0,115867 |
| ***Scd*** | -0,13432 | 0,289914 | 0,097704 | -0,38032 | -0,63388 |
| ***Cyp4f8*** | -0,05355 | 1,32477 | -0,16482 | -1,06064 | -0,8168 |
| ***Mapk3*** | -0,12178 | -0,2083 | -0,11816 | -0,24199 | -0,08334 |
| ***Gsn*** | -0,10068 | -0,48996 | -0,15483 | -0,00717 | -0,02146 |
| ***Pank2*** | -0,15873 | -0,06449 | -0,2655 | -0,14184 | -0,14488 |
| ***Crat*** | -0,13528 | -0,3682 | -0,04971 | -0,08027 | -0,14379 |
| ***Arv1*** | -0,01046 | -0,2308 | -0,34189 | -0,03724 | -0,161 |
| ***Elovl1*** | -0,08439 | -0,74338 | -0,09103 | 0,023073 | 0,096609 |
| ***Erlin1*** | 0,064623 | -0,30683 | -0,07959 | -0,29632 | -0,18502 |
| ***Cyp2c9*** | -0,67462 | 0,257784 | 0,167694 | -0,2441 | -0,31643 |
| ***Gal3st1*** | 0,031791 | 0,169543 | -0,41082 | -0,27603 | -0,33125 |
| ***Acot13*** | -0,20654 | -0,48348 | -0,03027 | -0,11397 | 0,00707 |
| ***Decr1*** | 0,10697 | 0,084374 | -0,32973 | -0,45541 | -0,24776 |
| ***Ccl2*** | 0,066708 | -1,28911 | -0,39182 | 0,091752 | 0,678362 |
| ***Npc2*** | -0,37777 | -0,36177 | -0,13016 | -0,07041 | 0,090643 |
| ***Stim1*** | 0,180011 | -0,43319 | -0,24302 | -0,24569 | -0,11133 |
| ***Tnfaip8l3*** | -0,36713 | -0,02865 | -0,13969 | -0,19453 | -0,13112 |
| ***Neu3*** | -0,24807 | -0,10517 | -0,3995 | -0,16853 | 0,059742 |
| ***Gm2a*** | -0,19122 | -0,08217 | -0,38998 | -0,13446 | -0,06716 |
| ***Slc22a5*** | 0,026135 | -0,21294 | 0,023082 | -0,34764 | -0,36809 |
| ***Cd82*** | -0,26025 | -0,49219 | -0,00367 | -0,10458 | -0,02133 |
| ***Pctp*** | -0,4482 | -0,24054 | 0,059516 | -0,06193 | -0,19305 |
| ***Mapk9*** | -0,00623 | -0,34585 | -0,12821 | -0,25198 | -0,15795 |
| ***Faah*** | 0,061569 | 0,020827 | -0,45332 | -0,27998 | -0,24073 |
| ***Pdha1*** | -0,04335 | -0,37375 | -0,31533 | -0,27336 | 0,103624 |
| ***Nr1h3*** | 0,045567 | -0,18155 | -0,28072 | -0,24717 | -0,25165 |
| ***Plin2*** | 0,099249 | -0,88332 | -0,13492 | -0,13418 | 0,130005 |
| ***Slc45a3*** | -0,26267 | 0,091107 | -0,03347 | -0,27401 | -0,44452 |
| ***Acadl*** | -0,20704 | -0,55124 | -0,02583 | -0,07497 | -0,06891 |
| ***Acad9*** | -0,39186 | -0,26258 | -0,23954 | -0,03637 | -0,01402 |
| ***Pdk4*** | -0,7756 | 0,003353 | 0,254408 | -0,1653 | -0,2616 |
| ***Crot*** | 0,047541 | 0,472301 | -0,58118 | -0,50706 | -0,38848 |
| ***Adipor2*** | -0,11821 | 0,070816 | -0,11016 | -0,35886 | -0,44666 |
| ***Ceacam1*** | -0,1119 | 0,280183 | -0,65997 | -0,21636 | -0,25564 |
| ***Mttp*** | -0,05097 | -0,37223 | -0,1664 | -0,00399 | -0,37249 |
| ***Acot12*** | -0,1094 | -0,24134 | 0,067688 | -0,28036 | -0,4328 |
| ***Mtor*** | 0,012855 | -0,15939 | -0,11537 | -0,44566 | -0,29787 |
| ***Tert*** | -0,56719 | 0,007105 | -0,06652 | -0,35385 | -0,03381 |
| ***Dab1*** | 0,021078 | 0,084041 | -0,29917 | -0,47563 | -0,36587 |
| ***Hsd17b4*** | 0,162203 | -0,53786 | -0,16633 | -0,25361 | -0,24445 |
| ***Lrp1*** | 0,060206 | -0,55842 | -0,2624 | -0,11997 | -0,17952 |
| ***Abcg5*** | -0,60954 | 0,480023 | 0,03509 | -0,40794 | -0,56029 |
| ***Grb14*** | 0,12025 | -0,74204 | -0,46291 | -0,0726 | 0,074658 |
| ***Oxsm*** | -0,11358 | -0,3831 | -0,20943 | -0,24405 | -0,13282 |
| ***Il33*** | -0,29263 | 0,166507 | -0,72088 | -0,42045 | 0,177044 |
| ***Erbb2*** | -0,10815 | -0,01312 | -0,46978 | -0,24009 | -0,26202 |
| ***Abcb11*** | -0,28694 | 0,895383 | -0,1812 | -0,56721 | -0,9809 |
| ***Fabp5*** | -0,71247 | 0,616043 | -0,73515 | -0,22654 | -0,06958 |
| ***Abcc3*** | -0,1613 | -0,49806 | -0,1044 | -0,23767 | -0,1381 |
| ***Asah2*** | -0,41224 | 0,042507 | -0,32461 | -0,27624 | -0,16936 |
| ***Slco1b3*** | 0,060062 | 1,853445 | -0,18141 | -1,3031 | -1,57247 |
| ***Abcc2*** | 0,030509 | -0,50224 | -0,16845 | -0,25012 | -0,25719 |
| ***Ptgr2*** | -0,16268 | -0,28895 | -0,13002 | -0,28744 | -0,29741 |
| ***Tecr*** | -0,36624 | 0,124547 | -0,49878 | -0,24254 | -0,2026 |
| ***Elovl7*** | -0,13624 | -0,75725 | -0,20544 | 0,083267 | -0,19549 |
| ***Anxa6*** | -0,23768 | -0,58634 | -0,27128 | -0,15766 | 0,020174 |
| ***Sigirr*** | -0,3199 | -0,15088 | -0,57206 | -0,1305 | -0,07765 |
| ***Abcd1*** | -0,49221 | 0,088577 | -0,0985 | -0,43144 | -0,32268 |
| ***Tlr2*** | -0,22506 | -0,32334 | -0,54692 | -0,18222 | 0,019467 |
| ***Tmem30b*** | -0,13327 | 0,09786 | -0,12863 | -0,32433 | -0,77471 |
| ***Klf5*** | 0,083686 | -1,62188 | -0,12032 | 0,342979 | 0,021565 |
| ***Pik3cb*** | -0,21367 | -0,8372 | -0,21036 | -0,02392 | -0,02048 |
| ***Elovl2*** | -0,1327 | 0,596818 | -0,41057 | -0,5967 | -0,77845 |
| ***Pcolce2*** | -0,01071 | 0,222022 | -0,40884 | -0,61582 | -0,51861 |
| ***Cert1*** | -0,42481 | -0,43365 | -0,14275 | -0,21424 | -0,1175 |
| ***Pld2*** | -0,14237 | -0,53318 | -0,49428 | -0,16364 | -0,00071 |
| ***Nudt19*** | -0,23136 | -0,28295 | -0,40921 | -0,29969 | -0,11247 |
| ***Npc1*** | -0,24579 | 0,014381 | -0,29323 | -0,412 | -0,40126 |
| ***Cpt1a*** | 0,141407 | -0,3976 | -0,44114 | -0,41383 | -0,24206 |
| ***Acsl5*** | -0,30028 | -0,43237 | -0,34635 | -0,18066 | -0,10034 |
| ***Pml*** | -0,15464 | -0,33835 | -0,49261 | -0,32207 | -0,08209 |
| ***Ehhadh*** | -0,21017 | -0,18064 | -0,0393 | -0,47618 | -0,4945 |
| ***Slc10a1*** | -0,28589 | 0,904126 | 0,417748 | -0,66668 | -1,77778 |
| ***Fgfr3*** | -0,23208 | -0,26151 | -0,22428 | -0,28027 | -0,42774 |
| ***Il1rn*** | -0,75085 | 0,012076 | -0,49055 | 0,042444 | -0,23981 |
| ***Ppt1*** | -0,28164 | -0,81679 | -0,18339 | -0,17066 | 0,002255 |
| ***Acat1*** | 0,022855 | 0,265225 | -0,44318 | -0,69614 | -0,61754 |
| ***Agt*** | -0,32361 | 0,085077 | -0,52515 | -0,41596 | -0,29566 |
| ***Glyat*** | 0,156617 | -0,50171 | -0,61619 | -0,35438 | -0,17099 |
| ***Foxa1*** | 0,558808 | -0,8413 | -0,40635 | -0,48428 | -0,32452 |
| ***Soat2*** | -0,2558 | -0,20424 | -0,22728 | -0,25872 | -0,55695 |
| ***Apoa4*** | -0,46224 | -0,92768 | -0,32289 | -0,02632 | 0,2323 |
| ***Il18*** | -0,15491 | 0,211891 | -0,75962 | -0,60076 | -0,21168 |
| ***Sgms1*** | -0,26215 | -0,65755 | -0,17451 | -0,26171 | -0,16705 |
| ***Cyp2j5*** | -0,12274 | 1,139584 | -0,98765 | -1,06818 | -0,49243 |
| ***Tp53*** | -0,03313 | -0,694 | -0,51593 | -0,13647 | -0,15904 |
| ***Pcyt1a*** | -0,15554 | -0,76428 | -0,24717 | -0,25354 | -0,17347 |
| ***Vamp7*** | -0,15539 | -0,49023 | -0,31997 | -0,30985 | -0,33078 |
| ***Il15*** | -0,59553 | -0,11774 | -0,45572 | -0,1838 | -0,26009 |
| ***Fasn*** | -0,78593 | -0,47232 | -0,08825 | 0,042331 | -0,31419 |
| ***Gpam*** | -0,22696 | -0,85832 | -0,13764 | -0,24223 | -0,15404 |
| ***Abcb4*** | 5,65E-05 | -0,36295 | -0,32981 | -0,44952 | -0,4935 |
| ***Kng1*** | -0,41652 | -0,33195 | -0,41554 | -0,32952 | -0,19744 |
| ***Akr1b1*** | 0,044134 | -0,50866 | -0,45581 | -0,40216 | -0,37285 |
| ***Gk*** | -0,33561 | 0,276574 | -0,57982 | -0,56334 | -0,51153 |
| ***Sntb1*** | -0,02769 | -0,71682 | -0,66776 | -0,24059 | -0,082 |
| ***Napepld*** | -0,19458 | -1,27637 | -0,57216 | 0,121144 | 0,160326 |
| ***Nceh1*** | 0,132946 | -1,31178 | -0,44293 | -0,22489 | 0,021838 |
| ***St3gal5*** | -0,26422 | -0,5203 | -0,27124 | -0,28675 | -0,48801 |
| ***Nr1h4*** | 0,255714 | -0,60739 | -0,50502 | -0,55488 | -0,43199 |
| ***S1pr2*** | -0,50301 | -0,74844 | -0,42161 | -0,18954 | 0,015191 |
| ***Cxcl12*** | -0,31424 | 0,331944 | -0,47419 | -0,75144 | -0,67271 |
| ***Tlr3*** | -0,50991 | -0,77709 | -0,64884 | -0,13957 | 0,162816 |
| ***Asah1*** | -0,38117 | -1,25264 | -0,40556 | -0,00066 | 0,101994 |
| ***Slc51b*** | -0,94048 | -0,25946 | -0,43251 | -0,19989 | -0,11032 |
| ***Hpgd*** | -0,56275 | -0,65989 | 0,03402 | -0,31819 | -0,44264 |
| ***Prkag2*** | -0,50905 | -0,84728 | -0,13855 | -0,19606 | -0,26978 |
| ***Cdcp1*** | -0,67129 | -0,45136 | -0,23891 | -0,21123 | -0,40572 |
| ***Apom*** | -0,33283 | -0,1507 | -0,93804 | -0,45161 | -0,17364 |
| ***Cyp4f12*** | -0,20052 | 1,162041 | -0,30998 | -1,52239 | -1,1952 |
| ***Ptgr1*** | -0,13695 | -0,48338 | -0,8686 | -0,44136 | -0,13926 |
| ***Pon1*** | -0,20672 | 0,160498 | 0,102142 | -0,76871 | -1,36881 |
| ***Fas*** | -0,26124 | -0,37775 | -0,62657 | -0,563 | -0,29446 |
| ***Ggt1*** | -0,0743 | -0,93226 | -0,37545 | -0,3906 | -0,39591 |
| ***Itga3*** | -0,23658 | -1,51486 | -0,41804 | -0,12034 | 0,087227 |
| ***Rarb*** | -0,53431 | -0,65308 | -0,60431 | -0,37019 | -0,04105 |
| ***Cadm1*** | -0,42542 | -0,78384 | -0,22827 | -0,38722 | -0,38498 |
| ***App*** | -0,64282 | -0,94085 | -0,48604 | -0,276 | -0,02858 |
| ***Mgll*** | -0,06135 | -0,14408 | -0,90209 | -0,639 | -0,63762 |
| ***Pltp*** | -0,54254 | -0,02021 | -0,53789 | -0,73388 | -0,55614 |
| ***Avpr1a*** | -0,95307 | 0,110293 | -0,10142 | -0,48861 | -1,0545 |
| ***Cyp2c54*** | -0,74654 | 0,881591 | -0,18301 | -0,88046 | -1,56803 |
| ***Acot1*** | 0,184901 | -0,6519 | -0,7775 | -0,66162 | -0,6257 |
| ***Vldlr*** | -1,65317 | -0,33405 | 0,599907 | -0,31061 | -0,84224 |
| ***Baat*** | -0,02758 | -0,14993 | -0,88211 | -0,74723 | -0,82395 |
| ***Lipa*** | -0,39884 | -1,07622 | -0,60296 | -0,43253 | -0,17624 |
| ***Rtn4*** | -1,37918 | -0,57005 | -0,21648 | -0,31862 | -0,21186 |
| ***Atp8a1*** | -0,39573 | -0,88063 | -0,56524 | -0,35161 | -0,55696 |
| ***Tnfsf10*** | -0,76842 | -0,42081 | -0,89224 | -0,39771 | -0,27281 |
| ***Ppargc1a*** | -0,79412 | -0,38501 | -0,49487 | -0,42794 | -0,66493 |
| ***Mfsd2a*** | -0,98722 | -0,83224 | -0,51057 | -0,13327 | -0,3093 |
| ***Pparg*** | 0,308282 | -1,26225 | -0,77772 | -0,62831 | -0,46886 |
| ***Pla1a*** | -0,50878 | -0,65272 | -0,89701 | -0,51342 | -0,26201 |
| ***Apcs*** | -0,71083 | -0,82579 | -1,01747 | -0,38636 | 0,102086 |
| ***Cyp2b19*** | -0,74333 | -0,60669 | -0,53875 | -0,34149 | -0,67054 |
| ***Ntrk2*** | -2,47005 | -0,14335 | -0,30147 | -0,01801 | -0,01306 |
| ***Pnpla3*** | -1,66471 | -0,09853 | -0,52087 | -0,51328 | -0,23482 |
| ***Cyp7a1*** | -0,10229 | 1,351961 | -0,28199 | -1,67746 | -2,37028 |
| ***Acaca*** | -0,81725 | -0,50812 | -0,39237 | -0,72828 | -0,71412 |
| ***Far1*** | -0,10446 | -1,62185 | -0,54734 | -0,69251 | -0,43154 |
| ***Prkca*** | -0,78907 | -1,45442 | -0,55646 | -0,30357 | -0,30056 |
| ***Mgst3*** | -0,9771 | -0,44419 | -1,12997 | -0,54633 | -0,3664 |
| ***Cyp4a11*** | -0,5732 | 0,642984 | -0,96937 | -1,57802 | -1,1825 |
| ***Nqo1*** | -1,16194 | -0,75202 | -0,53121 | -0,54596 | -0,73791 |
| ***Slco1a2*** | -1,25718 | -0,22591 | -0,69486 | -0,50164 | -1,06208 |
| ***Elovl6*** | -1,0008 | -1,48377 | -0,7571 | -0,17717 | -0,38 |
| ***Ptgds*** | -1,88192 | -0,85589 | -0,79971 | -0,40544 | -0,41432 |
| ***Acot4*** | -1,43112 | -1,54884 | -0,62867 | -0,50609 | -0,29482 |
| ***Hao2*** | -3,73433 | -0,786 | 0,012724 | 0,079001 | -0,16565 |
| ***Csf1*** | -0,26315 | -1,99638 | -1,44569 | -0,86568 | -0,10769 |
| ***Acot2*** | -0,6759 | -2,39627 | -1,1104 | -0,58939 | -0,27743 |
| ***Abcb1b*** | -2,13704 | -1,12408 | -0,84069 | -0,67715 | -0,42022 |
| ***Akr1c3*** | -0,27282 | -0,12655 | -2,54033 | -1,89342 | -0,62448 |
| ***Abcc4*** | -0,77985 | -1,81574 | -1,03435 | -0,8991 | -1,03666 |
| ***Lepr*** | -1,18575 | -0,92697 | -0,63828 | -1,08262 | -1,81091 |
| ***Acot1*** | -3,43591 | -0,78669 | -0,8108 | -0,81683 | -0,67339 |
| ***Acnat1/acnat2*** | -1,16445 | -2,28286 | -1,83788 | -0,89655 | -0,51546 |
| ***Cyp4a14*** | -0,75272 | -0,24857 | -1,13345 | -2,35025 | -2,68532 |
| ***Vnn1*** | -0,56203 | -1,95141 | -2,38867 | -1,60905 | -0,88811 |
| ***Abcb1*** | -3,15988 | -0,99045 | -1,36827 | -1,08103 | -0,80522 |
| ***Abcd2*** | -3,21303 | -0,4612 | -1,06522 | -1,32308 | -1,44821 |
| ***Cd36*** | -1,76494 | -1,54792 | -2,50848 | -1,88358 | -1,04423 |
| ***Slco1a4*** | -1,59891 | -1,37986 | -2,1885 | -2,29899 | -2,58894 |
| ***Cyp2c8*** | -3,0275 | -2,40431 | -1,64767 | -1,56727 | -2,10398 |

**Table S14:** Activation Z-score of genes involved in the metabolism of terpenoids male vs. female. Activation Z-score was calculated with IPA software from Qiagen.

| **Pathway Summary** | -3,593 | -0,195 | -2,368 | -2,647 | -2,461 |
| --- | --- | --- | --- | --- | --- |
| **Genes in the Metabolism of terpenoid network** | **0 h** | **24 h** | **48 h** | **72 h** | **96 h** |
| **Hsd3b4** | 5,399488 | 4,572805 | 1,809618 | 0,39681 | 0,154409 |
| ***Cyp7b1*** | 2,97897 | 3,393742 | 1,334546 | 0,90291 | 1,035637 |
| ***Atf3*** | 0,530338 | 1,134128 | 1,22024 | 1,094871 | 1,103943 |
| ***Serpina1*** | 1,141492 | 1,2407 | 1,497335 | 0,705409 | 0,48324 |
| ***Saa1*** | -0,22883 | 0,807029 | 1,679516 | 1,794978 | 0,628602 |
| ***Scp2*** | 0,905224 | 2,031537 | 0,985545 | 0,350656 | 0,253287 |
| ***Ugt2b17*** | 0,557028 | 2,213161 | 1,292475 | 0,591189 | -0,39135 |
| ***Slc10a2*** | 0,620419 | 0,93608 | 0,591403 | 0,981331 | 0,854708 |
| ***Cyp2e1*** | 0,155034 | 0,940182 | 2,273941 | 0,984777 | -0,50221 |
| ***Cxcl10*** | -0,03222 | 1,066905 | 0,903724 | 0,776905 | 0,529257 |
| ***Gpc1*** | 0,663466 | 0,725001 | 0,593157 | 0,60759 | 0,525036 |
| ***Cyp21a2*** | 0,032534 | 0,422623 | 0,500586 | 0,877801 | 1,143798 |
| ***Egfr*** | 1,511161 | 0,917405 | 0,196501 | 0,031613 | 0,151305 |
| ***Areg*** | -0,16675 | 1,281483 | 0,731572 | 0,577384 | 0,288823 |
| ***Cav1*** | 0,773773 | 0,658437 | 0,150822 | 0,097915 | 0,546004 |
| ***Cyp8b1*** | 0,538241 | 1,700563 | 1,224542 | -0,39323 | -1,03816 |
| ***Grem2*** | 0,396421 | 0,714194 | 0,50747 | 0,47375 | -0,14161 |
| ***Tiparp*** | -0,69317 | 0,82824 | 0,991947 | 0,726471 | 0,063382 |
| ***Spp1*** | 0,356681 | 0,816593 | -0,63844 | 0,132484 | 1,13767 |
| ***Igf1*** | -0,0711 | 0,534636 | 0,478904 | 0,342446 | 0,463394 |
| ***Adh4*** | 0,843657 | 1,099951 | -0,02072 | -0,12201 | -0,06158 |
| ***Bmp5*** | 0,17196 | 0,321528 | 0,446983 | 0,35513 | 0,404198 |
| ***Cyp1a2*** | 0,066898 | 1,509168 | 1,215758 | 0,619908 | -1,80321 |
| ***Bdnf*** | 0,244529 | 0,182207 | 0,014395 | 0,402227 | 0,692226 |
| ***Phyh*** | 0,283241 | 1,296553 | 0,151206 | -0,14818 | -0,18579 |
| ***Ugt2b28*** | 0,774388 | 0,659801 | 0,273055 | -0,19352 | -0,16884 |
| ***Cyp27a1*** | 0,099837 | 0,68324 | 0,612828 | 0,138729 | -0,20747 |
| ***Mt3*** | -0,13576 | 0,394342 | 0,353967 | 0,299001 | 0,405738 |
| ***Rpe65*** | 0,075574 | 0,74217 | 0,201409 | 0,162774 | 0,128783 |
| ***Ppard*** | 0,035931 | 0,504734 | 0,246923 | 0,193196 | 0,287906 |
| ***Bmp7*** | 0,657034 | 0,453625 | -0,06168 | 0,081589 | 0,079248 |
| ***Plekha1*** | 0,642429 | 0,204926 | 0,148417 | 0,191213 | 0,022494 |
| ***Hsd17b8*** | 0,523038 | 0,646799 | 0,059485 | 0,102213 | -0,12863 |
| ***Cebpa*** | 0,565119 | 1,128915 | 0,105326 | -0,23774 | -0,37059 |
| ***Mmp9*** | 0,170831 | 0,469305 | 0,34465 | 0,001376 | 0,194084 |
| ***Acaa1*** | 0,400371 | 0,929744 | 0,067515 | -0,18491 | -0,03445 |
| ***Abcg8*** | -0,39334 | 0,727529 | 0,795213 | 0,269695 | -0,23699 |
| ***Stc1*** | 0,02468 | 0,320192 | 0,401096 | 0,303051 | 0,071845 |
| ***Sdc3*** | 0,13482 | 0,395311 | 0,464591 | 0,077053 | 0,037178 |
| ***Lpl*** | 0,511842 | 0,147046 | 0,090463 | 0,161693 | 0,189924 |
| ***Lcat*** | -0,17959 | 0,579446 | 0,584129 | 0,271463 | -0,21531 |
| ***Ywhag*** | 0,339208 | 0,21306 | 0,216996 | 0,046067 | 0,21918 |
| ***Star*** | -0,06788 | 0,067333 | 0,275318 | 0,342523 | 0,407752 |
| ***Lgmn*** | 0,254681 | 0,226351 | 0,252948 | 0,173124 | 0,088871 |
| ***Bmp15*** | 0,169367 | 0,004754 | 0,173545 | 0,299256 | 0,32277 |
| ***Ran*** | 0,467298 | -0,03497 | 0,166899 | 0,135959 | 0,213766 |
| ***Srd5a1*** | 0,553331 | 0,549495 | -0,07521 | -0,04915 | -0,07225 |
| ***Ptgis*** | -5E-06 | 0,265702 | 0,309336 | 0,12827 | 0,199699 |
| ***Lrat*** | -0,06049 | 0,208794 | 0,084343 | 0,359785 | 0,303068 |
| ***Opn1sw*** | -0,38013 | 0,424165 | 0,250756 | 0,308269 | 0,268251 |
| ***Apoc1*** | 0,167749 | 0,812404 | 0,849487 | -0,26184 | -0,69744 |
| ***Lrp8*** | -0,18127 | -0,79845 | 0,44417 | 0,816511 | 0,569877 |
| ***Mecp2*** | 0,101847 | 0,547118 | 0,096328 | 0,146568 | -0,05329 |
| ***Sdc4*** | 0,136585 | 0,342044 | 0,258386 | 0,035638 | 0,065387 |
| ***Cyp2s1*** | -0,07623 | -0,17694 | 0,083406 | 0,723848 | 0,266478 |
| ***Calb1*** | 0,322164 | 0,059735 | 0,135254 | -0,02045 | 0,317934 |
| ***Adh5*** | 0,051015 | 0,302692 | 0,253513 | 0,082737 | 0,112555 |
| ***Npc1l1*** | 0,22953 | 0,339241 | 0,066838 | 0,126331 | 0,037662 |
| ***Ucn3*** | 0,064518 | 0,13883 | 0,039449 | 0,248824 | 0,301539 |
| ***Bmp2*** | 0,045494 | -0,21566 | 0,359039 | 0,444609 | 0,157484 |
| ***Lrp5*** | -0,01761 | 0,203937 | 0,403935 | 0,285467 | -0,08711 |
| ***Cyp1a1*** | -0,0377 | 0,221035 | 0,38464 | 0,078008 | 0,130361 |
| ***Abca4*** | -0,22516 | 0,274006 | 0,336817 | 0,065528 | 0,289708 |
| ***Il1b*** | 0,263769 | 0,310882 | -0,03707 | 0,045763 | 0,139904 |
| ***Hsd3b2*** | 0,486866 | 0,429578 | -0,0856 | -0,06416 | -0,07272 |
| ***Rdh14*** | 0,185383 | 0,405122 | 0,183859 | -0,01264 | -0,07262 |
| ***Hsd3b7*** | 0,077291 | 0,404465 | 0,271247 | -0,00801 | -0,07627 |
| ***Sp1*** | 0,094922 | 0,310516 | 0,068808 | 0,047906 | 0,139302 |
| ***Plb1*** | -0,00231 | -0,06923 | 0,469515 | 0,048135 | 0,20332 |
| ***Fshb*** | 0,2641 | -0,03187 | -0,13345 | 0,504294 | 0,038416 |
| ***Lhb*** | -0,04052 | 0,342593 | 0,237892 | -0,01577 | 0,103515 |
| ***Por*** | -0,22385 | 0,116112 | 0,419562 | 0,346398 | -0,05581 |
| ***Abca1*** | 0,121641 | 0,292213 | 0,159397 | 0,278491 | -0,26018 |
| ***Akr1c4*** | 0,140055 | 2,312705 | 0,392458 | -1,04147 | -1,21843 |
| ***Pla2g10*** | -0,24089 | 0,31235 | 0,127281 | 0,119424 | 0,250417 |
| ***Lipc*** | -0,33561 | 0,056819 | 0,199002 | 0,359127 | 0,275048 |
| ***Cacna1h*** | -0,19318 | 0,348514 | 0,102798 | -0,01861 | 0,304007 |
| ***Cln6*** | 0,000246 | -0,38528 | 0,431319 | 0,329534 | 0,163236 |
| ***Tnfrsf12a*** | 0,081349 | -0,04782 | 0,114627 | 0,093793 | 0,294744 |
| ***Pip4p1*** | 0,173227 | 0,199086 | -0,01227 | 0,136314 | 0,033213 |
| ***Fdps*** | 0,05692 | 0,55864 | -0,1969 | 0,043325 | 0,059465 |
| ***Tbxas1*** | -0,00737 | 0,224823 | 0,283397 | -0,15924 | 0,16072 |
| ***Scap*** | -0,00978 | 0,275538 | 0,275419 | 0,015266 | -0,06669 |
| ***Ins*** | -0,33103 | 0,458564 | 0,005813 | -0,03666 | 0,391596 |
| ***Cyp11b2*** | 0,076864 | 0,233931 | 0,212893 | -0,2635 | 0,227501 |
| ***Smad2*** | -0,03336 | 0,546036 | -0,00388 | 0,027412 | -0,05172 |
| ***Ucn2*** | -0,19689 | 0,0193 | -0,03087 | 0,323517 | 0,362181 |
| ***Tomm22*** | 0,323483 | -0,01981 | 0,08704 | -0,0045 | 0,067942 |
| ***Ugt2b7*** | 0,55257 | -0,32078 | 0,463908 | -0,14661 | -0,11281 |
| ***Nppa*** | 0,034337 | 0,318987 | 0,116168 | -0,02375 | -0,01468 |
| ***Adcyap1*** | -0,1195 | 0,290491 | -0,08756 | 0,037486 | 0,296229 |
| ***Cyp11a1*** | -0,05775 | 0,377209 | 0,045666 | -0,05022 | 0,096446 |
| ***Rest*** | 0,152846 | 0,340274 | 0,082494 | -0,02373 | -0,15445 |
| ***Pias4*** | -0,07189 | 0,370478 | -0,1006 | 0,1678 | 0,015086 |
| ***Trpv5*** | 0,078389 | 0,395147 | -0,08553 | -0,0178 | 0,00464 |
| ***Diaph1*** | -0,11536 | -0,00192 | 0,120935 | 0,329384 | 0,03452 |
| ***Cyp2d22*** | 0,071351 | 0,689449 | -0,00827 | -0,16298 | -0,23965 |
| ***Sgpl1*** | -0,17167 | 0,147311 | 0,181794 | -0,02678 | 0,196809 |
| ***Amh*** | -0,27951 | 0,015879 | 0,081219 | 0,133097 | 0,329991 |
| ***Fgl1*** | -0,60514 | -0,19903 | 0,468235 | 0,291501 | 0,295674 |
| ***Dpm1*** | -0,0462 | -0,06814 | 0,365244 | 0,169208 | -0,17621 |
| ***Aacs*** | -0,2629 | 0,407739 | 0,053999 | 0,033047 | 0,006437 |
| ***Dpm2*** | 0,087526 | -0,21279 | 0,11198 | 0,011425 | 0,238887 |
| ***Stat5b*** | -0,23896 | 0,314471 | 0,099072 | 0,045493 | 0,011286 |
| ***Ttr*** | -0,01991 | 0,374371 | 0,084331 | -0,10555 | -0,12829 |
| ***Retsat*** | 0,333377 | 0,907362 | 0,159814 | -0,41671 | -0,7916 |
| ***Ldlr*** | -0,19533 | -0,23531 | 0,05352 | 0,181182 | 0,380566 |
| ***Nfyb*** | -0,17682 | 0,471318 | 0,041167 | -0,18105 | 0,02928 |
| ***Hsd11b2*** | -0,26447 | 0,06894 | -0,03823 | 0,08626 | 0,330548 |
| ***Slc27a5*** | -0,19069 | 0,503285 | 0,492936 | -0,04094 | -0,59322 |
| ***Crhr2*** | -0,209 | 0,272133 | 0,154132 | -0,12835 | 0,073522 |
| ***Tnf*** | -0,18177 | -0,4173 | 0,296697 | 0,27893 | 0,183334 |
| ***Apoa2*** | -0,17361 | 0,299471 | 0,622124 | -0,05897 | -0,53274 |
| ***Stat5a*** | -0,24017 | 0,286443 | 0,032579 | -0,0361 | 0,110802 |
| ***Gba*** | -0,08949 | -0,30691 | 0,002287 | 0,281243 | 0,239962 |
| ***Rxra*** | 0,193827 | 0,169862 | 0,015074 | 0,019906 | -0,27327 |
| ***Scarb1*** | -0,17737 | 0,467088 | 0,007424 | 0,012039 | -0,19073 |
| ***Fdx1*** | 0,246646 | 0,014324 | -0,06385 | -0,02432 | -0,06093 |
| ***Nr3c1*** | -0,18234 | 0,22316 | 0,147401 | 0,048842 | -0,12813 |
| ***Cyp2c70*** | -0,17508 | 0,358332 | -0,41738 | -0,00279 | 0,344775 |
| ***Ptpn11*** | 0,277848 | -0,11732 | -0,15029 | 0,091185 | -0,0196 |
| ***Tspo*** | 0,199444 | -0,10564 | -0,08693 | -0,0227 | 0,093259 |
| ***Kpnb1*** | -0,00156 | -0,1564 | -0,03799 | 0,218253 | 0,053538 |
| ***Igfbp4*** | 0,023353 | 0,525892 | -0,55693 | 0,084373 | -0,00613 |
| ***Smpd1*** | 0,298504 | -0,25833 | -0,03771 | 0,003571 | 0,063222 |
| ***Htt*** | -0,11077 | -0,14556 | 0,015363 | 0,065637 | 0,222904 |
| ***Tnfsf4*** | 0,001562 | 0,438727 | -0,11306 | -0,08168 | -0,20057 |
| ***Cat*** | 0,079423 | 0,133675 | -0,05435 | -0,08199 | -0,03185 |
| ***Kcnma1*** | -0,29753 | 0,071285 | 0,058732 | 0,166292 | 0,045092 |
| ***Ahr*** | -0,14658 | 0,313906 | -0,04025 | -0,03685 | -0,07687 |
| ***Eno1*** | -0,11762 | -0,22969 | 0,06584 | 0,125296 | 0,143872 |
| ***Msmo1*** | -0,14019 | -0,50058 | 0,219667 | 0,165779 | 0,220969 |
| ***Rdh5*** | -0,31391 | 0,133303 | 0,132877 | 0,120966 | -0,1159 |
| ***Bmp4*** | 0,171514 | 0,081263 | -0,73047 | -0,11983 | 0,554589 |
| ***Sqle*** | -0,67397 | 0,014226 | 0,11547 | 0,248925 | 0,236359 |
| ***G6pd*** | -0,91356 | -0,81963 | 0,563423 | 0,760106 | 0,3419 |
| ***Gba2*** | 0,300028 | -0,10983 | -0,25287 | 0,121265 | -0,13927 |
| ***Srebf1*** | -0,75619 | 0,685746 | 0,064183 | -0,03825 | -0,08533 |
| ***Angptl3*** | -0,01735 | 0,479563 | 0,012199 | 0,059137 | -0,67252 |
| ***Cyp27b1*** | -0,68583 | 0,242121 | 0,083633 | 0,047049 | 0,156222 |
| ***Apof*** | -0,10418 | 0,377569 | -0,25427 | -0,25009 | 0,07323 |
| ***Cnbp*** | -0,1978 | 0,164906 | 0,009302 | -0,14764 | -0,01498 |
| ***Ces1*** | -0,12385 | 0,340526 | 0,219336 | 0,011072 | -0,6343 |
| ***Apoa1*** | 0,200219 | -0,07687 | -0,25979 | -0,08649 | 0,018263 |
| ***Pcsk9*** | -0,52601 | -0,42503 | 0,039007 | 0,336342 | 0,359866 |
| ***Fech*** | 0,206081 | -0,2036 | -0,07492 | -0,06584 | -0,07879 |
| ***Hnf1a*** | 0,141772 | -0,46934 | 0,133149 | 0,073791 | -0,10086 |
| ***Comt*** | 0,338135 | -0,06313 | -0,25532 | -0,2088 | -0,03251 |
| ***Inha*** | -0,42801 | -0,00526 | 0,147948 | -0,0045 | 0,066486 |
| ***Atp8b1*** | -0,01227 | -0,40007 | -0,09746 | 0,211675 | 0,070155 |
| ***Gpc4*** | 0,035287 | 0,113513 | -0,53848 | -0,0201 | 0,18069 |
| ***Hspa8*** | -0,01242 | -0,20995 | -0,02698 | -0,01897 | 0,028543 |
| ***Rara*** | 0,118619 | -0,32306 | -0,15279 | 0,129174 | -0,01458 |
| ***Dbi*** | 0,109036 | 0,036403 | -0,24376 | -0,13536 | -0,03999 |
| ***Pex2*** | 0,068347 | -0,15174 | 0,080045 | -0,24601 | -0,03156 |
| ***Rhoa*** | -0,13676 | -0,1495 | 0,013621 | -0,03237 | 0,023586 |
| ***Adh7*** | -0,01942 | 0,083544 | -0,20337 | 0,123429 | -0,29415 |
| ***Fgf7*** | -0,08165 | 0,136288 | -0,19965 | 0,163945 | -0,33884 |
| ***Nsdhl*** | -0,15331 | -0,00855 | -0,30276 | -0,01815 | 0,15572 |
| ***Pmvk*** | -0,38822 | 0,200157 | 0,036589 | -0,12994 | -0,08375 |
| ***Dhrs4*** | 0,191709 | 0,457548 | -0,36069 | -0,35123 | -0,30604 |
| ***Apoe*** | 0,00429 | 0,122289 | -0,03966 | -0,30261 | -0,15475 |
| ***Lss*** | -0,37367 | -0,13491 | 0,009673 | -0,05388 | 0,161206 |
| ***Mbtps1*** | 0,062942 | 0,065451 | -0,41234 | -0,03739 | -0,08008 |
| ***Serpina6*** | -0,73851 | 0,531935 | 0,070508 | -0,01966 | -0,2586 |
| ***Adh1c*** | -0,16409 | 0,881367 | -0,32695 | -0,40502 | -0,41997 |
| ***Sdc1*** | -0,55053 | 0,280058 | -0,0771 | -0,12444 | 0,036384 |
| ***Cln8*** | -0,0856 | -0,00625 | 0,235482 | -0,15397 | -0,4303 |
| ***Ebp*** | -0,22105 | 0,471879 | -0,38572 | -0,19354 | -0,11227 |
| ***Rdh12*** | -0,20221 | -0,40604 | 0,113847 | 0,009118 | 0,040574 |
| ***Nr0b2*** | -0,35622 | 0,233567 | 0,047325 | -0,39205 | 0,021575 |
| ***Gsta3*** | 0,082941 | 0,207171 | -0,25599 | -0,34971 | -0,13187 |
| ***Naaa*** | -0,04221 | -0,2246 | -0,09696 | 0,172792 | -0,2631 |
| ***Cyp51a1*** | -0,32394 | -0,46856 | 0,011095 | 0,040245 | 0,26191 |
| ***Cyp4v2*** | -0,06582 | 1,621262 | -0,45752 | -0,78953 | -0,79901 |
| ***Ywhah*** | 0,047272 | -0,40727 | -0,13391 | -0,07661 | 0,042428 |
| ***Cubn*** | 0,047163 | 0,041219 | -0,36452 | -0,21201 | -0,05415 |
| ***Hmgcr*** | -1,05361 | 0,128838 | 0,194174 | -0,00519 | 0,16514 |
| ***Nfya*** | -0,18407 | -0,06289 | -0,25294 | -0,10811 | 0,011487 |
| ***Rdh7*** | -0,09397 | 0,72575 | -0,39211 | -0,43639 | -0,4015 |
| ***Mvd*** | -0,48055 | -0,09876 | -0,14523 | -0,04437 | 0,167651 |
| ***Sc5d*** | -0,31835 | 0,126571 | -0,17349 | -0,20497 | -0,04536 |
| ***Cyp2c18*** | -0,2453 | 0,259722 | 0,049139 | -0,39986 | -0,29794 |
| ***Hsd11b1*** | -0,18894 | 0,270435 | -0,05613 | -0,22592 | -0,44326 |
| ***Lbr*** | -0,33512 | 0,072834 | -0,30892 | -0,01907 | -0,07172 |
| ***Opn1lw*** | -0,46082 | 0,010324 | -0,02205 | -0,09683 | -0,10304 |
| ***Acly*** | -0,53178 | -0,15754 | -0,04428 | 0,10239 | -0,05604 |
| ***Stard3*** | -0,19645 | -0,28329 | -0,0961 | -0,05702 | -0,06698 |
| ***Rora*** | 0,248717 | 0,183908 | -0,43177 | -0,19966 | -0,52553 |
| ***Cyp2j2*** | 0,072261 | 0,23827 | -0,40419 | -0,37816 | -0,2528 |
| ***Cyp3a7*** | -0,36401 | -0,43583 | -0,05304 | 0,112209 | 0,001546 |
| ***Slc37a4*** | -0,25393 | 0,120803 | 0,431362 | -0,25127 | -0,78958 |
| ***Amacr*** | 0,266114 | 0,465913 | -0,78026 | -0,35673 | -0,34628 |
| ***Plg*** | -0,03442 | 0,501651 | -0,11731 | -0,58013 | -0,52523 |
| ***Scd*** | -0,13432 | 0,289914 | 0,097704 | -0,38032 | -0,63388 |
| ***Sec14l2*** | 0,01917 | 0,028963 | -0,1995 | -0,16686 | -0,45464 |
| ***Arv1*** | -0,01046 | -0,2308 | -0,34189 | -0,03724 | -0,161 |
| ***Cyp2c9*** | -0,67462 | 0,257784 | 0,167694 | -0,2441 | -0,31643 |
| ***Cga*** | -0,51409 | -0,06749 | -0,12433 | -0,08328 | -0,04048 |
| ***Foxa2*** | 0,050876 | -0,561 | -0,04803 | -0,12144 | -0,16052 |
| ***Npc2*** | -0,37777 | -0,36177 | -0,13016 | -0,07041 | 0,090643 |
| ***Gpc3*** | -0,27275 | 0,211089 | 0,119358 | -0,57628 | -0,34262 |
| ***Plpp6*** | -0,24057 | -0,29598 | -0,15455 | -0,10519 | -0,07508 |
| ***Dhcr7*** | -0,22865 | -0,15788 | -0,20414 | -0,13482 | -0,15003 |
| ***Cyp26a1*** | -0,88258 | 0,748972 | -0,40208 | -0,30796 | -0,03217 |
| ***Aplp2*** | -0,15038 | -0,16879 | -0,28909 | -0,12813 | -0,14523 |
| ***Pctp*** | -0,4482 | -0,24054 | 0,059516 | -0,06193 | -0,19305 |
| ***Cyp2r1*** | 0,002848 | -0,22624 | -0,48449 | 0,007789 | -0,19752 |
| ***Nr1h3*** | 0,045567 | -0,18155 | -0,28072 | -0,24717 | -0,25165 |
| ***Bco1*** | -0,40829 | -1,00127 | 0,010991 | 0,36092 | 0,102706 |
| ***Hsd17b4*** | 0,162203 | -0,53786 | -0,16633 | -0,25361 | -0,24445 |
| ***Lrp1*** | 0,060206 | -0,55842 | -0,2624 | -0,11997 | -0,17952 |
| ***Abcg5*** | -0,60954 | 0,480023 | 0,03509 | -0,40794 | -0,56029 |
| ***Ttpa*** | -0,31002 | -0,3907 | -0,22847 | -0,03957 | -0,11531 |
| ***Bco2*** | -0,29506 | 0,14775 | -0,18341 | -0,42434 | -0,347 |
| ***Fdft1*** | -0,46574 | -0,23617 | -0,21606 | -0,15921 | -0,03858 |
| ***Agrn*** | -0,11492 | -0,60613 | -0,503 | -0,00508 | 0,10244 |
| ***Fgf1*** | -0,08053 | 0,240161 | -0,10593 | -0,55711 | -0,6425 |
| ***Srebf2*** | -0,43648 | -0,59157 | -0,18447 | -0,00592 | 0,067099 |
| ***Hsd17b7*** | -0,3581 | -0,62489 | -0,219 | 0,014853 | 0,034697 |
| ***Aldh1a1*** | 0,149176 | -0,68979 | -0,46523 | -0,18232 | 0,010048 |
| ***Aldh1a7*** | 0,005718 | -0,78892 | -0,30654 | -0,03241 | -0,06392 |
| ***Rdh11*** | -0,58042 | -0,44605 | 0,203302 | -0,23593 | -0,13288 |
| ***Cyp2g1*** | -0,24665 | 0,061046 | -0,38795 | -0,30989 | -0,32364 |
| ***Slco1a6*** | -0,20543 | -0,09649 | -0,25649 | -0,36321 | -0,33449 |
| ***Rdh10*** | -0,35614 | -0,46279 | 0,167625 | -0,14453 | -0,46173 |
| ***Dpagt1*** | -0,19469 | -0,57393 | -0,21439 | -0,15736 | -0,19059 |
| ***Npc1*** | -0,24579 | 0,014381 | -0,29323 | -0,412 | -0,40126 |
| ***Rarres2*** | -0,44404 | 0,208827 | -0,2729 | -0,33708 | -0,50316 |
| ***Xdh*** | -0,18054 | -0,28762 | -0,22192 | -0,2607 | -0,40273 |
| ***Inhba*** | -0,37941 | -0,2869 | -0,73343 | -0,23338 | 0,275028 |
| ***Aspa*** | -0,15206 | 0,33452 | -0,71284 | -0,415 | -0,42564 |
| ***Pde8a*** | -0,15892 | -0,55505 | -0,20848 | -0,11567 | -0,37352 |
| ***Sdc2*** | -0,18517 | 0,076258 | -0,5412 | -0,48652 | -0,28699 |
| ***Dhrs3*** | -0,16063 | 0,452663 | -0,29804 | -0,74504 | -0,67502 |
| ***Sult1e1*** | -0,77274 | 0,088906 | 0,193589 | -0,32624 | -0,62783 |
| ***Idi1*** | -0,30347 | -1,16207 | -0,29405 | 0,118936 | 0,18407 |
| ***Agt*** | -0,32361 | 0,085077 | -0,52515 | -0,41596 | -0,29566 |
| ***Hmgcs1*** | -0,42746 | -0,5808 | -0,25948 | -0,22627 | 0,013754 |
| ***Rorc*** | -0,31607 | -0,26802 | 0,325609 | -0,41753 | -0,81895 |
| ***Foxa1*** | 0,558808 | -0,8413 | -0,40635 | -0,48428 | -0,32452 |
| ***Soat2*** | -0,2558 | -0,20424 | -0,22728 | -0,25872 | -0,55695 |
| ***Apoa4*** | -0,46224 | -0,92768 | -0,32289 | -0,02632 | 0,2323 |
| ***Tp53*** | -0,03313 | -0,694 | -0,51593 | -0,13647 | -0,15904 |
| ***Nr1i2*** | -0,21815 | -0,37089 | -0,35606 | -0,21664 | -0,3979 |
| ***Cbr1*** | -0,5036 | -0,68863 | 0,2454 | -0,26627 | -0,35872 |
| ***Fasn*** | -0,78593 | -0,47232 | -0,08825 | 0,042331 | -0,31419 |
| ***Gpam*** | -0,22696 | -0,85832 | -0,13764 | -0,24223 | -0,15404 |
| ***Rdh16*** | -1,11795 | 0,483381 | -0,21248 | -0,30865 | -0,46387 |
| ***Nr5a2*** | -0,20778 | -0,56672 | -0,47756 | -0,20032 | -0,18869 |
| ***Esr1*** | -0,8671 | 0,042478 | -0,57455 | -0,17213 | -0,09752 |
| ***Acat2*** | -0,12932 | -0,64627 | -0,3273 | -0,35617 | -0,22326 |
| ***Akr1b1*** | 0,044134 | -0,50866 | -0,45581 | -0,40216 | -0,37285 |
| ***Napepld*** | -0,19458 | -1,27637 | -0,57216 | 0,121144 | 0,160326 |
| ***Nr1h4*** | 0,255714 | -0,60739 | -0,50502 | -0,55488 | -0,43199 |
| ***Hnf1b*** | 0,045085 | -1,06152 | -0,47855 | -0,14639 | -0,31524 |
| ***Apom*** | -0,33283 | -0,1507 | -0,93804 | -0,45161 | -0,17364 |
| ***Cyp4f12*** | -0,20052 | 1,162041 | -0,30998 | -1,52239 | -1,1952 |
| ***Pon1*** | -0,20672 | 0,160498 | 0,102142 | -0,76871 | -1,36881 |
| ***Aldh8a1*** | -0,129 | 0,635762 | -0,37848 | -0,93457 | -1,40599 |
| ***Itga6*** | -0,97647 | -0,70438 | -0,40466 | -0,08911 | -0,08462 |
| ***Akr1b10*** | -0,28129 | -0,34383 | -0,60945 | -0,59494 | -0,43029 |
| ***Nrg1*** | 0,098486 | -1,20854 | -0,67924 | -0,44801 | -0,11436 |
| ***App*** | -0,64282 | -0,94085 | -0,48604 | -0,276 | -0,02858 |
| ***Pltp*** | -0,54254 | -0,02021 | -0,53789 | -0,73388 | -0,55614 |
| ***Pmp22*** | 0,090633 | -0,13117 | -1,17002 | -0,8847 | -0,40893 |
| ***Dhcr24*** | -0,3609 | -1,20499 | -0,57054 | -0,21409 | -0,17061 |
| ***Vldlr*** | -1,65317 | -0,33405 | 0,599907 | -0,31061 | -0,84224 |
| ***Schip1*** | -0,32029 | -1,09387 | -0,48847 | -0,50288 | -0,15073 |
| ***Baat*** | -0,02758 | -0,14993 | -0,88211 | -0,74723 | -0,82395 |
| ***Lipa*** | -0,39884 | -1,07622 | -0,60296 | -0,43253 | -0,17624 |
| ***Tnfsf10*** | -0,76842 | -0,42081 | -0,89224 | -0,39771 | -0,27281 |
| ***Akr1c14*** | 0,028758 | 0,144925 | -1,63113 | -0,93911 | -0,36452 |
| ***Ppargc1a*** | -0,79412 | -0,38501 | -0,49487 | -0,42794 | -0,66493 |
| ***Cyp7a1*** | -0,10229 | 1,351961 | -0,28199 | -1,67746 | -2,37028 |
| ***Acaca*** | -0,81725 | -0,50812 | -0,39237 | -0,72828 | -0,71412 |
| ***Sult1a1*** | -0,73322 | -1,18578 | -0,9819 | -0,42152 | -0,10652 |
| ***G6pc*** | -0,13059 | 0,340476 | -0,01617 | -1,58284 | -2,08482 |
| ***Afp*** | 0,056886 | -0,48276 | -1,36436 | -1,32084 | -0,57073 |
| ***Rbp1*** | -0,73896 | -0,81934 | -1,09913 | -0,56668 | -0,49116 |
| ***Srd5a2*** | -0,44862 | -1,36603 | -0,96258 | -0,54396 | -0,40065 |
| ***Elovl6*** | -1,0008 | -1,48377 | -0,7571 | -0,17717 | -0,38 |
| ***Maoa*** | -0,70329 | -0,4951 | -1,32169 | -1,29354 | -0,54919 |
| ***Cyp39a1*** | -1,16036 | -1,21124 | -1,23069 | -0,78684 | -0,36593 |
| ***Akr1d1*** | -0,74179 | 0,643042 | -0,61832 | -1,92256 | -2,62267 |
| ***Akr1c3*** | -0,27282 | -0,12655 | -2,54033 | -1,89342 | -0,62448 |
| ***Lepr*** | -1,18575 | -0,92697 | -0,63828 | -1,08262 | -1,81091 |
| ***Cyp17a1*** | -3,07659 | -1,66558 | 0,31134 | 0,14851 | -1,75611 |
| ***Prlr*** | -1,84625 | -0,84082 | -1,60129 | -1,87303 | -1,77616 |
| ***Cyp2a6*** | -1,61421 | -1,43386 | -1,73229 | -1,86785 | -2,32511 |
| ***Sult2a1*** | -3,65458 | -0,33344 | -1,47038 | -1,9286 | -2,14337 |
| ***Cyp2b6*** | -4,87545 | -1,58102 | -1,3552 | -1,03239 | -1,02875 |
| ***Cyp3a5*** | -4,0533 | -3,72033 | -2,12901 | 0,626905 | -0,99918 |
| ***Cyp2c8*** | -3,0275 | -2,40431 | -1,64767 | -1,56727 | -2,10398 |
| ***Cyp2b13/cyp2b9*** | -7,26718 | -4,61594 | -3,54464 | -3,04065 | -2,84884 |

**Table S15:** Activation Z-score of genes involved in the synthesis of lipids male vs. female. Activation Z-score was calculated with IPA software from Qiagen.

| **Pathway Summary** | -2,836 | -0,762 | -1,981 | -2,804 | -2,414 |
| --- | --- | --- | --- | --- | --- |
| **genes in the synthesis of lipid network** | **0 h** | **24 h** | **48 h** | **72 h** | **96 h** |
| ***Elovl3*** | 5,046876 | 6,186568 | 2,899638 | 1,088746 | 0,664992 |
| ***Hsd3b4*** | 5,399488 | 4,572805 | 1,809618 | 0,39681 | 0,154409 |
| ***Serpine2*** | 1,837447 | 2,145044 | 2,285696 | 2,767708 | 2,700515 |
| ***Cyp7b1*** | 2,97897 | 3,393742 | 1,334546 | 0,90291 | 1,035637 |
| ***Fos*** | 0,477946 | 1,616629 | 1,925766 | 1,628887 | 1,742349 |
| ***Obp2b*** | 2,916129 | 1,226669 | 0,565316 | 0,405337 | 0,308385 |
| ***Atf3*** | 0,530338 | 1,134128 | 1,22024 | 1,094871 | 1,103943 |
| ***Serpina1*** | 1,141492 | 1,2407 | 1,497335 | 0,705409 | 0,48324 |
| ***Rgs2*** | 0,023304 | 1,504444 | 1,533327 | 1,273943 | 0,662307 |
| ***Scp2*** | 0,905224 | 2,031537 | 0,985545 | 0,350656 | 0,253287 |
| ***Egr1*** | 0,200368 | 0,967559 | 1,098924 | 1,112761 | 1,004121 |
| ***F2r*** | 1,338302 | 0,862247 | 0,770082 | 0,465858 | 0,599782 |
| ***Gdf15*** | 0,203624 | 1,295703 | 0,569867 | 0,822218 | 1,120081 |
| ***Cyp2e1*** | 0,155034 | 0,940182 | 2,273941 | 0,984777 | -0,50221 |
| ***Hmox1*** | 0,452339 | 0,564182 | 1,329974 | 0,634012 | 0,537807 |
| ***Fabp4*** | -0,12234 | 1,045039 | 1,446707 | 0,929734 | 0,149975 |
| ***Cxcl10*** | -0,03222 | 1,066905 | 0,903724 | 0,776905 | 0,529257 |
| ***Pkm*** | -0,08209 | 0,184047 | 0,898776 | 1,488772 | 0,741155 |
| ***Trib1*** | 0,651969 | 1,055379 | 0,313726 | 0,517737 | 0,678966 |
| ***Gpc1*** | 0,663466 | 0,725001 | 0,593157 | 0,60759 | 0,525036 |
| ***Cyp21a2*** | 0,032534 | 0,422623 | 0,500586 | 0,877801 | 1,143798 |
| ***Pgf*** | 0,079831 | 0,30252 | 1,032182 | 1,148335 | 0,332997 |
| ***Fabp1*** | 0,377904 | 2,480121 | 1,289524 | -0,39195 | -0,87061 |
| ***Egfr*** | 1,511161 | 0,917405 | 0,196501 | 0,031613 | 0,151305 |
| ***Areg*** | -0,16675 | 1,281483 | 0,731572 | 0,577384 | 0,288823 |
| ***Vegfa*** | -0,06545 | 0,80094 | 0,960118 | 0,861856 | 0,106438 |
| ***Flt1*** | 0,151854 | 0,164681 | 0,884142 | 0,863593 | 0,381496 |
| ***Cxcl2*** | 0,96606 | 0,145288 | 0,10557 | 0,506642 | 0,672174 |
| ***Bnip3*** | -0,06286 | 0,412747 | 0,954217 | 1,005272 | 0,084623 |
| ***Brd2*** | 0,267152 | 0,532132 | 0,458618 | 0,526304 | 0,486191 |
| ***Ednrb*** | 0,011695 | 1,038317 | 0,813442 | 0,432524 | -0,0453 |
| ***Cav1*** | 0,773773 | 0,658437 | 0,150822 | 0,097915 | 0,546004 |
| ***Cebpb*** | 0,197873 | 0,933786 | 0,337535 | 0,344411 | 0,258395 |
| ***Cyp8b1*** | 0,538241 | 1,700563 | 1,224542 | -0,39323 | -1,03816 |
| ***B3galt1*** | 0,823736 | 1,057828 | 0,130182 | 0,008273 | -0,03934 |
| ***Nfil3*** | 0,365117 | 0,769658 | 0,357472 | 0,323207 | 0,144676 |
| ***Grem2*** | 0,396421 | 0,714194 | 0,50747 | 0,47375 | -0,14161 |
| ***Serinc2*** | 1,050041 | 0,24407 | 0,028299 | 0,286496 | 0,318304 |
| ***Arsa*** | 1,038985 | 0,228933 | 0,338002 | 0,204897 | 0,008078 |
| ***Spp1*** | 0,356681 | 0,816593 | -0,63844 | 0,132484 | 1,13767 |
| ***Abcc1*** | -0,32994 | 0,570196 | 0,756808 | 0,448079 | 0,320496 |
| ***Ptges*** | -0,40226 | 0,469452 | 1,145467 | 0,366267 | 0,178699 |
| ***St8sia3*** | 0,202583 | 0,451822 | 0,988341 | 0,357582 | -0,2478 |
| ***Igf1*** | -0,0711 | 0,534636 | 0,478904 | 0,342446 | 0,463394 |
| ***Adh4*** | 0,843657 | 1,099951 | -0,02072 | -0,12201 | -0,06158 |
| ***Chpt1*** | 0,69388 | 1,210534 | 0,156533 | -0,12785 | -0,20762 |
| ***Socs3*** | 0,648692 | 0,44729 | 0,310238 | 0,230759 | 0,070863 |
| ***Bmp5*** | 0,17196 | 0,321528 | 0,446983 | 0,35513 | 0,404198 |
| ***Tac1*** | 0,27023 | 0,636432 | 0,47235 | 0,056696 | 0,253429 |
| ***Cyp1a2*** | 0,066898 | 1,509168 | 1,215758 | 0,619908 | -1,80321 |
| ***Ccn1*** | 0,813415 | -0,28874 | 0,297317 | 0,371764 | 0,414685 |
| ***Tlr4*** | -0,14703 | -0,00808 | 0,78129 | 0,494066 | 0,451944 |
| ***Kitlg*** | -0,02606 | 1,06252 | 0,234838 | 0,176335 | 0,112236 |
| ***Prkab2*** | -0,04086 | 0,604595 | 0,730222 | 0,366204 | -0,11625 |
| ***Bdnf*** | 0,244529 | 0,182207 | 0,014395 | 0,402227 | 0,692226 |
| ***Mitf*** | 0,478192 | -0,53408 | 0,256864 | 0,645143 | 0,677926 |
| ***Cyp2c23*** | 0,19853 | 1,306753 | 0,733897 | -0,19629 | -0,54483 |
| ***Klf4*** | -0,09039 | 0,189796 | 0,650331 | 0,501759 | 0,217074 |
| ***Eif4ebp1*** | 0,246025 | 0,279086 | 0,437429 | 0,286157 | 0,203797 |
| ***Kit*** | 0,288402 | 0,247651 | 0,559729 | 0,24703 | 0,108773 |
| ***Sh3kbp1*** | -0,24015 | 0,046639 | 0,940951 | 0,584719 | 0,116631 |
| ***Piga*** | 0,368322 | -0,09892 | 0,381809 | 0,510237 | 0,275699 |
| ***Tgfbr2*** | 0,22758 | 0,159388 | 0,48169 | 0,38976 | 0,164781 |
| ***Apoa5*** | -0,00171 | 1,200094 | 0,038027 | 0,117437 | 0,054655 |
| ***Cyp27a1*** | 0,099837 | 0,68324 | 0,612828 | 0,138729 | -0,20747 |
| ***Chrm3*** | 0,070267 | 0,431198 | 0,312648 | 0,231237 | 0,281056 |
| ***Igfbp2*** | 0,031061 | 0,922081 | 0,547374 | -0,04514 | -0,18594 |
| ***Ppard*** | 0,035931 | 0,504734 | 0,246923 | 0,193196 | 0,287906 |
| ***F2rl1*** | 0,068704 | -0,13066 | 0,236344 | 0,442689 | 0,606844 |
| ***Sirt1*** | -0,0669 | 0,763695 | 0,318872 | 0,227024 | -0,0192 |
| ***Hsd3b1*** | 0,876791 | 1,765216 | -0,32726 | -0,37014 | -0,72399 |
| ***Avp*** | 0,105017 | 0,100225 | 0,215471 | 0,348399 | 0,448057 |
| ***Bmp7*** | 0,657034 | 0,453625 | -0,06168 | 0,081589 | 0,079248 |
| ***Plekha1*** | 0,642429 | 0,204926 | 0,148417 | 0,191213 | 0,022494 |
| ***Ccl3l3*** | 0,240281 | 0,444464 | 0,384787 | 0,111675 | 0,025613 |
| ***Hsd17b8*** | 0,523038 | 0,646799 | 0,059485 | 0,102213 | -0,12863 |
| ***Cox7a2*** | 0,400648 | 0,258451 | 0,324671 | 0,123163 | 0,094148 |
| ***Mif*** | 0,042114 | 0,031338 | 0,387972 | 0,552037 | 0,186744 |
| ***Acsl1*** | 0,275928 | 1,416515 | 0,136382 | -0,25224 | -0,37723 |
| ***Pla2g5*** | 0,00819 | 0,536913 | 0,324362 | 0,118147 | 0,210228 |
| ***Sbf1*** | 0,456461 | 0,18058 | 0,121724 | 0,403903 | 0,034987 |
| ***Cebpa*** | 0,565119 | 1,128915 | 0,105326 | -0,23774 | -0,37059 |
| ***Mmp9*** | 0,170831 | 0,469305 | 0,34465 | 0,001376 | 0,194084 |
| ***Jazf1*** | -0,16473 | 0,30409 | 0,485301 | 0,189069 | 0,365711 |
| ***Cotl1*** | 0,14868 | 0,128827 | 0,200416 | 0,450562 | 0,248541 |
| ***Fosl1*** | -0,25278 | -0,43857 | 0,77401 | 0,646088 | 0,437394 |
| ***Abcg8*** | -0,39334 | 0,727529 | 0,795213 | 0,269695 | -0,23699 |
| ***Dpm3*** | 0,512559 | 0,19345 | 0,271458 | 0,112836 | 0,063532 |
| ***Runx1*** | -0,19519 | 0,095331 | 0,451294 | 0,511187 | 0,289887 |
| ***Anxa1*** | -0,06229 | -0,012 | 0,251502 | 0,404198 | 0,569906 |
| ***Rhoq*** | -0,2231 | 0,116176 | 0,408781 | 0,373661 | 0,463862 |
| ***Stc1*** | 0,02468 | 0,320192 | 0,401096 | 0,303051 | 0,071845 |
| ***Enpp2*** | 0,28131 | 1,556747 | -0,15064 | -0,2265 | -0,34825 |
| ***Lpl*** | 0,511842 | 0,147046 | 0,090463 | 0,161693 | 0,189924 |
| ***Btc*** | -0,11052 | 0,476393 | 0,300789 | 0,370847 | 0,063309 |
| ***Npr1*** | 0,224399 | 0,375116 | 0,130558 | 0,064464 | 0,301067 |
| ***Csnk2a2*** | 0,144079 | 0,411782 | 0,260595 | 0,257485 | 0,005596 |
| ***Ormdl3*** | 0,273645 | 0,701466 | 0,521223 | -0,18276 | -0,24647 |
| ***Trpv2*** | -0,07484 | 0,305257 | 0,305964 | 0,296258 | 0,227566 |
| ***Nr1d1*** | 0,702155 | 0,817909 | -0,12459 | -0,16076 | -0,17878 |
| ***Ffar4*** | -0,31033 | 0,062398 | 0,941238 | 0,43388 | -0,07842 |
| ***C5*** | 0,293912 | 1,290041 | -0,01347 | -0,21699 | -0,30486 |
| ***Tgfa*** | 0,387056 | 0,210526 | 0,146732 | 0,097411 | 0,206395 |
| ***Lcat*** | -0,17959 | 0,579446 | 0,584129 | 0,271463 | -0,21531 |
| ***Ywhag*** | 0,339208 | 0,21306 | 0,216996 | 0,046067 | 0,21918 |
| ***Ephx2*** | 0,424815 | 0,609694 | -0,06428 | 0,03513 | 0,023852 |
| ***Star*** | -0,06788 | 0,067333 | 0,275318 | 0,342523 | 0,407752 |
| ***Thrsp*** | -0,07521 | 1,394794 | 0,232433 | -0,05143 | -0,476 |
| ***Pip5k1b*** | -0,1052 | 0,344318 | 0,046992 | 0,21469 | 0,522942 |
| ***Polg*** | 0,054702 | 0,088632 | 0,332825 | 0,303947 | 0,24021 |
| ***Hacd4*** | 0,159192 | 0,10177 | 0,42503 | 0,169659 | 0,164038 |
| ***Atf4*** | -0,07623 | 0,409375 | 0,25378 | 0,177375 | 0,221857 |
| ***Chka*** | 0,426351 | 0,16697 | -0,22775 | 0,077874 | 0,541396 |
| ***Map2k1*** | 0,13442 | 0,207022 | 0,290068 | 0,177577 | 0,173668 |
| ***Bmp15*** | 0,169367 | 0,004754 | 0,173545 | 0,299256 | 0,32277 |
| ***Ins1*** | -0,15546 | 0,311846 | 0,152701 | 0,398085 | 0,245218 |
| ***Slc22a1*** | 0,081045 | 0,96672 | 0,360792 | -0,08863 | -0,3697 |
| ***Ran*** | 0,467298 | -0,03497 | 0,166899 | 0,135959 | 0,213766 |
| ***Inpp4a*** | 0,320868 | 0,314506 | 0,062814 | 0,200973 | 0,039548 |
| ***Alox5*** | 0,097119 | 0,514201 | -0,04708 | 0,266093 | 0,107334 |
| ***Ltf*** | 0,176336 | 0,19017 | 0,361557 | 0,134003 | 0,049525 |
| ***P2rx1*** | 0,267499 | 0,44075 | 0,267486 | -0,25087 | 0,184817 |
| ***Plin1*** | 0,023749 | 0,189395 | 0,063837 | 0,152108 | 0,479431 |
| ***Srd5a1*** | 0,553331 | 0,549495 | -0,07521 | -0,04915 | -0,07225 |
| ***Ptgis*** | -5E-06 | 0,265702 | 0,309336 | 0,12827 | 0,199699 |
| ***Pdgfa*** | -0,25078 | 0,417067 | 0,276433 | 0,222595 | 0,232966 |
| ***Lrat*** | -0,06049 | 0,208794 | 0,084343 | 0,359785 | 0,303068 |
| ***Gapvd1*** | 0,271123 | -0,11465 | 0,161051 | 0,290098 | 0,277341 |
| ***Oxt*** | 0,135888 | 0,12567 | 0,109471 | 0,174866 | 0,337703 |
| ***Il17a*** | -0,14899 | 0,064948 | 0,589141 | 0,131666 | 0,233734 |
| ***Esr2*** | -0,00057 | 0,018629 | 0,301336 | 0,113546 | 0,433366 |
| ***Abhd6*** | 0,049894 | -0,21583 | 0,5008 | 0,346503 | 0,159471 |
| ***Atf1*** | 0,112945 | 0,332149 | 0,056956 | 0,17816 | 0,154778 |
| ***Ednra*** | 0,005759 | 0,234124 | 0,26893 | -0,09709 | 0,414275 |
| ***Pgs1*** | 0,061149 | 0,242366 | -0,00696 | 0,322102 | 0,20377 |
| ***Calb1*** | 0,322164 | 0,059735 | 0,135254 | -0,02045 | 0,317934 |
| ***Gla*** | -0,25347 | -0,00908 | 0,31602 | 0,245103 | 0,504942 |
| ***Adh5*** | 0,051015 | 0,302692 | 0,253513 | 0,082737 | 0,112555 |
| ***Npc1l1*** | 0,22953 | 0,339241 | 0,066838 | 0,126331 | 0,037662 |
| ***Ucn3*** | 0,064518 | 0,13883 | 0,039449 | 0,248824 | 0,301539 |
| ***Bmp2*** | 0,045494 | -0,21566 | 0,359039 | 0,444609 | 0,157484 |
| ***Synj2*** | 0,53206 | 0,072132 | 0,075766 | 0,028447 | 0,074279 |
| ***Cyp1a1*** | -0,0377 | 0,221035 | 0,38464 | 0,078008 | 0,130361 |
| ***Nr4a3*** | -0,16451 | 0,339718 | 0,166224 | 0,009761 | 0,423004 |
| ***F2*** | 0,060368 | 0,238874 | 0,224434 | 0,151489 | 0,097842 |
| ***Cd4*** | 0,426659 | 0,284947 | -0,12784 | -0,09186 | 0,280133 |
| ***Etnk2*** | -0,15626 | 1,262089 | 0,200795 | -0,00099 | -0,5342 |
| ***Pdgfrb*** | -0,09307 | 0,304447 | 0,26106 | 0,131816 | 0,161443 |
| ***B4galt1*** | 0,149573 | 0,309996 | 0,244133 | 0,099127 | -0,04937 |
| ***Abca4*** | -0,22516 | 0,274006 | 0,336817 | 0,065528 | 0,289708 |
| ***Pi4k2a*** | 0,204324 | 0,087389 | 0,248339 | 0,139342 | 0,058594 |
| ***Fitm2*** | 0,328933 | 0,147821 | 0,42145 | 0,054906 | -0,21785 |
| ***Sod1*** | -0,00286 | 0,235516 | 0,313475 | 0,189561 | -0,0042 |
| ***Sf1*** | 0,215541 | 0,261608 | 0,075138 | 0,162234 | 0,014766 |
| ***Il1b*** | 0,263769 | 0,310882 | -0,03707 | 0,045763 | 0,139904 |
| ***Mtmr3*** | 0,383555 | 0,206457 | -0,03727 | 0,114365 | 0,05136 |
| ***Prkcd*** | 0,074277 | 0,348892 | 0,246503 | 0,028518 | 0,008762 |
| ***Sphk1*** | 0,142443 | -0,11255 | 0,202839 | 0,136431 | 0,33292 |
| ***Hsd3b2*** | 0,486866 | 0,429578 | -0,0856 | -0,06416 | -0,07272 |
| ***Mpdu1*** | 0,263456 | 0,010299 | 0,124477 | 0,091813 | 0,198868 |
| ***Nfkb1*** | 0,158929 | 0,198966 | -0,05669 | 0,144947 | 0,239545 |
| ***Pard3*** | 0,265936 | 0,352518 | 0,030513 | 0,078601 | -0,05036 |
| ***Lpin1*** | 0,304783 | 0,830156 | 0,490199 | -0,31185 | -0,64224 |
| ***Slc1a3*** | 0,092452 | 0,317137 | 0,003587 | 0,099027 | 0,15749 |
| ***Hsd3b7*** | 0,077291 | 0,404465 | 0,271247 | -0,00801 | -0,07627 |
| ***Crem*** | 0,186205 | 0,357768 | 0,263242 | -0,0429 | -0,09653 |
| ***Ybx1*** | 0,145971 | 0,024808 | 0,161103 | 0,251183 | 0,080434 |
| ***Sp1*** | 0,094922 | 0,310516 | 0,068808 | 0,047906 | 0,139302 |
| ***Rela*** | 0,172055 | 0,224581 | 0,095794 | 0,07538 | 0,090153 |
| ***Cntf*** | 0,152345 | 0,005822 | 0,321655 | 0,103383 | 0,070471 |
| ***Fgf8*** | -0,10914 | 0,220107 | -0,01562 | 0,189983 | 0,361373 |
| ***Fshb*** | 0,2641 | -0,03187 | -0,13345 | 0,504294 | 0,038416 |
| ***Gnrh1*** | 0,198382 | -0,29813 | 0,317144 | 0,157381 | 0,256163 |
| ***Lhb*** | -0,04052 | 0,342593 | 0,237892 | -0,01577 | 0,103515 |
| ***Sptlc3*** | 0,14261 | 0,269279 | 0,199262 | -0,1559 | 0,172219 |
| ***Iqgap1*** | 0,010259 | 0,153842 | 0,037506 | 0,180653 | 0,244312 |
| ***Fyn*** | 0,033787 | 0,57696 | 0,230998 | -0,11383 | -0,11226 |
| ***Hint2*** | 0,431696 | 0,541988 | 0,088866 | -0,11277 | -0,33827 |
| ***Kcnj5*** | 0,120785 | 0,116729 | 0,324221 | -0,07598 | 0,121034 |
| ***Por*** | -0,22385 | 0,116112 | 0,419562 | 0,346398 | -0,05581 |
| ***Abca1*** | 0,121641 | 0,292213 | 0,159397 | 0,278491 | -0,26018 |
| ***Akr1c4*** | 0,140055 | 2,312705 | 0,392458 | -1,04147 | -1,21843 |
| ***Agpat5*** | 0,055188 | -0,25311 | 0,759662 | 0,242211 | -0,21914 |
| ***Pla2g10*** | -0,24089 | 0,31235 | 0,127281 | 0,119424 | 0,250417 |
| ***Dhrs11*** | 0,254664 | 0,101704 | 0,00178 | 0,097253 | 0,111486 |
| ***Dhh*** | -0,15911 | 0,163838 | -0,10189 | 0,305226 | 0,347331 |
| ***Lipc*** | -0,33561 | 0,056819 | 0,199002 | 0,359127 | 0,275048 |
| ***Vwf*** | -0,04866 | 0,054573 | 0,400477 | 0,043841 | 0,101266 |
| ***Sgpp1*** | 0,166081 | 0,115594 | 0,221875 | 0,071908 | -0,02587 |
| ***Cacna1h*** | -0,19318 | 0,348514 | 0,102798 | -0,01861 | 0,304007 |
| ***Gsx2*** | -0,31893 | 0,418733 | 0,087855 | 0,136392 | 0,216841 |
| ***Mcat*** | 0,160198 | 0,294337 | -0,05166 | 0,122705 | 0,014659 |
| ***Tnfrsf12a*** | 0,081349 | -0,04782 | 0,114627 | 0,093793 | 0,294744 |
| ***Hmox2*** | 0,173071 | 0,017732 | 0,196636 | 0,067259 | 0,077508 |
| ***Scd4*** | 0,10853 | -0,07341 | 0,032148 | 0,085914 | 0,375685 |
| ***Fdps*** | 0,05692 | 0,55864 | -0,1969 | 0,043325 | 0,059465 |
| ***Abhd5*** | -0,06357 | 0,116803 | 0,159989 | 0,048191 | 0,257255 |
| ***Pigy*** | 0,260532 | 0,06316 | 0,030858 | 0,086128 | 0,077989 |
| ***Pgap2*** | 0,093826 | 0,315424 | 0,015258 | -0,00073 | 0,092216 |
| ***Snca*** | -0,42627 | 0,151173 | 0,30492 | 0,166235 | 0,313848 |
| ***Acbd6*** | 0,119008 | 0,093755 | 0,280415 | 0,085779 | -0,07518 |
| ***Tbxas1*** | -0,00737 | 0,224823 | 0,283397 | -0,15924 | 0,16072 |
| ***Socs1*** | -0,02489 | 0,159075 | 0,008826 | -0,02151 | 0,377607 |
| ***Gpat4*** | 0,440138 | 0,220668 | -0,1771 | 0,012453 | -0,00366 |
| ***Scap*** | -0,00978 | 0,275538 | 0,275419 | 0,015266 | -0,06669 |
| ***Ins*** | -0,33103 | 0,458564 | 0,005813 | -0,03666 | 0,391596 |
| ***Cyp11b2*** | 0,076864 | 0,233931 | 0,212893 | -0,2635 | 0,227501 |
| ***Cd209d*** | 0,091387 | 0,313581 | 0,071771 | -0,02437 | 0,034695 |
| ***Smad2*** | -0,03336 | 0,546036 | -0,00388 | 0,027412 | -0,05172 |
| ***B3galt2*** | 0,497177 | 0,059779 | -0,05838 | -0,03372 | 0,019352 |
| ***Ucn2*** | -0,19689 | 0,0193 | -0,03087 | 0,323517 | 0,362181 |
| ***Pik3c2a*** | 0,005251 | -0,19112 | 0,220942 | 0,330514 | 0,110934 |
| ***Plcg1*** | 0,076618 | 0,17445 | 0,163278 | -0,05111 | 0,113243 |
| ***Bdkrb2*** | 0,019251 | 0,389705 | 0,067532 | 0,156606 | -0,16923 |
| ***Pla2g1b*** | 0,262145 | -0,0239 | -0,01338 | 0,010848 | 0,225401 |
| ***Il24*** | -0,42282 | 0,269726 | 0,391287 | 0,178939 | 0,038473 |
| ***Tomm22*** | 0,323483 | -0,01981 | 0,08704 | -0,0045 | 0,067942 |
| ***Csnk2b*** | 0,07672 | 0,050343 | 0,09571 | 0,184862 | 0,044891 |
| ***Igf1r*** | -0,0981 | -0,0826 | 0,279362 | 0,110573 | 0,242523 |
| ***Angptl4*** | 0,581837 | 0,508041 | -0,05698 | -0,22451 | -0,35668 |
| ***Tnfrsf1b*** | -0,22865 | 0,432734 | 0,091466 | 0,057953 | 0,088033 |
| ***Ncs1*** | 0,063109 | -0,09089 | 0,389705 | 0,028521 | 0,048726 |
| ***Nppa*** | 0,034337 | 0,318987 | 0,116168 | -0,02375 | -0,01468 |
| ***Adcyap1*** | -0,1195 | 0,290491 | -0,08756 | 0,037486 | 0,296229 |
| ***Fgfr4*** | -0,1769 | -0,4449 | 0,367973 | 0,487909 | 0,182102 |
| ***Cyp11a1*** | -0,05775 | 0,377209 | 0,045666 | -0,05022 | 0,096446 |
| ***Slc27a4*** | 0,152291 | -0,23916 | 0,166582 | 0,259891 | 0,071442 |
| ***Adipor1*** | 0,056755 | -0,02311 | 0,123791 | 0,204018 | 0,041937 |
| ***Ada*** | -0,15487 | 0,116629 | 0,033557 | 0,081439 | 0,323756 |
| ***Rest*** | 0,152846 | 0,340274 | 0,082494 | -0,02373 | -0,15445 |
| ***Ncoa6*** | 0,510063 | 0,045254 | -0,11355 | 0,07317 | -0,12752 |
| ***Acp6*** | 0,00336 | 0,217946 | 0,265117 | 0,028942 | -0,12906 |
| ***Foxo3*** | 0,150165 | 0,280371 | 0,170242 | -0,06662 | -0,14944 |
| ***Mtarc2*** | 0,046098 | 0,331057 | 0,329515 | -0,05253 | -0,27194 |
| ***Wnt1*** | 0,033599 | 0,388916 | 0,293452 | -0,19901 | -0,13614 |
| ***Rgr*** | -0,11017 | 0,139853 | 0,088715 | -0,03751 | 0,29793 |
| ***Atf6*** | 0,142743 | -0,04721 | 0,200829 | 0,112034 | -0,03686 |
| ***Diaph1*** | -0,11536 | -0,00192 | 0,120935 | 0,329384 | 0,03452 |
| ***Fcer1g*** | 0,466088 | -0,02297 | 0,085672 | -0,05944 | -0,11566 |
| ***Xbp1*** | 0,283165 | 0,006163 | 0,028796 | 0,000284 | 0,033335 |
| ***Nos2*** | 0,047766 | 0,196393 | 0,014087 | -0,16033 | 0,249916 |
| ***Map3k1*** | -0,22776 | 0,608811 | 0,12805 | -0,11516 | -0,05375 |
| ***Foxl2*** | -0,46668 | 0,205172 | 0,158218 | 0,054849 | 0,385626 |
| ***Sspn*** | -0,39404 | -0,1089 | 0,475158 | 0,230308 | 0,12567 |
| ***Sgpl1*** | -0,17167 | 0,147311 | 0,181794 | -0,02678 | 0,196809 |
| ***Slc1a1*** | -0,25939 | 0,162354 | 0,024637 | 0,197187 | 0,201651 |
| ***Gpx4*** | 0,43353 | -0,0627 | -0,1227 | 0,010968 | 0,065181 |
| ***Clu*** | 0,169921 | 0,158111 | -0,1104 | -0,01523 | 0,119884 |
| ***Il16*** | -0,39255 | 0,136877 | 0,20861 | 0,112644 | 0,249318 |
| ***Arf3*** | -0,08165 | -0,18093 | 0,101787 | 0,272885 | 0,198853 |
| ***Tfcp2l1*** | -0,08999 | 0,328663 | -0,0014 | -0,10216 | 0,174766 |
| ***Rgn*** | -0,25904 | 1,131845 | 0,357521 | -0,2515 | -0,68179 |
| ***Osbpl6*** | -0,09706 | 0,227485 | -0,0502 | -0,05824 | 0,258722 |
| ***Amh*** | -0,27951 | 0,015879 | 0,081219 | 0,133097 | 0,329991 |
| ***Pip4k2c*** | -0,0982 | -0,04323 | 0,172879 | 0,128695 | 0,116593 |
| ***Odc1*** | -0,02867 | -0,07395 | 0,034439 | 0,238911 | 0,101241 |
| ***Alg9*** | -0,10689 | 0,100631 | 0,200708 | 0,151393 | -0,08167 |
| ***Aloxe3*** | 0,071539 | -0,43518 | 0,537271 | 0,017422 | 0,068522 |
| ***Lpin2*** | 0,156967 | 0,32045 | -0,14057 | -0,04561 | -0,03667 |
| ***Slc27a2*** | 0,179649 | 1,385061 | -0,21278 | -0,39159 | -0,7137 |
| ***Dpm1*** | -0,0462 | -0,06814 | 0,365244 | 0,169208 | -0,17621 |
| ***Prpf19*** | 0,247509 | 0,053185 | 0,072431 | -0,08793 | -0,0434 |
| ***Dpm2*** | 0,087526 | -0,21279 | 0,11198 | 0,011425 | 0,238887 |
| ***Kcnk3*** | -0,00286 | 0,262584 | 0,04446 | 0,03308 | -0,10329 |
| ***Stat5b*** | -0,23896 | 0,314471 | 0,099072 | 0,045493 | 0,011286 |
| ***A3galt2*** | -0,23166 | 0,394605 | 0,134691 | 0,009511 | -0,07663 |
| ***A4galt*** | -0,17343 | 0,436676 | -0,03531 | -0,27341 | 0,275621 |
| ***Slc9a3r2*** | 0,275201 | 0,14752 | -0,19208 | -0,07088 | 0,061981 |
| ***Ghrl*** | -0,25736 | 0,238318 | 0,189848 | 0,054328 | -0,0071 |
| ***Elovl4*** | 0,354012 | -0,08627 | 0,221409 | -0,17037 | -0,10198 |
| ***Cln3*** | -0,23828 | -0,25091 | 0,383911 | 0,179902 | 0,135794 |
| ***Pi4ka*** | 0,227025 | -0,35982 | 0,24356 | 0,245809 | -0,15747 |
| ***Cry1*** | -0,28141 | 0,185524 | 0,210696 | 0,139586 | -0,05626 |
| ***Klf11*** | 0,263954 | 0,461579 | 0,044926 | -0,37353 | -0,20067 |
| ***Prxl2b*** | 0,527311 | 0,104619 | -0,21713 | -0,00462 | -0,21587 |
| ***Pla2g2f*** | -0,13056 | 0,010144 | 0,013834 | 0,346739 | -0,0459 |
| ***Ghr*** | 0,020345 | 0,862462 | -0,07016 | -0,35706 | -0,26179 |
| ***Creb3l3*** | 0,384907 | 0,539266 | -0,04744 | -0,33531 | -0,35437 |
| ***Ldlr*** | -0,19533 | -0,23531 | 0,05352 | 0,181182 | 0,380566 |
| ***Nfyb*** | -0,17682 | 0,471318 | 0,041167 | -0,18105 | 0,02928 |
| ***Hsd11b2*** | -0,26447 | 0,06894 | -0,03823 | 0,08626 | 0,330548 |
| ***Fads2*** | -0,2342 | 0,280332 | 0,08521 | 0,085893 | -0,03826 |
| ***Pyurf*** | 0,285114 | 0,25196 | -0,18195 | -0,1652 | -0,01464 |
| ***Arnt*** | -0,02327 | 0,187907 | -0,00335 | -0,04279 | 0,055936 |
| ***Slc27a5*** | -0,19069 | 0,503285 | 0,492936 | -0,04094 | -0,59322 |
| ***Nfkbia*** | 0,010711 | 0,285474 | -0,04494 | -0,06765 | -0,0162 |
| ***Abat*** | -0,04001 | 0,729514 | -0,084 | -0,23929 | -0,20142 |
| ***Crhr2*** | -0,209 | 0,272133 | 0,154132 | -0,12835 | 0,073522 |
| ***Inpp4b*** | 0,045988 | 0,339949 | -0,18891 | -0,01495 | -0,02138 |
| ***Tnf*** | -0,18177 | -0,4173 | 0,296697 | 0,27893 | 0,183334 |
| ***Apoa2*** | -0,17361 | 0,299471 | 0,622124 | -0,05897 | -0,53274 |
| ***Mc5r*** | -0,47844 | 0,146795 | 0,229753 | -0,15003 | 0,405924 |
| ***Stat5a*** | -0,24017 | 0,286443 | 0,032579 | -0,0361 | 0,110802 |
| ***Cd83*** | -0,12075 | 0,344129 | 0,193813 | -0,10301 | -0,16707 |
| ***Synj1*** | 0,053365 | -0,21435 | 0,100531 | 0,094958 | 0,112213 |
| ***Smpd2*** | 0,373988 | 0,183463 | -0,043 | -0,20745 | -0,16175 |
| ***Etnk1*** | 0,334836 | -0,58022 | 0,12858 | 0,18708 | 0,066287 |
| ***Cerk*** | 0,37678 | -0,40871 | 0,24643 | -0,13216 | 0,053874 |
| ***Ddhd1*** | -0,07266 | -0,28113 | 0,048918 | 0,233523 | 0,202526 |
| ***Ncam1*** | -0,11443 | 0,012297 | 0,347844 | 0,266199 | -0,3835 |
| ***Acadvl*** | 0,04424 | 0,257297 | 0,054568 | -0,1052 | -0,12314 |
| ***Gba*** | -0,08949 | -0,30691 | 0,002287 | 0,281243 | 0,239962 |
| ***Nr2c2*** | 0,307347 | 0,275021 | -0,17379 | -0,19836 | -0,08375 |
| ***Angpt2*** | -0,02909 | -0,22349 | 0,483676 | 0,187039 | -0,29222 |
| ***Hacd1*** | -0,08739 | -0,47534 | 0,207957 | 0,271649 | 0,208672 |
| ***Rxra*** | 0,193827 | 0,169862 | 0,015074 | 0,019906 | -0,27327 |
| ***Hacd3*** | -0,11534 | 0,128228 | 0,462576 | -0,04605 | -0,30471 |
| ***Scarb1*** | -0,17737 | 0,467088 | 0,007424 | 0,012039 | -0,19073 |
| ***Inppl1*** | 0,322216 | -0,24926 | -0,13938 | 0,17604 | 0,003393 |
| ***Fdx1*** | 0,246646 | 0,014324 | -0,06385 | -0,02432 | -0,06093 |
| ***Nr3c1*** | -0,18234 | 0,22316 | 0,147401 | 0,048842 | -0,12813 |
| ***Pigq*** | 0,025572 | -0,20899 | 0,027701 | 0,221844 | 0,042117 |
| ***Cyp2c70*** | -0,17508 | 0,358332 | -0,41738 | -0,00279 | 0,344775 |
| ***Ptpmt1*** | 0,332062 | -0,17379 | 0,146425 | 0,019344 | -0,22093 |
| ***Rasgrp4*** | 0,067573 | 0,250997 | 0,103654 | -0,35031 | 0,025117 |
| ***Caln1*** | 0,244506 | 0,18034 | -0,10949 | -0,29417 | 0,071705 |
| ***Ptk2b*** | 0,292783 | -0,40693 | 0,011903 | 0,069851 | 0,123411 |
| ***Pla2g6*** | 0,554899 | -0,63084 | 0,04968 | -0,0147 | 0,130395 |
| ***Ptpn11*** | 0,277848 | -0,11732 | -0,15029 | 0,091185 | -0,0196 |
| ***Tspo*** | 0,199444 | -0,10564 | -0,08693 | -0,0227 | 0,093259 |
| ***Pnpla2*** | 0,247679 | -0,29319 | 0,098474 | 0,152412 | -0,12929 |
| ***Kpnb1*** | -0,00156 | -0,1564 | -0,03799 | 0,218253 | 0,053538 |
| ***Acot8*** | 0,294234 | -0,20949 | 0,082232 | -0,05299 | -0,03962 |
| ***Ptges3*** | -0,09485 | -0,13093 | 0,394845 | 0,005238 | -0,10124 |
| ***Igfbp4*** | 0,023353 | 0,525892 | -0,55693 | 0,084373 | -0,00613 |
| ***Smpd1*** | 0,298504 | -0,25833 | -0,03771 | 0,003571 | 0,063222 |
| ***Txn*** | 0,059856 | -0,18666 | -0,03048 | 0,111522 | 0,10477 |
| ***Zbtb20*** | 0,497959 | -0,38159 | -0,05741 | -0,04936 | 0,038485 |
| ***Htt*** | -0,11077 | -0,14556 | 0,015363 | 0,065637 | 0,222904 |
| ***Acot7*** | 0,136012 | -0,45218 | -0,0005 | 0,096697 | 0,264942 |
| ***Cat*** | 0,079423 | 0,133675 | -0,05435 | -0,08199 | -0,03185 |
| ***Map3k5*** | 0,220329 | -0,46408 | -0,06448 | 0,353496 | -0,00104 |
| ***Sirt2*** | -0,10579 | -0,20416 | 0,125559 | 0,120428 | 0,103995 |
| ***Set*** | 0,114621 | -0,38183 | 0,134717 | 0,162169 | 0,001224 |
| ***Oprl1*** | -0,23733 | 0,35561 | -0,09612 | -0,05376 | 0,05949 |
| ***Tmem38b*** | -0,20979 | 0,279945 | 0,014409 | -0,04765 | -0,02129 |
| ***Ahr*** | -0,14658 | 0,313906 | -0,04025 | -0,03685 | -0,07687 |
| ***Atf2*** | 0,06989 | 0,196257 | -0,04385 | -0,06945 | -0,15106 |
| ***Pla2g4d*** | -0,36998 | 0,429116 | -0,0879 | -0,08084 | 0,100895 |
| ***Smad4*** | -0,10835 | 0,213073 | -0,02205 | -0,07522 | -0,02434 |
| ***Fads1*** | -0,21349 | 0,219678 | 0,020275 | -0,02971 | -0,01415 |
| ***Ctdnep1*** | 0,193788 | -0,33646 | -0,14355 | 0,264501 | 0,001115 |
| ***Msmo1*** | -0,14019 | -0,50058 | 0,219667 | 0,165779 | 0,220969 |
| ***Pitpnm2*** | 0,213124 | -0,27368 | 0,093895 | 0,028734 | -0,10102 |
| ***Rdh5*** | -0,31391 | 0,133303 | 0,132877 | 0,120966 | -0,1159 |
| ***Bmp4*** | 0,171514 | 0,081263 | -0,73047 | -0,11983 | 0,554589 |
| ***Wdtc1*** | 0,195379 | 0,166197 | -0,05373 | -0,10326 | -0,25719 |
| ***Pik3r3*** | 0,293126 | -0,01107 | -0,15181 | -0,15931 | -0,02902 |
| ***Sqle*** | -0,67397 | 0,014226 | 0,11547 | 0,248925 | 0,236359 |
| ***Smad3*** | 0,136784 | 0,106913 | -0,0102 | -0,24429 | -0,04875 |
| ***Eif6*** | 0,027371 | -0,26231 | -0,00343 | -0,01225 | 0,190126 |
| ***G6pd*** | -0,91356 | -0,81963 | 0,563423 | 0,760106 | 0,3419 |
| ***Sirt4*** | -0,21548 | 0,198685 | 0,044321 | -0,10078 | 0,000804 |
| ***Bscl2*** | 0,024949 | 0,242515 | -0,16808 | -0,0596 | -0,11227 |
| ***Gcdh*** | 0,101655 | 0,665216 | -0,28731 | -0,28521 | -0,27466 |
| ***Gba2*** | 0,300028 | -0,10983 | -0,25287 | 0,121265 | -0,13927 |
| ***Camp*** | -0,3413 | 0,170226 | 0,074399 | 0,066094 | -0,05043 |
| ***Chkb*** | 0,179283 | -0,05772 | 0,076193 | -0,03283 | -0,24861 |
| ***Cnksr1*** | -0,23223 | -0,22111 | 0,094262 | -0,07117 | 0,342324 |
| ***Ptafr*** | -0,43369 | 0,309562 | 0,048657 | -0,13854 | 0,121557 |
| ***Prkci*** | 0,024551 | -0,43889 | 0,186525 | 0,082533 | 0,04227 |
| ***Dennd1a*** | 0,030219 | -0,38118 | 0,088053 | 0,112109 | 0,025128 |
| ***Osbpl9*** | 0,069014 | -0,18285 | -0,00128 | 0,029076 | -0,04136 |
| ***Cdipt*** | 0,109616 | -0,26769 | -0,09877 | 0,049172 | 0,077938 |
| ***Srebf1*** | -0,75619 | 0,685746 | 0,064183 | -0,03825 | -0,08533 |
| ***Pemt*** | -0,23697 | 0,989664 | 0,319489 | -0,40521 | -0,8003 |
| ***Pias1*** | -0,01999 | -0,31838 | 0,043718 | 0,17472 | -0,01429 |
| ***Mtmr9*** | 0,184884 | -0,30989 | -0,11936 | 0,019357 | 0,090072 |
| ***Angptl3*** | -0,01735 | 0,479563 | 0,012199 | 0,059137 | -0,67252 |
| ***Bax*** | 0,015485 | -0,26442 | 0,05446 | -0,0615 | 0,110614 |
| ***Mapk14*** | 0,220987 | -0,26318 | 0,014724 | -0,02297 | -0,09842 |
| ***Sh3glb1*** | -0,09143 | -0,22929 | 0,044958 | 0,071863 | 0,051179 |
| ***Itgb1*** | -0,09358 | -0,30318 | 0,048568 | 0,112872 | 0,081351 |
| ***Pld1*** | -0,00404 | -0,54508 | -0,09043 | 0,187057 | 0,297768 |
| ***Eif4ebp2*** | 0,21679 | -0,38658 | -0,10803 | 0,188426 | -0,06668 |
| ***Cyp27b1*** | -0,68583 | 0,242121 | 0,083633 | 0,047049 | 0,156222 |
| ***Dolk*** | 0,10666 | -0,29264 | 0,238343 | -0,07462 | -0,13548 |
| ***St3gal2*** | -0,07541 | -0,2725 | -0,06813 | 0,137458 | 0,120375 |
| ***C3*** | -0,17852 | 8,03E-05 | -0,12049 | 0,058399 | 0,081334 |
| ***Vac14*** | 0,21733 | -0,5822 | 0,025768 | 0,127949 | 0,047837 |
| ***Fn1*** | -0,17803 | 0,115056 | -0,16879 | -0,00665 | 0,063373 |
| ***Dhrs7b*** | 0,209418 | -0,28588 | -0,0884 | -0,10934 | 0,092293 |
| ***Cnbp*** | -0,1978 | 0,164906 | 0,009302 | -0,14764 | -0,01498 |
| ***Ces1*** | -0,12385 | 0,340526 | 0,219336 | 0,011072 | -0,6343 |
| ***Orai1*** | -0,09673 | -0,61993 | 0,181397 | 0,12278 | 0,214529 |
| ***Pik3cd*** | -0,26532 | 0,047348 | 0,051176 | 0,075089 | -0,11023 |
| ***Apoa1*** | 0,200219 | -0,07687 | -0,25979 | -0,08649 | 0,018263 |
| ***Serpinc1*** | 0,000837 | 0,419675 | 0,144461 | -0,24383 | -0,53269 |
| ***Pla2g4f*** | -0,328 | -0,04694 | 0,123387 | -0,09503 | 0,132622 |
| ***Pon2*** | 0,295087 | -0,47237 | -0,19 | 0,01826 | 0,132092 |
| ***Samd8*** | -0,01179 | 0,122294 | -0,04145 | -0,06953 | -0,21956 |
| ***Pip4k2b*** | 0,071975 | -0,51034 | -0,06187 | 0,152456 | 0,126419 |
| ***Hnf1a*** | 0,141772 | -0,46934 | 0,133149 | 0,073791 | -0,10086 |
| ***Inha*** | -0,42801 | -0,00526 | 0,147948 | -0,0045 | 0,066486 |
| ***Atp8b1*** | -0,01227 | -0,40007 | -0,09746 | 0,211675 | 0,070155 |
| ***Rac1*** | 0,004156 | -0,36322 | 0,012891 | 0,016666 | 0,099646 |
| ***Ptger2*** | -0,09062 | -0,20236 | -0,19651 | 0,448995 | -0,19034 |
| ***Hpgds*** | -0,43071 | 0,138671 | 0,02627 | 0,073397 | -0,04638 |
| ***Hspa8*** | -0,01242 | -0,20995 | -0,02698 | -0,01897 | 0,028543 |
| ***Pdhb*** | 0,06376 | 0,009939 | 0,002782 | -0,12147 | -0,19501 |
| ***Pigf*** | 0,409506 | -0,57083 | 0,026605 | -0,09168 | -0,01541 |
| ***Nfe2l2*** | 0,237238 | 0,092282 | -0,35333 | -0,29459 | 0,071303 |
| ***Plscr1*** | 0,120276 | -0,77387 | -0,3144 | 0,258643 | 0,453488 |
| ***Ager*** | -0,37751 | 0,010039 | -0,07772 | -0,07408 | 0,26053 |
| ***Dbi*** | 0,109036 | 0,036403 | -0,24376 | -0,13536 | -0,03999 |
| ***Mtmr2*** | 0,082731 | 0,023319 | -0,09211 | -0,28693 | -0,00688 |
| ***Atp1a1*** | -0,15936 | -0,41783 | 0,023192 | 0,219062 | 0,054036 |
| ***Pex2*** | 0,068347 | -0,15174 | 0,080045 | -0,24601 | -0,03156 |
| ***Rhoa*** | -0,13676 | -0,1495 | 0,013621 | -0,03237 | 0,023586 |
| ***Grm5*** | -0,37597 | 0,062509 | -0,00667 | -0,19003 | 0,215252 |
| ***Bmx*** | -0,24461 | 0,124989 | -0,03119 | -0,09745 | -0,04732 |
| ***Ppt2*** | 0,170545 | -0,58907 | -0,04 | 0,130633 | 0,022986 |
| ***Plekha3*** | -0,12586 | -0,33085 | 0,044013 | 0,055548 | 0,047378 |
| ***Sptlc2*** | -0,31244 | -0,16422 | -0,25597 | 0,165366 | 0,255729 |
| ***Pik3r1*** | 0,090286 | -0,57779 | -0,2358 | 0,144529 | 0,263983 |
| ***Scd2*** | -0,43642 | -0,0554 | -0,01182 | -0,0192 | 0,20793 |
| ***Fgf7*** | -0,08165 | 0,136288 | -0,19965 | 0,163945 | -0,33884 |
| ***Gpd1*** | 0,25139 | 0,157219 | -0,22075 | -0,27529 | -0,23447 |
| ***Nsdhl*** | -0,15331 | -0,00855 | -0,30276 | -0,01815 | 0,15572 |
| ***Ppara*** | 0,284157 | 0,016818 | -0,01524 | -0,10652 | -0,51136 |
| ***Pigo*** | -0,12102 | -0,25631 | 0,03147 | 0,098042 | -0,09074 |
| ***Eef1a1*** | -0,04348 | -0,14783 | -0,05712 | -0,0585 | -0,03357 |
| ***Park7*** | 0,073974 | -0,24848 | -0,13259 | 0,041246 | -0,08749 |
| ***P2rx7*** | -0,39759 | -0,02834 | -0,0222 | 0,050312 | 0,038123 |
| ***Pi4k2b*** | 0,056801 | -0,08395 | 0,033039 | -0,11308 | -0,25691 |
| ***Pmvk*** | -0,38822 | 0,200157 | 0,036589 | -0,12994 | -0,08375 |
| ***Arf1*** | -0,02908 | -0,17576 | -0,04708 | -0,06413 | -0,05279 |
| ***Apoe*** | 0,00429 | 0,122289 | -0,03966 | -0,30261 | -0,15475 |
| ***Erlin2*** | -0,22985 | -0,51883 | 0,156051 | 0,027061 | 0,189336 |
| ***Tsc2*** | 0,232717 | -0,47205 | -0,12306 | 9,57E-05 | -0,01591 |
| ***Dab2*** | -0,15637 | 0,159993 | 0,122507 | -0,48975 | -0,01782 |
| ***Naa40*** | 0,166896 | -0,33088 | -0,10867 | -0,00717 | -0,10186 |
| ***Acsm1*** | -0,06478 | 1,698055 | 0,122459 | -1,0631 | -1,07973 |
| ***Phka1*** | 0,057963 | -0,55851 | 0,011748 | 0,053309 | 0,045559 |
| ***Mapk10*** | -0,29999 | 0,071825 | -0,06007 | -0,18061 | 0,078565 |
| ***Lss*** | -0,37367 | -0,13491 | 0,009673 | -0,05388 | 0,161206 |
| ***Me1*** | -0,27869 | 0,030381 | -0,09373 | -0,02535 | -0,02935 |
| ***Ptdss2*** | 0,170719 | -0,17374 | 0,05155 | -0,02325 | -0,42453 |
| ***B4galnt1*** | -0,22002 | -0,27144 | -0,01329 | 0,109422 | -0,00439 |
| ***Mbtps1*** | 0,062942 | 0,065451 | -0,41234 | -0,03739 | -0,08008 |
| ***Cd9*** | -0,24868 | 0,058871 | -0,30968 | 0,046527 | 0,047127 |
| ***Large1*** | 0,509803 | -1,06261 | -0,15702 | 0,061787 | 0,240405 |
| ***Inpp5k*** | -0,04817 | -0,26143 | 0,047599 | 0,083098 | -0,2323 |
| ***Alg12*** | 0,256034 | -0,30536 | -0,19279 | -0,09025 | -0,08126 |
| ***Pdia3*** | 0,289203 | -0,42388 | -0,27819 | 0,026608 | -0,02869 |
| ***Elovl5*** | -0,2241 | 0,445751 | -0,17497 | -0,27147 | -0,19038 |
| ***Pnpla8*** | -0,02232 | -0,0185 | 0,08681 | -0,16123 | -0,30148 |
| ***Pgd*** | -0,26509 | -0,22812 | 0,048566 | -0,02044 | 0,045067 |
| ***Mtmr14*** | -0,08559 | -0,05768 | -0,25696 | 0,03931 | -0,05986 |
| ***Dgkq*** | -0,08991 | -0,25209 | -0,00504 | 0,121109 | -0,19845 |
| ***Alg3*** | 0,083868 | -0,50885 | 0,029384 | 0,028316 | -0,06386 |
| ***Pde3b*** | -0,06062 | 0,05197 | -0,11567 | -0,03503 | -0,27351 |
| ***Adh1c*** | -0,16409 | 0,881367 | -0,32695 | -0,40502 | -0,41997 |
| ***Sgms2*** | -0,12935 | -0,39279 | -0,04223 | 0,094152 | 0,030405 |
| ***Ebp*** | -0,22105 | 0,471879 | -0,38572 | -0,19354 | -0,11227 |
| ***Decr2*** | -0,17105 | 0,40993 | -0,12647 | -0,32775 | -0,22645 |
| ***Slc27a1*** | -0,41268 | 0,148235 | 0,088034 | -0,06771 | -0,20017 |
| ***Nr0b2*** | -0,35622 | 0,233567 | 0,047325 | -0,39205 | 0,021575 |
| ***Gsta3*** | 0,082941 | 0,207171 | -0,25599 | -0,34971 | -0,13187 |
| ***Pik3r4*** | -0,00762 | 0,057253 | -0,08123 | -0,32561 | -0,09624 |
| ***Lclat1*** | -0,22461 | -0,0068 | -0,05603 | -0,08514 | -0,08289 |
| ***Acss2*** | -0,47749 | 0,754739 | -0,27084 | -0,25899 | -0,21873 |
| ***Me2*** | 0,17193 | 0,088743 | 0,326317 | -0,41003 | -0,64892 |
| ***Aldh3b1*** | -0,26231 | 0,03008 | -0,35622 | 0,084234 | 0,031547 |
| ***Acsm3*** | 0,104651 | 1,190503 | -0,61333 | -0,63344 | -0,52236 |
| ***Sphk2*** | 0,053698 | -0,31016 | -0,07302 | -0,01502 | -0,13099 |
| ***Cyp51a1*** | -0,32394 | -0,46856 | 0,011095 | 0,040245 | 0,26191 |
| ***Agpat3*** | 0,10976 | -0,3169 | -0,05055 | -0,06696 | -0,1553 |
| ***Hspa5*** | 0,034901 | -0,31971 | -0,09675 | -0,05292 | -0,06005 |
| ***Vtn*** | -0,07626 | 0,194188 | 0,082433 | -0,44226 | -0,25958 |
| ***Hsd17b13*** | -0,0717 | -0,33975 | -0,02248 | -0,04596 | -0,0319 |
| ***Insr*** | -0,04304 | -0,30847 | -0,01885 | -0,02239 | -0,12176 |
| ***Serinc1*** | -0,12528 | -0,06839 | -0,04171 | -0,19832 | -0,08303 |
| ***Sptlc1*** | -0,01337 | -0,45987 | 0,132835 | -0,10487 | -0,07282 |
| ***Mknk1*** | 0,178907 | -0,27213 | -0,1018 | -0,19094 | -0,13346 |
| ***Sorbs1*** | 0,139075 | -0,07047 | 0,079866 | -0,26281 | -0,40649 |
| ***Degs1*** | -0,12657 | -0,18738 | -0,01994 | -0,06146 | -0,13375 |
| ***Ptdss1*** | -0,24964 | -0,04251 | -0,22116 | -0,03904 | 0,019207 |
| ***Gstm4*** | -0,10794 | -0,03032 | 0,081005 | -0,06848 | -0,40839 |
| ***Hsd17b11*** | 0,108704 | -0,09837 | -0,07938 | -0,1876 | -0,28589 |
| ***Prkaa2*** | 0,026958 | -0,16209 | -0,16135 | -0,10442 | -0,16972 |
| ***Hmgcr*** | -1,05361 | 0,128838 | 0,194174 | -0,00519 | 0,16514 |
| ***Acox2*** | 0,207147 | -0,5143 | -0,00712 | -0,1187 | -0,13892 |
| ***Pten*** | -0,06088 | -0,2313 | -0,00484 | -0,14557 | -0,13593 |
| ***Hif1a*** | -0,06375 | -0,27724 | -0,25817 | 0,067145 | -0,05719 |
| ***Nfya*** | -0,18407 | -0,06289 | -0,25294 | -0,10811 | 0,011487 |
| ***Rdh7*** | -0,09397 | 0,72575 | -0,39211 | -0,43639 | -0,4015 |
| ***Ppcs*** | -0,06869 | -0,20115 | -0,10706 | -0,24008 | 0,015927 |
| ***Mvd*** | -0,48055 | -0,09876 | -0,14523 | -0,04437 | 0,167651 |
| ***Gpld1*** | -0,24967 | 0,463596 | -0,08726 | -0,36645 | -0,3653 |
| ***Osbp*** | 0,216893 | -0,62377 | -0,11025 | -0,01803 | -0,07237 |
| ***Akt1*** | -0,12742 | -0,47145 | -0,12531 | -0,0356 | 0,152021 |
| ***Gnpat*** | -0,1407 | -0,35624 | -0,0603 | 0,100031 | -0,15151 |
| ***Mtm1*** | -0,12409 | -0,12677 | 0,137794 | -0,1124 | -0,38462 |
| ***Sc5d*** | -0,31835 | 0,126571 | -0,17349 | -0,20497 | -0,04536 |
| ***Hnf4a*** | 0,050151 | -0,0984 | -0,05882 | -0,18117 | -0,33054 |
| ***Pdss1*** | -0,14239 | -0,66399 | 0,07768 | 0,134604 | -0,02571 |
| ***Hacd2*** | -0,20426 | -0,25938 | -0,05941 | -0,02494 | -0,08535 |
| ***Cers2*** | -0,2211 | -0,23697 | -0,0219 | -0,07556 | -0,08701 |
| ***Hsd11b1*** | -0,18894 | 0,270435 | -0,05613 | -0,22592 | -0,44326 |
| ***Hrh1*** | -0,34244 | -0,13853 | 0,104488 | -0,22612 | -0,04703 |
| ***Lpgat1*** | -0,05591 | -0,44222 | -0,20386 | -0,01972 | 0,067357 |
| ***Lbr*** | -0,33512 | 0,072834 | -0,30892 | -0,01907 | -0,07172 |
| ***Tnfrsf1a*** | -0,28457 | -0,31858 | 0,02452 | 0,128835 | -0,232 |
| ***Inpp5e*** | -0,16599 | -0,24439 | -0,10893 | -0,06506 | -0,09855 |
| ***Acly*** | -0,53178 | -0,15754 | -0,04428 | 0,10239 | -0,05604 |
| ***Dgat2*** | -0,08642 | 0,211404 | -0,25964 | -0,24013 | -0,31512 |
| ***Ocrl*** | -0,16671 | -0,35329 | -0,09241 | -0,11351 | 0,03534 |
| ***Acsl4*** | 0,022254 | -0,66087 | -0,26081 | -0,00454 | 0,213346 |
| ***Stard3*** | -0,19645 | -0,28329 | -0,0961 | -0,05702 | -0,06698 |
| ***Plekha8*** | -0,18055 | -0,1673 | -0,20704 | 0,063617 | -0,20944 |
| ***Alg2*** | -0,07839 | -0,14061 | -0,17591 | -0,11553 | -0,19528 |
| ***Med1*** | 0,053763 | -0,41944 | -0,10408 | -0,04778 | -0,19516 |
| ***Ppat*** | -0,0756 | -0,1928 | -0,38376 | -0,0108 | -0,05405 |
| ***Dlat*** | -0,3087 | -0,2988 | -0,04747 | -0,01842 | -0,04631 |
| ***Myd88*** | 0,190685 | -0,79805 | -0,19427 | 0,007765 | 0,069196 |
| ***Pfkfb2*** | 0,057992 | -0,35214 | -0,31403 | 0,03468 | -0,15137 |
| ***Rab27a*** | -0,19029 | 0,240772 | -0,39714 | -0,21802 | -0,16054 |
| ***Selenoi*** | -0,20959 | -0,18168 | -0,13933 | -0,08984 | -0,10495 |
| ***Mtmr4*** | 0,293347 | -0,76603 | -0,10641 | -0,1422 | -0,00706 |
| ***Pkp2*** | 0,065687 | -0,34501 | -0,15107 | -0,25019 | -0,04859 |
| ***Awat1*** | 0,015113 | -0,12656 | -0,21645 | -0,46849 | 0,065396 |
| ***Gpat3*** | -0,40771 | -0,14956 | -0,19328 | -0,03526 | 0,053116 |
| ***Mtmr7*** | -0,12012 | -0,38142 | -0,11513 | 0,008095 | -0,12729 |
| ***Jak2*** | -0,02781 | -0,30249 | -0,25278 | 0,041496 | -0,19887 |
| ***Amacr*** | 0,266114 | 0,465913 | -0,78026 | -0,35673 | -0,34628 |
| ***Plg*** | -0,03442 | 0,501651 | -0,11731 | -0,58013 | -0,52523 |
| ***Scd*** | -0,13432 | 0,289914 | 0,097704 | -0,38032 | -0,63388 |
| ***Alg14*** | -0,06775 | -0,52556 | 0,008195 | -0,04816 | -0,13237 |
| ***Plcb1*** | 0,41932 | -0,57034 | -0,37457 | -0,19463 | -0,05126 |
| ***Sec14l2*** | 0,01917 | 0,028963 | -0,1995 | -0,16686 | -0,45464 |
| ***Mapk3*** | -0,12178 | -0,2083 | -0,11816 | -0,24199 | -0,08334 |
| ***Rgs3*** | -0,48555 | -0,11677 | 0,027202 | -0,08414 | -0,11899 |
| ***Crls1*** | -0,56569 | 0,193631 | -0,05049 | -0,11349 | -0,24249 |
| ***Arv1*** | -0,01046 | -0,2308 | -0,34189 | -0,03724 | -0,161 |
| ***Elovl1*** | -0,08439 | -0,74338 | -0,09103 | 0,023073 | 0,096609 |
| ***Rab4a*** | 0,156441 | -0,64076 | -0,02065 | 0,037615 | -0,33265 |
| ***Erlin1*** | 0,064623 | -0,30683 | -0,07959 | -0,29632 | -0,18502 |
| ***Cyp2c9*** | -0,67462 | 0,257784 | 0,167694 | -0,2441 | -0,31643 |
| ***Mgat2*** | -0,02621 | -0,42459 | -0,17964 | -0,11328 | -0,06805 |
| ***Gal3st1*** | 0,031791 | 0,169543 | -0,41082 | -0,27603 | -0,33125 |
| ***Babam2*** | -0,104 | -0,36585 | -0,05374 | -0,19604 | -0,10763 |
| ***Plce1*** | -0,04291 | -0,48522 | -0,17329 | 0,048268 | -0,17428 |
| ***Cga*** | -0,51409 | -0,06749 | -0,12433 | -0,08328 | -0,04048 |
| ***Decr1*** | 0,10697 | 0,084374 | -0,32973 | -0,45541 | -0,24776 |
| ***Ccl2*** | 0,066708 | -1,28911 | -0,39182 | 0,091752 | 0,678362 |
| ***Npc2*** | -0,37777 | -0,36177 | -0,13016 | -0,07041 | 0,090643 |
| ***Stim1*** | 0,180011 | -0,43319 | -0,24302 | -0,24569 | -0,11133 |
| ***Pigg*** | -0,28223 | -0,2663 | 0,027103 | -0,06674 | -0,2713 |
| ***Fig4*** | -0,02555 | -0,5778 | -0,14152 | -0,08147 | -0,03368 |
| ***Neu3*** | -0,24807 | -0,10517 | -0,3995 | -0,16853 | 0,059742 |
| ***Plpp6*** | -0,24057 | -0,29598 | -0,15455 | -0,10519 | -0,07508 |
| ***Npnt*** | 0,03637 | 0,027742 | -0,07541 | -0,23147 | -0,63056 |
| ***Dhcr7*** | -0,22865 | -0,15788 | -0,20414 | -0,13482 | -0,15003 |
| ***Cyp26a1*** | -0,88258 | 0,748972 | -0,40208 | -0,30796 | -0,03217 |
| ***Nrg4*** | -0,42351 | -0,22017 | -0,03922 | -0,15685 | -0,03866 |
| ***Cd82*** | -0,26025 | -0,49219 | -0,00367 | -0,10458 | -0,02133 |
| ***Pctp*** | -0,4482 | -0,24054 | 0,059516 | -0,06193 | -0,19305 |
| ***Esrrg*** | -0,54109 | 0,285128 | -0,14111 | -0,28518 | -0,20333 |
| ***Mapk9*** | -0,00623 | -0,34585 | -0,12821 | -0,25198 | -0,15795 |
| ***Faah*** | 0,061569 | 0,020827 | -0,45332 | -0,27998 | -0,24073 |
| ***Cyp2r1*** | 0,002848 | -0,22624 | -0,48449 | 0,007789 | -0,19752 |
| ***Pdha1*** | -0,04335 | -0,37375 | -0,31533 | -0,27336 | 0,103624 |
| ***Cnep1r1*** | -0,21241 | -0,17938 | -0,14926 | -0,2981 | -0,07127 |
| ***Phb*** | -0,13907 | -0,44114 | -0,09516 | -0,16773 | -0,0692 |
| ***Nr1h3*** | 0,045567 | -0,18155 | -0,28072 | -0,24717 | -0,25165 |
| ***Plin2*** | 0,099249 | -0,88332 | -0,13492 | -0,13418 | 0,130005 |
| ***Slc45a3*** | -0,26267 | 0,091107 | -0,03347 | -0,27401 | -0,44452 |
| ***Acadl*** | -0,20704 | -0,55124 | -0,02583 | -0,07497 | -0,06891 |
| ***Pklr*** | -0,19806 | -0,27517 | -0,31287 | -0,11076 | -0,03737 |
| ***Bco1*** | -0,40829 | -1,00127 | 0,010991 | 0,36092 | 0,102706 |
| ***Pdk4*** | -0,7756 | 0,003353 | 0,254408 | -0,1653 | -0,2616 |
| ***Etnppl*** | 0,053914 | 0,337004 | 0,168883 | -0,42296 | -1,08339 |
| ***Alg8*** | 0,229921 | -0,85438 | -0,35375 | 0,092532 | -0,06547 |
| ***Pip5k1c*** | -0,21435 | -0,45938 | -0,21608 | -0,03237 | -0,03858 |
| ***Adipor2*** | -0,11821 | 0,070816 | -0,11016 | -0,35886 | -0,44666 |
| ***Ceacam1*** | -0,1119 | 0,280183 | -0,65997 | -0,21636 | -0,25564 |
| ***Mttp*** | -0,05097 | -0,37223 | -0,1664 | -0,00399 | -0,37249 |
| ***Cds2*** | -0,39322 | -0,22957 | -0,07803 | -0,19952 | -0,07235 |
| ***Abhd3*** | -0,17661 | 0,683633 | -0,5163 | -0,42886 | -0,55082 |
| ***Prkd2*** | -0,29435 | -0,28173 | -0,18867 | -0,17459 | -0,0635 |
| ***Taz*** | 0,05904 | -0,26099 | -0,11091 | -0,34407 | -0,34664 |
| ***Mtor*** | 0,012855 | -0,15939 | -0,11537 | -0,44566 | -0,29787 |
| ***Idh1*** | -0,07333 | -0,07805 | -0,38205 | -0,35781 | -0,13272 |
| ***Pex7*** | -0,17079 | -0,01757 | -0,31339 | -0,32498 | -0,20551 |
| ***Dab1*** | 0,021078 | 0,084041 | -0,29917 | -0,47563 | -0,36587 |
| ***Mtmr12*** | -0,18235 | -0,27993 | -0,33231 | -0,13219 | -0,1118 |
| ***Hsd17b4*** | 0,162203 | -0,53786 | -0,16633 | -0,25361 | -0,24445 |
| ***Crebl2*** | 0,022431 | -0,59029 | -0,37768 | -0,12828 | 0,033598 |
| ***Tirap*** | -0,15604 | -0,2766 | -0,19457 | -0,31248 | -0,10088 |
| ***Pisd*** | -0,11942 | -0,36406 | -0,26575 | -0,18759 | -0,11967 |
| ***Adrb2*** | -0,01087 | -0,54006 | -0,22739 | -0,07428 | -0,2077 |
| ***Abcg5*** | -0,60954 | 0,480023 | 0,03509 | -0,40794 | -0,56029 |
| ***Grb14*** | 0,12025 | -0,74204 | -0,46291 | -0,0726 | 0,074658 |
| ***Oxsm*** | -0,11358 | -0,3831 | -0,20943 | -0,24405 | -0,13282 |
| ***Il33*** | -0,29263 | 0,166507 | -0,72088 | -0,42045 | 0,177044 |
| ***Erbb2*** | -0,10815 | -0,01312 | -0,46978 | -0,24009 | -0,26202 |
| ***Dhdds*** | -0,05602 | -0,58557 | -0,14279 | -0,21557 | -0,10496 |
| ***Fdft1*** | -0,46574 | -0,23617 | -0,21606 | -0,15921 | -0,03858 |
| ***Kras*** | -0,26815 | -0,15401 | -0,19245 | -0,26561 | -0,23842 |
| ***Abcb11*** | -0,28694 | 0,895383 | -0,1812 | -0,56721 | -0,9809 |
| ***Fabp5*** | -0,71247 | 0,616043 | -0,73515 | -0,22654 | -0,06958 |
| ***Asah2*** | -0,41224 | 0,042507 | -0,32461 | -0,27624 | -0,16936 |
| ***Pik3c2g*** | -0,48782 | -0,08919 | -0,10013 | -0,11043 | -0,35399 |
| ***Fgf1*** | -0,08053 | 0,240161 | -0,10593 | -0,55711 | -0,6425 |
| ***Srebf2*** | -0,43648 | -0,59157 | -0,18447 | -0,00592 | 0,067099 |
| ***Hsd17b7*** | -0,3581 | -0,62489 | -0,219 | 0,014853 | 0,034697 |
| ***Prkab1*** | -0,21304 | 0,004241 | -0,24166 | -0,39474 | -0,30941 |
| ***Stard7*** | -0,00115 | -0,44108 | -0,14181 | -0,20581 | -0,36728 |
| ***Pigp*** | -0,33646 | -0,09706 | -0,14081 | -0,34328 | -0,25493 |
| ***Thrb*** | 0,064914 | -0,58191 | -0,25835 | -0,15244 | -0,24924 |
| ***Aldh1a1*** | 0,149176 | -0,68979 | -0,46523 | -0,18232 | 0,010048 |
| ***Tecr*** | -0,36624 | 0,124547 | -0,49878 | -0,24254 | -0,2026 |
| ***Aldh1a7*** | 0,005718 | -0,78892 | -0,30654 | -0,03241 | -0,06392 |
| ***Pigl*** | -0,33922 | -0,73969 | -0,24621 | 0,127739 | 0,000332 |
| ***Elovl7*** | -0,13624 | -0,75725 | -0,20544 | 0,083267 | -0,19549 |
| ***St3gal4*** | -0,06625 | -0,21452 | -0,5941 | -0,23928 | -0,10367 |
| ***Impa1*** | -0,06924 | -0,89226 | -0,12935 | -0,05178 | -0,07605 |
| ***Pigu*** | -0,16095 | -0,81583 | -0,144 | -0,01759 | -0,10569 |
| ***Sigirr*** | -0,3199 | -0,15088 | -0,57206 | -0,1305 | -0,07765 |
| ***Rdh10*** | -0,35614 | -0,46279 | 0,167625 | -0,14453 | -0,46173 |
| ***Tlr2*** | -0,22506 | -0,32334 | -0,54692 | -0,18222 | 0,019467 |
| ***Acer2*** | -0,3638 | -0,05134 | -0,28168 | -0,34721 | -0,22747 |
| ***Gpd1l*** | -0,24473 | -0,43758 | -0,11251 | -0,20277 | -0,28271 |
| ***Cept1*** | -0,42838 | -0,33095 | -0,21794 | -0,1799 | -0,12701 |
| ***Agk*** | -0,24584 | -0,41972 | -0,17823 | -0,2621 | -0,1825 |
| ***Klf5*** | 0,083686 | -1,62188 | -0,12032 | 0,342979 | 0,021565 |
| ***Pik3cb*** | -0,21367 | -0,8372 | -0,21036 | -0,02392 | -0,02048 |
| ***Smpd4*** | -0,14623 | -0,55605 | -0,04851 | -0,37469 | -0,18605 |
| ***Elovl2*** | -0,1327 | 0,596818 | -0,41057 | -0,5967 | -0,77845 |
| ***Dpagt1*** | -0,19469 | -0,57393 | -0,21439 | -0,15736 | -0,19059 |
| ***Cert1*** | -0,42481 | -0,43365 | -0,14275 | -0,21424 | -0,1175 |
| ***Pld2*** | -0,14237 | -0,53318 | -0,49428 | -0,16364 | -0,00071 |
| ***Npc1*** | -0,24579 | 0,014381 | -0,29323 | -0,412 | -0,40126 |
| ***Slc44a3*** | -0,0732 | -0,90911 | -0,37755 | 0,006955 | 0,010962 |
| ***Rarres2*** | -0,44404 | 0,208827 | -0,2729 | -0,33708 | -0,50316 |
| ***Xdh*** | -0,18054 | -0,28762 | -0,22192 | -0,2607 | -0,40273 |
| ***Inhba*** | -0,37941 | -0,2869 | -0,73343 | -0,23338 | 0,275028 |
| ***Acsl5*** | -0,30028 | -0,43237 | -0,34635 | -0,18066 | -0,10034 |
| ***Plpp2*** | -0,44512 | -0,80011 | -0,26111 | -0,03867 | 0,181912 |
| ***Aspa*** | -0,15206 | 0,33452 | -0,71284 | -0,415 | -0,42564 |
| ***Pde8a*** | -0,15892 | -0,55505 | -0,20848 | -0,11567 | -0,37352 |
| ***Fgfr3*** | -0,23208 | -0,26151 | -0,22428 | -0,28027 | -0,42774 |
| ***Il1rn*** | -0,75085 | 0,012076 | -0,49055 | 0,042444 | -0,23981 |
| ***Gpr39*** | 0,045331 | -0,689 | -0,31633 | -0,28969 | -0,19346 |
| ***Pon3*** | -0,08 | -0,52909 | -0,44654 | -0,28606 | -0,10196 |
| ***Sult1e1*** | -0,77274 | 0,088906 | 0,193589 | -0,32624 | -0,62783 |
| ***Ppt1*** | -0,28164 | -0,81679 | -0,18339 | -0,17066 | 0,002255 |
| ***Agpat2*** | -0,03707 | -0,32925 | -0,47236 | -0,26184 | -0,35562 |
| ***Idi1*** | -0,30347 | -1,16207 | -0,29405 | 0,118936 | 0,18407 |
| ***Acat1*** | 0,022855 | 0,265225 | -0,44318 | -0,69614 | -0,61754 |
| ***Agt*** | -0,32361 | 0,085077 | -0,52515 | -0,41596 | -0,29566 |
| ***Hmgcs1*** | -0,42746 | -0,5808 | -0,25948 | -0,22627 | 0,013754 |
| ***Foxa1*** | 0,558808 | -0,8413 | -0,40635 | -0,48428 | -0,32452 |
| ***Tcf7l2*** | -0,31506 | -0,7708 | -0,1655 | -0,15239 | -0,09592 |
| ***Soat2*** | -0,2558 | -0,20424 | -0,22728 | -0,25872 | -0,55695 |
| ***Apoa4*** | -0,46224 | -0,92768 | -0,32289 | -0,02632 | 0,2323 |
| ***Pigb*** | -0,10892 | -0,87659 | -0,44137 | -0,05521 | -0,02934 |
| ***Il18*** | -0,15491 | 0,211891 | -0,75962 | -0,60076 | -0,21168 |
| ***Sgms1*** | -0,26215 | -0,65755 | -0,17451 | -0,26171 | -0,16705 |
| ***Tp53*** | -0,03313 | -0,694 | -0,51593 | -0,13647 | -0,15904 |
| ***Prkdc*** | -0,00705 | -0,58037 | -0,49181 | -0,35542 | -0,12027 |
| ***Slc44a1*** | -0,49117 | -0,23034 | -0,42417 | -0,14217 | -0,26827 |
| ***Plpp1*** | -0,4651 | -0,47728 | -0,39279 | -0,13063 | -0,11282 |
| ***Kdsr*** | -0,20155 | -0,67888 | -0,24928 | -0,3033 | -0,15867 |
| ***Rufy1*** | -0,09461 | -0,72914 | -0,21957 | -0,28368 | -0,26679 |
| ***Pcyt1a*** | -0,15554 | -0,76428 | -0,24717 | -0,25354 | -0,17347 |
| ***Il15*** | -0,59553 | -0,11774 | -0,45572 | -0,1838 | -0,26009 |
| ***Fasn*** | -0,78593 | -0,47232 | -0,08825 | 0,042331 | -0,31419 |
| ***Gpam*** | -0,22696 | -0,85832 | -0,13764 | -0,24223 | -0,15404 |
| ***Rdh16*** | -1,11795 | 0,483381 | -0,21248 | -0,30865 | -0,46387 |
| ***Nr5a2*** | -0,20778 | -0,56672 | -0,47756 | -0,20032 | -0,18869 |
| ***Sh3yl1*** | -0,75787 | -0,12231 | -0,12471 | -0,36881 | -0,28055 |
| ***Pgap1*** | -0,15334 | -0,35012 | -0,44428 | -0,21335 | -0,49721 |
| ***Esr1*** | -0,8671 | 0,042478 | -0,57455 | -0,17213 | -0,09752 |
| ***Anpep*** | -0,12711 | 0,352258 | -1,03752 | -0,40898 | -0,44873 |
| ***Serinc5*** | -0,26967 | -0,58329 | -0,3214 | -0,44021 | -0,06622 |
| ***Acat2*** | -0,12932 | -0,64627 | -0,3273 | -0,35617 | -0,22326 |
| ***Plpp3*** | -0,29058 | -0,36186 | -0,48651 | -0,36117 | -0,18591 |
| ***Kng1*** | -0,41652 | -0,33195 | -0,41554 | -0,32952 | -0,19744 |
| ***Akr1b1*** | 0,044134 | -0,50866 | -0,45581 | -0,40216 | -0,37285 |
| ***Camkk2*** | -0,20439 | -1,09832 | -0,43801 | 0,090404 | -0,07779 |
| ***Napepld*** | -0,19458 | -1,27637 | -0,57216 | 0,121144 | 0,160326 |
| ***Bcl2*** | -0,06053 | -0,19298 | -1,1254 | -0,45546 | 0,040528 |
| ***Casp8*** | 0,183469 | -0,91967 | -0,74514 | -0,21352 | -0,13342 |
| ***St3gal5*** | -0,26422 | -0,5203 | -0,27124 | -0,28675 | -0,48801 |
| ***Nr1h4*** | 0,255714 | -0,60739 | -0,50502 | -0,55488 | -0,43199 |
| ***S1pr2*** | -0,50301 | -0,74844 | -0,42161 | -0,18954 | 0,015191 |
| ***Alg6*** | -0,28685 | -0,50994 | -0,37891 | -0,26049 | -0,42764 |
| ***Cxcl12*** | -0,31424 | 0,331944 | -0,47419 | -0,75144 | -0,67271 |
| ***Aldh3a2*** | -0,07275 | -0,76701 | -0,39468 | -0,41599 | -0,25463 |
| ***Tlr3*** | -0,50991 | -0,77709 | -0,64884 | -0,13957 | 0,162816 |
| ***Acer3*** | -0,57307 | -0,42693 | -0,27763 | -0,31425 | -0,3316 |
| ***Asah1*** | -0,38117 | -1,25264 | -0,40556 | -0,00066 | 0,101994 |
| ***Hpgd*** | -0,56275 | -0,65989 | 0,03402 | -0,31819 | -0,44264 |
| ***Prkag2*** | -0,50905 | -0,84728 | -0,13855 | -0,19606 | -0,26978 |
| ***Nr2f2*** | -0,02633 | -0,4037 | -0,5152 | -0,52636 | -0,50426 |
| ***Slc44a2*** | -0,20286 | -0,15864 | -0,70526 | -0,58495 | -0,37479 |
| ***Smpd3*** | -0,30263 | -1,14887 | -0,67039 | -0,07308 | 0,162832 |
| ***Arntl*** | -0,28962 | -1,1286 | -0,28489 | -0,08829 | -0,24788 |
| ***Apom*** | -0,33283 | -0,1507 | -0,93804 | -0,45161 | -0,17364 |
| ***Plaat3*** | -0,25542 | -0,37084 | -0,45793 | -0,60211 | -0,38193 |
| ***Agmo*** | -0,48299 | 0,599428 | -0,96118 | -0,65029 | -0,58035 |
| ***Pon1*** | -0,20672 | 0,160498 | 0,102142 | -0,76871 | -1,36881 |
| ***Dpp4*** | -0,50342 | -0,23887 | -0,52092 | -0,48077 | -0,34043 |
| ***Fas*** | -0,26124 | -0,37775 | -0,62657 | -0,563 | -0,29446 |
| ***Nr1i3*** | -0,76069 | 0,127178 | -0,24139 | -0,64651 | -0,60801 |
| ***Itga3*** | -0,23658 | -1,51486 | -0,41804 | -0,12034 | 0,087227 |
| ***Rarb*** | -0,53431 | -0,65308 | -0,60431 | -0,37019 | -0,04105 |
| ***Cadm1*** | -0,42542 | -0,78384 | -0,22827 | -0,38722 | -0,38498 |
| ***Aldh8a1*** | -0,129 | 0,635762 | -0,37848 | -0,93457 | -1,40599 |
| ***Itga6*** | -0,97647 | -0,70438 | -0,40466 | -0,08911 | -0,08462 |
| ***Nrg1*** | 0,098486 | -1,20854 | -0,67924 | -0,44801 | -0,11436 |
| ***App*** | -0,64282 | -0,94085 | -0,48604 | -0,276 | -0,02858 |
| ***Dgka*** | -0,29734 | -0,07124 | -1,07226 | -0,52443 | -0,40956 |
| ***Pltp*** | -0,54254 | -0,02021 | -0,53789 | -0,73388 | -0,55614 |
| ***Avpr1a*** | -0,95307 | 0,110293 | -0,10142 | -0,48861 | -1,0545 |
| ***Bhmt*** | -0,14246 | 1,050284 | -1,5266 | -1,09965 | -0,7783 |
| ***Homer2*** | -0,59846 | -0,40744 | -0,69071 | -0,45056 | -0,35904 |
| ***Dhcr24*** | -0,3609 | -1,20499 | -0,57054 | -0,21409 | -0,17061 |
| ***Osbpl3*** | -0,08443 | -0,97686 | -0,89314 | -0,60889 | -0,01109 |
| ***Baat*** | -0,02758 | -0,14993 | -0,88211 | -0,74723 | -0,82395 |
| ***Prkd3*** | -0,08954 | -0,27329 | -0,74934 | -0,84385 | -0,69256 |
| ***Tnfsf10*** | -0,76842 | -0,42081 | -0,89224 | -0,39771 | -0,27281 |
| ***Ppargc1a*** | -0,79412 | -0,38501 | -0,49487 | -0,42794 | -0,66493 |
| ***Mfsd2a*** | -0,98722 | -0,83224 | -0,51057 | -0,13327 | -0,3093 |
| ***Pparg*** | 0,308282 | -1,26225 | -0,77772 | -0,62831 | -0,46886 |
| ***Slc6a6*** | -0,32552 | -0,82539 | -0,56231 | -0,87239 | -0,33093 |
| ***Agtr1*** | -0,49251 | -0,62224 | -0,73842 | -0,69699 | -0,43405 |
| ***Pnpla3*** | -1,66471 | -0,09853 | -0,52087 | -0,51328 | -0,23482 |
| ***Grb10*** | -0,41701 | -0,3467 | -0,27657 | -0,92674 | -1,09772 |
| ***Cyp7a1*** | -0,10229 | 1,351961 | -0,28199 | -1,67746 | -2,37028 |
| ***Acaca*** | -0,81725 | -0,50812 | -0,39237 | -0,72828 | -0,71412 |
| ***Angpt1*** | -0,11474 | -0,16376 | 0,009827 | -1,22848 | -1,71706 |
| ***Akr1c20*** | -0,28915 | 0,839712 | -1,67866 | -1,3019 | -0,96607 |
| ***Far1*** | -0,10446 | -1,62185 | -0,54734 | -0,69251 | -0,43154 |
| ***Prkca*** | -0,78907 | -1,45442 | -0,55646 | -0,30357 | -0,30056 |
| ***Mgst3*** | -0,9771 | -0,44419 | -1,12997 | -0,54633 | -0,3664 |
| ***G6pc*** | -0,13059 | 0,340476 | -0,01617 | -1,58284 | -2,08482 |
| ***Cyp4a11*** | -0,5732 | 0,642984 | -0,96937 | -1,57802 | -1,1825 |
| ***Rbp1*** | -0,73896 | -0,81934 | -1,09913 | -0,56668 | -0,49116 |
| ***Srd5a2*** | -0,44862 | -1,36603 | -0,96258 | -0,54396 | -0,40065 |
| ***Plscr2*** | -0,63206 | -1,50198 | -0,64968 | -0,46542 | -0,50527 |
| ***Elovl6*** | -1,0008 | -1,48377 | -0,7571 | -0,17717 | -0,38 |
| ***Ptgds*** | -1,88192 | -0,85589 | -0,79971 | -0,40544 | -0,41432 |
| ***Csf1*** | -0,26315 | -1,99638 | -1,44569 | -0,86568 | -0,10769 |
| ***Cers6*** | -2,07437 | -1,2178 | -0,63313 | -0,40597 | -0,38715 |
| ***Cyp39a1*** | -1,16036 | -1,21124 | -1,23069 | -0,78684 | -0,36593 |
| ***Hexb*** | -1,93124 | -1,05373 | -0,57878 | -0,6661 | -0,6034 |
| ***Abcb1b*** | -2,13704 | -1,12408 | -0,84069 | -0,67715 | -0,42022 |
| ***Akr1d1*** | -0,74179 | 0,643042 | -0,61832 | -1,92256 | -2,62267 |
| ***Akr1c3*** | -0,27282 | -0,12655 | -2,54033 | -1,89342 | -0,62448 |
| ***Abcc4*** | -0,77985 | -1,81574 | -1,03435 | -0,8991 | -1,03666 |
| ***Lepr*** | -1,18575 | -0,92697 | -0,63828 | -1,08262 | -1,81091 |
| ***Cyp17a1*** | -3,07659 | -1,66558 | 0,31134 | 0,14851 | -1,75611 |
| ***Ar*** | -1,20432 | -1,20092 | -1,31942 | -1,29383 | -1,37866 |
| ***Cyp4a14*** | -0,75272 | -0,24857 | -1,13345 | -2,35025 | -2,68532 |
| ***Abcb1*** | -3,15988 | -0,99045 | -1,36827 | -1,08103 | -0,80522 |
| ***Prlr*** | -1,84625 | -0,84082 | -1,60129 | -1,87303 | -1,77616 |
| ***Cd36*** | -1,76494 | -1,54792 | -2,50848 | -1,88358 | -1,04423 |
| ***Cyp2c8*** | -3,0275 | -2,40431 | -1,64767 | -1,56727 | -2,10398 |
| ***Fmo3*** | -5,79658 | -3,84383 | -4,39118 | -4,23965 | -3,20229 |

**Table S16:** Activation Z-score of genes involved in the transport of lipids male vs. female. Activation Z-score was calculated with IPA software from Qiagen.

| **Pathway Summary** | -1,974 | -0,945 | -2,243 | -2,754 | -2,405 |
| --- | --- | --- | --- | --- | --- |
| **genes in the transport of lipid network** | **0 h** | **24 h** | **48 h** | **72 h** | **96 h** |
| ***Slco1a1*** | 3,103735 | 4,931852 | 3,018541 | 2,176901 | 1,075615 |
| ***Saa1*** | -0,22883 | 0,807029 | 1,679516 | 1,794978 | 0,628602 |
| ***Scp2*** | 0,905224 | 2,031537 | 0,985545 | 0,350656 | 0,253287 |
| ***F2r*** | 1,338302 | 0,862247 | 0,770082 | 0,465858 | 0,599782 |
| ***Gdf15*** | 0,203624 | 1,295703 | 0,569867 | 0,822218 | 1,120081 |
| ***Slc10a2*** | 0,620419 | 0,93608 | 0,591403 | 0,981331 | 0,854708 |
| ***Fabp4*** | -0,12234 | 1,045039 | 1,446707 | 0,929734 | 0,149975 |
| ***Apobec1*** | 1,068508 | 0,595942 | 0,522453 | 0,411619 | 0,364742 |
| ***Fabp1*** | 0,377904 | 2,480121 | 1,289524 | -0,39195 | -0,87061 |
| ***Slco3a1*** | 0,218247 | 0,035296 | 0,407058 | 0,749166 | 1,112706 |
| ***Cav1*** | 0,773773 | 0,658437 | 0,150822 | 0,097915 | 0,546004 |
| ***Msn*** | -0,30299 | -0,12472 | 0,895414 | 0,692852 | 0,991822 |
| ***Hcar2*** | 0,07692 | 1,692572 | 0,145806 | -0,05036 | -0,05117 |
| ***Atp11a*** | 0,228036 | 0,308245 | 0,417813 | 0,410396 | 0,403075 |
| ***Abcc1*** | -0,32994 | 0,570196 | 0,756808 | 0,448079 | 0,320496 |
| ***Igf1*** | -0,0711 | 0,534636 | 0,478904 | 0,342446 | 0,463394 |
| ***Abcg2*** | 1,022892 | -0,17177 | -0,15845 | 0,248904 | 0,622251 |
| ***Il22*** | 0,257143 | 0,221181 | 0,380083 | 0,32858 | 0,361546 |
| ***Fabp2*** | 0,430475 | 1,37847 | -0,22077 | -0,13115 | 0,084636 |
| ***Bdnf*** | 0,244529 | 0,182207 | 0,014395 | 0,402227 | 0,692226 |
| ***Apoa5*** | -0,00171 | 1,200094 | 0,038027 | 0,117437 | 0,054655 |
| ***S100a8*** | 0,316813 | 0,389164 | 0,164116 | 0,207476 | 0,302258 |
| ***Ppard*** | 0,035931 | 0,504734 | 0,246923 | 0,193196 | 0,287906 |
| ***Sirt1*** | -0,0669 | 0,763695 | 0,318872 | 0,227024 | -0,0192 |
| ***Acsl1*** | 0,275928 | 1,416515 | 0,136382 | -0,25224 | -0,37723 |
| ***Abcg8*** | -0,39334 | 0,727529 | 0,795213 | 0,269695 | -0,23699 |
| ***Lpl*** | 0,511842 | 0,147046 | 0,090463 | 0,161693 | 0,189924 |
| ***Gpihbp1*** | 0,125463 | 0,410104 | 0,52057 | -0,14343 | 0,181107 |
| ***Lcat*** | -0,17959 | 0,579446 | 0,584129 | 0,271463 | -0,21531 |
| ***Ano6*** | 0,182379 | -0,01837 | 0,248597 | 0,39548 | 0,231441 |
| ***Star*** | -0,06788 | 0,067333 | 0,275318 | 0,342523 | 0,407752 |
| ***Chka*** | 0,426351 | 0,16697 | -0,22775 | 0,077874 | 0,541396 |
| ***Reln*** | -0,11612 | 0,046375 | 0,407064 | 0,275971 | 0,323788 |
| ***Lrat*** | -0,06049 | 0,208794 | 0,084343 | 0,359785 | 0,303068 |
| ***Slc13a5*** | -0,21528 | 0,850017 | 0,396288 | -0,07046 | -0,09864 |
| ***Mfsd2b*** | 0,00675 | 0,169819 | 0,069104 | 0,278894 | 0,328563 |
| ***Npc1l1*** | 0,22953 | 0,339241 | 0,066838 | 0,126331 | 0,037662 |
| ***Spns2*** | -0,53938 | -0,1185 | 0,477213 | 0,765442 | 0,198112 |
| ***F2*** | 0,060368 | 0,238874 | 0,224434 | 0,151489 | 0,097842 |
| ***Abca4*** | -0,22516 | 0,274006 | 0,336817 | 0,065528 | 0,289708 |
| ***Il1b*** | 0,263769 | 0,310882 | -0,03707 | 0,045763 | 0,139904 |
| ***Kcnn4*** | 0,259664 | -0,08772 | 0,175254 | 0,194479 | 0,178763 |
| ***Prkcd*** | 0,074277 | 0,348892 | 0,246503 | 0,028518 | 0,008762 |
| ***Stx1a*** | 0,114638 | 0,434371 | 0,114831 | 0,064172 | -0,0609 |
| ***Sp1*** | 0,094922 | 0,310516 | 0,068808 | 0,047906 | 0,139302 |
| ***Lrp10*** | 0,218111 | -0,32731 | 0,238422 | 0,311185 | 0,212797 |
| ***Lipg*** | -0,59591 | 0,579194 | 0,290071 | 0,226069 | 0,131944 |
| ***Map2k3*** | 0,106438 | 0,031734 | 0,048844 | 0,2398 | 0,194726 |
| ***Abca1*** | 0,121641 | 0,292213 | 0,159397 | 0,278491 | -0,26018 |
| ***Lamtor1*** | 0,079259 | -0,06169 | 0,228716 | 0,204765 | 0,136131 |
| ***Akr1c4*** | 0,140055 | 2,312705 | 0,392458 | -1,04147 | -1,21843 |
| ***Atp11b*** | 0,200468 | 0,064039 | 0,30509 | 0,085004 | -0,08501 |
| ***Pla2g10*** | -0,24089 | 0,31235 | 0,127281 | 0,119424 | 0,250417 |
| ***Lipc*** | -0,33561 | 0,056819 | 0,199002 | 0,359127 | 0,275048 |
| ***Itgav*** | 0,136453 | -0,41853 | 0,320676 | 0,1807 | 0,281092 |
| ***Irak1*** | 0,213248 | -0,22601 | 0,158575 | 0,292531 | 0,061371 |
| ***Stard5*** | 0,083151 | 0,345222 | 0,468553 | -0,09486 | -0,31932 |
| ***Rxrb*** | 0,457257 | 0,219044 | 0,120287 | -0,14449 | -0,17246 |
| ***Slc51a*** | -0,06567 | 0,345798 | 0,206038 | -0,09768 | 0,074967 |
| ***Slc27a4*** | 0,152291 | -0,23916 | 0,166582 | 0,259891 | 0,071442 |
| ***Triap1*** | 0,156075 | 0,396196 | 0,141518 | -0,03061 | -0,32942 |
| ***Clu*** | 0,169921 | 0,158111 | -0,1104 | -0,01523 | 0,119884 |
| ***Hras*** | 0,40146 | -0,23578 | -0,11532 | 0,234695 | 0,023973 |
| ***Cul3*** | 0,020882 | 0,207118 | 0,034457 | 0,08637 | -0,07733 |
| ***Slc27a2*** | 0,179649 | 1,385061 | -0,21278 | -0,39159 | -0,7137 |
| ***Abca12*** | -0,1599 | 0,141797 | 0,32541 | -0,11128 | 0,029805 |
| ***Slc9a3r2*** | 0,275201 | 0,14752 | -0,19208 | -0,07088 | 0,061981 |
| ***Ghr*** | 0,020345 | 0,862462 | -0,07016 | -0,35706 | -0,26179 |
| ***Ldlr*** | -0,19533 | -0,23531 | 0,05352 | 0,181182 | 0,380566 |
| ***Slc27a5*** | -0,19069 | 0,503285 | 0,492936 | -0,04094 | -0,59322 |
| ***Nfkbia*** | 0,010711 | 0,285474 | -0,04494 | -0,06765 | -0,0162 |
| ***Tnf*** | -0,18177 | -0,4173 | 0,296697 | 0,27893 | 0,183334 |
| ***Apoa2*** | -0,17361 | 0,299471 | 0,622124 | -0,05897 | -0,53274 |
| ***Scarb1*** | -0,17737 | 0,467088 | 0,007424 | 0,012039 | -0,19073 |
| ***Tmem30a*** | -0,04702 | 0,283345 | -0,00767 | -0,05335 | -0,06974 |
| ***Abcc6*** | -0,08474 | 0,294524 | 0,027542 | -0,08805 | -0,04459 |
| ***Slc25a13*** | -0,13467 | -0,22342 | 0,44586 | 0,19267 | -0,18293 |
| ***Scarb2*** | 0,203062 | -0,10702 | -0,06929 | 0,062819 | -0,00247 |
| ***Tspo*** | 0,199444 | -0,10564 | -0,08693 | -0,0227 | 0,093259 |
| ***Smpd1*** | 0,298504 | -0,25833 | -0,03771 | 0,003571 | 0,063222 |
| ***Akap13*** | 0,371115 | -0,58444 | 0,023724 | 0,158101 | 0,00517 |
| ***Slc25a10*** | 0,11865 | 0,696297 | -0,35526 | -0,25848 | -0,2367 |
| ***Slc6a11*** | -0,24472 | -0,0248 | -0,00414 | -0,11504 | 0,282429 |
| ***Arhgef11*** | 0,119541 | -0,26957 | -0,01881 | 0,049776 | 0,002864 |
| ***Srebf1*** | -0,75619 | 0,685746 | 0,064183 | -0,03825 | -0,08533 |
| ***Vps4b*** | -0,15861 | 0,143235 | -0,01297 | -0,07382 | -0,02915 |
| ***Cpt1b*** | 0,439677 | -0,44974 | 0,025189 | -0,08737 | -0,06385 |
| ***Apof*** | -0,10418 | 0,377569 | -0,25427 | -0,25009 | 0,07323 |
| ***Ces1*** | -0,12385 | 0,340526 | 0,219336 | 0,011072 | -0,6343 |
| ***Ttc39b*** | -0,21905 | 0,019599 | -0,13306 | 0,025097 | 0,10313 |
| ***Apoa1*** | 0,200219 | -0,07687 | -0,25979 | -0,08649 | 0,018263 |
| ***Pla2g4f*** | -0,328 | -0,04694 | 0,123387 | -0,09503 | 0,132622 |
| ***Hbp1*** | 0,182819 | 0,246372 | -0,08863 | -0,37777 | -0,18233 |
| ***Hnf1a*** | 0,141772 | -0,46934 | 0,133149 | 0,073791 | -0,10086 |
| ***Atp8b1*** | -0,01227 | -0,40007 | -0,09746 | 0,211675 | 0,070155 |
| ***Rac1*** | 0,004156 | -0,36322 | 0,012891 | 0,016666 | 0,099646 |
| ***Hspa8*** | -0,01242 | -0,20995 | -0,02698 | -0,01897 | 0,028543 |
| ***Aqp8*** | -0,71873 | 0,170539 | 0,222727 | 0,155749 | -0,08264 |
| ***Plscr1*** | 0,120276 | -0,77387 | -0,3144 | 0,258643 | 0,453488 |
| ***Ager*** | -0,37751 | 0,010039 | -0,07772 | -0,07408 | 0,26053 |
| ***Rhoa*** | -0,13676 | -0,1495 | 0,013621 | -0,03237 | 0,023586 |
| ***Slc25a17*** | 0,163416 | -0,30211 | -0,15694 | -0,05449 | 0,064203 |
| ***Scd2*** | -0,43642 | -0,0554 | -0,01182 | -0,0192 | 0,20793 |
| ***Ppara*** | 0,284157 | 0,016818 | -0,01524 | -0,10652 | -0,51136 |
| ***Cpt2*** | 0,166767 | -0,26646 | 0,024149 | -0,12539 | -0,15092 |
| ***P2rx7*** | -0,39759 | -0,02834 | -0,0222 | 0,050312 | 0,038123 |
| ***Abca5*** | 0,090601 | -0,44144 | -0,15628 | -0,00613 | 0,151655 |
| ***Apoe*** | 0,00429 | 0,122289 | -0,03966 | -0,30261 | -0,15475 |
| ***Got2*** | -0,08117 | -0,21755 | -0,02099 | 0,011125 | -0,07308 |
| ***Lss*** | -0,37367 | -0,13491 | 0,009673 | -0,05388 | 0,161206 |
| ***Canx*** | 0,013316 | -0,27514 | -0,10962 | 0,000647 | -0,02993 |
| ***Serpina6*** | -0,73851 | 0,531935 | 0,070508 | -0,01966 | -0,2586 |
| ***Slc27a1*** | -0,41268 | 0,148235 | 0,088034 | -0,06771 | -0,20017 |
| ***Slc6a13*** | -0,13277 | 0,569141 | -0,40062 | -0,28111 | -0,20817 |
| ***Psap*** | -0,19939 | 0,011129 | -0,06714 | -0,18966 | -0,03282 |
| ***Pitpna*** | -0,04177 | -0,55117 | 0,005303 | 0,079987 | 0,023119 |
| ***Ptch1*** | 0,262288 | -0,12041 | 0,031938 | -0,26919 | -0,39448 |
| ***Ephx1*** | 0,253109 | -0,37561 | -0,06008 | -0,12743 | -0,2109 |
| ***Apoh*** | -0,13351 | 0,128208 | -0,03296 | -0,26651 | -0,22174 |
| ***Slco2a1*** | 0,046009 | -1,01397 | -0,20624 | 0,30514 | 0,313243 |
| ***Pitpnb*** | -0,08261 | -0,15436 | -0,09378 | -0,15169 | -0,0915 |
| ***Slc36a1*** | -0,05822 | -0,34103 | -0,16696 | 0,073714 | -0,10515 |
| ***Osbp*** | 0,216893 | -0,62377 | -0,11025 | -0,01803 | -0,07237 |
| ***Fadd*** | 0,106753 | -0,48414 | -0,24516 | -0,03754 | 0,013868 |
| ***Acsl4*** | 0,022254 | -0,66087 | -0,26081 | -0,00454 | 0,213346 |
| ***Lbp*** | -0,33897 | -0,19207 | -0,26068 | -0,01096 | 0,109726 |
| ***Aqp9*** | -0,00116 | 0,275407 | -0,33264 | -0,34037 | -0,29478 |
| ***Stard3*** | -0,19645 | -0,28329 | -0,0961 | -0,05702 | -0,06698 |
| ***Plekha8*** | -0,18055 | -0,1673 | -0,20704 | 0,063617 | -0,20944 |
| ***Abcc10*** | 0,146101 | -0,29311 | -0,29604 | -0,09186 | -0,17382 |
| ***Tjp2*** | -0,26705 | -0,39514 | -0,14166 | -0,07741 | 0,158415 |
| ***Jak2*** | -0,02781 | -0,30249 | -0,25278 | 0,041496 | -0,19887 |
| ***Atp11c*** | 0,121907 | -0,51146 | -0,07754 | 0,001083 | -0,28015 |
| ***Slc16a1*** | -0,4491 | 0,02104 | -0,20466 | -0,24386 | 0,115867 |
| ***Scd*** | -0,13432 | 0,289914 | 0,097704 | -0,38032 | -0,63388 |
| ***Gsn*** | -0,10068 | -0,48996 | -0,15483 | -0,00717 | -0,02146 |
| ***Crat*** | -0,13528 | -0,3682 | -0,04971 | -0,08027 | -0,14379 |
| ***Arv1*** | -0,01046 | -0,2308 | -0,34189 | -0,03724 | -0,161 |
| ***Npc2*** | -0,37777 | -0,36177 | -0,13016 | -0,07041 | 0,090643 |
| ***Tnfaip8l3*** | -0,36713 | -0,02865 | -0,13969 | -0,19453 | -0,13112 |
| ***Gm2a*** | -0,19122 | -0,08217 | -0,38998 | -0,13446 | -0,06716 |
| ***Pctp*** | -0,4482 | -0,24054 | 0,059516 | -0,06193 | -0,19305 |
| ***Mapk9*** | -0,00623 | -0,34585 | -0,12821 | -0,25198 | -0,15795 |
| ***Nr1h3*** | 0,045567 | -0,18155 | -0,28072 | -0,24717 | -0,25165 |
| ***Plin2*** | 0,099249 | -0,88332 | -0,13492 | -0,13418 | 0,130005 |
| ***Crot*** | 0,047541 | 0,472301 | -0,58118 | -0,50706 | -0,38848 |
| ***Mttp*** | -0,05097 | -0,37223 | -0,1664 | -0,00399 | -0,37249 |
| ***Tert*** | -0,56719 | 0,007105 | -0,06652 | -0,35385 | -0,03381 |
| ***Lrp1*** | 0,060206 | -0,55842 | -0,2624 | -0,11997 | -0,17952 |
| ***Abcg5*** | -0,60954 | 0,480023 | 0,03509 | -0,40794 | -0,56029 |
| ***Il33*** | -0,29263 | 0,166507 | -0,72088 | -0,42045 | 0,177044 |
| ***Abcb11*** | -0,28694 | 0,895383 | -0,1812 | -0,56721 | -0,9809 |
| ***Fabp5*** | -0,71247 | 0,616043 | -0,73515 | -0,22654 | -0,06958 |
| ***Abcc3*** | -0,1613 | -0,49806 | -0,1044 | -0,23767 | -0,1381 |
| ***Slco1b3*** | 0,060062 | 1,853445 | -0,18141 | -1,3031 | -1,57247 |
| ***Abcc2*** | 0,030509 | -0,50224 | -0,16845 | -0,25012 | -0,25719 |
| ***Anxa6*** | -0,23768 | -0,58634 | -0,27128 | -0,15766 | 0,020174 |
| ***Abcd1*** | -0,49221 | 0,088577 | -0,0985 | -0,43144 | -0,32268 |
| ***Tmem30b*** | -0,13327 | 0,09786 | -0,12863 | -0,32433 | -0,77471 |
| ***Pcolce2*** | -0,01071 | 0,222022 | -0,40884 | -0,61582 | -0,51861 |
| ***Cert1*** | -0,42481 | -0,43365 | -0,14275 | -0,21424 | -0,1175 |
| ***Pld2*** | -0,14237 | -0,53318 | -0,49428 | -0,16364 | -0,00071 |
| ***Npc1*** | -0,24579 | 0,014381 | -0,29323 | -0,412 | -0,40126 |
| ***Cpt1a*** | 0,141407 | -0,3976 | -0,44114 | -0,41383 | -0,24206 |
| ***Acsl5*** | -0,30028 | -0,43237 | -0,34635 | -0,18066 | -0,10034 |
| ***Slc10a1*** | -0,28589 | 0,904126 | 0,417748 | -0,66668 | -1,77778 |
| ***Acat1*** | 0,022855 | 0,265225 | -0,44318 | -0,69614 | -0,61754 |
| ***Soat2*** | -0,2558 | -0,20424 | -0,22728 | -0,25872 | -0,55695 |
| ***Apoa4*** | -0,46224 | -0,92768 | -0,32289 | -0,02632 | 0,2323 |
| ***Tp53*** | -0,03313 | -0,694 | -0,51593 | -0,13647 | -0,15904 |
| ***Pcyt1a*** | -0,15554 | -0,76428 | -0,24717 | -0,25354 | -0,17347 |
| ***Vamp7*** | -0,15539 | -0,49023 | -0,31997 | -0,30985 | -0,33078 |
| ***Abcb4*** | 5,65E-05 | -0,36295 | -0,32981 | -0,44952 | -0,4935 |
| ***Sntb1*** | -0,02769 | -0,71682 | -0,66776 | -0,24059 | -0,082 |
| ***Nceh1*** | 0,132946 | -1,31178 | -0,44293 | -0,22489 | 0,021838 |
| ***Nr1h4*** | 0,255714 | -0,60739 | -0,50502 | -0,55488 | -0,43199 |
| ***Slc51b*** | -0,94048 | -0,25946 | -0,43251 | -0,19989 | -0,11032 |
| ***Cdcp1*** | -0,67129 | -0,45136 | -0,23891 | -0,21123 | -0,40572 |
| ***Apom*** | -0,33283 | -0,1507 | -0,93804 | -0,45161 | -0,17364 |
| ***Pon1*** | -0,20672 | 0,160498 | 0,102142 | -0,76871 | -1,36881 |
| ***App*** | -0,64282 | -0,94085 | -0,48604 | -0,276 | -0,02858 |
| ***Pltp*** | -0,54254 | -0,02021 | -0,53789 | -0,73388 | -0,55614 |
| ***Vldlr*** | -1,65317 | -0,33405 | 0,599907 | -0,31061 | -0,84224 |
| ***Lipa*** | -0,39884 | -1,07622 | -0,60296 | -0,43253 | -0,17624 |
| ***Rtn4*** | -1,37918 | -0,57005 | -0,21648 | -0,31862 | -0,21186 |
| ***Atp8a1*** | -0,39573 | -0,88063 | -0,56524 | -0,35161 | -0,55696 |
| ***Mfsd2a*** | -0,98722 | -0,83224 | -0,51057 | -0,13327 | -0,3093 |
| ***Pparg*** | 0,308282 | -1,26225 | -0,77772 | -0,62831 | -0,46886 |
| ***Pla1a*** | -0,50878 | -0,65272 | -0,89701 | -0,51342 | -0,26201 |
| ***Apcs*** | -0,71083 | -0,82579 | -1,01747 | -0,38636 | 0,102086 |
| ***Ntrk2*** | -2,47005 | -0,14335 | -0,30147 | -0,01801 | -0,01306 |
| ***Cyp7a1*** | -0,10229 | 1,351961 | -0,28199 | -1,67746 | -2,37028 |
| ***Slco1a2*** | -1,25718 | -0,22591 | -0,69486 | -0,50164 | -1,06208 |
| ***Abcb1b*** | -2,13704 | -1,12408 | -0,84069 | -0,67715 | -0,42022 |
| ***Abcc4*** | -0,77985 | -1,81574 | -1,03435 | -0,8991 | -1,03666 |
| ***Vnn1*** | -0,56203 | -1,95141 | -2,38867 | -1,60905 | -0,88811 |
| ***Abcb1*** | -3,15988 | -0,99045 | -1,36827 | -1,08103 | -0,80522 |
| ***Cd36*** | -1,76494 | -1,54792 | -2,50848 | -1,88358 | -1,04423 |
| ***Slco1a4*** | -1,59891 | -1,37986 | -2,1885 | -2,29899 | -2,58894 |

**Table S17:** Activation Z-score of genes involved in the oxidation of lipids male vs. female. Activation Z-score was calculated with IPA software from Qiagen.

| **Pathway Summary** | -1,759 | 0,898 | -1,597 | -2,287 | -2,664 |
| --- | --- | --- | --- | --- | --- |
| **genes in the oxidation of lipid network** | **0 h** | **24 h** | **48 h** | **72 h** | **96 h** |
| ***Obp2b*** | 2,916129 | 1,226669 | 0,565316 | 0,405337 | 0,308385 |
| ***Cyp2e1*** | 0,155034 | 0,940182 | 2,273941 | 0,984777 | -0,50221 |
| ***Hao1*** | 1,062404 | 2,229046 | 0,036742 | 0,065764 | 0,092746 |
| ***Fabp4*** | -0,12234 | 1,045039 | 1,446707 | 0,929734 | 0,149975 |
| ***Slc2a1*** | 0,247876 | 0,605368 | 0,992706 | 0,942282 | 0,601681 |
| ***Fabp1*** | 0,377904 | 2,480121 | 1,289524 | -0,39195 | -0,87061 |
| ***Aox3*** | 1,014655 | 1,687725 | 0,032444 | -0,4236 | -0,3305 |
| ***Adh4*** | 0,843657 | 1,099951 | -0,02072 | -0,12201 | -0,06158 |
| ***Cyp1a2*** | 0,066898 | 1,509168 | 1,215758 | 0,619908 | -1,80321 |
| ***Fabp2*** | 0,430475 | 1,37847 | -0,22077 | -0,13115 | 0,084636 |
| ***Phyh*** | 0,283241 | 1,296553 | 0,151206 | -0,14818 | -0,18579 |
| ***Nucb2*** | 0,75137 | -0,21708 | 0,317646 | 0,22747 | 0,259298 |
| ***Cyp27a1*** | 0,099837 | 0,68324 | 0,612828 | 0,138729 | -0,20747 |
| ***Sesn2*** | 0,114528 | 0,629052 | 0,199023 | 0,091054 | 0,267788 |
| ***Ppard*** | 0,035931 | 0,504734 | 0,246923 | 0,193196 | 0,287906 |
| ***Sirt1*** | -0,0669 | 0,763695 | 0,318872 | 0,227024 | -0,0192 |
| ***Mif*** | 0,042114 | 0,031338 | 0,387972 | 0,552037 | 0,186744 |
| ***Acsl1*** | 0,275928 | 1,416515 | 0,136382 | -0,25224 | -0,37723 |
| ***Acaa1*** | 0,400371 | 0,929744 | 0,067515 | -0,18491 | -0,03445 |
| ***Lpl*** | 0,511842 | 0,147046 | 0,090463 | 0,161693 | 0,189924 |
| ***Lcat*** | -0,17959 | 0,579446 | 0,584129 | 0,271463 | -0,21531 |
| ***Epo*** | 0,210116 | 0,2431 | 0,394999 | 0,258655 | -0,07248 |
| ***Abcc9*** | 0,004074 | 0,205181 | 0,433963 | 0,296835 | 0,078834 |
| ***Ins1*** | -0,15546 | 0,311846 | 0,152701 | 0,398085 | 0,245218 |
| ***Alox5*** | 0,097119 | 0,514201 | -0,04708 | 0,266093 | 0,107334 |
| ***Hsd17b10*** | 0,401057 | 0,621969 | 0,075927 | -0,05791 | -0,11075 |
| ***Acads*** | 0,342201 | 0,496937 | -0,02047 | -0,052 | 0,157163 |
| ***Wnt16*** | -0,05705 | 0,421468 | 0,20081 | 0,124623 | 0,226214 |
| ***Plin1*** | 0,023749 | 0,189395 | 0,063837 | 0,152108 | 0,479431 |
| ***Pdk2*** | 0,128752 | 0,363429 | 0,460486 | 0,134277 | -0,20922 |
| ***Sat1*** | -0,33138 | 0,393837 | 0,114153 | 0,278872 | 0,398208 |
| ***Crtc2*** | 0,079784 | 0,391697 | 0,155761 | 0,174674 | 0,040834 |
| ***Adh5*** | 0,051015 | 0,302692 | 0,253513 | 0,082737 | 0,112555 |
| ***Cyp1a1*** | -0,0377 | 0,221035 | 0,38464 | 0,078008 | 0,130361 |
| ***Nr4a3*** | -0,16451 | 0,339718 | 0,166224 | 0,009761 | 0,423004 |
| ***Acad10*** | 0,041873 | 0,58672 | 0,019932 | 0,265903 | -0,14637 |
| ***C1qtnf5*** | -0,08421 | 0,317356 | 0,173376 | 0,113237 | 0,227445 |
| ***Nrf1*** | 0,26316 | 0,739826 | -0,055 | -0,01539 | -0,19097 |
| ***Fitm2*** | 0,328933 | 0,147821 | 0,42145 | 0,054906 | -0,21785 |
| ***Il1b*** | 0,263769 | 0,310882 | -0,03707 | 0,045763 | 0,139904 |
| ***Cisd1*** | 0,247091 | 0,45393 | 0,076713 | 0,054741 | -0,11991 |
| ***Pex5*** | 0,358129 | 0,184889 | 0,389115 | -0,02045 | -0,23866 |
| ***Lpin1*** | 0,304783 | 0,830156 | 0,490199 | -0,31185 | -0,64224 |
| ***Cntf*** | 0,152345 | 0,005822 | 0,321655 | 0,103383 | 0,070471 |
| ***Vasp*** | -0,15743 | -0,0705 | 0,186711 | 0,314704 | 0,379149 |
| ***Aox1*** | 0,802634 | -0,23928 | 0,070857 | -0,07202 | 0,055333 |
| ***Por*** | -0,22385 | 0,116112 | 0,419562 | 0,346398 | -0,05581 |
| ***Abca1*** | 0,121641 | 0,292213 | 0,159397 | 0,278491 | -0,26018 |
| ***Akr1c4*** | 0,140055 | 2,312705 | 0,392458 | -1,04147 | -1,21843 |
| ***Mcat*** | 0,160198 | 0,294337 | -0,05166 | 0,122705 | 0,014659 |
| ***Abhd5*** | -0,06357 | 0,116803 | 0,159989 | 0,048191 | 0,257255 |
| ***Acadsb*** | 0,162206 | 0,336091 | -0,06282 | -0,0483 | 0,12505 |
| ***Tysnd1*** | 0,319976 | 0,170525 | 0,024379 | 0,023928 | -0,02769 |
| ***Snca*** | -0,42627 | 0,151173 | 0,30492 | 0,166235 | 0,313848 |
| ***Ppargc1b*** | 0,222911 | 0,302329 | 0,065047 | -0,02786 | -0,06079 |
| ***Cyp11b2*** | 0,076864 | 0,233931 | 0,212893 | -0,2635 | 0,227501 |
| ***Traf6*** | -0,0804 | 0,086174 | 0,300036 | 0,090793 | 0,066224 |
| ***Pla2g1b*** | 0,262145 | -0,0239 | -0,01338 | 0,010848 | 0,225401 |
| ***Pex13*** | 0,113692 | 0,118337 | 0,446837 | -0,1045 | -0,11591 |
| ***Csnk2b*** | 0,07672 | 0,050343 | 0,09571 | 0,184862 | 0,044891 |
| ***Igf1r*** | -0,0981 | -0,0826 | 0,279362 | 0,110573 | 0,242523 |
| ***Angptl4*** | 0,581837 | 0,508041 | -0,05698 | -0,22451 | -0,35668 |
| ***Acox1*** | 0,201106 | 0,527651 | 0,197574 | -0,20825 | -0,29447 |
| ***Slc27a4*** | 0,152291 | -0,23916 | 0,166582 | 0,259891 | 0,071442 |
| ***Adipor1*** | 0,056755 | -0,02311 | 0,123791 | 0,204018 | 0,041937 |
| ***Acaa2*** | 0,088348 | 1,205352 | -0,2018 | -0,36845 | -0,3216 |
| ***Foxo3*** | 0,150165 | 0,280371 | 0,170242 | -0,06662 | -0,14944 |
| ***Pebp1*** | 0,255059 | 0,090682 | 0,01275 | 0,07844 | -0,05793 |
| ***Stk25*** | 0,190238 | -0,10705 | 0,088566 | 0,103183 | 0,059687 |
| ***Gnas*** | 0,157345 | -0,00359 | 0,066071 | 0,077306 | -0,00319 |
| ***Bdh2*** | 0,055083 | 0,476438 | -0,1999 | -0,11685 | 0,045387 |
| ***Crtc3*** | -0,01524 | 0,346289 | -0,02403 | 0,105226 | -0,1638 |
| ***Slc27a2*** | 0,179649 | 1,385061 | -0,21278 | -0,39159 | -0,7137 |
| ***Pecr*** | -0,07769 | 0,757183 | -0,12921 | -0,12135 | -0,19212 |
| ***Ghrl*** | -0,25736 | 0,238318 | 0,189848 | 0,054328 | -0,0071 |
| ***Eci1*** | 0,191785 | 0,457583 | -0,13468 | -0,0355 | -0,27868 |
| ***Lonp2*** | 0,175537 | 0,365258 | -0,0901 | -0,15976 | -0,12067 |
| ***Tnf*** | -0,18177 | -0,4173 | 0,296697 | 0,27893 | 0,183334 |
| ***Apoa2*** | -0,17361 | 0,299471 | 0,622124 | -0,05897 | -0,53274 |
| ***Mc5r*** | -0,47844 | 0,146795 | 0,229753 | -0,15003 | 0,405924 |
| ***Ksr2*** | -0,25932 | -0,00745 | 0,111444 | -0,00557 | 0,314614 |
| ***Acadvl*** | 0,04424 | 0,257297 | 0,054568 | -0,1052 | -0,12314 |
| ***Scarb1*** | -0,17737 | 0,467088 | 0,007424 | 0,012039 | -0,19073 |
| ***Pnpla2*** | 0,247679 | -0,29319 | 0,098474 | 0,152412 | -0,12929 |
| ***Cat*** | 0,079423 | 0,133675 | -0,05435 | -0,08199 | -0,03185 |
| ***Hsd17b6*** | -0,85447 | 0,627825 | 0,266065 | 0,140589 | -0,14635 |
| ***Lrpprc*** | 0,157178 | -0,46375 | 0,21635 | 0,196295 | -0,0811 |
| ***Cartpt*** | -0,24809 | 0,105997 | 0,057639 | -0,16222 | 0,236838 |
| ***Rdh5*** | -0,31391 | 0,133303 | 0,132877 | 0,120966 | -0,1159 |
| ***Epas1*** | 0,107156 | 0,425131 | -0,27261 | -0,08373 | -0,22467 |
| ***Sirt4*** | -0,21548 | 0,198685 | 0,044321 | -0,10078 | 0,000804 |
| ***Gcdh*** | 0,101655 | 0,665216 | -0,28731 | -0,28521 | -0,27466 |
| ***Srebf1*** | -0,75619 | 0,685746 | 0,064183 | -0,03825 | -0,08533 |
| ***Wnt2b*** | -0,24021 | 0,381736 | -0,0254 | -0,29238 | 0,044515 |
| ***Cpt1b*** | 0,439677 | -0,44974 | 0,025189 | -0,08737 | -0,06385 |
| ***Mapk14*** | 0,220987 | -0,26318 | 0,014724 | -0,02297 | -0,09842 |
| ***C3*** | -0,17852 | 8,03E-05 | -0,12049 | 0,058399 | 0,081334 |
| ***Cycs*** | 0,046517 | -0,23245 | 0,016117 | 0,02394 | -0,02406 |
| ***Mecr*** | 0,338302 | -0,05856 | -0,26117 | -0,08225 | -0,11077 |
| ***Apoa1*** | 0,200219 | -0,07687 | -0,25979 | -0,08649 | 0,018263 |
| ***Pon2*** | 0,295087 | -0,47237 | -0,19 | 0,01826 | 0,132092 |
| ***Bloc1s1*** | 0,065027 | -0,22069 | -0,04954 | 0,005379 | -0,0268 |
| ***Acox3*** | 0,261327 | -0,09882 | -0,01784 | -0,26157 | -0,12673 |
| ***Echs1*** | -0,10056 | 0,263699 | -0,22657 | -0,14549 | -0,04605 |
| ***Pex2*** | 0,068347 | -0,15174 | 0,080045 | -0,24601 | -0,03156 |
| ***Slc25a17*** | 0,163416 | -0,30211 | -0,15694 | -0,05449 | 0,064203 |
| ***Serpinf1*** | -0,17477 | 0,42893 | -0,18892 | -0,31322 | -0,04496 |
| ***Atg7*** | 0,193859 | 0,230731 | -0,31903 | -0,38279 | -0,02735 |
| ***Ppara*** | 0,284157 | 0,016818 | -0,01524 | -0,10652 | -0,51136 |
| ***Immt*** | 0,068994 | -0,33123 | -0,12185 | 0,001712 | 0,03129 |
| ***Cpt2*** | 0,166767 | -0,26646 | 0,024149 | -0,12539 | -0,15092 |
| ***P2rx7*** | -0,39759 | -0,02834 | -0,0222 | 0,050312 | 0,038123 |
| ***Apoe*** | 0,00429 | 0,122289 | -0,03966 | -0,30261 | -0,15475 |
| ***Hadha*** | 0,048218 | -0,04795 | -0,04955 | -0,1482 | -0,18075 |
| ***Got2*** | -0,08117 | -0,21755 | -0,02099 | 0,011125 | -0,07308 |
| ***Pnpla8*** | -0,02232 | -0,0185 | 0,08681 | -0,16123 | -0,30148 |
| ***Hacl1*** | -0,4887 | 0,469336 | -0,09953 | -0,14284 | -0,16455 |
| ***Adh1c*** | -0,16409 | 0,881367 | -0,32695 | -0,40502 | -0,41997 |
| ***Uchl3*** | -0,09658 | -0,59858 | 0,214475 | 0,108079 | -0,06785 |
| ***Slc27a1*** | -0,41268 | 0,148235 | 0,088034 | -0,06771 | -0,20017 |
| ***Nr0b2*** | -0,35622 | 0,233567 | 0,047325 | -0,39205 | 0,021575 |
| ***Stat6*** | 0,273005 | -0,43594 | -0,2113 | -0,07114 | -0,00908 |
| ***Lclat1*** | -0,22461 | -0,0068 | -0,05603 | -0,08514 | -0,08289 |
| ***Acadm*** | -0,03751 | 0,003338 | 0,012985 | -0,22625 | -0,21789 |
| ***Insr*** | -0,04304 | -0,30847 | -0,01885 | -0,02239 | -0,12176 |
| ***Eci2*** | 0,191046 | 0,091902 | -0,17084 | -0,39478 | -0,26362 |
| ***Slco2a1*** | 0,046009 | -1,01397 | -0,20624 | 0,30514 | 0,313243 |
| ***Prkaa2*** | 0,026958 | -0,16209 | -0,16135 | -0,10442 | -0,16972 |
| ***Acox2*** | 0,207147 | -0,5143 | -0,00712 | -0,1187 | -0,13892 |
| ***Hif1a*** | -0,06375 | -0,27724 | -0,25817 | 0,067145 | -0,05719 |
| ***Akt1*** | -0,12742 | -0,47145 | -0,12531 | -0,0356 | 0,152021 |
| ***Acbd5*** | 0,036182 | 0,094417 | -0,17579 | -0,28295 | -0,30968 |
| ***Hsd11b1*** | -0,18894 | 0,270435 | -0,05613 | -0,22592 | -0,44326 |
| ***Dgat2*** | -0,08642 | 0,211404 | -0,25964 | -0,24013 | -0,31512 |
| ***Acsl4*** | 0,022254 | -0,66087 | -0,26081 | -0,00454 | 0,213346 |
| ***Ide*** | 0,140645 | -0,60517 | -0,08274 | -0,05303 | -0,09874 |
| ***Sirt5*** | 0,019741 | -0,08465 | -0,23853 | -0,18128 | -0,21806 |
| ***Cyp2j2*** | 0,072261 | 0,23827 | -0,40419 | -0,37816 | -0,2528 |
| ***Scd*** | -0,13432 | 0,289914 | 0,097704 | -0,38032 | -0,63388 |
| ***Hadhb*** | -0,00902 | -0,26719 | -0,00514 | -0,30467 | -0,20535 |
| ***Cyp2c9*** | -0,67462 | 0,257784 | 0,167694 | -0,2441 | -0,31643 |
| ***Decr1*** | 0,10697 | 0,084374 | -0,32973 | -0,45541 | -0,24776 |
| ***Slc22a5*** | 0,026135 | -0,21294 | 0,023082 | -0,34764 | -0,36809 |
| ***Esrrg*** | -0,54109 | 0,285128 | -0,14111 | -0,28518 | -0,20333 |
| ***Plin2*** | 0,099249 | -0,88332 | -0,13492 | -0,13418 | 0,130005 |
| ***Acadl*** | -0,20704 | -0,55124 | -0,02583 | -0,07497 | -0,06891 |
| ***Acad11*** | -0,08057 | -0,06572 | -0,00887 | -0,43239 | -0,34124 |
| ***Pdk4*** | -0,7756 | 0,003353 | 0,254408 | -0,1653 | -0,2616 |
| ***Crot*** | 0,047541 | 0,472301 | -0,58118 | -0,50706 | -0,38848 |
| ***Adipor2*** | -0,11821 | 0,070816 | -0,11016 | -0,35886 | -0,44666 |
| ***Ceacam1*** | -0,1119 | 0,280183 | -0,65997 | -0,21636 | -0,25564 |
| ***Mttp*** | -0,05097 | -0,37223 | -0,1664 | -0,00399 | -0,37249 |
| ***Hadh*** | -0,2833 | 0,412573 | -0,43077 | -0,41483 | -0,27304 |
| ***Mtor*** | 0,012855 | -0,15939 | -0,11537 | -0,44566 | -0,29787 |
| ***Pex7*** | -0,17079 | -0,01757 | -0,31339 | -0,32498 | -0,20551 |
| ***Cyb5r4*** | -0,23801 | -0,74587 | 0,016898 | 0,002044 | -0,07298 |
| ***Hsd17b4*** | 0,162203 | -0,53786 | -0,16633 | -0,25361 | -0,24445 |
| ***Adrb2*** | -0,01087 | -0,54006 | -0,22739 | -0,07428 | -0,2077 |
| ***Kras*** | -0,26815 | -0,15401 | -0,19245 | -0,26561 | -0,23842 |
| ***Hmgcs2*** | -0,00749 | 0,551565 | -0,54596 | -0,59436 | -0,53061 |
| ***Srebf2*** | -0,43648 | -0,59157 | -0,18447 | -0,00592 | 0,067099 |
| ***Thrb*** | 0,064914 | -0,58191 | -0,25835 | -0,15244 | -0,24924 |
| ***Aldh1a1*** | 0,149176 | -0,68979 | -0,46523 | -0,18232 | 0,010048 |
| ***Aldh1a7*** | 0,005718 | -0,78892 | -0,30654 | -0,03241 | -0,06392 |
| ***Mcu*** | -0,44396 | -0,53601 | -0,26586 | -0,09492 | 0,129141 |
| ***Nt5c2*** | -0,28444 | -0,53902 | -0,23814 | -0,08771 | -0,07632 |
| ***Abcd1*** | -0,49221 | 0,088577 | -0,0985 | -0,43144 | -0,32268 |
| ***Cpt1a*** | 0,141407 | -0,3976 | -0,44114 | -0,41383 | -0,24206 |
| ***Acsl5*** | -0,30028 | -0,43237 | -0,34635 | -0,18066 | -0,10034 |
| ***Ehhadh*** | -0,21017 | -0,18064 | -0,0393 | -0,47618 | -0,4945 |
| ***C2cd5*** | -0,17154 | -0,41825 | -0,36472 | -0,26164 | -0,23377 |
| ***Tp53*** | -0,03313 | -0,694 | -0,51593 | -0,13647 | -0,15904 |
| ***Aadac*** | 0,009432 | 0,910068 | -0,45339 | -1,1259 | -0,89432 |
| ***Il15*** | -0,59553 | -0,11774 | -0,45572 | -0,1838 | -0,26009 |
| ***Fasn*** | -0,78593 | -0,47232 | -0,08825 | 0,042331 | -0,31419 |
| ***Gpam*** | -0,22696 | -0,85832 | -0,13764 | -0,24223 | -0,15404 |
| ***Rdh16*** | -1,11795 | 0,483381 | -0,21248 | -0,30865 | -0,46387 |
| ***Esr1*** | -0,8671 | 0,042478 | -0,57455 | -0,17213 | -0,09752 |
| ***Camkk2*** | -0,20439 | -1,09832 | -0,43801 | 0,090404 | -0,07779 |
| ***Bcl2*** | -0,06053 | -0,19298 | -1,1254 | -0,45546 | 0,040528 |
| ***Irs1*** | -0,02384 | -0,72107 | -0,35943 | -0,17462 | -0,53636 |
| ***Aldh3a2*** | -0,07275 | -0,76701 | -0,39468 | -0,41599 | -0,25463 |
| ***Ucp2*** | 0,348379 | -0,17992 | -1,08821 | -0,76601 | -0,25163 |
| ***Hpgd*** | -0,56275 | -0,65989 | 0,03402 | -0,31819 | -0,44264 |
| ***Prkag2*** | -0,50905 | -0,84728 | -0,13855 | -0,19606 | -0,26978 |
| ***Cdcp1*** | -0,67129 | -0,45136 | -0,23891 | -0,21123 | -0,40572 |
| ***Pon1*** | -0,20672 | 0,160498 | 0,102142 | -0,76871 | -1,36881 |
| ***Nr1i3*** | -0,76069 | 0,127178 | -0,24139 | -0,64651 | -0,60801 |
| ***App*** | -0,64282 | -0,94085 | -0,48604 | -0,276 | -0,02858 |
| ***Ppargc1a*** | -0,79412 | -0,38501 | -0,49487 | -0,42794 | -0,66493 |
| ***Pparg*** | 0,308282 | -1,26225 | -0,77772 | -0,62831 | -0,46886 |
| ***Acaca*** | -0,81725 | -0,50812 | -0,39237 | -0,72828 | -0,71412 |
| ***Cyp4a11*** | -0,5732 | 0,642984 | -0,96937 | -1,57802 | -1,1825 |
| ***Rbp1*** | -0,73896 | -0,81934 | -1,09913 | -0,56668 | -0,49116 |
| ***Hao2*** | -3,73433 | -0,786 | 0,012724 | 0,079001 | -0,16565 |
| ***Rgs16*** | -1,2624 | 0,446081 | -0,93258 | -1,38671 | -1,85131 |
| ***Akr1c3*** | -0,27282 | -0,12655 | -2,54033 | -1,89342 | -0,62448 |
| ***Cyp4a14*** | -0,75272 | -0,24857 | -1,13345 | -2,35025 | -2,68532 |
| ***Abcd2*** | -3,21303 | -0,4612 | -1,06522 | -1,32308 | -1,44821 |
| ***Cd36*** | -1,76494 | -1,54792 | -2,50848 | -1,88358 | -1,04423 |
| ***Cyp2b6*** | -4,87545 | -1,58102 | -1,3552 | -1,03239 | -1,02875 |
| ***Cyp3a5*** | -4,0533 | -3,72033 | -2,12901 | 0,626905 | -0,99918 |
| ***Cyp2c8*** | -3,0275 | -2,40431 | -1,64767 | -1,56727 | -2,10398 |

**Table S18:** Activation Z-score of genes involved in cholesterol transport male vs. female. Activation Z-score was calculated with IPA software from Qiagen.

| **Pathway Summary** | -1,19 | -1,687 | no Z-score | -2,763 | -2,259 |
| --- | --- | --- | --- | --- | --- |
| **genes in the cholesterol transport network** | **0 h** | **24 h** | **48 h** | **72 h** | **96 h** |
| ***Saa1*** | -0,22883 | 0,807029 | 1,679516 | 1,794978 | 0,628602 |
| ***Scp2*** | 0,905224 | 2,031537 | 0,985545 | 0,350656 | 0,253287 |
| ***F2r*** | 1,338302 | 0,862247 | 0,770082 | 0,465858 | 0,599782 |
| ***Gdf15*** | 0,203624 | 1,295703 | 0,569867 | 0,822218 | 1,120081 |
| ***Fabp4*** | -0,12234 | 1,045039 | 1,446707 | 0,929734 | 0,149975 |
| ***Apobec1*** | 1,068508 | 0,595942 | 0,522453 | 0,411619 | 0,364742 |
| ***Fabp1*** | 0,377904 | 2,480121 | 1,289524 | -0,39195 | -0,87061 |
| ***Cav1*** | 0,773773 | 0,658437 | 0,150822 | 0,097915 | 0,546004 |
| ***Msn*** | -0,30299 | -0,12472 | 0,895414 | 0,692852 | 0,991822 |
| ***Hcar2*** | 0,07692 | 1,692572 | 0,145806 | -0,05036 | -0,05117 |
| ***Il22*** | 0,257143 | 0,221181 | 0,380083 | 0,32858 | 0,361546 |
| ***Apoa5*** | -0,00171 | 1,200094 | 0,038027 | 0,117437 | 0,054655 |
| ***S100a8*** | 0,316813 | 0,389164 | 0,164116 | 0,207476 | 0,302258 |
| ***Ppard*** | 0,035931 | 0,504734 | 0,246923 | 0,193196 | 0,287906 |
| ***Sirt1*** | -0,0669 | 0,763695 | 0,318872 | 0,227024 | -0,0192 |
| ***Acsl1*** | 0,275928 | 1,416515 | 0,136382 | -0,25224 | -0,37723 |
| ***Abcg8*** | -0,39334 | 0,727529 | 0,795213 | 0,269695 | -0,23699 |
| ***Lpl*** | 0,511842 | 0,147046 | 0,090463 | 0,161693 | 0,189924 |
| ***Lcat*** | -0,17959 | 0,579446 | 0,584129 | 0,271463 | -0,21531 |
| ***Star*** | -0,06788 | 0,067333 | 0,275318 | 0,342523 | 0,407752 |
| ***Reln*** | -0,11612 | 0,046375 | 0,407064 | 0,275971 | 0,323788 |
| ***Npc1l1*** | 0,22953 | 0,339241 | 0,066838 | 0,126331 | 0,037662 |
| ***F2*** | 0,060368 | 0,238874 | 0,224434 | 0,151489 | 0,097842 |
| ***Il1b*** | 0,263769 | 0,310882 | -0,03707 | 0,045763 | 0,139904 |
| ***Prkcd*** | 0,074277 | 0,348892 | 0,246503 | 0,028518 | 0,008762 |
| ***Sp1*** | 0,094922 | 0,310516 | 0,068808 | 0,047906 | 0,139302 |
| ***Lipg*** | -0,59591 | 0,579194 | 0,290071 | 0,226069 | 0,131944 |
| ***Map2k3*** | 0,106438 | 0,031734 | 0,048844 | 0,2398 | 0,194726 |
| ***Abca1*** | 0,121641 | 0,292213 | 0,159397 | 0,278491 | -0,26018 |
| ***Lipc*** | -0,33561 | 0,056819 | 0,199002 | 0,359127 | 0,275048 |
| ***Irak1*** | 0,213248 | -0,22601 | 0,158575 | 0,292531 | 0,061371 |
| ***Stard5*** | 0,083151 | 0,345222 | 0,468553 | -0,09486 | -0,31932 |
| ***Rxrb*** | 0,457257 | 0,219044 | 0,120287 | -0,14449 | -0,17246 |
| ***Slc51a*** | -0,06567 | 0,345798 | 0,206038 | -0,09768 | 0,074967 |
| ***Clu*** | 0,169921 | 0,158111 | -0,1104 | -0,01523 | 0,119884 |
| ***Hras*** | 0,40146 | -0,23578 | -0,11532 | 0,234695 | 0,023973 |
| ***Cul3*** | 0,020882 | 0,207118 | 0,034457 | 0,08637 | -0,07733 |
| ***Ldlr*** | -0,19533 | -0,23531 | 0,05352 | 0,181182 | 0,380566 |
| ***Nfkbia*** | 0,010711 | 0,285474 | -0,04494 | -0,06765 | -0,0162 |
| ***Tnf*** | -0,18177 | -0,4173 | 0,296697 | 0,27893 | 0,183334 |
| ***Apoa2*** | -0,17361 | 0,299471 | 0,622124 | -0,05897 | -0,53274 |
| ***Scarb1*** | -0,17737 | 0,467088 | 0,007424 | 0,012039 | -0,19073 |
| ***Tspo*** | 0,199444 | -0,10564 | -0,08693 | -0,0227 | 0,093259 |
| ***Smpd1*** | 0,298504 | -0,25833 | -0,03771 | 0,003571 | 0,063222 |
| ***Akap13*** | 0,371115 | -0,58444 | 0,023724 | 0,158101 | 0,00517 |
| ***Arhgef11*** | 0,119541 | -0,26957 | -0,01881 | 0,049776 | 0,002864 |
| ***Srebf1*** | -0,75619 | 0,685746 | 0,064183 | -0,03825 | -0,08533 |
| ***Vps4b*** | -0,15861 | 0,143235 | -0,01297 | -0,07382 | -0,02915 |
| ***Apof*** | -0,10418 | 0,377569 | -0,25427 | -0,25009 | 0,07323 |
| ***Ces1*** | -0,12385 | 0,340526 | 0,219336 | 0,011072 | -0,6343 |
| ***Ttc39b*** | -0,21905 | 0,019599 | -0,13306 | 0,025097 | 0,10313 |
| ***Apoa1*** | 0,200219 | -0,07687 | -0,25979 | -0,08649 | 0,018263 |
| ***Hnf1a*** | 0,141772 | -0,46934 | 0,133149 | 0,073791 | -0,10086 |
| ***Rac1*** | 0,004156 | -0,36322 | 0,012891 | 0,016666 | 0,099646 |
| ***Ager*** | -0,37751 | 0,010039 | -0,07772 | -0,07408 | 0,26053 |
| ***Rhoa*** | -0,13676 | -0,1495 | 0,013621 | -0,03237 | 0,023586 |
| ***Scd2*** | -0,43642 | -0,0554 | -0,01182 | -0,0192 | 0,20793 |
| ***Ppara*** | 0,284157 | 0,016818 | -0,01524 | -0,10652 | -0,51136 |
| ***Abca5*** | 0,090601 | -0,44144 | -0,15628 | -0,00613 | 0,151655 |
| ***Apoe*** | 0,00429 | 0,122289 | -0,03966 | -0,30261 | -0,15475 |
| ***Lss*** | -0,37367 | -0,13491 | 0,009673 | -0,05388 | 0,161206 |
| ***Canx*** | 0,013316 | -0,27514 | -0,10962 | 0,000647 | -0,02993 |
| ***Ptch1*** | 0,262288 | -0,12041 | 0,031938 | -0,26919 | -0,39448 |
| ***Stard3*** | -0,19645 | -0,28329 | -0,0961 | -0,05702 | -0,06698 |
| ***Jak2*** | -0,02781 | -0,30249 | -0,25278 | 0,041496 | -0,19887 |
| ***Scd*** | -0,13432 | 0,289914 | 0,097704 | -0,38032 | -0,63388 |
| ***Npc2*** | -0,37777 | -0,36177 | -0,13016 | -0,07041 | 0,090643 |
| ***Pctp*** | -0,4482 | -0,24054 | 0,059516 | -0,06193 | -0,19305 |
| ***Mapk9*** | -0,00623 | -0,34585 | -0,12821 | -0,25198 | -0,15795 |
| ***Nr1h3*** | 0,045567 | -0,18155 | -0,28072 | -0,24717 | -0,25165 |
| ***Mttp*** | -0,05097 | -0,37223 | -0,1664 | -0,00399 | -0,37249 |
| ***Tert*** | -0,56719 | 0,007105 | -0,06652 | -0,35385 | -0,03381 |
| ***Lrp1*** | 0,060206 | -0,55842 | -0,2624 | -0,11997 | -0,17952 |
| ***Abcg5*** | -0,60954 | 0,480023 | 0,03509 | -0,40794 | -0,56029 |
| ***Il33*** | -0,29263 | 0,166507 | -0,72088 | -0,42045 | 0,177044 |
| ***Abcb11*** | -0,28694 | 0,895383 | -0,1812 | -0,56721 | -0,9809 |
| ***Anxa6*** | -0,23768 | -0,58634 | -0,27128 | -0,15766 | 0,020174 |
| ***Pcolce2*** | -0,01071 | 0,222022 | -0,40884 | -0,61582 | -0,51861 |
| ***Pld2*** | -0,14237 | -0,53318 | -0,49428 | -0,16364 | -0,00071 |
| ***Npc1*** | -0,24579 | 0,014381 | -0,29323 | -0,412 | -0,40126 |
| ***Acat1*** | 0,022855 | 0,265225 | -0,44318 | -0,69614 | -0,61754 |
| ***Soat2*** | -0,2558 | -0,20424 | -0,22728 | -0,25872 | -0,55695 |
| ***Apoa4*** | -0,46224 | -0,92768 | -0,32289 | -0,02632 | 0,2323 |
| ***Tp53*** | -0,03313 | -0,694 | -0,51593 | -0,13647 | -0,15904 |
| ***Pcyt1a*** | -0,15554 | -0,76428 | -0,24717 | -0,25354 | -0,17347 |
| ***Abcb4*** | 5,65E-05 | -0,36295 | -0,32981 | -0,44952 | -0,4935 |
| ***Sntb1*** | -0,02769 | -0,71682 | -0,66776 | -0,24059 | -0,082 |
| ***Nceh1*** | 0,132946 | -1,31178 | -0,44293 | -0,22489 | 0,021838 |
| ***Nr1h4*** | 0,255714 | -0,60739 | -0,50502 | -0,55488 | -0,43199 |
| ***Slc51b*** | -0,94048 | -0,25946 | -0,43251 | -0,19989 | -0,11032 |
| ***Apom*** | -0,33283 | -0,1507 | -0,93804 | -0,45161 | -0,17364 |
| ***Pon1*** | -0,20672 | 0,160498 | 0,102142 | -0,76871 | -1,36881 |
| ***App*** | -0,64282 | -0,94085 | -0,48604 | -0,276 | -0,02858 |
| ***Pltp*** | -0,54254 | -0,02021 | -0,53789 | -0,73388 | -0,55614 |
| ***Vldlr*** | -1,65317 | -0,33405 | 0,599907 | -0,31061 | -0,84224 |
| ***Lipa*** | -0,39884 | -1,07622 | -0,60296 | -0,43253 | -0,17624 |
| ***Pparg*** | 0,308282 | -1,26225 | -0,77772 | -0,62831 | -0,46886 |
| ***Pla1a*** | -0,50878 | -0,65272 | -0,89701 | -0,51342 | -0,26201 |
| ***Apcs*** | -0,71083 | -0,82579 | -1,01747 | -0,38636 | 0,102086 |
| ***Cyp7a1*** | -0,10229 | 1,351961 | -0,28199 | -1,67746 | -2,37028 |
| ***Abcb1b*** | -2,13704 | -1,12408 | -0,84069 | -0,67715 | -0,42022 |
| ***Vnn1*** | -0,56203 | -1,95141 | -2,38867 | -1,60905 | -0,88811 |
| ***Abcb1*** | -3,15988 | -0,99045 | -1,36827 | -1,08103 | -0,80522 |
| ***Cd36*** | -1,76494 | -1,54792 | -2,50848 | -1,88358 | -1,04423 |

**Table S19:** Activation Z-score of genes involved in the transport of steroids male vs. female. Activation Z-score was calculated with IPA software from Qiagen.

| **Pathway Summary** | -1,438 | -1,277 | no Z-score | -3,243 | -2,777 |
| --- | --- | --- | --- | --- | --- |
| **genes in the transport of steroid network** | **0 h** | **24 h** | **48 h** | **72 h** | **96 h** |
| ***Slco1a1*** | 3,103735 | 4,931852 | 3,018541 | 2,176901 | 1,075615 |
| ***Saa1*** | -0,22883 | 0,807029 | 1,679516 | 1,794978 | 0,628602 |
| ***Scp2*** | 0,905224 | 2,031537 | 0,985545 | 0,350656 | 0,253287 |
| ***F2r*** | 1,338302 | 0,862247 | 0,770082 | 0,465858 | 0,599782 |
| ***Gdf15*** | 0,203624 | 1,295703 | 0,569867 | 0,822218 | 1,120081 |
| ***Slc10a2*** | 0,620419 | 0,93608 | 0,591403 | 0,981331 | 0,854708 |
| ***Fabp4*** | -0,12234 | 1,045039 | 1,446707 | 0,929734 | 0,149975 |
| ***Apobec1*** | 1,068508 | 0,595942 | 0,522453 | 0,411619 | 0,364742 |
| ***Fabp1*** | 0,377904 | 2,480121 | 1,289524 | -0,39195 | -0,87061 |
| ***Cav1*** | 0,773773 | 0,658437 | 0,150822 | 0,097915 | 0,546004 |
| ***Msn*** | -0,30299 | -0,12472 | 0,895414 | 0,692852 | 0,991822 |
| ***Hcar2*** | 0,07692 | 1,692572 | 0,145806 | -0,05036 | -0,05117 |
| ***Abcc1*** | -0,32994 | 0,570196 | 0,756808 | 0,448079 | 0,320496 |
| ***Il22*** | 0,257143 | 0,221181 | 0,380083 | 0,32858 | 0,361546 |
| ***Apoa5*** | -0,00171 | 1,200094 | 0,038027 | 0,117437 | 0,054655 |
| ***S100a8*** | 0,316813 | 0,389164 | 0,164116 | 0,207476 | 0,302258 |
| ***Ppard*** | 0,035931 | 0,504734 | 0,246923 | 0,193196 | 0,287906 |
| ***Sirt1*** | -0,0669 | 0,763695 | 0,318872 | 0,227024 | -0,0192 |
| ***Acsl1*** | 0,275928 | 1,416515 | 0,136382 | -0,25224 | -0,37723 |
| ***Abcg8*** | -0,39334 | 0,727529 | 0,795213 | 0,269695 | -0,23699 |
| ***Lpl*** | 0,511842 | 0,147046 | 0,090463 | 0,161693 | 0,189924 |
| ***Lcat*** | -0,17959 | 0,579446 | 0,584129 | 0,271463 | -0,21531 |
| ***Star*** | -0,06788 | 0,067333 | 0,275318 | 0,342523 | 0,407752 |
| ***Reln*** | -0,11612 | 0,046375 | 0,407064 | 0,275971 | 0,323788 |
| ***Npc1l1*** | 0,22953 | 0,339241 | 0,066838 | 0,126331 | 0,037662 |
| ***F2*** | 0,060368 | 0,238874 | 0,224434 | 0,151489 | 0,097842 |
| ***Il1b*** | 0,263769 | 0,310882 | -0,03707 | 0,045763 | 0,139904 |
| ***Prkcd*** | 0,074277 | 0,348892 | 0,246503 | 0,028518 | 0,008762 |
| ***Sp1*** | 0,094922 | 0,310516 | 0,068808 | 0,047906 | 0,139302 |
| ***Lipg*** | -0,59591 | 0,579194 | 0,290071 | 0,226069 | 0,131944 |
| ***Map2k3*** | 0,106438 | 0,031734 | 0,048844 | 0,2398 | 0,194726 |
| ***Abca1*** | 0,121641 | 0,292213 | 0,159397 | 0,278491 | -0,26018 |
| ***Akr1c4*** | 0,140055 | 2,312705 | 0,392458 | -1,04147 | -1,21843 |
| ***Lipc*** | -0,33561 | 0,056819 | 0,199002 | 0,359127 | 0,275048 |
| ***Irak1*** | 0,213248 | -0,22601 | 0,158575 | 0,292531 | 0,061371 |
| ***Stard5*** | 0,083151 | 0,345222 | 0,468553 | -0,09486 | -0,31932 |
| ***Rxrb*** | 0,457257 | 0,219044 | 0,120287 | -0,14449 | -0,17246 |
| ***Slc51a*** | -0,06567 | 0,345798 | 0,206038 | -0,09768 | 0,074967 |
| ***Clu*** | 0,169921 | 0,158111 | -0,1104 | -0,01523 | 0,119884 |
| ***Hras*** | 0,40146 | -0,23578 | -0,11532 | 0,234695 | 0,023973 |
| ***Cul3*** | 0,020882 | 0,207118 | 0,034457 | 0,08637 | -0,07733 |
| ***Slc9a3r2*** | 0,275201 | 0,14752 | -0,19208 | -0,07088 | 0,061981 |
| ***Ldlr*** | -0,19533 | -0,23531 | 0,05352 | 0,181182 | 0,380566 |
| ***Nfkbia*** | 0,010711 | 0,285474 | -0,04494 | -0,06765 | -0,0162 |
| ***Tnf*** | -0,18177 | -0,4173 | 0,296697 | 0,27893 | 0,183334 |
| ***Apoa2*** | -0,17361 | 0,299471 | 0,622124 | -0,05897 | -0,53274 |
| ***Scarb1*** | -0,17737 | 0,467088 | 0,007424 | 0,012039 | -0,19073 |
| ***Tspo*** | 0,199444 | -0,10564 | -0,08693 | -0,0227 | 0,093259 |
| ***Smpd1*** | 0,298504 | -0,25833 | -0,03771 | 0,003571 | 0,063222 |
| ***Akap13*** | 0,371115 | -0,58444 | 0,023724 | 0,158101 | 0,00517 |
| ***Arhgef11*** | 0,119541 | -0,26957 | -0,01881 | 0,049776 | 0,002864 |
| ***Srebf1*** | -0,75619 | 0,685746 | 0,064183 | -0,03825 | -0,08533 |
| ***Vps4b*** | -0,15861 | 0,143235 | -0,01297 | -0,07382 | -0,02915 |
| ***Apof*** | -0,10418 | 0,377569 | -0,25427 | -0,25009 | 0,07323 |
| ***Ces1*** | -0,12385 | 0,340526 | 0,219336 | 0,011072 | -0,6343 |
| ***Ttc39b*** | -0,21905 | 0,019599 | -0,13306 | 0,025097 | 0,10313 |
| ***Apoa1*** | 0,200219 | -0,07687 | -0,25979 | -0,08649 | 0,018263 |
| ***Hnf1a*** | 0,141772 | -0,46934 | 0,133149 | 0,073791 | -0,10086 |
| ***Rac1*** | 0,004156 | -0,36322 | 0,012891 | 0,016666 | 0,099646 |
| ***Hspa8*** | -0,01242 | -0,20995 | -0,02698 | -0,01897 | 0,028543 |
| ***Aqp8*** | -0,71873 | 0,170539 | 0,222727 | 0,155749 | -0,08264 |
| ***Ager*** | -0,37751 | 0,010039 | -0,07772 | -0,07408 | 0,26053 |
| ***Rhoa*** | -0,13676 | -0,1495 | 0,013621 | -0,03237 | 0,023586 |
| ***Scd2*** | -0,43642 | -0,0554 | -0,01182 | -0,0192 | 0,20793 |
| ***Ppara*** | 0,284157 | 0,016818 | -0,01524 | -0,10652 | -0,51136 |
| ***Abca5*** | 0,090601 | -0,44144 | -0,15628 | -0,00613 | 0,151655 |
| ***Apoe*** | 0,00429 | 0,122289 | -0,03966 | -0,30261 | -0,15475 |
| ***Lss*** | -0,37367 | -0,13491 | 0,009673 | -0,05388 | 0,161206 |
| ***Canx*** | 0,013316 | -0,27514 | -0,10962 | 0,000647 | -0,02993 |
| ***Serpina6*** | -0,73851 | 0,531935 | 0,070508 | -0,01966 | -0,2586 |
| ***Ptch1*** | 0,262288 | -0,12041 | 0,031938 | -0,26919 | -0,39448 |
| ***Ephx1*** | 0,253109 | -0,37561 | -0,06008 | -0,12743 | -0,2109 |
| ***Osbp*** | 0,216893 | -0,62377 | -0,11025 | -0,01803 | -0,07237 |
| ***Aqp9*** | -0,00116 | 0,275407 | -0,33264 | -0,34037 | -0,29478 |
| ***Stard3*** | -0,19645 | -0,28329 | -0,0961 | -0,05702 | -0,06698 |
| ***Tjp2*** | -0,26705 | -0,39514 | -0,14166 | -0,07741 | 0,158415 |
| ***Jak2*** | -0,02781 | -0,30249 | -0,25278 | 0,041496 | -0,19887 |
| ***Scd*** | -0,13432 | 0,289914 | 0,097704 | -0,38032 | -0,63388 |
| ***Gsn*** | -0,10068 | -0,48996 | -0,15483 | -0,00717 | -0,02146 |
| ***Npc2*** | -0,37777 | -0,36177 | -0,13016 | -0,07041 | 0,090643 |
| ***Pctp*** | -0,4482 | -0,24054 | 0,059516 | -0,06193 | -0,19305 |
| ***Mapk9*** | -0,00623 | -0,34585 | -0,12821 | -0,25198 | -0,15795 |
| ***Nr1h3*** | 0,045567 | -0,18155 | -0,28072 | -0,24717 | -0,25165 |
| ***Mttp*** | -0,05097 | -0,37223 | -0,1664 | -0,00399 | -0,37249 |
| ***Tert*** | -0,56719 | 0,007105 | -0,06652 | -0,35385 | -0,03381 |
| ***Lrp1*** | 0,060206 | -0,55842 | -0,2624 | -0,11997 | -0,17952 |
| ***Abcg5*** | -0,60954 | 0,480023 | 0,03509 | -0,40794 | -0,56029 |
| ***Il33*** | -0,29263 | 0,166507 | -0,72088 | -0,42045 | 0,177044 |
| ***Abcb11*** | -0,28694 | 0,895383 | -0,1812 | -0,56721 | -0,9809 |
| ***Abcc3*** | -0,1613 | -0,49806 | -0,1044 | -0,23767 | -0,1381 |
| ***Slco1b3*** | 0,060062 | 1,853445 | -0,18141 | -1,3031 | -1,57247 |
| ***Abcc2*** | 0,030509 | -0,50224 | -0,16845 | -0,25012 | -0,25719 |
| ***Anxa6*** | -0,23768 | -0,58634 | -0,27128 | -0,15766 | 0,020174 |
| ***Pcolce2*** | -0,01071 | 0,222022 | -0,40884 | -0,61582 | -0,51861 |
| ***Pld2*** | -0,14237 | -0,53318 | -0,49428 | -0,16364 | -0,00071 |
| ***Npc1*** | -0,24579 | 0,014381 | -0,29323 | -0,412 | -0,40126 |
| ***Slc10a1*** | -0,28589 | 0,904126 | 0,417748 | -0,66668 | -1,77778 |
| ***Acat1*** | 0,022855 | 0,265225 | -0,44318 | -0,69614 | -0,61754 |
| ***Soat2*** | -0,2558 | -0,20424 | -0,22728 | -0,25872 | -0,55695 |
| ***Apoa4*** | -0,46224 | -0,92768 | -0,32289 | -0,02632 | 0,2323 |
| ***Tp53*** | -0,03313 | -0,694 | -0,51593 | -0,13647 | -0,15904 |
| ***Pcyt1a*** | -0,15554 | -0,76428 | -0,24717 | -0,25354 | -0,17347 |
| ***Abcb4*** | 5,65E-05 | -0,36295 | -0,32981 | -0,44952 | -0,4935 |
| ***Sntb1*** | -0,02769 | -0,71682 | -0,66776 | -0,24059 | -0,082 |
| ***Nceh1*** | 0,132946 | -1,31178 | -0,44293 | -0,22489 | 0,021838 |
| ***Nr1h4*** | 0,255714 | -0,60739 | -0,50502 | -0,55488 | -0,43199 |
| ***Slc51b*** | -0,94048 | -0,25946 | -0,43251 | -0,19989 | -0,11032 |
| ***Apom*** | -0,33283 | -0,1507 | -0,93804 | -0,45161 | -0,17364 |
| ***Pon1*** | -0,20672 | 0,160498 | 0,102142 | -0,76871 | -1,36881 |
| ***App*** | -0,64282 | -0,94085 | -0,48604 | -0,276 | -0,02858 |
| ***Pltp*** | -0,54254 | -0,02021 | -0,53789 | -0,73388 | -0,55614 |
| ***Vldlr*** | -1,65317 | -0,33405 | 0,599907 | -0,31061 | -0,84224 |
| ***Lipa*** | -0,39884 | -1,07622 | -0,60296 | -0,43253 | -0,17624 |
| ***Pparg*** | 0,308282 | -1,26225 | -0,77772 | -0,62831 | -0,46886 |
| ***Pla1a*** | -0,50878 | -0,65272 | -0,89701 | -0,51342 | -0,26201 |
| ***Apcs*** | -0,71083 | -0,82579 | -1,01747 | -0,38636 | 0,102086 |
| ***Cyp7a1*** | -0,10229 | 1,351961 | -0,28199 | -1,67746 | -2,37028 |
| ***Slco1a2*** | -1,25718 | -0,22591 | -0,69486 | -0,50164 | -1,06208 |
| ***Abcb1b*** | -2,13704 | -1,12408 | -0,84069 | -0,67715 | -0,42022 |
| ***Abcc4*** | -0,77985 | -1,81574 | -1,03435 | -0,8991 | -1,03666 |
| ***Vnn1*** | -0,56203 | -1,95141 | -2,38867 | -1,60905 | -0,88811 |
| ***Abcb1*** | -3,15988 | -0,99045 | -1,36827 | -1,08103 | -0,80522 |
| ***Cd36*** | -1,76494 | -1,54792 | -2,50848 | -1,88358 | -1,04423 |
| ***Slco1a4*** | -1,59891 | -1,37986 | -2,1885 | -2,29899 | -2,58894 |

**Table S20:** Activation Z-score of genes involved in the concentration of fatty acids male vs. female. Activation Z-score was calculated with IPA software from Qiagen.

| **Pathway Summary** | 0,209 | 0,052 | 0,756 | 1,305 | -0,756 |
| --- | --- | --- | --- | --- | --- |
| **genes in the concentration of fatty acid network** | **0 h** | **24 h** | **48 h** | **72 h** | **96 h** |
| ***Elovl3*** | 5,046876 | 6,186568 | 2,899638 | 1,088746 | 0,664992 |
| ***Fos*** | 0,477946 | 1,616629 | 1,925766 | 1,628887 | 1,742349 |
| ***Ndufa4l2*** | 0,132229 | 0,911015 | 1,788626 | 2,336622 | 0,905423 |
| ***Serpina1*** | 1,141492 | 1,2407 | 1,497335 | 0,705409 | 0,48324 |
| ***Scp2*** | 0,905224 | 2,031537 | 0,985545 | 0,350656 | 0,253287 |
| ***Cyp2e1*** | 0,155034 | 0,940182 | 2,273941 | 0,984777 | -0,50221 |
| ***Hmox1*** | 0,452339 | 0,564182 | 1,329974 | 0,634012 | 0,537807 |
| ***Fabp4*** | -0,12234 | 1,045039 | 1,446707 | 0,929734 | 0,149975 |
| ***Slc2a1*** | 0,247876 | 0,605368 | 0,992706 | 0,942282 | 0,601681 |
| ***Fabp1*** | 0,377904 | 2,480121 | 1,289524 | -0,39195 | -0,87061 |
| ***Egfr*** | 1,511161 | 0,917405 | 0,196501 | 0,031613 | 0,151305 |
| ***Cav1*** | 0,773773 | 0,658437 | 0,150822 | 0,097915 | 0,546004 |
| ***Nts*** | 0,038556 | 0,300871 | 0,855685 | 0,633089 | 0,333839 |
| ***Cebpb*** | 0,197873 | 0,933786 | 0,337535 | 0,344411 | 0,258395 |
| ***Hcar2*** | 0,07692 | 1,692572 | 0,145806 | -0,05036 | -0,05117 |
| ***Ptges*** | -0,40226 | 0,469452 | 1,145467 | 0,366267 | 0,178699 |
| ***Igf1*** | -0,0711 | 0,534636 | 0,478904 | 0,342446 | 0,463394 |
| ***Sstr2*** | 0,719295 | 0,582043 | 0,353417 | 0,025651 | 0,047641 |
| ***Cyp1a2*** | 0,066898 | 1,509168 | 1,215758 | 0,619908 | -1,80321 |
| ***Abcg2*** | 1,022892 | -0,17177 | -0,15845 | 0,248904 | 0,622251 |
| ***Rgs4*** | 0,147076 | 0,324701 | 0,293039 | 0,181583 | 0,59948 |
| ***Bdnf*** | 0,244529 | 0,182207 | 0,014395 | 0,402227 | 0,692226 |
| ***P2rx4*** | 0,381128 | 0,163489 | 0,25108 | 0,111622 | 0,544992 |
| ***Tgfbr2*** | 0,22758 | 0,159388 | 0,48169 | 0,38976 | 0,164781 |
| ***Phyh*** | 0,283241 | 1,296553 | 0,151206 | -0,14818 | -0,18579 |
| ***Cyp27a1*** | 0,099837 | 0,68324 | 0,612828 | 0,138729 | -0,20747 |
| ***Kdm3a*** | 0,138353 | 0,593175 | 0,601079 | 0,264674 | -0,31629 |
| ***Ppard*** | 0,035931 | 0,504734 | 0,246923 | 0,193196 | 0,287906 |
| ***Ptger4*** | 0,153896 | 0,428614 | 0,404995 | 0,173284 | 0,085753 |
| ***Sirt1*** | -0,0669 | 0,763695 | 0,318872 | 0,227024 | -0,0192 |
| ***Avp*** | 0,105017 | 0,100225 | 0,215471 | 0,348399 | 0,448057 |
| ***Acsl1*** | 0,275928 | 1,416515 | 0,136382 | -0,25224 | -0,37723 |
| ***Pla2g5*** | 0,00819 | 0,536913 | 0,324362 | 0,118147 | 0,210228 |
| ***Gnai1*** | 0,24009 | 0,085879 | 0,3033 | 0,310778 | 0,25472 |
| ***Cebpa*** | 0,565119 | 1,128915 | 0,105326 | -0,23774 | -0,37059 |
| ***Pex11a*** | 0,683714 | 0,143333 | 0,334841 | 0,140108 | -0,11715 |
| ***Mmp9*** | 0,170831 | 0,469305 | 0,34465 | 0,001376 | 0,194084 |
| ***Pgam2*** | 0,06039 | 0,218658 | 0,299257 | 0,171652 | 0,358826 |
| ***Lpl*** | 0,511842 | 0,147046 | 0,090463 | 0,161693 | 0,189924 |
| ***Efna2*** | 0,533068 | 0,256092 | 0,053721 | 0,014407 | 0,22662 |
| ***Daglb*** | 0,399863 | -0,18166 | 0,350153 | 0,305537 | 0,206685 |
| ***Tgfa*** | 0,387056 | 0,210526 | 0,146732 | 0,097411 | 0,206395 |
| ***Ephx2*** | 0,424815 | 0,609694 | -0,06428 | 0,03513 | 0,023852 |
| ***Thrsp*** | -0,07521 | 1,394794 | 0,232433 | -0,05143 | -0,476 |
| ***Epm2a*** | -0,25402 | 0,441024 | 0,401765 | 0,297824 | 0,114253 |
| ***Ins1*** | -0,15546 | 0,311846 | 0,152701 | 0,398085 | 0,245218 |
| ***Alox5*** | 0,097119 | 0,514201 | -0,04708 | 0,266093 | 0,107334 |
| ***Plin1*** | 0,023749 | 0,189395 | 0,063837 | 0,152108 | 0,479431 |
| ***Ptgis*** | -5E-06 | 0,265702 | 0,309336 | 0,12827 | 0,199699 |
| ***Yap1*** | 0,474368 | 0,230755 | -0,04042 | 0,156288 | 0,063745 |
| ***Oxt*** | 0,135888 | 0,12567 | 0,109471 | 0,174866 | 0,337703 |
| ***Sat1*** | -0,33138 | 0,393837 | 0,114153 | 0,278872 | 0,398208 |
| ***Nos1*** | 0,124981 | 0,244935 | 0,146299 | 0,011221 | 0,280653 |
| ***Cyp1a1*** | -0,0377 | 0,221035 | 0,38464 | 0,078008 | 0,130361 |
| ***F2*** | 0,060368 | 0,238874 | 0,224434 | 0,151489 | 0,097842 |
| ***Abca4*** | -0,22516 | 0,274006 | 0,336817 | 0,065528 | 0,289708 |
| ***Sod1*** | -0,00286 | 0,235516 | 0,313475 | 0,189561 | -0,0042 |
| ***Adrb3*** | 0,440709 | 0,256087 | 0,119164 | 0,018807 | -0,11038 |
| ***Il1b*** | 0,263769 | 0,310882 | -0,03707 | 0,045763 | 0,139904 |
| ***Cidea*** | 0,047785 | -0,07975 | 0,416233 | 0,033413 | 0,292272 |
| ***Ptgdr2*** | 0,038371 | 0,49403 | 0,040428 | -0,03706 | 0,148339 |
| ***Lpin1*** | 0,304783 | 0,830156 | 0,490199 | -0,31185 | -0,64224 |
| ***Hsd3b7*** | 0,077291 | 0,404465 | 0,271247 | -0,00801 | -0,07627 |
| ***Cntf*** | 0,152345 | 0,005822 | 0,321655 | 0,103383 | 0,070471 |
| ***Gnrh1*** | 0,198382 | -0,29813 | 0,317144 | 0,157381 | 0,256163 |
| ***Pla2g10*** | -0,24089 | 0,31235 | 0,127281 | 0,119424 | 0,250417 |
| ***Hs6st1*** | 0,087558 | -0,02053 | 0,183297 | 0,348937 | -0,04189 |
| ***Adgrf5*** | -0,44011 | 0,284782 | 0,45173 | 0,370797 | -0,11611 |
| ***Kat5*** | 0,062314 | 0,18536 | 0,136812 | 0,11235 | 0,05349 |
| ***Abhd5*** | -0,06357 | 0,116803 | 0,159989 | 0,048191 | 0,257255 |
| ***Snca*** | -0,42627 | 0,151173 | 0,30492 | 0,166235 | 0,313848 |
| ***Cidec*** | 0,307489 | -0,93111 | 0,683086 | 0,239585 | 0,210129 |
| ***Oga*** | 0,39386 | 0,101236 | -0,02562 | -0,06272 | 0,09608 |
| ***Ppargc1b*** | 0,222911 | 0,302329 | 0,065047 | -0,02786 | -0,06079 |
| ***E4f1*** | -0,18629 | 0,620178 | 0,087937 | -0,03681 | 0,005259 |
| ***Scap*** | -0,00978 | 0,275538 | 0,275419 | 0,015266 | -0,06669 |
| ***Ins*** | -0,33103 | 0,458564 | 0,005813 | -0,03666 | 0,391596 |
| ***Lpar3*** | 0,328185 | 0,113734 | 0,042901 | 0,17195 | -0,175 |
| ***Angptl4*** | 0,581837 | 0,508041 | -0,05698 | -0,22451 | -0,35668 |
| ***Adcyap1*** | -0,1195 | 0,290491 | -0,08756 | 0,037486 | 0,296229 |
| ***Zc3h10*** | -0,20181 | 0,294775 | 0,081469 | 0,116513 | 0,125626 |
| ***Slc27a4*** | 0,152291 | -0,23916 | 0,166582 | 0,259891 | 0,071442 |
| ***Cbl*** | -0,16746 | 0,399345 | 0,120721 | 0,156473 | -0,10746 |
| ***Cbs/cbsl*** | 0,047661 | 0,340664 | -0,1621 | -0,00901 | 0,184036 |
| ***Gpd2*** | 0,076561 | -0,02297 | -0,04922 | 0,139018 | 0,244428 |
| ***Kap*** | 0,112993 | 0,336205 | -0,00452 | -0,01631 | -0,06658 |
| ***Nos2*** | 0,047766 | 0,196393 | 0,014087 | -0,16033 | 0,249916 |
| ***Dnm2*** | 0,177527 | -0,06997 | 0,044639 | 0,094817 | 0,092828 |
| ***Il9*** | -0,04555 | 0,101348 | -0,07837 | -0,04608 | 0,403974 |
| ***Slc1a1*** | -0,25939 | 0,162354 | 0,024637 | 0,197187 | 0,201651 |
| ***Hint1*** | 0,281629 | -0,27312 | 0,256025 | 0,104908 | -0,06904 |
| ***Gnas*** | 0,157345 | -0,00359 | 0,066071 | 0,077306 | -0,00319 |
| ***Fgl1*** | -0,60514 | -0,19903 | 0,468235 | 0,291501 | 0,295674 |
| ***Crtc3*** | -0,01524 | 0,346289 | -0,02403 | 0,105226 | -0,1638 |
| ***Npy1r*** | 0,247693 | 0,041606 | 0,077397 | -0,00081 | -0,12691 |
| ***Ghrl*** | -0,25736 | 0,238318 | 0,189848 | 0,054328 | -0,0071 |
| ***Cln3*** | -0,23828 | -0,25091 | 0,383911 | 0,179902 | 0,135794 |
| ***Eci1*** | 0,191785 | 0,457583 | -0,13468 | -0,0355 | -0,27868 |
| ***Ghr*** | 0,020345 | 0,862462 | -0,07016 | -0,35706 | -0,26179 |
| ***Ldlr*** | -0,19533 | -0,23531 | 0,05352 | 0,181182 | 0,380566 |
| ***Ell*** | 0,334325 | 0,054944 | 0,075763 | -0,08828 | -0,19445 |
| ***Fads2*** | -0,2342 | 0,280332 | 0,08521 | 0,085893 | -0,03826 |
| ***Crhr2*** | -0,209 | 0,272133 | 0,154132 | -0,12835 | 0,073522 |
| ***Tnf*** | -0,18177 | -0,4173 | 0,296697 | 0,27893 | 0,183334 |
| ***Apoa2*** | -0,17361 | 0,299471 | 0,622124 | -0,05897 | -0,53274 |
| ***Cerk*** | 0,37678 | -0,40871 | 0,24643 | -0,13216 | 0,053874 |
| ***Rxra*** | 0,193827 | 0,169862 | 0,015074 | 0,019906 | -0,27327 |
| ***Scarb1*** | -0,17737 | 0,467088 | 0,007424 | 0,012039 | -0,19073 |
| ***Nr3c1*** | -0,18234 | 0,22316 | 0,147401 | 0,048842 | -0,12813 |
| ***Slc25a13*** | -0,13467 | -0,22342 | 0,44586 | 0,19267 | -0,18293 |
| ***Hsd17b12*** | 0,157407 | -0,54123 | 0,098661 | 0,176826 | 0,198638 |
| ***Pnpla2*** | 0,247679 | -0,29319 | 0,098474 | 0,152412 | -0,12929 |
| ***Ptges3*** | -0,09485 | -0,13093 | 0,394845 | 0,005238 | -0,10124 |
| ***Loc102724788/prodh*** | -0,30097 | 0,39402 | -0,07611 | -0,03998 | 0,084271 |
| ***Htt*** | -0,11077 | -0,14556 | 0,015363 | 0,065637 | 0,222904 |
| ***Sirt3*** | 0,047248 | 0,309398 | 0,022808 | -0,15282 | -0,19995 |
| ***Ahr*** | -0,14658 | 0,313906 | -0,04025 | -0,03685 | -0,07687 |
| ***Cartpt*** | -0,24809 | 0,105997 | 0,057639 | -0,16222 | 0,236838 |
| ***Adora1*** | -0,02515 | 0,460472 | 0,296181 | -0,19368 | -0,5675 |
| ***G6pd*** | -0,91356 | -0,81963 | 0,563423 | 0,760106 | 0,3419 |
| ***Sirt4*** | -0,21548 | 0,198685 | 0,044321 | -0,10078 | 0,000804 |
| ***Bscl2*** | 0,024949 | 0,242515 | -0,16808 | -0,0596 | -0,11227 |
| ***Extl1*** | -0,37672 | 0,136722 | 0,000479 | 0,042108 | 0,121939 |
| ***Camp*** | -0,3413 | 0,170226 | 0,074399 | 0,066094 | -0,05043 |
| ***Prkci*** | 0,024551 | -0,43889 | 0,186525 | 0,082533 | 0,04227 |
| ***Slc6a11*** | -0,24472 | -0,0248 | -0,00414 | -0,11504 | 0,282429 |
| ***Srebf1*** | -0,75619 | 0,685746 | 0,064183 | -0,03825 | -0,08533 |
| ***Azgp1*** | -0,00885 | 0,382591 | -0,28781 | -0,18657 | -0,03767 |
| ***Angptl3*** | -0,01735 | 0,479563 | 0,012199 | 0,059137 | -0,67252 |
| ***C3*** | -0,17852 | 8,03E-05 | -0,12049 | 0,058399 | 0,081334 |
| ***Rock2*** | 0,295396 | -0,29271 | -0,18228 | -0,03032 | 0,026191 |
| ***Ces1*** | -0,12385 | 0,340526 | 0,219336 | 0,011072 | -0,6343 |
| ***Pon2*** | 0,295087 | -0,47237 | -0,19 | 0,01826 | 0,132092 |
| ***Hpgds*** | -0,43071 | 0,138671 | 0,02627 | 0,073397 | -0,04638 |
| ***Dbi*** | 0,109036 | 0,036403 | -0,24376 | -0,13536 | -0,03999 |
| ***Pex2*** | 0,068347 | -0,15174 | 0,080045 | -0,24601 | -0,03156 |
| ***Cysltr2*** | -0,32996 | -0,04093 | -0,02947 | 0,107027 | -0,00464 |
| ***Atg7*** | 0,193859 | 0,230731 | -0,31903 | -0,38279 | -0,02735 |
| ***Sptlc2*** | -0,31244 | -0,16422 | -0,25597 | 0,165366 | 0,255729 |
| ***Scd2*** | -0,43642 | -0,0554 | -0,01182 | -0,0192 | 0,20793 |
| ***Fgf7*** | -0,08165 | 0,136288 | -0,19965 | 0,163945 | -0,33884 |
| ***Ppara*** | 0,284157 | 0,016818 | -0,01524 | -0,10652 | -0,51136 |
| ***Gna11*** | 0,117817 | -0,38846 | -0,07016 | 0,053208 | -0,05876 |
| ***Sftpd*** | -0,16552 | 0,098252 | -0,002 | -0,35054 | 0,067491 |
| ***Apoe*** | 0,00429 | 0,122289 | -0,03966 | -0,30261 | -0,15475 |
| ***Hadha*** | 0,048218 | -0,04795 | -0,04955 | -0,1482 | -0,18075 |
| ***Naa40*** | 0,166896 | -0,33088 | -0,10867 | -0,00717 | -0,10186 |
| ***Mbtps1*** | 0,062942 | 0,065451 | -0,41234 | -0,03739 | -0,08008 |
| ***Inpp5k*** | -0,04817 | -0,26143 | 0,047599 | 0,083098 | -0,2323 |
| ***Elovl5*** | -0,2241 | 0,445751 | -0,17497 | -0,27147 | -0,19038 |
| ***Pnpla8*** | -0,02232 | -0,0185 | 0,08681 | -0,16123 | -0,30148 |
| ***Pde3b*** | -0,06062 | 0,05197 | -0,11567 | -0,03503 | -0,27351 |
| ***Slc27a1*** | -0,41268 | 0,148235 | 0,088034 | -0,06771 | -0,20017 |
| ***Stat6*** | 0,273005 | -0,43594 | -0,2113 | -0,07114 | -0,00908 |
| ***Nos3*** | -0,44887 | 0,068903 | -0,05055 | -0,06996 | 0,013571 |
| ***Hspa5*** | 0,034901 | -0,31971 | -0,09675 | -0,05292 | -0,06005 |
| ***Insr*** | -0,04304 | -0,30847 | -0,01885 | -0,02239 | -0,12176 |
| ***Tardbp*** | -0,14653 | -0,25438 | -0,00405 | -0,06842 | -0,07168 |
| ***Slco2a1*** | 0,046009 | -1,01397 | -0,20624 | 0,30514 | 0,313243 |
| ***Efna5*** | -0,10752 | 0,368022 | -0,12622 | -0,34529 | -0,35821 |
| ***Prkaa2*** | 0,026958 | -0,16209 | -0,16135 | -0,10442 | -0,16972 |
| ***Igf2bp2*** | -0,19494 | 0,242714 | -0,23411 | -0,36201 | -0,02736 |
| ***Pten*** | -0,06088 | -0,2313 | -0,00484 | -0,14557 | -0,13593 |
| ***Xpa*** | -0,01753 | 0,002382 | -0,12123 | -0,31444 | -0,14139 |
| ***Akt1*** | -0,12742 | -0,47145 | -0,12531 | -0,0356 | 0,152021 |
| ***Hsd11b1*** | -0,18894 | 0,270435 | -0,05613 | -0,22592 | -0,44326 |
| ***Hrh1*** | -0,34244 | -0,13853 | 0,104488 | -0,22612 | -0,04703 |
| ***Tyk2*** | 0,010038 | -0,2758 | -0,26825 | -0,17562 | 0,040721 |
| ***Adra1b*** | -0,21175 | 0,299552 | -0,33611 | -0,34551 | -0,08002 |
| ***Tnfrsf1a*** | -0,28457 | -0,31858 | 0,02452 | 0,128835 | -0,232 |
| ***Acly*** | -0,53178 | -0,15754 | -0,04428 | 0,10239 | -0,05604 |
| ***Dgat2*** | -0,08642 | 0,211404 | -0,25964 | -0,24013 | -0,31512 |
| ***Acsl4*** | 0,022254 | -0,66087 | -0,26081 | -0,00454 | 0,213346 |
| ***Slc30a7*** | -0,0057 | -0,51473 | -0,06318 | -0,02453 | -0,08975 |
| ***Pank1*** | -0,19551 | 0,55274 | -0,18486 | -0,32048 | -0,55342 |
| ***Sirt5*** | 0,019741 | -0,08465 | -0,23853 | -0,18128 | -0,21806 |
| ***Bbs12*** | -0,37029 | -0,05847 | -0,14843 | -0,1092 | -0,02322 |
| ***Elmo1*** | 0,201524 | -0,76485 | 0,116796 | -0,22041 | -0,05777 |
| ***Amacr*** | 0,266114 | 0,465913 | -0,78026 | -0,35673 | -0,34628 |
| ***Il18bp*** | -0,67711 | -0,15449 | -0,15196 | 0,01522 | 0,207936 |
| ***Scd*** | -0,13432 | 0,289914 | 0,097704 | -0,38032 | -0,63388 |
| ***Pank2*** | -0,15873 | -0,06449 | -0,2655 | -0,14184 | -0,14488 |
| ***Crat*** | -0,13528 | -0,3682 | -0,04971 | -0,08027 | -0,14379 |
| ***Arv1*** | -0,01046 | -0,2308 | -0,34189 | -0,03724 | -0,161 |
| ***Klb*** | -0,33321 | 0,761566 | -0,40744 | -0,33194 | -0,47152 |
| ***Pck1*** | 0,022757 | 2,085707 | -0,98927 | -1,0657 | -0,8563 |
| ***Acot13*** | -0,20654 | -0,48348 | -0,03027 | -0,11397 | 0,00707 |
| ***Decr1*** | 0,10697 | 0,084374 | -0,32973 | -0,45541 | -0,24776 |
| ***Ccl2*** | 0,066708 | -1,28911 | -0,39182 | 0,091752 | 0,678362 |
| ***Ppp1r3c*** | -0,20615 | 0,757792 | 0,253787 | -0,85739 | -0,80804 |
| ***Nuak2*** | 0,406218 | -0,31669 | -0,24485 | -0,30523 | -0,41702 |
| ***Pctp*** | -0,4482 | -0,24054 | 0,059516 | -0,06193 | -0,19305 |
| ***Faah*** | 0,061569 | 0,020827 | -0,45332 | -0,27998 | -0,24073 |
| ***Aifm1*** | -0,08824 | -0,35303 | -0,21819 | -0,20021 | -0,06116 |
| ***Plin2*** | 0,099249 | -0,88332 | -0,13492 | -0,13418 | 0,130005 |
| ***Acadl*** | -0,20704 | -0,55124 | -0,02583 | -0,07497 | -0,06891 |
| ***Pdk4*** | -0,7756 | 0,003353 | 0,254408 | -0,1653 | -0,2616 |
| ***Mttp*** | -0,05097 | -0,37223 | -0,1664 | -0,00399 | -0,37249 |
| ***Hadh*** | -0,2833 | 0,412573 | -0,43077 | -0,41483 | -0,27304 |
| ***Mboat7*** | -0,18279 | -0,3159 | -0,03117 | -0,31255 | -0,15441 |
| ***Taz*** | 0,05904 | -0,26099 | -0,11091 | -0,34407 | -0,34664 |
| ***Idh1*** | -0,07333 | -0,07805 | -0,38205 | -0,35781 | -0,13272 |
| ***Cideb*** | -0,0803 | -0,06647 | -0,34073 | -0,26516 | -0,27135 |
| ***Hsd17b4*** | 0,162203 | -0,53786 | -0,16633 | -0,25361 | -0,24445 |
| ***Lrp1*** | 0,060206 | -0,55842 | -0,2624 | -0,11997 | -0,17952 |
| ***Adrb2*** | -0,01087 | -0,54006 | -0,22739 | -0,07428 | -0,2077 |
| ***Oxsm*** | -0,11358 | -0,3831 | -0,20943 | -0,24405 | -0,13282 |
| ***Erbb2*** | -0,10815 | -0,01312 | -0,46978 | -0,24009 | -0,26202 |
| ***Kras*** | -0,26815 | -0,15401 | -0,19245 | -0,26561 | -0,23842 |
| ***Fabp5*** | -0,71247 | 0,616043 | -0,73515 | -0,22654 | -0,06958 |
| ***Thrb*** | 0,064914 | -0,58191 | -0,25835 | -0,15244 | -0,24924 |
| ***Pfkfb1*** | 0,426791 | -0,72513 | -0,32207 | -0,16839 | -0,39845 |
| ***Ogt*** | -0,34307 | -0,25212 | -0,20671 | -0,23484 | -0,18719 |
| ***Abcd1*** | -0,49221 | 0,088577 | -0,0985 | -0,43144 | -0,32268 |
| ***Tlr2*** | -0,22506 | -0,32334 | -0,54692 | -0,18222 | 0,019467 |
| ***Elovl2*** | -0,1327 | 0,596818 | -0,41057 | -0,5967 | -0,77845 |
| ***Cpt1a*** | 0,141407 | -0,3976 | -0,44114 | -0,41383 | -0,24206 |
| ***Xdh*** | -0,18054 | -0,28762 | -0,22192 | -0,2607 | -0,40273 |
| ***Pml*** | -0,15464 | -0,33835 | -0,49261 | -0,32207 | -0,08209 |
| ***Ehhadh*** | -0,21017 | -0,18064 | -0,0393 | -0,47618 | -0,4945 |
| ***Il1rn*** | -0,75085 | 0,012076 | -0,49055 | 0,042444 | -0,23981 |
| ***Pon3*** | -0,08 | -0,52909 | -0,44654 | -0,28606 | -0,10196 |
| ***Agpat2*** | -0,03707 | -0,32925 | -0,47236 | -0,26184 | -0,35562 |
| ***Agt*** | -0,32361 | 0,085077 | -0,52515 | -0,41596 | -0,29566 |
| ***Prdx3*** | -0,23881 | -0,69977 | -0,2737 | -0,20182 | -0,07735 |
| ***Il15ra*** | 0,1906 | -0,62726 | -0,39665 | -0,40524 | -0,27123 |
| ***Il18*** | -0,15491 | 0,211891 | -0,75962 | -0,60076 | -0,21168 |
| ***Plscr3*** | -0,12548 | -0,40818 | -0,56052 | -0,32793 | -0,15016 |
| ***Fasn*** | -0,78593 | -0,47232 | -0,08825 | 0,042331 | -0,31419 |
| ***Gpam*** | -0,22696 | -0,85832 | -0,13764 | -0,24223 | -0,15404 |
| ***Kng1*** | -0,41652 | -0,33195 | -0,41554 | -0,32952 | -0,19744 |
| ***Akr1b1*** | 0,044134 | -0,50866 | -0,45581 | -0,40216 | -0,37285 |
| ***Gk*** | -0,33561 | 0,276574 | -0,57982 | -0,56334 | -0,51153 |
| ***Napepld*** | -0,19458 | -1,27637 | -0,57216 | 0,121144 | 0,160326 |
| ***Snrk*** | -0,34232 | -0,39845 | -0,24646 | -0,33381 | -0,48073 |
| ***Txnip*** | 0,181313 | 0,06371 | 0,261129 | -1,08844 | -1,22374 |
| ***Irs1*** | -0,02384 | -0,72107 | -0,35943 | -0,17462 | -0,53636 |
| ***Nceh1*** | 0,132946 | -1,31178 | -0,44293 | -0,22489 | 0,021838 |
| ***Nr1h4*** | 0,255714 | -0,60739 | -0,50502 | -0,55488 | -0,43199 |
| ***Cxcl12*** | -0,31424 | 0,331944 | -0,47419 | -0,75144 | -0,67271 |
| ***Ucp2*** | 0,348379 | -0,17992 | -1,08821 | -0,76601 | -0,25163 |
| ***Hpgd*** | -0,56275 | -0,65989 | 0,03402 | -0,31819 | -0,44264 |
| ***Arntl*** | -0,28962 | -1,1286 | -0,28489 | -0,08829 | -0,24788 |
| ***Plaat3*** | -0,25542 | -0,37084 | -0,45793 | -0,60211 | -0,38193 |
| ***Pon1*** | -0,20672 | 0,160498 | 0,102142 | -0,76871 | -1,36881 |
| ***Mertk*** | -0,64951 | -0,62557 | -0,2044 | 0,043208 | -0,66573 |
| ***Gck*** | -0,2082 | 0,481251 | -1,06026 | -0,70313 | -0,69032 |
| ***App*** | -0,64282 | -0,94085 | -0,48604 | -0,276 | -0,02858 |
| ***Mgll*** | -0,06135 | -0,14408 | -0,90209 | -0,639 | -0,63762 |
| ***Pltp*** | -0,54254 | -0,02021 | -0,53789 | -0,73388 | -0,55614 |
| ***Vldlr*** | -1,65317 | -0,33405 | 0,599907 | -0,31061 | -0,84224 |
| ***Lipa*** | -0,39884 | -1,07622 | -0,60296 | -0,43253 | -0,17624 |
| ***Ppargc1a*** | -0,79412 | -0,38501 | -0,49487 | -0,42794 | -0,66493 |
| ***Mfsd2a*** | -0,98722 | -0,83224 | -0,51057 | -0,13327 | -0,3093 |
| ***Pparg*** | 0,308282 | -1,26225 | -0,77772 | -0,62831 | -0,46886 |
| ***Acaca*** | -0,81725 | -0,50812 | -0,39237 | -0,72828 | -0,71412 |
| ***G6pc*** | -0,13059 | 0,340476 | -0,01617 | -1,58284 | -2,08482 |
| ***Slc2a2*** | -0,29992 | -0,64015 | -0,84233 | -1,01141 | -0,84855 |
| ***Ces1g*** | -0,50341 | 0,343339 | -0,57869 | -1,01469 | -1,90591 |
| ***Cyp4a11*** | -0,5732 | 0,642984 | -0,96937 | -1,57802 | -1,1825 |
| ***Ptgds*** | -1,88192 | -0,85589 | -0,79971 | -0,40544 | -0,41432 |
| ***Csf1*** | -0,26315 | -1,99638 | -1,44569 | -0,86568 | -0,10769 |
| ***Acot2*** | -0,6759 | -2,39627 | -1,1104 | -0,58939 | -0,27743 |
| ***Lepr*** | -1,18575 | -0,92697 | -0,63828 | -1,08262 | -1,81091 |
| ***Abcd2*** | -3,21303 | -0,4612 | -1,06522 | -1,32308 | -1,44821 |
| ***Cd36*** | -1,76494 | -1,54792 | -2,50848 | -1,88358 | -1,04423 |
| ***Fmo3*** | -5,79658 | -3,84383 | -4,39118 | -4,23965 | -3,20229 |

**Table S21:** Activation Z-score of genes involved in the concentration of lipids is male vs. female. Activation Z-score was calculated with IPA software from Qiagen.

| **Pathway Summary** | -0,644 | 0,518 | -1,026 | -0,264 | -1,609 |
| --- | --- | --- | --- | --- | --- |
| **genes in the concentration of lipid network** | **0 h** | **24 h** | **48 h** | **72 h** | **96 h** |
| ***Elovl3*** | 5,046876 | 6,186568 | 2,899638 | 1,088746 | 0,664992 |
| ***Slco1a1*** | 3,103735 | 4,931852 | 3,018541 | 2,176901 | 1,075615 |
| ***Fos*** | 0,477946 | 1,616629 | 1,925766 | 1,628887 | 1,742349 |
| ***Ndufa4l2*** | 0,132229 | 0,911015 | 1,788626 | 2,336622 | 0,905423 |
| ***Serpine1*** | -0,40088 | 0,778444 | 1,611431 | 2,051347 | 1,266997 |
| ***Atf3*** | 0,530338 | 1,134128 | 1,22024 | 1,094871 | 1,103943 |
| ***Serpina1*** | 1,141492 | 1,2407 | 1,497335 | 0,705409 | 0,48324 |
| ***Adm*** | -0,10817 | 1,401906 | 1,589678 | 1,394712 | 0,57117 |
| ***Saa1*** | -0,22883 | 0,807029 | 1,679516 | 1,794978 | 0,628602 |
| ***Serpina12*** | 1,818211 | 1,359641 | 0,496692 | 0,518742 | 0,347824 |
| ***Scp2*** | 0,905224 | 2,031537 | 0,985545 | 0,350656 | 0,253287 |
| ***Egr1*** | 0,200368 | 0,967559 | 1,098924 | 1,112761 | 1,004121 |
| ***Plcl1*** | -0,13783 | 0,992468 | 1,146341 | 1,245308 | 0,792632 |
| ***F2r*** | 1,338302 | 0,862247 | 0,770082 | 0,465858 | 0,599782 |
| ***Slc10a2*** | 0,620419 | 0,93608 | 0,591403 | 0,981331 | 0,854708 |
| ***Cyp2e1*** | 0,155034 | 0,940182 | 2,273941 | 0,984777 | -0,50221 |
| ***Dio3*** | 0,114912 | 0,092035 | 1,429579 | 1,216279 | 0,800777 |
| ***Hmox1*** | 0,452339 | 0,564182 | 1,329974 | 0,634012 | 0,537807 |
| ***Fabp4*** | -0,12234 | 1,045039 | 1,446707 | 0,929734 | 0,149975 |
| ***Slc2a1*** | 0,247876 | 0,605368 | 0,992706 | 0,942282 | 0,601681 |
| ***Il1r1*** | 1,076155 | 1,553913 | -0,1904 | 0,443291 | 0,453751 |
| ***Trib1*** | 0,651969 | 1,055379 | 0,313726 | 0,517737 | 0,678966 |
| ***Apobec1*** | 1,068508 | 0,595942 | 0,522453 | 0,411619 | 0,364742 |
| ***Fabp1*** | 0,377904 | 2,480121 | 1,289524 | -0,39195 | -0,87061 |
| ***Jun*** | 0,719868 | 0,883187 | 0,549306 | 0,296255 | 0,375915 |
| ***Egfr*** | 1,511161 | 0,917405 | 0,196501 | 0,031613 | 0,151305 |
| ***Vegfa*** | -0,06545 | 0,80094 | 0,960118 | 0,861856 | 0,106438 |
| ***Cpe*** | -0,27018 | -0,21873 | 0,754673 | 1,116941 | 1,178173 |
| ***Gulo*** | 0,061363 | 1,195343 | 0,58747 | 0,447976 | 0,181136 |
| ***Lgals3*** | -0,20388 | 1,006761 | -0,00589 | 0,613036 | 0,930013 |
| ***Brd2*** | 0,267152 | 0,532132 | 0,458618 | 0,526304 | 0,486191 |
| ***Cav1*** | 0,773773 | 0,658437 | 0,150822 | 0,097915 | 0,546004 |
| ***Sort1*** | 0,866583 | 0,277318 | 0,31254 | 0,372022 | 0,372949 |
| ***Nts*** | 0,038556 | 0,300871 | 0,855685 | 0,633089 | 0,333839 |
| ***Cebpb*** | 0,197873 | 0,933786 | 0,337535 | 0,344411 | 0,258395 |
| ***Cyp8b1*** | 0,538241 | 1,700563 | 1,224542 | -0,39323 | -1,03816 |
| ***Nfil3*** | 0,365117 | 0,769658 | 0,357472 | 0,323207 | 0,144676 |
| ***Tgm2*** | -0,0872 | 0,169539 | 0,582522 | 0,639144 | 0,547246 |
| ***Hcar2*** | 0,07692 | 1,692572 | 0,145806 | -0,05036 | -0,05117 |
| ***Spp1*** | 0,356681 | 0,816593 | -0,63844 | 0,132484 | 1,13767 |
| ***Ptges*** | -0,40226 | 0,469452 | 1,145467 | 0,366267 | 0,178699 |
| ***Igf1*** | -0,0711 | 0,534636 | 0,478904 | 0,342446 | 0,463394 |
| ***Npy*** | 0,211691 | 0,403351 | 0,386468 | 0,485626 | 0,242127 |
| ***Sstr2*** | 0,719295 | 0,582043 | 0,353417 | 0,025651 | 0,047641 |
| ***Tac1*** | 0,27023 | 0,636432 | 0,47235 | 0,056696 | 0,253429 |
| ***Hmga1*** | 0,076572 | -0,20427 | 0,643688 | 0,860151 | 0,281759 |
| ***Dio1*** | 0,208914 | 1,159571 | 0,082653 | 0,202437 | -3,4E-05 |
| ***Sgk1*** | -1,18774 | 0,746598 | 0,813101 | 0,766849 | 0,51218 |
| ***Cyp1a2*** | 0,066898 | 1,509168 | 1,215758 | 0,619908 | -1,80321 |
| ***Tlr4*** | -0,14703 | -0,00808 | 0,78129 | 0,494066 | 0,451944 |
| ***Abcg2*** | 1,022892 | -0,17177 | -0,15845 | 0,248904 | 0,622251 |
| ***Kitlg*** | -0,02606 | 1,06252 | 0,234838 | 0,176335 | 0,112236 |
| ***Rgs4*** | 0,147076 | 0,324701 | 0,293039 | 0,181583 | 0,59948 |
| ***Fabp2*** | 0,430475 | 1,37847 | -0,22077 | -0,13115 | 0,084636 |
| ***Bdnf*** | 0,244529 | 0,182207 | 0,014395 | 0,402227 | 0,692226 |
| ***P2rx4*** | 0,381128 | 0,163489 | 0,25108 | 0,111622 | 0,544992 |
| ***Tgfbr2*** | 0,22758 | 0,159388 | 0,48169 | 0,38976 | 0,164781 |
| ***Apoa5*** | -0,00171 | 1,200094 | 0,038027 | 0,117437 | 0,054655 |
| ***Phyh*** | 0,283241 | 1,296553 | 0,151206 | -0,14818 | -0,18579 |
| ***S100a8*** | 0,316813 | 0,389164 | 0,164116 | 0,207476 | 0,302258 |
| ***Scnn1a*** | -0,01385 | 0,869851 | 0,227544 | 0,234164 | 0,044117 |
| ***Nucb2*** | 0,75137 | -0,21708 | 0,317646 | 0,22747 | 0,259298 |
| ***Cyp27a1*** | 0,099837 | 0,68324 | 0,612828 | 0,138729 | -0,20747 |
| ***Chrm3*** | 0,070267 | 0,431198 | 0,312648 | 0,231237 | 0,281056 |
| ***Gcgr*** | 0,358078 | 0,957776 | 0,453834 | -0,14892 | -0,29726 |
| ***Rpe65*** | 0,075574 | 0,74217 | 0,201409 | 0,162774 | 0,128783 |
| ***Kdm3a*** | 0,138353 | 0,593175 | 0,601079 | 0,264674 | -0,31629 |
| ***Mapkapk2*** | 0,422276 | 0,184214 | 0,140233 | 0,328419 | 0,195745 |
| ***Ppard*** | 0,035931 | 0,504734 | 0,246923 | 0,193196 | 0,287906 |
| ***Slc1a2*** | -0,09227 | 0,545465 | 0,404099 | 0,127111 | 0,276297 |
| ***Ptger4*** | 0,153896 | 0,428614 | 0,404995 | 0,173284 | 0,085753 |
| ***Sirt1*** | -0,0669 | 0,763695 | 0,318872 | 0,227024 | -0,0192 |
| ***Cxcl14*** | 0,2448 | 0,390031 | 0,140508 | 0,156825 | 0,28947 |
| ***Avp*** | 0,105017 | 0,100225 | 0,215471 | 0,348399 | 0,448057 |
| ***Bmp7*** | 0,657034 | 0,453625 | -0,06168 | 0,081589 | 0,079248 |
| ***Il25*** | 0,095375 | -0,01305 | 0,212678 | 0,331942 | 0,577525 |
| ***Mif*** | 0,042114 | 0,031338 | 0,387972 | 0,552037 | 0,186744 |
| ***Acsl1*** | 0,275928 | 1,416515 | 0,136382 | -0,25224 | -0,37723 |
| ***Pla2g5*** | 0,00819 | 0,536913 | 0,324362 | 0,118147 | 0,210228 |
| ***Gnai1*** | 0,24009 | 0,085879 | 0,3033 | 0,310778 | 0,25472 |
| ***Cebpa*** | 0,565119 | 1,128915 | 0,105326 | -0,23774 | -0,37059 |
| ***Steap4*** | 0,210981 | 1,010364 | -0,21656 | -0,03034 | 0,213214 |
| ***Pex11a*** | 0,683714 | 0,143333 | 0,334841 | 0,140108 | -0,11715 |
| ***Mmp9*** | 0,170831 | 0,469305 | 0,34465 | 0,001376 | 0,194084 |
| ***Abcg8*** | -0,39334 | 0,727529 | 0,795213 | 0,269695 | -0,23699 |
| ***Runx1*** | -0,19519 | 0,095331 | 0,451294 | 0,511187 | 0,289887 |
| ***Dpp7*** | 0,423313 | 0,208382 | 0,150052 | 0,089273 | 0,272534 |
| ***Enpp2*** | 0,28131 | 1,556747 | -0,15064 | -0,2265 | -0,34825 |
| ***Pgam2*** | 0,06039 | 0,218658 | 0,299257 | 0,171652 | 0,358826 |
| ***Lpl*** | 0,511842 | 0,147046 | 0,090463 | 0,161693 | 0,189924 |
| ***Npr1*** | 0,224399 | 0,375116 | 0,130558 | 0,064464 | 0,301067 |
| ***Gpihbp1*** | 0,125463 | 0,410104 | 0,52057 | -0,14343 | 0,181107 |
| ***Kpna1*** | 0,43178 | -0,00429 | 0,411543 | 0,171575 | 0,081244 |
| ***Efna2*** | 0,533068 | 0,256092 | 0,053721 | 0,014407 | 0,22662 |
| ***Daglb*** | 0,399863 | -0,18166 | 0,350153 | 0,305537 | 0,206685 |
| ***Ffar4*** | -0,31033 | 0,062398 | 0,941238 | 0,43388 | -0,07842 |
| ***C5*** | 0,293912 | 1,290041 | -0,01347 | -0,21699 | -0,30486 |
| ***Tgfa*** | 0,387056 | 0,210526 | 0,146732 | 0,097411 | 0,206395 |
| ***Pde10a*** | -0,30072 | 0,216486 | 0,503411 | 0,267784 | 0,353929 |
| ***Lcat*** | -0,17959 | 0,579446 | 0,584129 | 0,271463 | -0,21531 |
| ***Mras*** | 0,174646 | 1,003178 | -0,15429 | -0,08634 | 0,102783 |
| ***Ephx2*** | 0,424815 | 0,609694 | -0,06428 | 0,03513 | 0,023852 |
| ***Golph3*** | 0,18172 | 0,357689 | 0,290936 | 0,024546 | 0,174293 |
| ***Star*** | -0,06788 | 0,067333 | 0,275318 | 0,342523 | 0,407752 |
| ***Thrsp*** | -0,07521 | 1,394794 | 0,232433 | -0,05143 | -0,476 |
| ***Pip5k1b*** | -0,1052 | 0,344318 | 0,046992 | 0,21469 | 0,522942 |
| ***Polg*** | 0,054702 | 0,088632 | 0,332825 | 0,303947 | 0,24021 |
| ***Epm2a*** | -0,25402 | 0,441024 | 0,401765 | 0,297824 | 0,114253 |
| ***Cry2*** | 0,092577 | 0,291065 | 0,500655 | 0,085334 | 0,027654 |
| ***Atf4*** | -0,07623 | 0,409375 | 0,25378 | 0,177375 | 0,221857 |
| ***Chka*** | 0,426351 | 0,16697 | -0,22775 | 0,077874 | 0,541396 |
| ***Map2k1*** | 0,13442 | 0,207022 | 0,290068 | 0,177577 | 0,173668 |
| ***Chga*** | 0,149858 | 0,576997 | 0,077503 | 0,222002 | -0,05262 |
| ***Ins1*** | -0,15546 | 0,311846 | 0,152701 | 0,398085 | 0,245218 |
| ***Slc22a1*** | 0,081045 | 0,96672 | 0,360792 | -0,08863 | -0,3697 |
| ***Inpp4a*** | 0,320868 | 0,314506 | 0,062814 | 0,200973 | 0,039548 |
| ***Alox5*** | 0,097119 | 0,514201 | -0,04708 | 0,266093 | 0,107334 |
| ***Gtf2ird1*** | 0,312966 | 0,514535 | -0,08062 | 0,028278 | 0,153623 |
| ***B4galt2*** | 0,182783 | 0,386771 | 0,10953 | 0,160859 | 0,079021 |
| ***Plin1*** | 0,023749 | 0,189395 | 0,063837 | 0,152108 | 0,479431 |
| ***Srd5a1*** | 0,553331 | 0,549495 | -0,07521 | -0,04915 | -0,07225 |
| ***Dlg2*** | 0,199088 | 0,039515 | 0,33588 | 0,116886 | 0,214669 |
| ***Ptgis*** | -5E-06 | 0,265702 | 0,309336 | 0,12827 | 0,199699 |
| ***Prkg1*** | -0,0166 | 0,263047 | 0,285348 | 0,037454 | 0,331461 |
| ***Spred2*** | 0,401181 | 0,078425 | 0,219958 | 0,091201 | 0,107266 |
| ***Lrat*** | -0,06049 | 0,208794 | 0,084343 | 0,359785 | 0,303068 |
| ***Neu1*** | 0,098679 | -0,2194 | 0,24831 | 0,317569 | 0,447105 |
| ***Yap1*** | 0,474368 | 0,230755 | -0,04042 | 0,156288 | 0,063745 |
| ***Oxt*** | 0,135888 | 0,12567 | 0,109471 | 0,174866 | 0,337703 |
| ***Pdk2*** | 0,128752 | 0,363429 | 0,460486 | 0,134277 | -0,20922 |
| ***Il17a*** | -0,14899 | 0,064948 | 0,589141 | 0,131666 | 0,233734 |
| ***Apoc1*** | 0,167749 | 0,812404 | 0,849487 | -0,26184 | -0,69744 |
| ***Esr2*** | -0,00057 | 0,018629 | 0,301336 | 0,113546 | 0,433366 |
| ***Slc13a5*** | -0,21528 | 0,850017 | 0,396288 | -0,07046 | -0,09864 |
| ***Sat1*** | -0,33138 | 0,393837 | 0,114153 | 0,278872 | 0,398208 |
| ***Crtc2*** | 0,079784 | 0,391697 | 0,155761 | 0,174674 | 0,040834 |
| ***Abhd6*** | 0,049894 | -0,21583 | 0,5008 | 0,346503 | 0,159471 |
| ***Mecp2*** | 0,101847 | 0,547118 | 0,096328 | 0,146568 | -0,05329 |
| ***Nsun2*** | 0,257022 | -0,08414 | 0,312976 | 0,227877 | 0,11047 |
| ***Kdm5b*** | 0,295004 | 0,503413 | 0,298959 | -0,18785 | -0,08925 |
| ***Nos1*** | 0,124981 | 0,244935 | 0,146299 | 0,011221 | 0,280653 |
| ***Gla*** | -0,25347 | -0,00908 | 0,31602 | 0,245103 | 0,504942 |
| ***Npc1l1*** | 0,22953 | 0,339241 | 0,066838 | 0,126331 | 0,037662 |
| ***Lrp5*** | -0,01761 | 0,203937 | 0,403935 | 0,285467 | -0,08711 |
| ***Gpr1*** | 0,066469 | 0,137167 | 0,089834 | 0,075921 | 0,414014 |
| ***Spns2*** | -0,53938 | -0,1185 | 0,477213 | 0,765442 | 0,198112 |
| ***Cyp1a1*** | -0,0377 | 0,221035 | 0,38464 | 0,078008 | 0,130361 |
| ***F2*** | 0,060368 | 0,238874 | 0,224434 | 0,151489 | 0,097842 |
| ***Cd4*** | 0,426659 | 0,284947 | -0,12784 | -0,09186 | 0,280133 |
| ***Pdgfrb*** | -0,09307 | 0,304447 | 0,26106 | 0,131816 | 0,161443 |
| ***Prl3d1*** | 0,128478 | -0,13321 | 0,283107 | -0,0148 | 0,502034 |
| ***Gatm*** | -0,05157 | 0,331376 | 0,199776 | -0,05124 | 0,333 |
| ***Tspyl5*** | 0,112538 | 0,396839 | 0,070951 | 0,110299 | 0,068513 |
| ***B4galt1*** | 0,149573 | 0,309996 | 0,244133 | 0,099127 | -0,04937 |
| ***Pip5kl1*** | 0,04201 | 0,114227 | 0,107806 | 0,130713 | 0,353853 |
| ***Abca4*** | -0,22516 | 0,274006 | 0,336817 | 0,065528 | 0,289708 |
| ***Fitm2*** | 0,328933 | 0,147821 | 0,42145 | 0,054906 | -0,21785 |
| ***Ndst3*** | 0,133059 | 0,19889 | 0,028859 | 0,32905 | 0,043503 |
| ***Sod1*** | -0,00286 | 0,235516 | 0,313475 | 0,189561 | -0,0042 |
| ***Lck*** | -0,01917 | 0,13758 | 0,239302 | 0,367501 | 0,003282 |
| ***Adrb3*** | 0,440709 | 0,256087 | 0,119164 | 0,018807 | -0,11038 |
| ***Il1b*** | 0,263769 | 0,310882 | -0,03707 | 0,045763 | 0,139904 |
| ***C1qa*** | 0,606322 | 0,203574 | 0,294951 | -0,26445 | -0,12163 |
| ***Mtmr3*** | 0,383555 | 0,206457 | -0,03727 | 0,114365 | 0,05136 |
| ***Cisd1*** | 0,247091 | 0,45393 | 0,076713 | 0,054741 | -0,11991 |
| ***Anp32b*** | -0,07882 | 0,242156 | 0,165695 | 0,220076 | 0,160992 |
| ***Cidea*** | 0,047785 | -0,07975 | 0,416233 | 0,033413 | 0,292272 |
| ***Prkcd*** | 0,074277 | 0,348892 | 0,246503 | 0,028518 | 0,008762 |
| ***Sphk1*** | 0,142443 | -0,11255 | 0,202839 | 0,136431 | 0,33292 |
| ***Mrap*** | 0,100434 | 0,006699 | 0,380563 | 0,197834 | 0,01638 |
| ***Ptgdr2*** | 0,038371 | 0,49403 | 0,040428 | -0,03706 | 0,148339 |
| ***Lpin1*** | 0,304783 | 0,830156 | 0,490199 | -0,31185 | -0,64224 |
| ***Hsd3b7*** | 0,077291 | 0,404465 | 0,271247 | -0,00801 | -0,07627 |
| ***Pafah1b1*** | 0,021576 | 0,182636 | 0,202084 | 0,218195 | 0,034888 |
| ***Cntf*** | 0,152345 | 0,005822 | 0,321655 | 0,103383 | 0,070471 |
| ***Fshb*** | 0,2641 | -0,03187 | -0,13345 | 0,504294 | 0,038416 |
| ***Grina*** | -0,16792 | 0,167773 | 0,398115 | 0,189325 | 0,049378 |
| ***Lipg*** | -0,59591 | 0,579194 | 0,290071 | 0,226069 | 0,131944 |
| ***Gnrh1*** | 0,198382 | -0,29813 | 0,317144 | 0,157381 | 0,256163 |
| ***Lhb*** | -0,04052 | 0,342593 | 0,237892 | -0,01577 | 0,103515 |
| ***Tg*** | -0,28613 | 0,184095 | 0,030879 | 0,203537 | 0,488864 |
| ***Hint2*** | 0,431696 | 0,541988 | 0,088866 | -0,11277 | -0,33827 |
| ***Cux1*** | 0,528188 | -0,1953 | 0,018576 | 0,235127 | 0,022676 |
| ***Rag2*** | 0,149637 | 0,378204 | -0,09636 | 0,073283 | 0,099775 |
| ***Por*** | -0,22385 | 0,116112 | 0,419562 | 0,346398 | -0,05581 |
| ***Cort*** | -0,17739 | 0,272676 | 0,12229 | 0,192127 | 0,184718 |
| ***Abca1*** | 0,121641 | 0,292213 | 0,159397 | 0,278491 | -0,26018 |
| ***Adra2a*** | -0,08752 | 0,116509 | 0,411827 | 0,001719 | 0,147574 |
| ***Tgif1*** | 0,290001 | 0,069619 | -0,12446 | 0,102239 | 0,235781 |
| ***Slc3a2*** | -0,15896 | 0,077229 | 0,320463 | 0,201231 | 0,13099 |
| ***Aqp3*** | 0,083718 | 0,419574 | 0,176862 | -0,02911 | -0,08146 |
| ***Pla2g10*** | -0,24089 | 0,31235 | 0,127281 | 0,119424 | 0,250417 |
| ***Traf3*** | 0,406177 | -0,26282 | 0,13948 | 0,264066 | 0,019655 |
| ***Sod2*** | 0,112468 | 0,368751 | 0,010485 | 0,083511 | -0,01597 |
| ***P2ry2*** | 0,434822 | 0,028217 | 0,224714 | 0,254641 | -0,38443 |
| ***Hs6st1*** | 0,087558 | -0,02053 | 0,183297 | 0,348937 | -0,04189 |
| ***Dhh*** | -0,15911 | 0,163838 | -0,10189 | 0,305226 | 0,347331 |
| ***Lipc*** | -0,33561 | 0,056819 | 0,199002 | 0,359127 | 0,275048 |
| ***Adgrf5*** | -0,44011 | 0,284782 | 0,45173 | 0,370797 | -0,11611 |
| ***Kat5*** | 0,062314 | 0,18536 | 0,136812 | 0,11235 | 0,05349 |
| ***Sgpp1*** | 0,166081 | 0,115594 | 0,221875 | 0,071908 | -0,02587 |
| ***Pth1r*** | -0,01084 | -0,06993 | 0,041336 | 0,276241 | 0,310089 |
| ***Galnt2*** | -0,02136 | 0,205236 | 0,174264 | 0,067285 | 0,115229 |
| ***Zbtb16*** | 0,120876 | 0,510964 | -0,06542 | 0,010297 | -0,03633 |
| ***Tnfrsf12a*** | 0,081349 | -0,04782 | 0,114627 | 0,093793 | 0,294744 |
| ***Rai1*** | -0,03154 | 0,238342 | 0,014509 | 0,096661 | 0,205066 |
| ***Alk*** | -0,1052 | 0,317195 | -0,11394 | 0,167914 | 0,253471 |
| ***Abhd5*** | -0,06357 | 0,116803 | 0,159989 | 0,048191 | 0,257255 |
| ***Sap18*** | -0,04806 | 0,106963 | 0,200816 | 0,213781 | 0,040042 |
| ***Pycard*** | -0,22804 | 0,519267 | 0,101148 | -0,12957 | 0,249948 |
| ***Snca*** | -0,42627 | 0,151173 | 0,30492 | 0,166235 | 0,313848 |
| ***Cidec*** | 0,307489 | -0,93111 | 0,683086 | 0,239585 | 0,210129 |
| ***Oga*** | 0,39386 | 0,101236 | -0,02562 | -0,06272 | 0,09608 |
| ***Ppargc1b*** | 0,222911 | 0,302329 | 0,065047 | -0,02786 | -0,06079 |
| ***Pmch*** | -0,04544 | 0,42489 | 0,053835 | 0,060474 | 0,00658 |
| ***Slc12a2*** | 0,051478 | 0,331979 | 0,055887 | 0,006698 | 0,046823 |
| ***Gpat4*** | 0,440138 | 0,220668 | -0,1771 | 0,012453 | -0,00366 |
| ***E4f1*** | -0,18629 | 0,620178 | 0,087937 | -0,03681 | 0,005259 |
| ***Scap*** | -0,00978 | 0,275538 | 0,275419 | 0,015266 | -0,06669 |
| ***Ins*** | -0,33103 | 0,458564 | 0,005813 | -0,03666 | 0,391596 |
| ***Aqp12a/aqp12b*** | -0,04483 | 0,178133 | 0,283906 | 0,005926 | 0,063571 |
| ***Lpar3*** | 0,328185 | 0,113734 | 0,042901 | 0,17195 | -0,175 |
| ***Ucn2*** | -0,19689 | 0,0193 | -0,03087 | 0,323517 | 0,362181 |
| ***Pik3c2a*** | 0,005251 | -0,19112 | 0,220942 | 0,330514 | 0,110934 |
| ***Prkar2b*** | -0,27859 | 0,108796 | 0,194686 | 0,282446 | 0,160946 |
| ***Slc51a*** | -0,06567 | 0,345798 | 0,206038 | -0,09768 | 0,074967 |
| ***Pla2g1b*** | 0,262145 | -0,0239 | -0,01338 | 0,010848 | 0,225401 |
| ***Asgr2*** | -0,2653 | 0,288239 | 0,280472 | 0,360083 | -0,20714 |
| ***H6pd*** | 0,451188 | 0,070876 | 0,019639 | -0,09563 | 0,009311 |
| ***Igf1r*** | -0,0981 | -0,0826 | 0,279362 | 0,110573 | 0,242523 |
| ***Angptl4*** | 0,581837 | 0,508041 | -0,05698 | -0,22451 | -0,35668 |
| ***Timp1*** | -0,14604 | 0,092355 | -0,10405 | 0,172475 | 0,431418 |
| ***Rln1*** | 0,056615 | 0,308993 | 0,108052 | -0,14209 | 0,107024 |
| ***Nrbf2*** | 0,143585 | 0,420956 | 0,114356 | -0,15393 | -0,08798 |
| ***Rrad*** | -0,15489 | -0,47769 | 0,357607 | 0,2649 | 0,444081 |
| ***Nppa*** | 0,034337 | 0,318987 | 0,116168 | -0,02375 | -0,01468 |
| ***Prlhr*** | 0,037706 | 0,042947 | 0,150854 | -0,04449 | 0,241512 |
| ***Adcyap1*** | -0,1195 | 0,290491 | -0,08756 | 0,037486 | 0,296229 |
| ***Zc3h10*** | -0,20181 | 0,294775 | 0,081469 | 0,116513 | 0,125626 |
| ***Fgfr4*** | -0,1769 | -0,4449 | 0,367973 | 0,487909 | 0,182102 |
| ***Pnpla5*** | -0,40347 | 0,177508 | 0,269477 | 0,224015 | 0,146383 |
| ***Ffar1*** | -0,07263 | 0,337083 | -0,11989 | -0,14563 | 0,412865 |
| ***Cyp11a1*** | -0,05775 | 0,377209 | 0,045666 | -0,05022 | 0,096446 |
| ***Slc27a4*** | 0,152291 | -0,23916 | 0,166582 | 0,259891 | 0,071442 |
| ***E2f1*** | 0,383477 | 0,036203 | -0,14638 | -0,01541 | 0,148558 |
| ***Adipor1*** | 0,056755 | -0,02311 | 0,123791 | 0,204018 | 0,041937 |
| ***Cbl*** | -0,16746 | 0,399345 | 0,120721 | 0,156473 | -0,10746 |
| ***Cbs/cbsl*** | 0,047661 | 0,340664 | -0,1621 | -0,00901 | 0,184036 |
| ***Ada*** | -0,15487 | 0,116629 | 0,033557 | 0,081439 | 0,323756 |
| ***Slc4a4*** | -0,29421 | -0,01695 | 0,0106 | 0,392826 | 0,300786 |
| ***Bbs10*** | 0,409935 | 0,114662 | -0,14764 | -0,03457 | 0,047301 |
| ***Gpd2*** | 0,076561 | -0,02297 | -0,04922 | 0,139018 | 0,244428 |
| ***Acp6*** | 0,00336 | 0,217946 | 0,265117 | 0,028942 | -0,12906 |
| ***Foxo3*** | 0,150165 | 0,280371 | 0,170242 | -0,06662 | -0,14944 |
| ***Trpv5*** | 0,078389 | 0,395147 | -0,08553 | -0,0178 | 0,00464 |
| ***Col4a3*** | 0,32682 | 0,168688 | -0,30258 | 0,090477 | 0,085998 |
| ***Th*** | -0,14832 | 0,407995 | 0,027018 | 0,189619 | -0,11209 |
| ***Kap*** | 0,112993 | 0,336205 | -0,00452 | -0,01631 | -0,06658 |
| ***Npy4r*** | -0,17818 | 0,327742 | 0,037374 | 0,067327 | 0,107044 |
| ***Fcer1g*** | 0,466088 | -0,02297 | 0,085672 | -0,05944 | -0,11566 |
| ***Xbp1*** | 0,283165 | 0,006163 | 0,028796 | 0,000284 | 0,033335 |
| ***Gata4*** | 0,013929 | 0,227963 | 0,004888 | -0,03094 | 0,134905 |
| ***Ep300*** | 0,223775 | -0,03702 | 0,045473 | 0,279534 | -0,16359 |
| ***Nos2*** | 0,047766 | 0,196393 | 0,014087 | -0,16033 | 0,249916 |
| ***Rgcc*** | -0,33256 | 0,233761 | 0,130112 | 0,124505 | 0,189548 |
| ***Dnm2*** | 0,177527 | -0,06997 | 0,044639 | 0,094817 | 0,092828 |
| ***Il9*** | -0,04555 | 0,101348 | -0,07837 | -0,04608 | 0,403974 |
| ***Stk25*** | 0,190238 | -0,10705 | 0,088566 | 0,103183 | 0,059687 |
| ***Sgpl1*** | -0,17167 | 0,147311 | 0,181794 | -0,02678 | 0,196809 |
| ***Pthlh*** | -0,32784 | 0,318648 | 0,083369 | 0,228069 | 0,024495 |
| ***Slc1a1*** | -0,25939 | 0,162354 | 0,024637 | 0,197187 | 0,201651 |
| ***Clu*** | 0,169921 | 0,158111 | -0,1104 | -0,01523 | 0,119884 |
| ***Atp2a2*** | 0,128012 | -0,22251 | 0,127965 | 0,20986 | 0,076742 |
| ***Src*** | -0,27544 | 0,024078 | 0,202661 | 0,159584 | 0,199357 |
| ***Hras*** | 0,40146 | -0,23578 | -0,11532 | 0,234695 | 0,023973 |
| ***Hint1*** | 0,281629 | -0,27312 | 0,256025 | 0,104908 | -0,06904 |
| ***Rgn*** | -0,25904 | 1,131845 | 0,357521 | -0,2515 | -0,68179 |
| ***Gnas*** | 0,157345 | -0,00359 | 0,066071 | 0,077306 | -0,00319 |
| ***Dio2*** | 0,09743 | -0,09686 | -0,07633 | 0,034872 | 0,325238 |
| ***Amh*** | -0,27951 | 0,015879 | 0,081219 | 0,133097 | 0,329991 |
| ***Agrp*** | -0,26221 | 0,454005 | 0,046157 | 0,033995 | 0,005452 |
| ***Galr3*** | -0,11189 | 0,427221 | -0,41417 | 0,142869 | 0,21663 |
| ***Aloxe3*** | 0,071539 | -0,43518 | 0,537271 | 0,017422 | 0,068522 |
| ***Fgl1*** | -0,60514 | -0,19903 | 0,468235 | 0,291501 | 0,295674 |
| ***Crtc3*** | -0,01524 | 0,346289 | -0,02403 | 0,105226 | -0,1638 |
| ***Prpf19*** | 0,247509 | 0,053185 | 0,072431 | -0,08793 | -0,0434 |
| ***Npy1r*** | 0,247693 | 0,041606 | 0,077397 | -0,00081 | -0,12691 |
| ***Kcnk3*** | -0,00286 | 0,262584 | 0,04446 | 0,03308 | -0,10329 |
| ***Rilp*** | 0,022063 | 0,325001 | -0,118 | -0,02773 | 0,030987 |
| ***Cldn16*** | -0,06315 | 0,304914 | -0,04512 | -0,07172 | 0,106233 |
| ***Hp*** | 0,146085 | 0,417231 | -0,32424 | -0,09493 | 0,086643 |
| ***A3galt2*** | -0,23166 | 0,394605 | 0,134691 | 0,009511 | -0,07663 |
| ***A4galt*** | -0,17343 | 0,436676 | -0,03531 | -0,27341 | 0,275621 |
| ***Grhl3*** | -0,08287 | 0,112833 | -0,07182 | -0,04773 | 0,311379 |
| ***Ormdl1*** | 0,065653 | 0,238703 | -0,0413 | -0,11032 | 0,066434 |
| ***Ghrl*** | -0,25736 | 0,238318 | 0,189848 | 0,054328 | -0,0071 |
| ***Cln3*** | -0,23828 | -0,25091 | 0,383911 | 0,179902 | 0,135794 |
| ***Lactb*** | 0,28749 | 0,074647 | 0,036979 | 0,000241 | -0,19206 |
| ***Ttr*** | -0,01991 | 0,374371 | 0,084331 | -0,10555 | -0,12829 |
| ***G6pc2*** | -0,25135 | 0,21277 | 0,01594 | 0,100865 | 0,123581 |
| ***Eci1*** | 0,191785 | 0,457583 | -0,13468 | -0,0355 | -0,27868 |
| ***Pi4ka*** | 0,227025 | -0,35982 | 0,24356 | 0,245809 | -0,15747 |
| ***Siah2*** | -0,01172 | 0,380758 | 0,025625 | -0,11201 | -0,08449 |
| ***Cry1*** | -0,28141 | 0,185524 | 0,210696 | 0,139586 | -0,05626 |
| ***Kcna6*** | -0,05215 | 0,34668 | 0,091492 | -0,07151 | -0,1166 |
| ***Ghr*** | 0,020345 | 0,862462 | -0,07016 | -0,35706 | -0,26179 |
| ***Map3k7*** | 0,331366 | -0,11687 | 0,027811 | 0,048484 | -0,09728 |
| ***Gata6*** | 0,246508 | 0,014495 | 0,073944 | -0,08173 | -0,06542 |
| ***Creb3l3*** | 0,384907 | 0,539266 | -0,04744 | -0,33531 | -0,35437 |
| ***Gsk3a*** | 0,079988 | -0,12934 | -0,0048 | 0,275579 | -0,03598 |
| ***Ldlr*** | -0,19533 | -0,23531 | 0,05352 | 0,181182 | 0,380566 |
| ***Hsd11b2*** | -0,26447 | 0,06894 | -0,03823 | 0,08626 | 0,330548 |
| ***Ell*** | 0,334325 | 0,054944 | 0,075763 | -0,08828 | -0,19445 |
| ***Fads2*** | -0,2342 | 0,280332 | 0,08521 | 0,085893 | -0,03826 |
| ***Sidt2*** | -0,1165 | -0,2445 | 0,252335 | 0,298435 | -0,01344 |
| ***Arnt*** | -0,02327 | 0,187907 | -0,00335 | -0,04279 | 0,055936 |
| ***Phgdh*** | -0,37389 | -0,2253 | -0,11459 | 0,328638 | 0,556468 |
| ***Rcn3*** | -0,31829 | 0,154371 | 0,141068 | -0,13166 | 0,321979 |
| ***Sox17*** | -0,37317 | 0,061962 | 0,098822 | 0,134528 | 0,242789 |
| ***Gfer*** | 0,277634 | -0,11273 | 0,059768 | 0,044205 | -0,10632 |
| ***Crhr2*** | -0,209 | 0,272133 | 0,154132 | -0,12835 | 0,073522 |
| ***Tnf*** | -0,18177 | -0,4173 | 0,296697 | 0,27893 | 0,183334 |
| ***Edil3*** | -0,0684 | -0,01399 | -0,09755 | -0,00929 | 0,348314 |
| ***Apoa2*** | -0,17361 | 0,299471 | 0,622124 | -0,05897 | -0,53274 |
| ***Synj1*** | 0,053365 | -0,21435 | 0,100531 | 0,094958 | 0,112213 |
| ***Smpd2*** | 0,373988 | 0,183463 | -0,043 | -0,20745 | -0,16175 |
| ***Cerk*** | 0,37678 | -0,40871 | 0,24643 | -0,13216 | 0,053874 |
| ***Sec16b*** | 0,145443 | -0,73409 | 0,141735 | 0,400965 | 0,173858 |
| ***Gba*** | -0,08949 | -0,30691 | 0,002287 | 0,281243 | 0,239962 |
| ***Nr2c2*** | 0,307347 | 0,275021 | -0,17379 | -0,19836 | -0,08375 |
| ***Angpt2*** | -0,02909 | -0,22349 | 0,483676 | 0,187039 | -0,29222 |
| ***Rxra*** | 0,193827 | 0,169862 | 0,015074 | 0,019906 | -0,27327 |
| ***Scarb1*** | -0,17737 | 0,467088 | 0,007424 | 0,012039 | -0,19073 |
| ***Inppl1*** | 0,322216 | -0,24926 | -0,13938 | 0,17604 | 0,003393 |
| ***Fuca1*** | 0,096608 | 0,185472 | -0,08699 | -0,02084 | -0,06202 |
| ***Nr3c1*** | -0,18234 | 0,22316 | 0,147401 | 0,048842 | -0,12813 |
| ***Akt3*** | -0,24716 | -0,00756 | 0,243473 | 0,466583 | -0,35147 |
| ***Ptpmt1*** | 0,332062 | -0,17379 | 0,146425 | 0,019344 | -0,22093 |
| ***Slc25a13*** | -0,13467 | -0,22342 | 0,44586 | 0,19267 | -0,18293 |
| ***Caln1*** | 0,244506 | 0,18034 | -0,10949 | -0,29417 | 0,071705 |
| ***Hsd17b12*** | 0,157407 | -0,54123 | 0,098661 | 0,176826 | 0,198638 |
| ***Pla2g6*** | 0,554899 | -0,63084 | 0,04968 | -0,0147 | 0,130395 |
| ***Ptpn11*** | 0,277848 | -0,11732 | -0,15029 | 0,091185 | -0,0196 |
| ***Pnpla2*** | 0,247679 | -0,29319 | 0,098474 | 0,152412 | -0,12929 |
| ***Ptges3*** | -0,09485 | -0,13093 | 0,394845 | 0,005238 | -0,10124 |
| ***Smpd1*** | 0,298504 | -0,25833 | -0,03771 | 0,003571 | 0,063222 |
| ***Slc4a5*** | -0,29224 | 0,055889 | -0,06684 | 0,188491 | 0,183856 |
| ***Loc102724788/prodh*** | -0,30097 | 0,39402 | -0,07611 | -0,03998 | 0,084271 |
| ***Htt*** | -0,11077 | -0,14556 | 0,015363 | 0,065637 | 0,222904 |
| ***Atg5*** | -0,02352 | 0,168563 | 0,017086 | -0,0226 | -0,09226 |
| ***Kcnma1*** | -0,29753 | 0,071285 | 0,058732 | 0,166292 | 0,045092 |
| ***Sirt3*** | 0,047248 | 0,309398 | 0,022808 | -0,15282 | -0,19995 |
| ***Ahr*** | -0,14658 | 0,313906 | -0,04025 | -0,03685 | -0,07687 |
| ***Ptpn1*** | 0,437937 | -0,19246 | -0,10771 | -0,12734 | 1,21E-05 |
| ***Avpr1b*** | -0,4407 | 0,167051 | 0,145986 | 0,094031 | 0,039529 |
| ***Gprc6a*** | 0,372585 | -0,01791 | -0,22916 | -0,04782 | -0,08179 |
| ***Slc23a2*** | 0,013048 | 0,437582 | -0,00679 | -0,11345 | -0,3352 |
| ***Cartpt*** | -0,24809 | 0,105997 | 0,057639 | -0,16222 | 0,236838 |
| ***Smad4*** | -0,10835 | 0,213073 | -0,02205 | -0,07522 | -0,02434 |
| ***Ctdnep1*** | 0,193788 | -0,33646 | -0,14355 | 0,264501 | 0,001115 |
| ***Ncoa5*** | 0,369041 | -0,18318 | -0,06476 | -0,21848 | 0,073363 |
| ***Abhd12*** | 0,213606 | -0,40306 | -0,09802 | 0,1232 | 0,136127 |
| ***Adora1*** | -0,02515 | 0,460472 | 0,296181 | -0,19368 | -0,5675 |
| ***Gnat1*** | -0,35967 | -0,17673 | 0,272882 | 0,155199 | 0,074056 |
| ***Slc26a3*** | -0,21409 | 0,338524 | 0,066967 | -0,09916 | -0,12936 |
| ***Bmp4*** | 0,171514 | 0,081263 | -0,73047 | -0,11983 | 0,554589 |
| ***Epas1*** | 0,107156 | 0,425131 | -0,27261 | -0,08373 | -0,22467 |
| ***Smad3*** | 0,136784 | 0,106913 | -0,0102 | -0,24429 | -0,04875 |
| ***Rsc1a1*** | 0,032899 | 0,013093 | 0,040263 | 0,039339 | -0,19302 |
| ***G6pd*** | -0,91356 | -0,81963 | 0,563423 | 0,760106 | 0,3419 |
| ***Scg5*** | -0,41317 | 0,053442 | -0,02387 | 0,168028 | 0,146947 |
| ***Sirt4*** | -0,21548 | 0,198685 | 0,044321 | -0,10078 | 0,000804 |
| ***Bscl2*** | 0,024949 | 0,242515 | -0,16808 | -0,0596 | -0,11227 |
| ***Extl1*** | -0,37672 | 0,136722 | 0,000479 | 0,042108 | 0,121939 |
| ***Gba2*** | 0,300028 | -0,10983 | -0,25287 | 0,121265 | -0,13927 |
| ***Camp*** | -0,3413 | 0,170226 | 0,074399 | 0,066094 | -0,05043 |
| ***Prkci*** | 0,024551 | -0,43889 | 0,186525 | 0,082533 | 0,04227 |
| ***Slc6a11*** | -0,24472 | -0,0248 | -0,00414 | -0,11504 | 0,282429 |
| ***Bcr*** | 0,131687 | 0,049307 | -0,2822 | 0,041924 | -0,05062 |
| ***Nampt*** | -0,40323 | 0,39578 | 0,212441 | -0,00136 | -0,32939 |
| ***Srebf1*** | -0,75619 | 0,685746 | 0,064183 | -0,03825 | -0,08533 |
| ***Eef1a2*** | -0,3274 | 0,021317 | 0,134282 | -0,10705 | 0,147264 |
| ***Pemt*** | -0,23697 | 0,989664 | 0,319489 | -0,40521 | -0,8003 |
| ***Azgp1*** | -0,00885 | 0,382591 | -0,28781 | -0,18657 | -0,03767 |
| ***Angptl3*** | -0,01735 | 0,479563 | 0,012199 | 0,059137 | -0,67252 |
| ***Bax*** | 0,015485 | -0,26442 | 0,05446 | -0,0615 | 0,110614 |
| ***Pttg1*** | 0,002443 | 0,360908 | -0,06424 | -0,20441 | -0,24062 |
| ***Pld1*** | -0,00404 | -0,54508 | -0,09043 | 0,187057 | 0,297768 |
| ***Epb41*** | 0,204174 | -0,46832 | -0,08681 | 0,144948 | 0,049357 |
| ***Cyp27b1*** | -0,68583 | 0,242121 | 0,083633 | 0,047049 | 0,156222 |
| ***C3*** | -0,17852 | 8,03E-05 | -0,12049 | 0,058399 | 0,081334 |
| ***Fkbp4*** | 0,042169 | -0,3762 | 0,045305 | 0,037686 | 0,091123 |
| ***Vac14*** | 0,21733 | -0,5822 | 0,025768 | 0,127949 | 0,047837 |
| ***Pcmt1*** | 0,129249 | -0,29306 | 0,2376 | -0,10905 | -0,12959 |
| ***Rab7a*** | 0,052434 | -0,25016 | -0,07997 | 0,077602 | 0,029921 |
| ***Macroh2a1*** | -0,01885 | -0,18706 | 0,090669 | -0,05809 | 0,002547 |
| ***Cspg4*** | -0,38176 | 0,127018 | 0,219829 | -0,19893 | 0,058491 |
| ***Mc2r*** | 0,00145 | -0,24047 | -0,25341 | 0,201722 | 0,111836 |
| ***Rock2*** | 0,295396 | -0,29271 | -0,18228 | -0,03032 | 0,026191 |
| ***Cfb*** | -0,10803 | 0,309383 | -0,21804 | -0,10759 | -0,05946 |
| ***Ces1*** | -0,12385 | 0,340526 | 0,219336 | 0,011072 | -0,6343 |
| ***Apoa1*** | 0,200219 | -0,07687 | -0,25979 | -0,08649 | 0,018263 |
| ***Psen2*** | -0,17601 | 0,531063 | -0,16283 | -0,09156 | -0,30579 |
| ***Sigmar1*** | -0,19445 | -0,37438 | 0,068116 | 0,088142 | 0,202521 |
| ***Clock*** | 0,191288 | -0,34065 | -0,10692 | 0,030621 | 0,010816 |
| ***Pcsk9*** | -0,52601 | -0,42503 | 0,039007 | 0,336342 | 0,359866 |
| ***Pon2*** | 0,295087 | -0,47237 | -0,19 | 0,01826 | 0,132092 |
| ***Hnf1a*** | 0,141772 | -0,46934 | 0,133149 | 0,073791 | -0,10086 |
| ***Comt*** | 0,338135 | -0,06313 | -0,25532 | -0,2088 | -0,03251 |
| ***Inha*** | -0,42801 | -0,00526 | 0,147948 | -0,0045 | 0,066486 |
| ***Atp8b1*** | -0,01227 | -0,40007 | -0,09746 | 0,211675 | 0,070155 |
| ***Rac1*** | 0,004156 | -0,36322 | 0,012891 | 0,016666 | 0,099646 |
| ***Uaca*** | 0,188491 | -0,33582 | -0,14017 | 0,034623 | 0,019827 |
| ***Prl3b1*** | -0,04059 | -0,04654 | 0,103684 | -0,27156 | 0,020439 |
| ***Slc6a4*** | -0,32581 | -0,10536 | -0,01426 | 0,104938 | 0,105315 |
| ***Naglu*** | -0,20126 | -0,45917 | 0,003876 | 0,165193 | 0,256003 |
| ***Hpgds*** | -0,43071 | 0,138671 | 0,02627 | 0,073397 | -0,04638 |
| ***Bpnt1*** | 0,040749 | -0,29033 | 0,135123 | -0,08834 | -0,03691 |
| ***Rara*** | 0,118619 | -0,32306 | -0,15279 | 0,129174 | -0,01458 |
| ***Chst10*** | -0,37018 | -0,01537 | -0,1227 | 0,059074 | 0,20577 |
| ***Sik3*** | 0,120441 | -0,29053 | -0,03961 | 0,040252 | -0,08242 |
| ***Aqp8*** | -0,71873 | 0,170539 | 0,222727 | 0,155749 | -0,08264 |
| ***Lgr5*** | -0,33982 | 0,136406 | -0,01954 | -0,11372 | 0,077548 |
| ***Lypla2*** | -0,12933 | -0,34098 | 0,05954 | 0,081493 | 0,057094 |
| ***Dbi*** | 0,109036 | 0,036403 | -0,24376 | -0,13536 | -0,03999 |
| ***Atp1a1*** | -0,15936 | -0,41783 | 0,023192 | 0,219062 | 0,054036 |
| ***Pex2*** | 0,068347 | -0,15174 | 0,080045 | -0,24601 | -0,03156 |
| ***Rhoa*** | -0,13676 | -0,1495 | 0,013621 | -0,03237 | 0,023586 |
| ***Cysltr2*** | -0,32996 | -0,04093 | -0,02947 | 0,107027 | -0,00464 |
| ***Atg7*** | 0,193859 | 0,230731 | -0,31903 | -0,38279 | -0,02735 |
| ***Sptlc2*** | -0,31244 | -0,16422 | -0,25597 | 0,165366 | 0,255729 |
| ***Pik3r1*** | 0,090286 | -0,57779 | -0,2358 | 0,144529 | 0,263983 |
| ***Scd2*** | -0,43642 | -0,0554 | -0,01182 | -0,0192 | 0,20793 |
| ***Fgf7*** | -0,08165 | 0,136288 | -0,19965 | 0,163945 | -0,33884 |
| ***Ppara*** | 0,284157 | 0,016818 | -0,01524 | -0,10652 | -0,51136 |
| ***Eef1a1*** | -0,04348 | -0,14783 | -0,05712 | -0,0585 | -0,03357 |
| ***Gna11*** | 0,117817 | -0,38846 | -0,07016 | 0,053208 | -0,05876 |
| ***Ubr1*** | -0,10765 | -0,23963 | 0,017054 | -0,05908 | 0,041492 |
| ***Sftpd*** | -0,16552 | 0,098252 | -0,002 | -0,35054 | 0,067491 |
| ***P2rx7*** | -0,39759 | -0,02834 | -0,0222 | 0,050312 | 0,038123 |
| ***Prkce*** | 0,064813 | -0,4592 | -0,03295 | 0,190098 | -0,12429 |
| ***Hmgn1*** | 0,015842 | -0,41325 | 0,074513 | -0,07857 | 0,036205 |
| ***Arf1*** | -0,02908 | -0,17576 | -0,04708 | -0,06413 | -0,05279 |
| ***Apoe*** | 0,00429 | 0,122289 | -0,03966 | -0,30261 | -0,15475 |
| ***Cacna1b*** | -0,66315 | 0,077493 | 0,049701 | -0,02331 | 0,185651 |
| ***Hadha*** | 0,048218 | -0,04795 | -0,04955 | -0,1482 | -0,18075 |
| ***Naa40*** | 0,166896 | -0,33088 | -0,10867 | -0,00717 | -0,10186 |
| ***Pla2g15*** | -0,18275 | -0,48308 | 0,281231 | -0,05829 | 0,056042 |
| ***Scnn1b*** | -0,09665 | -0,04399 | -0,25684 | 0,057373 | -0,04988 |
| ***B4galnt1*** | -0,22002 | -0,27144 | -0,01329 | 0,109422 | -0,00439 |
| ***Mbtps1*** | 0,062942 | 0,065451 | -0,41234 | -0,03739 | -0,08008 |
| ***Inpp5k*** | -0,04817 | -0,26143 | 0,047599 | 0,083098 | -0,2323 |
| ***Ptk2*** | 0,271355 | -0,35804 | -0,05002 | -0,10527 | -0,1699 |
| ***Srsf2*** | -0,27629 | -0,46884 | 0,102153 | 0,118632 | 0,111697 |
| ***Serpina6*** | -0,73851 | 0,531935 | 0,070508 | -0,01966 | -0,2586 |
| ***Elovl5*** | -0,2241 | 0,445751 | -0,17497 | -0,27147 | -0,19038 |
| ***Pnpla8*** | -0,02232 | -0,0185 | 0,08681 | -0,16123 | -0,30148 |
| ***Mtmr14*** | -0,08559 | -0,05768 | -0,25696 | 0,03931 | -0,05986 |
| ***Lmna*** | -0,11056 | -0,49526 | -0,03898 | 0,036944 | 0,186785 |
| ***Dgkq*** | -0,08991 | -0,25209 | -0,00504 | 0,121109 | -0,19845 |
| ***Pde3b*** | -0,06062 | 0,05197 | -0,11567 | -0,03503 | -0,27351 |
| ***Adh1c*** | -0,16409 | 0,881367 | -0,32695 | -0,40502 | -0,41997 |
| ***Nkx2-1*** | -0,17326 | -0,06626 | 0,095315 | 0,098483 | -0,39113 |
| ***Sgms2*** | -0,12935 | -0,39279 | -0,04223 | 0,094152 | 0,030405 |
| ***Ezh2*** | 0,123533 | -0,09263 | -0,18177 | -0,21917 | -0,07101 |
| ***Slc27a1*** | -0,41268 | 0,148235 | 0,088034 | -0,06771 | -0,20017 |
| ***Rdh12*** | -0,20221 | -0,40604 | 0,113847 | 0,009118 | 0,040574 |
| ***Nr0b2*** | -0,35622 | 0,233567 | 0,047325 | -0,39205 | 0,021575 |
| ***Naaa*** | -0,04221 | -0,2246 | -0,09696 | 0,172792 | -0,2631 |
| ***Stat6*** | 0,273005 | -0,43594 | -0,2113 | -0,07114 | -0,00908 |
| ***Ptpn6*** | -0,15087 | -0,10891 | -0,30655 | -0,02284 | 0,128052 |
| ***Pdcd6ip*** | 0,011965 | -0,37531 | -0,12664 | 0,02417 | 0,002978 |
| ***Sphk2*** | 0,053698 | -0,31016 | -0,07302 | -0,01502 | -0,13099 |
| ***Slc9a3*** | -0,31363 | 0,140505 | -0,08035 | -0,14339 | -0,08026 |
| ***Psap*** | -0,19939 | 0,011129 | -0,06714 | -0,18966 | -0,03282 |
| ***Col18a1*** | -0,04681 | 0,335269 | -0,47504 | -0,12839 | -0,16507 |
| ***Pitpna*** | -0,04177 | -0,55117 | 0,005303 | 0,079987 | 0,023119 |
| ***Nos3*** | -0,44887 | 0,068903 | -0,05055 | -0,06996 | 0,013571 |
| ***Hspa5*** | 0,034901 | -0,31971 | -0,09675 | -0,05292 | -0,06005 |
| ***Vtn*** | -0,07626 | 0,194188 | 0,082433 | -0,44226 | -0,25958 |
| ***Stk39*** | -0,17954 | 0,305643 | 0,080196 | -0,3769 | -0,33819 |
| ***Slc34a2*** | -0,88511 | 0,071501 | 0,013447 | 0,198904 | 0,088975 |
| ***Insr*** | -0,04304 | -0,30847 | -0,01885 | -0,02239 | -0,12176 |
| ***Ctsd*** | -0,22888 | -0,15331 | -0,0349 | -0,05525 | -0,04359 |
| ***Sptlc1*** | -0,01337 | -0,45987 | 0,132835 | -0,10487 | -0,07282 |
| ***Sorbs1*** | 0,139075 | -0,07047 | 0,079866 | -0,26281 | -0,40649 |
| ***Ubiad1*** | 0,062486 | -0,13714 | -0,17219 | -0,07707 | -0,19697 |
| ***Onecut1*** | 0,673633 | -0,44932 | -0,54448 | 0,010427 | -0,21462 |
| ***Helz2*** | 0,089805 | -0,19073 | -0,25008 | -0,09299 | -0,08429 |
| ***Gstk1*** | -0,18235 | 0,980101 | -0,47865 | -0,43221 | -0,41973 |
| ***Ptdss1*** | -0,24964 | -0,04251 | -0,22116 | -0,03904 | 0,019207 |
| ***Tardbp*** | -0,14653 | -0,25438 | -0,00405 | -0,06842 | -0,07168 |
| ***Hdac5*** | 0,116794 | -0,23882 | -0,13526 | -0,13365 | -0,16138 |
| ***Slco2a1*** | 0,046009 | -1,01397 | -0,20624 | 0,30514 | 0,313243 |
| ***Efna5*** | -0,10752 | 0,368022 | -0,12622 | -0,34529 | -0,35821 |
| ***Prkaa2*** | 0,026958 | -0,16209 | -0,16135 | -0,10442 | -0,16972 |
| ***Hmgcr*** | -1,05361 | 0,128838 | 0,194174 | -0,00519 | 0,16514 |
| ***Saa2-saa4*** | 0,244261 | 0,828813 | -0,8911 | -0,49218 | -0,26217 |
| ***Pitpnb*** | -0,08261 | -0,15436 | -0,09378 | -0,15169 | -0,0915 |
| ***Lyst*** | 0,002538 | -0,32392 | 0,058084 | -0,08212 | -0,22961 |
| ***Igf2bp2*** | -0,19494 | 0,242714 | -0,23411 | -0,36201 | -0,02736 |
| ***Pten*** | -0,06088 | -0,2313 | -0,00484 | -0,14557 | -0,13593 |
| ***Il4r*** | 0,007806 | 0,036066 | -0,16856 | -0,19677 | -0,25987 |
| ***Hif1a*** | -0,06375 | -0,27724 | -0,25817 | 0,067145 | -0,05719 |
| ***Xpa*** | -0,01753 | 0,002382 | -0,12123 | -0,31444 | -0,14139 |
| ***Lgals8*** | 0,098617 | -0,19609 | -0,12526 | -0,24418 | -0,12703 |
| ***Gucy2c*** | -0,44326 | 0,015786 | 0,131384 | -0,36043 | 0,050101 |
| ***Osbp*** | 0,216893 | -0,62377 | -0,11025 | -0,01803 | -0,07237 |
| ***Akt1*** | -0,12742 | -0,47145 | -0,12531 | -0,0356 | 0,152021 |
| ***Mtm1*** | -0,12409 | -0,12677 | 0,137794 | -0,1124 | -0,38462 |
| ***Sc5d*** | -0,31835 | 0,126571 | -0,17349 | -0,20497 | -0,04536 |
| ***Hnf4a*** | 0,050151 | -0,0984 | -0,05882 | -0,18117 | -0,33054 |
| ***Gne*** | 0,132959 | -0,22054 | 0,021975 | -0,12753 | -0,42869 |
| ***Arhgdia*** | -0,11931 | -0,56751 | -0,11756 | 0,138041 | 0,04289 |
| ***Icam1*** | 0,028746 | -0,47266 | -0,35183 | -0,13022 | 0,302092 |
| ***Jdp2*** | -0,06718 | 0,0799 | -0,36563 | -0,3744 | 0,097755 |
| ***Cers2*** | -0,2211 | -0,23697 | -0,0219 | -0,07556 | -0,08701 |
| ***Hsd11b1*** | -0,18894 | 0,270435 | -0,05613 | -0,22592 | -0,44326 |
| ***Fadd*** | 0,106753 | -0,48414 | -0,24516 | -0,03754 | 0,013868 |
| ***Hrh1*** | -0,34244 | -0,13853 | 0,104488 | -0,22612 | -0,04703 |
| ***Lpgat1*** | -0,05591 | -0,44222 | -0,20386 | -0,01972 | 0,067357 |
| ***Plcl2*** | -0,14263 | 0,227116 | -0,1606 | -0,32853 | -0,25868 |
| ***Tyk2*** | 0,010038 | -0,2758 | -0,26825 | -0,17562 | 0,040721 |
| ***Adra1b*** | -0,21175 | 0,299552 | -0,33611 | -0,34551 | -0,08002 |
| ***Tnfrsf1a*** | -0,28457 | -0,31858 | 0,02452 | 0,128835 | -0,232 |
| ***Inpp5e*** | -0,16599 | -0,24439 | -0,10893 | -0,06506 | -0,09855 |
| ***Acly*** | -0,53178 | -0,15754 | -0,04428 | 0,10239 | -0,05604 |
| ***Dgat2*** | -0,08642 | 0,211404 | -0,25964 | -0,24013 | -0,31512 |
| ***Ocrl*** | -0,16671 | -0,35329 | -0,09241 | -0,11351 | 0,03534 |
| ***Acsl4*** | 0,022254 | -0,66087 | -0,26081 | -0,00454 | 0,213346 |
| ***Lbp*** | -0,33897 | -0,19207 | -0,26068 | -0,01096 | 0,109726 |
| ***Aqp9*** | -0,00116 | 0,275407 | -0,33264 | -0,34037 | -0,29478 |
| ***Afap1*** | 0,028336 | 0,245332 | -0,05185 | -0,42686 | -0,49282 |
| ***Slc30a7*** | -0,0057 | -0,51473 | -0,06318 | -0,02453 | -0,08975 |
| ***Cd59*** | -0,0193 | 0,05104 | -0,03612 | -0,56651 | -0,1274 |
| ***Stard3*** | -0,19645 | -0,28329 | -0,0961 | -0,05702 | -0,06698 |
| ***Pank1*** | -0,19551 | 0,55274 | -0,18486 | -0,32048 | -0,55342 |
| ***Sirt5*** | 0,019741 | -0,08465 | -0,23853 | -0,18128 | -0,21806 |
| ***Bbs12*** | -0,37029 | -0,05847 | -0,14843 | -0,1092 | -0,02322 |
| ***Rora*** | 0,248717 | 0,183908 | -0,43177 | -0,19966 | -0,52553 |
| ***Myd88*** | 0,190685 | -0,79805 | -0,19427 | 0,007765 | 0,069196 |
| ***Elmo1*** | 0,201524 | -0,76485 | 0,116796 | -0,22041 | -0,05777 |
| ***Rab27a*** | -0,19029 | 0,240772 | -0,39714 | -0,21802 | -0,16054 |
| ***Gpr12*** | -0,30764 | -0,10047 | -0,2402 | 0,094568 | -0,17826 |
| ***Gpat3*** | -0,40771 | -0,14956 | -0,19328 | -0,03526 | 0,053116 |
| ***Cyp3a7*** | -0,36401 | -0,43583 | -0,05304 | 0,112209 | 0,001546 |
| ***Jak2*** | -0,02781 | -0,30249 | -0,25278 | 0,041496 | -0,19887 |
| ***Slc37a4*** | -0,25393 | 0,120803 | 0,431362 | -0,25127 | -0,78958 |
| ***Amacr*** | 0,266114 | 0,465913 | -0,78026 | -0,35673 | -0,34628 |
| ***Plg*** | -0,03442 | 0,501651 | -0,11731 | -0,58013 | -0,52523 |
| ***Il18bp*** | -0,67711 | -0,15449 | -0,15196 | 0,01522 | 0,207936 |
| ***Scd*** | -0,13432 | 0,289914 | 0,097704 | -0,38032 | -0,63388 |
| ***Kif13b*** | -0,10431 | -0,57096 | -0,3311 | 0,000255 | 0,237186 |
| ***Pdzk1*** | -0,29082 | 0,097614 | -0,14233 | -0,14967 | -0,28653 |
| ***Sec14l2*** | 0,01917 | 0,028963 | -0,1995 | -0,16686 | -0,45464 |
| ***Mapk3*** | -0,12178 | -0,2083 | -0,11816 | -0,24199 | -0,08334 |
| ***Pank2*** | -0,15873 | -0,06449 | -0,2655 | -0,14184 | -0,14488 |
| ***Crat*** | -0,13528 | -0,3682 | -0,04971 | -0,08027 | -0,14379 |
| ***Arv1*** | -0,01046 | -0,2308 | -0,34189 | -0,03724 | -0,161 |
| ***Klb*** | -0,33321 | 0,761566 | -0,40744 | -0,33194 | -0,47152 |
| ***Elovl1*** | -0,08439 | -0,74338 | -0,09103 | 0,023073 | 0,096609 |
| ***F7*** | -0,28298 | 0,447662 | -0,18694 | -0,3037 | -0,47445 |
| ***Pck1*** | 0,022757 | 2,085707 | -0,98927 | -1,0657 | -0,8563 |
| ***Gal3st1*** | 0,031791 | 0,169543 | -0,41082 | -0,27603 | -0,33125 |
| ***Acot13*** | -0,20654 | -0,48348 | -0,03027 | -0,11397 | 0,00707 |
| ***Inpp5b*** | -0,07103 | -0,17058 | -0,26844 | -0,1809 | -0,13715 |
| ***Cga*** | -0,51409 | -0,06749 | -0,12433 | -0,08328 | -0,04048 |
| ***Foxa2*** | 0,050876 | -0,561 | -0,04803 | -0,12144 | -0,16052 |
| ***Decr1*** | 0,10697 | 0,084374 | -0,32973 | -0,45541 | -0,24776 |
| ***Ccl2*** | 0,066708 | -1,28911 | -0,39182 | 0,091752 | 0,678362 |
| ***Npc2*** | -0,37777 | -0,36177 | -0,13016 | -0,07041 | 0,090643 |
| ***Flot2*** | -0,30772 | -0,19986 | -0,28756 | -0,10725 | 0,048118 |
| ***Ppp1r3c*** | -0,20615 | 0,757792 | 0,253787 | -0,85739 | -0,80804 |
| ***Fig4*** | -0,02555 | -0,5778 | -0,14152 | -0,08147 | -0,03368 |
| ***Neu3*** | -0,24807 | -0,10517 | -0,3995 | -0,16853 | 0,059742 |
| ***Tnik*** | 0,038399 | -0,10285 | -0,19746 | -0,1282 | -0,47223 |
| ***Klf15*** | 0,061813 | 0,288931 | -0,33865 | -0,36139 | -0,5246 |
| ***Dhcr7*** | -0,22865 | -0,15788 | -0,20414 | -0,13482 | -0,15003 |
| ***Cyp26a1*** | -0,88258 | 0,748972 | -0,40208 | -0,30796 | -0,03217 |
| ***Nuak2*** | 0,406218 | -0,31669 | -0,24485 | -0,30523 | -0,41702 |
| ***Pctp*** | -0,4482 | -0,24054 | 0,059516 | -0,06193 | -0,19305 |
| ***Dlg4*** | -0,50511 | -0,05985 | -0,35011 | 0,009915 | 0,019942 |
| ***Faah*** | 0,061569 | 0,020827 | -0,45332 | -0,27998 | -0,24073 |
| ***Rab9a*** | -0,08222 | -0,17571 | -0,17938 | -0,23311 | -0,22621 |
| ***Cnep1r1*** | -0,21241 | -0,17938 | -0,14926 | -0,2981 | -0,07127 |
| ***Lsr*** | -0,17324 | -0,54944 | -0,24564 | 0,061841 | -0,00853 |
| ***Nr1h3*** | 0,045567 | -0,18155 | -0,28072 | -0,24717 | -0,25165 |
| ***Kdm5c*** | 0,076582 | -0,01924 | -0,17948 | -0,36561 | -0,43177 |
| ***Aifm1*** | -0,08824 | -0,35303 | -0,21819 | -0,20021 | -0,06116 |
| ***Plin2*** | 0,099249 | -0,88332 | -0,13492 | -0,13418 | 0,130005 |
| ***Acadl*** | -0,20704 | -0,55124 | -0,02583 | -0,07497 | -0,06891 |
| ***Bco1*** | -0,40829 | -1,00127 | 0,010991 | 0,36092 | 0,102706 |
| ***Entpd5*** | 0,156464 | -0,39686 | -0,02675 | -0,25253 | -0,41727 |
| ***Pdk4*** | -0,7756 | 0,003353 | 0,254408 | -0,1653 | -0,2616 |
| ***Vcam1*** | 0,14272 | -0,08553 | -0,26698 | -0,16611 | -0,57392 |
| ***Ifngr1*** | -0,13345 | -0,30465 | -0,26463 | -0,21911 | -0,02838 |
| ***Nat2*** | -0,40048 | -0,12426 | -0,43352 | -0,03088 | 0,032331 |
| ***Pip5k1c*** | -0,21435 | -0,45938 | -0,21608 | -0,03237 | -0,03858 |
| ***Adipor2*** | -0,11821 | 0,070816 | -0,11016 | -0,35886 | -0,44666 |
| ***Ceacam1*** | -0,1119 | 0,280183 | -0,65997 | -0,21636 | -0,25564 |
| ***Mttp*** | -0,05097 | -0,37223 | -0,1664 | -0,00399 | -0,37249 |
| ***Cds2*** | -0,39322 | -0,22957 | -0,07803 | -0,19952 | -0,07235 |
| ***Ppp3ca*** | -0,04094 | -0,42466 | -0,12271 | -0,17327 | -0,21151 |
| ***Dnajc7*** | -0,04284 | -0,5412 | 0,021931 | -0,29237 | -0,12199 |
| ***Lpcat3*** | -0,09517 | -0,3828 | -0,1587 | -0,18809 | -0,15224 |
| ***Cr1l*** | -0,15732 | -0,48739 | -0,20058 | -0,02679 | -0,11211 |
| ***Hadh*** | -0,2833 | 0,412573 | -0,43077 | -0,41483 | -0,27304 |
| ***Mboat7*** | -0,18279 | -0,3159 | -0,03117 | -0,31255 | -0,15441 |
| ***Cfh*** | -0,04942 | 0,49017 | -0,19626 | -0,71788 | -0,52788 |
| ***Taz*** | 0,05904 | -0,26099 | -0,11091 | -0,34407 | -0,34664 |
| ***Mtor*** | 0,012855 | -0,15939 | -0,11537 | -0,44566 | -0,29787 |
| ***Idh1*** | -0,07333 | -0,07805 | -0,38205 | -0,35781 | -0,13272 |
| ***Cideb*** | -0,0803 | -0,06647 | -0,34073 | -0,26516 | -0,27135 |
| ***Oma1*** | 0,026562 | -0,72651 | 0,001576 | -0,11092 | -0,22281 |
| ***Cdk4*** | -0,10627 | -0,41942 | -0,26368 | -0,14062 | -0,1066 |
| ***Lcn2*** | -2,07597 | -0,14677 | 0,223995 | 0,571115 | 0,390084 |
| ***Hsd17b4*** | 0,162203 | -0,53786 | -0,16633 | -0,25361 | -0,24445 |
| ***Lrp1*** | 0,060206 | -0,55842 | -0,2624 | -0,11997 | -0,17952 |
| ***Adrb2*** | -0,01087 | -0,54006 | -0,22739 | -0,07428 | -0,2077 |
| ***Abcg5*** | -0,60954 | 0,480023 | 0,03509 | -0,40794 | -0,56029 |
| ***Nedd4l*** | -0,10732 | -0,15282 | -0,31087 | -0,30478 | -0,18824 |
| ***Oxsm*** | -0,11358 | -0,3831 | -0,20943 | -0,24405 | -0,13282 |
| ***Ttpa*** | -0,31002 | -0,3907 | -0,22847 | -0,03957 | -0,11531 |
| ***Il33*** | -0,29263 | 0,166507 | -0,72088 | -0,42045 | 0,177044 |
| ***Erbb2*** | -0,10815 | -0,01312 | -0,46978 | -0,24009 | -0,26202 |
| ***Glb1*** | -0,22182 | -0,46823 | -0,1157 | -0,21303 | -0,07813 |
| ***Bco2*** | -0,29506 | 0,14775 | -0,18341 | -0,42434 | -0,347 |
| ***Anxa5*** | -0,01861 | -0,66775 | -0,21834 | -0,14331 | -0,06661 |
| ***Fdft1*** | -0,46574 | -0,23617 | -0,21606 | -0,15921 | -0,03858 |
| ***Kras*** | -0,26815 | -0,15401 | -0,19245 | -0,26561 | -0,23842 |
| ***Abcb11*** | -0,28694 | 0,895383 | -0,1812 | -0,56721 | -0,9809 |
| ***Fgfr2*** | -0,35329 | 0,060919 | -0,3489 | -0,43475 | -0,05061 |
| ***Fabp5*** | -0,71247 | 0,616043 | -0,73515 | -0,22654 | -0,06958 |
| ***Acp3*** | -0,73856 | 0,314863 | -0,29109 | -0,26268 | -0,15119 |
| ***Flot1*** | -0,20217 | -0,57138 | -0,18126 | -0,23436 | 0,057347 |
| ***Abcc3*** | -0,1613 | -0,49806 | -0,1044 | -0,23767 | -0,1381 |
| ***Asah2*** | -0,41224 | 0,042507 | -0,32461 | -0,27624 | -0,16936 |
| ***Parpbp*** | 0,223021 | -0,06672 | -1,01109 | -0,33915 | 0,052163 |
| ***Slco1b3*** | 0,060062 | 1,853445 | -0,18141 | -1,3031 | -1,57247 |
| ***Fgf1*** | -0,08053 | 0,240161 | -0,10593 | -0,55711 | -0,6425 |
| ***Abcc2*** | 0,030509 | -0,50224 | -0,16845 | -0,25012 | -0,25719 |
| ***Srebf2*** | -0,43648 | -0,59157 | -0,18447 | -0,00592 | 0,067099 |
| ***Prkab1*** | -0,21304 | 0,004241 | -0,24166 | -0,39474 | -0,30941 |
| ***Gas7*** | 0,034909 | -1,02158 | -0,55221 | 0,162408 | 0,208575 |
| ***Thrb*** | 0,064914 | -0,58191 | -0,25835 | -0,15244 | -0,24924 |
| ***Aldh1a1*** | 0,149176 | -0,68979 | -0,46523 | -0,18232 | 0,010048 |
| ***Pfkfb1*** | 0,426791 | -0,72513 | -0,32207 | -0,16839 | -0,39845 |
| ***Plat*** | -0,1987 | -0,33539 | -0,50693 | -0,24632 | 0,08422 |
| ***Btn1a1*** | -0,18193 | -0,23296 | -0,23607 | -0,36407 | -0,19135 |
| ***Ogt*** | -0,34307 | -0,25212 | -0,20671 | -0,23484 | -0,18719 |
| ***Anxa6*** | -0,23768 | -0,58634 | -0,27128 | -0,15766 | 0,020174 |
| ***Crp*** | -0,19494 | -0,08311 | -0,40456 | -0,21334 | -0,34008 |
| ***Abcd1*** | -0,49221 | 0,088577 | -0,0985 | -0,43144 | -0,32268 |
| ***Tlr2*** | -0,22506 | -0,32334 | -0,54692 | -0,18222 | 0,019467 |
| ***Wrn*** | -0,29844 | -0,06752 | -0,44964 | -0,30861 | -0,16156 |
| ***Pik3cb*** | -0,21367 | -0,8372 | -0,21036 | -0,02392 | -0,02048 |
| ***Elovl2*** | -0,1327 | 0,596818 | -0,41057 | -0,5967 | -0,77845 |
| ***Cert1*** | -0,42481 | -0,43365 | -0,14275 | -0,21424 | -0,1175 |
| ***Pld2*** | -0,14237 | -0,53318 | -0,49428 | -0,16364 | -0,00071 |
| ***Npc1*** | -0,24579 | 0,014381 | -0,29323 | -0,412 | -0,40126 |
| ***Cpt1a*** | 0,141407 | -0,3976 | -0,44114 | -0,41383 | -0,24206 |
| ***Xdh*** | -0,18054 | -0,28762 | -0,22192 | -0,2607 | -0,40273 |
| ***Inhba*** | -0,37941 | -0,2869 | -0,73343 | -0,23338 | 0,275028 |
| ***Plpp2*** | -0,44512 | -0,80011 | -0,26111 | -0,03867 | 0,181912 |
| ***Slc16a2*** | -0,04951 | 0,056159 | -0,64401 | -0,22569 | -0,50829 |
| ***Pml*** | -0,15464 | -0,33835 | -0,49261 | -0,32207 | -0,08209 |
| ***Ehhadh*** | -0,21017 | -0,18064 | -0,0393 | -0,47618 | -0,4945 |
| ***Dhrs3*** | -0,16063 | 0,452663 | -0,29804 | -0,74504 | -0,67502 |
| ***Il1rn*** | -0,75085 | 0,012076 | -0,49055 | 0,042444 | -0,23981 |
| ***Pon3*** | -0,08 | -0,52909 | -0,44654 | -0,28606 | -0,10196 |
| ***Sult1e1*** | -0,77274 | 0,088906 | 0,193589 | -0,32624 | -0,62783 |
| ***Agpat2*** | -0,03707 | -0,32925 | -0,47236 | -0,26184 | -0,35562 |
| ***Acat1*** | 0,022855 | 0,265225 | -0,44318 | -0,69614 | -0,61754 |
| ***Agt*** | -0,32361 | 0,085077 | -0,52515 | -0,41596 | -0,29566 |
| ***Prdx3*** | -0,23881 | -0,69977 | -0,2737 | -0,20182 | -0,07735 |
| ***Foxa1*** | 0,558808 | -0,8413 | -0,40635 | -0,48428 | -0,32452 |
| ***Tcf7l2*** | -0,31506 | -0,7708 | -0,1655 | -0,15239 | -0,09592 |
| ***Soat2*** | -0,2558 | -0,20424 | -0,22728 | -0,25872 | -0,55695 |
| ***Apoa4*** | -0,46224 | -0,92768 | -0,32289 | -0,02632 | 0,2323 |
| ***Il15ra*** | 0,1906 | -0,62726 | -0,39665 | -0,40524 | -0,27123 |
| ***Il18*** | -0,15491 | 0,211891 | -0,75962 | -0,60076 | -0,21168 |
| ***Sgms1*** | -0,26215 | -0,65755 | -0,17451 | -0,26171 | -0,16705 |
| ***Atp7b*** | -0,33 | -0,37341 | -0,18064 | -0,23327 | -0,41056 |
| ***Cyp2j5*** | -0,12274 | 1,139584 | -0,98765 | -1,06818 | -0,49243 |
| ***Tp53*** | -0,03313 | -0,694 | -0,51593 | -0,13647 | -0,15904 |
| ***Lima1*** | -0,04401 | -1,10373 | -0,45343 | -0,12859 | 0,183929 |
| ***Aadac*** | 0,009432 | 0,910068 | -0,45339 | -1,1259 | -0,89432 |
| ***Nr1i2*** | -0,21815 | -0,37089 | -0,35606 | -0,21664 | -0,3979 |
| ***Plscr3*** | -0,12548 | -0,40818 | -0,56052 | -0,32793 | -0,15016 |
| ***Pcyt1a*** | -0,15554 | -0,76428 | -0,24717 | -0,25354 | -0,17347 |
| ***Fasn*** | -0,78593 | -0,47232 | -0,08825 | 0,042331 | -0,31419 |
| ***Gpam*** | -0,22696 | -0,85832 | -0,13764 | -0,24223 | -0,15404 |
| ***Rdh16*** | -1,11795 | 0,483381 | -0,21248 | -0,30865 | -0,46387 |
| ***Abcb4*** | 5,65E-05 | -0,36295 | -0,32981 | -0,44952 | -0,4935 |
| ***Nr5a2*** | -0,20778 | -0,56672 | -0,47756 | -0,20032 | -0,18869 |
| ***Esr1*** | -0,8671 | 0,042478 | -0,57455 | -0,17213 | -0,09752 |
| ***Acat2*** | -0,12932 | -0,64627 | -0,3273 | -0,35617 | -0,22326 |
| ***Plpp3*** | -0,29058 | -0,36186 | -0,48651 | -0,36117 | -0,18591 |
| ***Kng1*** | -0,41652 | -0,33195 | -0,41554 | -0,32952 | -0,19744 |
| ***Akr1b1*** | 0,044134 | -0,50866 | -0,45581 | -0,40216 | -0,37285 |
| ***Gk*** | -0,33561 | 0,276574 | -0,57982 | -0,56334 | -0,51153 |
| ***Apaf1*** | -0,37001 | -0,05315 | -0,54746 | -0,3873 | -0,39245 |
| ***Napepld*** | -0,19458 | -1,27637 | -0,57216 | 0,121144 | 0,160326 |
| ***Bcl2*** | -0,06053 | -0,19298 | -1,1254 | -0,45546 | 0,040528 |
| ***Snrk*** | -0,34232 | -0,39845 | -0,24646 | -0,33381 | -0,48073 |
| ***Txnip*** | 0,181313 | 0,06371 | 0,261129 | -1,08844 | -1,22374 |
| ***Irs1*** | -0,02384 | -0,72107 | -0,35943 | -0,17462 | -0,53636 |
| ***Nceh1*** | 0,132946 | -1,31178 | -0,44293 | -0,22489 | 0,021838 |
| ***Casp8*** | 0,183469 | -0,91967 | -0,74514 | -0,21352 | -0,13342 |
| ***St3gal5*** | -0,26422 | -0,5203 | -0,27124 | -0,28675 | -0,48801 |
| ***Nr1h4*** | 0,255714 | -0,60739 | -0,50502 | -0,55488 | -0,43199 |
| ***Dab2ip*** | -0,21214 | -0,84962 | -0,61871 | -0,14876 | -0,0223 |
| ***Cxcl12*** | -0,31424 | 0,331944 | -0,47419 | -0,75144 | -0,67271 |
| ***Acer3*** | -0,57307 | -0,42693 | -0,27763 | -0,31425 | -0,3316 |
| ***Ucp2*** | 0,348379 | -0,17992 | -1,08821 | -0,76601 | -0,25163 |
| ***Asah1*** | -0,38117 | -1,25264 | -0,40556 | -0,00066 | 0,101994 |
| ***Hpgd*** | -0,56275 | -0,65989 | 0,03402 | -0,31819 | -0,44264 |
| ***Nr2f2*** | -0,02633 | -0,4037 | -0,5152 | -0,52636 | -0,50426 |
| ***Smpd3*** | -0,30263 | -1,14887 | -0,67039 | -0,07308 | 0,162832 |
| ***Arntl*** | -0,28962 | -1,1286 | -0,28489 | -0,08829 | -0,24788 |
| ***Apom*** | -0,33283 | -0,1507 | -0,93804 | -0,45161 | -0,17364 |
| ***Plaat3*** | -0,25542 | -0,37084 | -0,45793 | -0,60211 | -0,38193 |
| ***Agmo*** | -0,48299 | 0,599428 | -0,96118 | -0,65029 | -0,58035 |
| ***Pon1*** | -0,20672 | 0,160498 | 0,102142 | -0,76871 | -1,36881 |
| ***Mertk*** | -0,64951 | -0,62557 | -0,2044 | 0,043208 | -0,66573 |
| ***Fas*** | -0,26124 | -0,37775 | -0,62657 | -0,563 | -0,29446 |
| ***Nr1i3*** | -0,76069 | 0,127178 | -0,24139 | -0,64651 | -0,60801 |
| ***Gck*** | -0,2082 | 0,481251 | -1,06026 | -0,70313 | -0,69032 |
| ***Aldh8a1*** | -0,129 | 0,635762 | -0,37848 | -0,93457 | -1,40599 |
| ***Tlr5*** | 0,089034 | -1,42584 | -0,5282 | -0,17704 | -0,24871 |
| ***App*** | -0,64282 | -0,94085 | -0,48604 | -0,276 | -0,02858 |
| ***Mgll*** | -0,06135 | -0,14408 | -0,90209 | -0,639 | -0,63762 |
| ***Pltp*** | -0,54254 | -0,02021 | -0,53789 | -0,73388 | -0,55614 |
| ***Utp14c*** | -0,88622 | -0,18311 | -0,44022 | -0,40626 | -0,47723 |
| ***Avpr1a*** | -0,95307 | 0,110293 | -0,10142 | -0,48861 | -1,0545 |
| ***Bhmt*** | -0,14246 | 1,050284 | -1,5266 | -1,09965 | -0,7783 |
| ***Pmp22*** | 0,090633 | -0,13117 | -1,17002 | -0,8847 | -0,40893 |
| ***Dhcr24*** | -0,3609 | -1,20499 | -0,57054 | -0,21409 | -0,17061 |
| ***Vldlr*** | -1,65317 | -0,33405 | 0,599907 | -0,31061 | -0,84224 |
| ***Tm6sf2*** | -1,19522 | -0,84065 | -0,71426 | 0,182455 | -0,03303 |
| ***Lipa*** | -0,39884 | -1,07622 | -0,60296 | -0,43253 | -0,17624 |
| ***Abca3*** | -0,76772 | -0,5493 | -0,55935 | -0,44614 | -0,37678 |
| ***Tnfsf10*** | -0,76842 | -0,42081 | -0,89224 | -0,39771 | -0,27281 |
| ***Ppargc1a*** | -0,79412 | -0,38501 | -0,49487 | -0,42794 | -0,66493 |
| ***Mfsd2a*** | -0,98722 | -0,83224 | -0,51057 | -0,13327 | -0,3093 |
| ***Akr1b7*** | -1,02823 | -1,0534 | -0,25297 | -0,03276 | -0,44382 |
| ***Pparg*** | 0,308282 | -1,26225 | -0,77772 | -0,62831 | -0,46886 |
| ***Pla1a*** | -0,50878 | -0,65272 | -0,89701 | -0,51342 | -0,26201 |
| ***Ntrk2*** | -2,47005 | -0,14335 | -0,30147 | -0,01801 | -0,01306 |
| ***Agtr1*** | -0,49251 | -0,62224 | -0,73842 | -0,69699 | -0,43405 |
| ***Pnpla3*** | -1,66471 | -0,09853 | -0,52087 | -0,51328 | -0,23482 |
| ***Cyp7a1*** | -0,10229 | 1,351961 | -0,28199 | -1,67746 | -2,37028 |
| ***Tsc22d3*** | -0,39389 | -0,33672 | -0,72255 | -0,8845 | -0,81437 |
| ***Acaca*** | -0,81725 | -0,50812 | -0,39237 | -0,72828 | -0,71412 |
| ***Cdkn2c*** | -0,55609 | -0,24207 | -0,83805 | -0,84092 | -0,73512 |
| ***Rxrg*** | -0,82884 | -0,95299 | -0,79999 | -0,3721 | -0,44445 |
| ***Prkca*** | -0,78907 | -1,45442 | -0,55646 | -0,30357 | -0,30056 |
| ***G6pc*** | -0,13059 | 0,340476 | -0,01617 | -1,58284 | -2,08482 |
| ***Gckr*** | -0,33706 | -0,0648 | -1,0615 | -1,10045 | -0,98599 |
| ***Slc2a2*** | -0,29992 | -0,64015 | -0,84233 | -1,01141 | -0,84855 |
| ***Ces1g*** | -0,50341 | 0,343339 | -0,57869 | -1,01469 | -1,90591 |
| ***Cyp4a11*** | -0,5732 | 0,642984 | -0,96937 | -1,57802 | -1,1825 |
| ***Afp*** | 0,056886 | -0,48276 | -1,36436 | -1,32084 | -0,57073 |
| ***Rbp1*** | -0,73896 | -0,81934 | -1,09913 | -0,56668 | -0,49116 |
| ***Srd5a2*** | -0,44862 | -1,36603 | -0,96258 | -0,54396 | -0,40065 |
| ***Nqo1*** | -1,16194 | -0,75202 | -0,53121 | -0,54596 | -0,73791 |
| ***Bche*** | -0,81906 | -0,67084 | -0,88411 | -0,88749 | -0,56786 |
| ***Srgap3*** | -0,68708 | -1,70664 | -0,87661 | -0,40182 | -0,41003 |
| ***Ptgfr*** | -0,27706 | -0,57687 | -1,61591 | -1,34509 | -0,50328 |
| ***Ptgds*** | -1,88192 | -0,85589 | -0,79971 | -0,40544 | -0,41432 |
| ***Grk5*** | -0,03453 | -2,04594 | -1,58535 | -0,8047 | -0,20052 |
| ***Csf1*** | -0,26315 | -1,99638 | -1,44569 | -0,86568 | -0,10769 |
| ***Cers6*** | -2,07437 | -1,2178 | -0,63313 | -0,40597 | -0,38715 |
| ***Hexb*** | -1,93124 | -1,05373 | -0,57878 | -0,6661 | -0,6034 |
| ***Acot2*** | -0,6759 | -2,39627 | -1,1104 | -0,58939 | -0,27743 |
| ***Abcb1b*** | -2,13704 | -1,12408 | -0,84069 | -0,67715 | -0,42022 |
| ***Abcc4*** | -0,77985 | -1,81574 | -1,03435 | -0,8991 | -1,03666 |
| ***Lepr*** | -1,18575 | -0,92697 | -0,63828 | -1,08262 | -1,81091 |
| ***Cyp17a1*** | -3,07659 | -1,66558 | 0,31134 | 0,14851 | -1,75611 |
| ***Ar*** | -1,20432 | -1,20092 | -1,31942 | -1,29383 | -1,37866 |
| ***Cyp4a14*** | -0,75272 | -0,24857 | -1,13345 | -2,35025 | -2,68532 |
| ***Vnn1*** | -0,56203 | -1,95141 | -2,38867 | -1,60905 | -0,88811 |
| ***Abcb1*** | -3,15988 | -0,99045 | -1,36827 | -1,08103 | -0,80522 |
| ***Abcd2*** | -3,21303 | -0,4612 | -1,06522 | -1,32308 | -1,44821 |
| ***Prlr*** | -1,84625 | -0,84082 | -1,60129 | -1,87303 | -1,77616 |
| ***Cd36*** | -1,76494 | -1,54792 | -2,50848 | -1,88358 | -1,04423 |
| ***Cyp3a5*** | -4,0533 | -3,72033 | -2,12901 | 0,626905 | -0,99918 |
| ***Fmo3*** | -5,79658 | -3,84383 | -4,39118 | -4,23965 | -3,20229 |

**Table S22:** Activation Z-score of genes involved in hepatic steatosis male vs. female. Activation Z-score was calculated with IPA software from Qiagen.

| **Pathway Summary** | -0,356 | -0,429 | -1,409 | 0,982 | 1,866 |
| --- | --- | --- | --- | --- | --- |
| **genes in the hepatic steatosis network** | **0 h** | **24 h** | **48 h** | **72 h** | **96 h** |
| ***Obp2b*** | 2,916129 | 1,226669 | 0,565316 | 0,405337 | 0,308385 |
| ***Serpina1*** | 1,141492 | 1,2407 | 1,497335 | 0,705409 | 0,48324 |
| ***Cyp2e1*** | 0,155034 | 0,940182 | 2,273941 | 0,984777 | -0,50221 |
| ***Hmox1*** | 0,452339 | 0,564182 | 1,329974 | 0,634012 | 0,537807 |
| ***Fabp4*** | -0,12234 | 1,045039 | 1,446707 | 0,929734 | 0,149975 |
| ***Pkm*** | -0,08209 | 0,184047 | 0,898776 | 1,488772 | 0,741155 |
| ***Gstp1*** | 1,882254 | 0,337857 | 0,177296 | 0,244776 | 0,194589 |
| ***Jun*** | 0,719868 | 0,883187 | 0,549306 | 0,296255 | 0,375915 |
| ***Lgals3*** | -0,20388 | 1,006761 | -0,00589 | 0,613036 | 0,930013 |
| ***Cav1*** | 0,773773 | 0,658437 | 0,150822 | 0,097915 | 0,546004 |
| ***Sort1*** | 0,866583 | 0,277318 | 0,31254 | 0,372022 | 0,372949 |
| ***Cd44*** | -0,02436 | -0,27368 | 0,553021 | 0,715762 | 1,002493 |
| ***Klf2*** | -0,01321 | 0,284792 | 0,579505 | 0,353681 | 0,724173 |
| ***Tiparp*** | -0,69317 | 0,82824 | 0,991947 | 0,726471 | 0,063382 |
| ***Spp1*** | 0,356681 | 0,816593 | -0,63844 | 0,132484 | 1,13767 |
| ***Socs3*** | 0,648692 | 0,44729 | 0,310238 | 0,230759 | 0,070863 |
| ***Upp1*** | -0,26582 | 0,36771 | 1,075423 | 0,581889 | -0,09338 |
| ***Tlr4*** | -0,14703 | -0,00808 | 0,78129 | 0,494066 | 0,451944 |
| ***Il22*** | 0,257143 | 0,221181 | 0,380083 | 0,32858 | 0,361546 |
| ***Egln1*** | -0,0439 | 0,344829 | 0,699547 | 0,449301 | -0,05074 |
| ***Phyh*** | 0,283241 | 1,296553 | 0,151206 | -0,14818 | -0,18579 |
| ***Gcgr*** | 0,358078 | 0,957776 | 0,453834 | -0,14892 | -0,29726 |
| ***Kdm3a*** | 0,138353 | 0,593175 | 0,601079 | 0,264674 | -0,31629 |
| ***Ptger4*** | 0,153896 | 0,428614 | 0,404995 | 0,173284 | 0,085753 |
| ***Sirt1*** | -0,0669 | 0,763695 | 0,318872 | 0,227024 | -0,0192 |
| ***Il25*** | 0,095375 | -0,01305 | 0,212678 | 0,331942 | 0,577525 |
| ***Acsl1*** | 0,275928 | 1,416515 | 0,136382 | -0,25224 | -0,37723 |
| ***Steap4*** | 0,210981 | 1,010364 | -0,21656 | -0,03034 | 0,213214 |
| ***Pex11a*** | 0,683714 | 0,143333 | 0,334841 | 0,140108 | -0,11715 |
| ***Egln3*** | -0,34663 | 0,30036 | 0,814625 | 0,637779 | -0,25935 |
| ***Dpp7*** | 0,423313 | 0,208382 | 0,150052 | 0,089273 | 0,272534 |
| ***Vhl*** | 0,468378 | 0,633775 | 0,379216 | -0,16675 | -0,18123 |
| ***Lpl*** | 0,511842 | 0,147046 | 0,090463 | 0,161693 | 0,189924 |
| ***Pde4a*** | 0,203206 | 0,36449 | 0,453103 | -0,07201 | 0,137968 |
| ***Cavin3*** | 0,024777 | 0,16728 | 0,392514 | 0,210198 | 0,283498 |
| ***Pde3a*** | 0,106559 | 0,338179 | 0,257521 | 0,040993 | 0,320154 |
| ***Ffar4*** | -0,31033 | 0,062398 | 0,941238 | 0,43388 | -0,07842 |
| ***Epm2a*** | -0,25402 | 0,441024 | 0,401765 | 0,297824 | 0,114253 |
| ***Atf4*** | -0,07623 | 0,409375 | 0,25378 | 0,177375 | 0,221857 |
| ***Krt8*** | 0,399849 | -0,09516 | -0,03355 | 0,281915 | 0,392077 |
| ***Alox5*** | 0,097119 | 0,514201 | -0,04708 | 0,266093 | 0,107334 |
| ***Acads*** | 0,342201 | 0,496937 | -0,02047 | -0,052 | 0,157163 |
| ***Uts2*** | -0,06382 | 0,498859 | 0,292468 | 0,161809 | 0,015595 |
| ***Pdk2*** | 0,128752 | 0,363429 | 0,460486 | 0,134277 | -0,20922 |
| ***Il17a*** | -0,14899 | 0,064948 | 0,589141 | 0,131666 | 0,233734 |
| ***Gnmt*** | -0,05569 | 0,88987 | 0,999099 | -0,16203 | -0,83998 |
| ***Med13*** | 0,168915 | 0,092668 | 0,386759 | 0,324642 | -0,15625 |
| ***Slc39a14*** | -0,11062 | 0,456807 | 0,326991 | 0,144975 | -0,01222 |
| ***Npc1l1*** | 0,22953 | 0,339241 | 0,066838 | 0,126331 | 0,037662 |
| ***Ucn3*** | 0,064518 | 0,13883 | 0,039449 | 0,248824 | 0,301539 |
| ***Ddc*** | 0,206844 | -0,16771 | 0,632523 | 0,134063 | -0,03427 |
| ***Gatm*** | -0,05157 | 0,331376 | 0,199776 | -0,05124 | 0,333 |
| ***Impdh1*** | 0,263911 | -0,07666 | 0,293344 | 0,209357 | 0,057139 |
| ***Sod1*** | -0,00286 | 0,235516 | 0,313475 | 0,189561 | -0,0042 |
| ***Adrb3*** | 0,440709 | 0,256087 | 0,119164 | 0,018807 | -0,11038 |
| ***Il1b*** | 0,263769 | 0,310882 | -0,03707 | 0,045763 | 0,139904 |
| ***Cidea*** | 0,047785 | -0,07975 | 0,416233 | 0,033413 | 0,292272 |
| ***Lpin1*** | 0,304783 | 0,830156 | 0,490199 | -0,31185 | -0,64224 |
| ***Pafah1b1*** | 0,021576 | 0,182636 | 0,202084 | 0,218195 | 0,034888 |
| ***Cntf*** | 0,152345 | 0,005822 | 0,321655 | 0,103383 | 0,070471 |
| ***Clpp*** | 0,389331 | 0,082392 | 0,041418 | 0,075206 | 0,029805 |
| ***Hint2*** | 0,431696 | 0,541988 | 0,088866 | -0,11277 | -0,33827 |
| ***Por*** | -0,22385 | 0,116112 | 0,419562 | 0,346398 | -0,05581 |
| ***Abca1*** | 0,121641 | 0,292213 | 0,159397 | 0,278491 | -0,26018 |
| ***Lrp6*** | 0,182804 | 0,272129 | 0,053366 | 0,159756 | -0,09003 |
| ***Sod2*** | 0,112468 | 0,368751 | 0,010485 | 0,083511 | -0,01597 |
| ***Lipc*** | -0,33561 | 0,056819 | 0,199002 | 0,359127 | 0,275048 |
| ***Rbm38*** | 0,104674 | 0,258571 | 0,141681 | 0,126556 | -0,10469 |
| ***Abhd5*** | -0,06357 | 0,116803 | 0,159989 | 0,048191 | 0,257255 |
| ***Ern1*** | 0,183891 | 0,516285 | -0,01345 | -0,08706 | -0,08846 |
| ***Tysnd1*** | 0,319976 | 0,170525 | 0,024379 | 0,023928 | -0,02769 |
| ***Cidec*** | 0,307489 | -0,93111 | 0,683086 | 0,239585 | 0,210129 |
| ***Ppargc1b*** | 0,222911 | 0,302329 | 0,065047 | -0,02786 | -0,06079 |
| ***Ins*** | -0,33103 | 0,458564 | 0,005813 | -0,03666 | 0,391596 |
| ***Noct*** | -0,88356 | -0,31637 | 0,390114 | 0,667259 | 0,621873 |
| ***Prkar2b*** | -0,27859 | 0,108796 | 0,194686 | 0,282446 | 0,160946 |
| ***Pex13*** | 0,113692 | 0,118337 | 0,446837 | -0,1045 | -0,11591 |
| ***Igf1r*** | -0,0981 | -0,0826 | 0,279362 | 0,110573 | 0,242523 |
| ***Pde4c*** | 0,158342 | 0,347474 | 0,110752 | -0,06979 | -0,0968 |
| ***Man2c1*** | 0,025947 | 0,005506 | 0,079637 | 0,105157 | 0,226213 |
| ***Acox1*** | 0,201106 | 0,527651 | 0,197574 | -0,20825 | -0,29447 |
| ***Mthfr*** | 0,304212 | 0,08496 | 0,063125 | -0,11523 | 0,083403 |
| ***Adcyap1*** | -0,1195 | 0,290491 | -0,08756 | 0,037486 | 0,296229 |
| ***Slc27a4*** | 0,152291 | -0,23916 | 0,166582 | 0,259891 | 0,071442 |
| ***Acaa2*** | 0,088348 | 1,205352 | -0,2018 | -0,36845 | -0,3216 |
| ***Cbs/cbsl*** | 0,047661 | 0,340664 | -0,1621 | -0,00901 | 0,184036 |
| ***Gpd2*** | 0,076561 | -0,02297 | -0,04922 | 0,139018 | 0,244428 |
| ***Foxo3*** | 0,150165 | 0,280371 | 0,170242 | -0,06662 | -0,14944 |
| ***Atf6*** | 0,142743 | -0,04721 | 0,200829 | 0,112034 | -0,03686 |
| ***Pc*** | 0,020949 | 0,875786 | -0,03364 | -0,34693 | -0,1527 |
| ***Xbp1*** | 0,283165 | 0,006163 | 0,028796 | 0,000284 | 0,033335 |
| ***Rgcc*** | -0,33256 | 0,233761 | 0,130112 | 0,124505 | 0,189548 |
| ***Stk25*** | 0,190238 | -0,10705 | 0,088566 | 0,103183 | 0,059687 |
| ***Clu*** | 0,169921 | 0,158111 | -0,1104 | -0,01523 | 0,119884 |
| ***Atp2a2*** | 0,128012 | -0,22251 | 0,127965 | 0,20986 | 0,076742 |
| ***Rgn*** | -0,25904 | 1,131845 | 0,357521 | -0,2515 | -0,68179 |
| ***Fstl3*** | -0,15628 | -0,07644 | 0,009345 | 0,14553 | 0,352179 |
| ***Fgl1*** | -0,60514 | -0,19903 | 0,468235 | 0,291501 | 0,295674 |
| ***Mapk13*** | -0,10682 | 0,118433 | 0,036508 | -0,03219 | 0,2349 |
| ***Crtc3*** | -0,01524 | 0,346289 | -0,02403 | 0,105226 | -0,1638 |
| ***Npy1r*** | 0,247693 | 0,041606 | 0,077397 | -0,00081 | -0,12691 |
| ***Ghrl*** | -0,25736 | 0,238318 | 0,189848 | 0,054328 | -0,0071 |
| ***Gpt*** | -0,12324 | 0,53074 | -0,11914 | -0,05885 | -0,03134 |
| ***Ghr*** | 0,020345 | 0,862462 | -0,07016 | -0,35706 | -0,26179 |
| ***Ldlr*** | -0,19533 | -0,23531 | 0,05352 | 0,181182 | 0,380566 |
| ***Ikbkg*** | 0,382264 | -0,1386 | 0,21655 | -0,18448 | -0,09849 |
| ***Sidt2*** | -0,1165 | -0,2445 | 0,252335 | 0,298435 | -0,01344 |
| ***Arnt*** | -0,02327 | 0,187907 | -0,00335 | -0,04279 | 0,055936 |
| ***Slc27a5*** | -0,19069 | 0,503285 | 0,492936 | -0,04094 | -0,59322 |
| ***Tnf*** | -0,18177 | -0,4173 | 0,296697 | 0,27893 | 0,183334 |
| ***Acadvl*** | 0,04424 | 0,257297 | 0,054568 | -0,1052 | -0,12314 |
| ***Nr2c2*** | 0,307347 | 0,275021 | -0,17379 | -0,19836 | -0,08375 |
| ***Slc25a13*** | -0,13467 | -0,22342 | 0,44586 | 0,19267 | -0,18293 |
| ***Ptpn11*** | 0,277848 | -0,11732 | -0,15029 | 0,091185 | -0,0196 |
| ***Pnpla2*** | 0,247679 | -0,29319 | 0,098474 | 0,152412 | -0,12929 |
| ***Id1*** | -0,61048 | 0,140221 | -0,5304 | 0,441345 | 0,617336 |
| ***Cat*** | 0,079423 | 0,133675 | -0,05435 | -0,08199 | -0,03185 |
| ***Map3k5*** | 0,220329 | -0,46408 | -0,06448 | 0,353496 | -0,00104 |
| ***Sirt3*** | 0,047248 | 0,309398 | 0,022808 | -0,15282 | -0,19995 |
| ***Mark3*** | 0,182549 | -0,23314 | 0,093471 | 0,075281 | -0,09903 |
| ***Ahr*** | -0,14658 | 0,313906 | -0,04025 | -0,03685 | -0,07687 |
| ***Gprc6a*** | 0,372585 | -0,01791 | -0,22916 | -0,04782 | -0,08179 |
| ***Impdh2*** | -0,01611 | -0,3725 | 0,270461 | 0,077357 | 0,029898 |
| ***Psme3*** | 0,159401 | -0,33118 | 0,063483 | 0,041861 | 0,049522 |
| ***Ncoa5*** | 0,369041 | -0,18318 | -0,06476 | -0,21848 | 0,073363 |
| ***Adora1*** | -0,02515 | 0,460472 | 0,296181 | -0,19368 | -0,5675 |
| ***Epas1*** | 0,107156 | 0,425131 | -0,27261 | -0,08373 | -0,22467 |
| ***Scg5*** | -0,41317 | 0,053442 | -0,02387 | 0,168028 | 0,146947 |
| ***Sirt4*** | -0,21548 | 0,198685 | 0,044321 | -0,10078 | 0,000804 |
| ***Bscl2*** | 0,024949 | 0,242515 | -0,16808 | -0,0596 | -0,11227 |
| ***Ifnar2*** | -0,12873 | 0,270189 | -0,05546 | -0,12597 | -0,06578 |
| ***Osmr*** | -0,45264 | -0,47341 | 0,071939 | 0,329877 | 0,398829 |
| ***Srebf1*** | -0,75619 | 0,685746 | 0,064183 | -0,03825 | -0,08533 |
| ***Nucks1*** | 0,10991 | -0,29242 | 0,016085 | 0,008675 | 0,026766 |
| ***Pemt*** | -0,23697 | 0,989664 | 0,319489 | -0,40521 | -0,8003 |
| ***Pde7b*** | -0,5789 | 0,44472 | 0,063145 | 0,002435 | -0,06997 |
| ***Bax*** | 0,015485 | -0,26442 | 0,05446 | -0,0615 | 0,110614 |
| ***C3*** | -0,17852 | 8,03E-05 | -0,12049 | 0,058399 | 0,081334 |
| ***Macroh2a1*** | -0,01885 | -0,18706 | 0,090669 | -0,05809 | 0,002547 |
| ***Cspg4*** | -0,38176 | 0,127018 | 0,219829 | -0,19893 | 0,058491 |
| ***S100a10*** | -0,0297 | -0,33488 | -0,06532 | 0,123405 | 0,107003 |
| ***Ttc39b*** | -0,21905 | 0,019599 | -0,13306 | 0,025097 | 0,10313 |
| ***Lats2*** | 0,542567 | -0,35082 | -0,11293 | -0,21854 | -0,07599 |
| ***Hnf1a*** | 0,141772 | -0,46934 | 0,133149 | 0,073791 | -0,10086 |
| ***Pdhb*** | 0,06376 | 0,009939 | 0,002782 | -0,12147 | -0,19501 |
| ***Sik3*** | 0,120441 | -0,29053 | -0,03961 | 0,040252 | -0,08242 |
| ***Pex2*** | 0,068347 | -0,15174 | 0,080045 | -0,24601 | -0,03156 |
| ***Gpd1*** | 0,25139 | 0,157219 | -0,22075 | -0,27529 | -0,23447 |
| ***Ppara*** | 0,284157 | 0,016818 | -0,01524 | -0,10652 | -0,51136 |
| ***Gna11*** | 0,117817 | -0,38846 | -0,07016 | 0,053208 | -0,05876 |
| ***Cpt2*** | 0,166767 | -0,26646 | 0,024149 | -0,12539 | -0,15092 |
| ***Miga2*** | 0,115721 | -0,13483 | -0,11608 | -0,28095 | 0,058656 |
| ***Pde7a*** | 0,482914 | -0,14448 | -0,26467 | -0,27109 | -0,17304 |
| ***Apoe*** | 0,00429 | 0,122289 | -0,03966 | -0,30261 | -0,15475 |
| ***Hadha*** | 0,048218 | -0,04795 | -0,04955 | -0,1482 | -0,18075 |
| ***Gstz1*** | -0,02332 | 0,812286 | -0,42883 | -0,45182 | -0,28936 |
| ***Pla2g15*** | -0,18275 | -0,48308 | 0,281231 | -0,05829 | 0,056042 |
| ***Srsf2*** | -0,27629 | -0,46884 | 0,102153 | 0,118632 | 0,111697 |
| ***Elovl5*** | -0,2241 | 0,445751 | -0,17497 | -0,27147 | -0,19038 |
| ***Lmna*** | -0,11056 | -0,49526 | -0,03898 | 0,036944 | 0,186785 |
| ***Pde3b*** | -0,06062 | 0,05197 | -0,11567 | -0,03503 | -0,27351 |
| ***Nr0b2*** | -0,35622 | 0,233567 | 0,047325 | -0,39205 | 0,021575 |
| ***Stat6*** | 0,273005 | -0,43594 | -0,2113 | -0,07114 | -0,00908 |
| ***Mmut*** | -0,15988 | 0,362459 | -0,26539 | -0,22667 | -0,17404 |
| ***Acadm*** | -0,03751 | 0,003338 | 0,012985 | -0,22625 | -0,21789 |
| ***Acss2*** | -0,47749 | 0,754739 | -0,27084 | -0,25899 | -0,21873 |
| ***Pitpna*** | -0,04177 | -0,55117 | 0,005303 | 0,079987 | 0,023119 |
| ***Nos3*** | -0,44887 | 0,068903 | -0,05055 | -0,06996 | 0,013571 |
| ***Hsd17b13*** | -0,0717 | -0,33975 | -0,02248 | -0,04596 | -0,0319 |
| ***Insr*** | -0,04304 | -0,30847 | -0,01885 | -0,02239 | -0,12176 |
| ***Helz2*** | 0,089805 | -0,19073 | -0,25008 | -0,09299 | -0,08429 |
| ***Adk*** | -0,11049 | 0,108394 | -0,26408 | -0,1524 | -0,13424 |
| ***Hmgcr*** | -1,05361 | 0,128838 | 0,194174 | -0,00519 | 0,16514 |
| ***Acox2*** | 0,207147 | -0,5143 | -0,00712 | -0,1187 | -0,13892 |
| ***Pten*** | -0,06088 | -0,2313 | -0,00484 | -0,14557 | -0,13593 |
| ***Xpa*** | -0,01753 | 0,002382 | -0,12123 | -0,31444 | -0,14139 |
| ***Gucy2c*** | -0,44326 | 0,015786 | 0,131384 | -0,36043 | 0,050101 |
| ***Akt1*** | -0,12742 | -0,47145 | -0,12531 | -0,0356 | 0,152021 |
| ***Ifnar1*** | 0,040489 | -0,36728 | -0,18736 | -0,0778 | -0,0215 |
| ***Hsd11b1*** | -0,18894 | 0,270435 | -0,05613 | -0,22592 | -0,44326 |
| ***Tnfrsf1a*** | -0,28457 | -0,31858 | 0,02452 | 0,128835 | -0,232 |
| ***Acsl4*** | 0,022254 | -0,66087 | -0,26081 | -0,00454 | 0,213346 |
| ***Lbp*** | -0,33897 | -0,19207 | -0,26068 | -0,01096 | 0,109726 |
| ***Pank1*** | -0,19551 | 0,55274 | -0,18486 | -0,32048 | -0,55342 |
| ***Rora*** | 0,248717 | 0,183908 | -0,43177 | -0,19966 | -0,52553 |
| ***Jak2*** | -0,02781 | -0,30249 | -0,25278 | 0,041496 | -0,19887 |
| ***Bbs2*** | -0,59891 | 0,325985 | -0,21417 | -0,12939 | -0,13956 |
| ***Scd*** | -0,13432 | 0,289914 | 0,097704 | -0,38032 | -0,63388 |
| ***Fbxw7*** | -0,10334 | -0,61396 | -0,06142 | 0,143038 | -0,12559 |
| ***Pank2*** | -0,15873 | -0,06449 | -0,2655 | -0,14184 | -0,14488 |
| ***Il6st*** | 0,010569 | -0,39101 | -0,17124 | -0,18416 | -0,04298 |
| ***Arv1*** | -0,01046 | -0,2308 | -0,34189 | -0,03724 | -0,161 |
| ***Blvra*** | -0,08281 | -0,34504 | -0,16895 | -0,08781 | -0,11185 |
| ***Pck1*** | 0,022757 | 2,085707 | -0,98927 | -1,0657 | -0,8563 |
| ***Acot13*** | -0,20654 | -0,48348 | -0,03027 | -0,11397 | 0,00707 |
| ***Decr1*** | 0,10697 | 0,084374 | -0,32973 | -0,45541 | -0,24776 |
| ***Ccl2*** | 0,066708 | -1,28911 | -0,39182 | 0,091752 | 0,678362 |
| ***Nrg4*** | -0,42351 | -0,22017 | -0,03922 | -0,15685 | -0,03866 |
| ***Slc22a5*** | 0,026135 | -0,21294 | 0,023082 | -0,34764 | -0,36809 |
| ***Pdha1*** | -0,04335 | -0,37375 | -0,31533 | -0,27336 | 0,103624 |
| ***Nr1h3*** | 0,045567 | -0,18155 | -0,28072 | -0,24717 | -0,25165 |
| ***Plin2*** | 0,099249 | -0,88332 | -0,13492 | -0,13418 | 0,130005 |
| ***Acadl*** | -0,20704 | -0,55124 | -0,02583 | -0,07497 | -0,06891 |
| ***Sirpa*** | -0,16171 | 0,135121 | -0,23706 | -0,44892 | -0,22267 |
| ***Adipor2*** | -0,11821 | 0,070816 | -0,11016 | -0,35886 | -0,44666 |
| ***Mttp*** | -0,05097 | -0,37223 | -0,1664 | -0,00399 | -0,37249 |
| ***Dnajc7*** | -0,04284 | -0,5412 | 0,021931 | -0,29237 | -0,12199 |
| ***Fancc*** | -0,24745 | -0,58998 | 0,013134 | 0,010376 | -0,1986 |
| ***Cideb*** | -0,0803 | -0,06647 | -0,34073 | -0,26516 | -0,27135 |
| ***Oma1*** | 0,026562 | -0,72651 | 0,001576 | -0,11092 | -0,22281 |
| ***Casp3*** | -0,2797 | 0,3566 | -0,38052 | -0,58323 | -0,14989 |
| ***Lcn2*** | -2,07597 | -0,14677 | 0,223995 | 0,571115 | 0,390084 |
| ***Hsd17b4*** | 0,162203 | -0,53786 | -0,16633 | -0,25361 | -0,24445 |
| ***Adrb2*** | -0,01087 | -0,54006 | -0,22739 | -0,07428 | -0,2077 |
| ***Adra1a*** | -0,26925 | 0,1351 | -0,25675 | -0,42211 | -0,26748 |
| ***Grb14*** | 0,12025 | -0,74204 | -0,46291 | -0,0726 | 0,074658 |
| ***Abcb11*** | -0,28694 | 0,895383 | -0,1812 | -0,56721 | -0,9809 |
| ***Fgfr2*** | -0,35329 | 0,060919 | -0,3489 | -0,43475 | -0,05061 |
| ***Fabp5*** | -0,71247 | 0,616043 | -0,73515 | -0,22654 | -0,06958 |
| ***Fgf1*** | -0,08053 | 0,240161 | -0,10593 | -0,55711 | -0,6425 |
| ***Prkab1*** | -0,21304 | 0,004241 | -0,24166 | -0,39474 | -0,30941 |
| ***Rbpj*** | -0,2803 | -0,17942 | -0,2094 | -0,35165 | -0,18262 |
| ***Pde4b*** | -0,91394 | 1,051372 | -0,18185 | -0,52769 | -0,66602 |
| ***Tlr2*** | -0,22506 | -0,32334 | -0,54692 | -0,18222 | 0,019467 |
| ***Sucnr1*** | 0,08957 | 0,879468 | -0,87695 | -0,76374 | -0,59107 |
| ***Elovl2*** | -0,1327 | 0,596818 | -0,41057 | -0,5967 | -0,77845 |
| ***Cpt1a*** | 0,141407 | -0,3976 | -0,44114 | -0,41383 | -0,24206 |
| ***Ehhadh*** | -0,21017 | -0,18064 | -0,0393 | -0,47618 | -0,4945 |
| ***Pde8a*** | -0,15892 | -0,55505 | -0,20848 | -0,11567 | -0,37352 |
| ***Sult1e1*** | -0,77274 | 0,088906 | 0,193589 | -0,32624 | -0,62783 |
| ***F8*** | -0,47234 | -0,24103 | -0,06107 | -0,41908 | -0,26044 |
| ***Agpat2*** | -0,03707 | -0,32925 | -0,47236 | -0,26184 | -0,35562 |
| ***Ticam1*** | 0,103459 | -0,8443 | -0,31803 | -0,16079 | -0,2695 |
| ***Rorc*** | -0,31607 | -0,26802 | 0,325609 | -0,41753 | -0,81895 |
| ***Soat2*** | -0,2558 | -0,20424 | -0,22728 | -0,25872 | -0,55695 |
| ***Il18*** | -0,15491 | 0,211891 | -0,75962 | -0,60076 | -0,21168 |
| ***Tp53*** | -0,03313 | -0,694 | -0,51593 | -0,13647 | -0,15904 |
| ***Nr1i2*** | -0,21815 | -0,37089 | -0,35606 | -0,21664 | -0,3979 |
| ***Pcyt1a*** | -0,15554 | -0,76428 | -0,24717 | -0,25354 | -0,17347 |
| ***Fasn*** | -0,78593 | -0,47232 | -0,08825 | 0,042331 | -0,31419 |
| ***Esr1*** | -0,8671 | 0,042478 | -0,57455 | -0,17213 | -0,09752 |
| ***Gk*** | -0,33561 | 0,276574 | -0,57982 | -0,56334 | -0,51153 |
| ***Camkk2*** | -0,20439 | -1,09832 | -0,43801 | 0,090404 | -0,07779 |
| ***Txnip*** | 0,181313 | 0,06371 | 0,261129 | -1,08844 | -1,22374 |
| ***Nr1h4*** | 0,255714 | -0,60739 | -0,50502 | -0,55488 | -0,43199 |
| ***Ucp2*** | 0,348379 | -0,17992 | -1,08821 | -0,76601 | -0,25163 |
| ***Prkag2*** | -0,50905 | -0,84728 | -0,13855 | -0,19606 | -0,26978 |
| ***Arntl*** | -0,28962 | -1,1286 | -0,28489 | -0,08829 | -0,24788 |
| ***Pnrc2*** | -0,27433 | -0,21556 | -0,47448 | -0,59026 | -0,51233 |
| ***Plaat3*** | -0,25542 | -0,37084 | -0,45793 | -0,60211 | -0,38193 |
| ***Dpp4*** | -0,50342 | -0,23887 | -0,52092 | -0,48077 | -0,34043 |
| ***Nr1i3*** | -0,76069 | 0,127178 | -0,24139 | -0,64651 | -0,60801 |
| ***Gck*** | -0,2082 | 0,481251 | -1,06026 | -0,70313 | -0,69032 |
| ***Tlr5*** | 0,089034 | -1,42584 | -0,5282 | -0,17704 | -0,24871 |
| ***Bhmt*** | -0,14246 | 1,050284 | -1,5266 | -1,09965 | -0,7783 |
| ***Tm6sf2*** | -1,19522 | -0,84065 | -0,71426 | 0,182455 | -0,03303 |
| ***Mat1a*** | -0,30661 | 0,86826 | -0,50442 | -1,49196 | -1,21545 |
| ***Lipa*** | -0,39884 | -1,07622 | -0,60296 | -0,43253 | -0,17624 |
| ***Ppargc1a*** | -0,79412 | -0,38501 | -0,49487 | -0,42794 | -0,66493 |
| ***Mfsd2a*** | -0,98722 | -0,83224 | -0,51057 | -0,13327 | -0,3093 |
| ***Akr1b7*** | -1,02823 | -1,0534 | -0,25297 | -0,03276 | -0,44382 |
| ***Pparg*** | 0,308282 | -1,26225 | -0,77772 | -0,62831 | -0,46886 |
| ***Agtr1*** | -0,49251 | -0,62224 | -0,73842 | -0,69699 | -0,43405 |
| ***Pnpla3*** | -1,66471 | -0,09853 | -0,52087 | -0,51328 | -0,23482 |
| ***Tsc22d3*** | -0,39389 | -0,33672 | -0,72255 | -0,8845 | -0,81437 |
| ***Acaca*** | -0,81725 | -0,50812 | -0,39237 | -0,72828 | -0,71412 |
| ***Plk1*** | 0,395094 | -0,02675 | -2,51075 | -1,02875 | -0,25865 |
| ***G6pc*** | -0,13059 | 0,340476 | -0,01617 | -1,58284 | -2,08482 |
| ***Gbp2*** | -0,86438 | -1,00523 | -0,73189 | -0,69396 | -0,19629 |
| ***Pdgfc*** | -0,24588 | -0,79194 | -0,87006 | -1,05117 | -0,55016 |
| ***Rbl1*** | -0,58928 | -0,91153 | -1,10301 | -0,54222 | -0,41017 |
| ***Ces1g*** | -0,50341 | 0,343339 | -0,57869 | -1,01469 | -1,90591 |
| ***Cyp4a11*** | -0,5732 | 0,642984 | -0,96937 | -1,57802 | -1,1825 |
| ***Rgs16*** | -1,2624 | 0,446081 | -0,93258 | -1,38671 | -1,85131 |
| ***Ar*** | -1,20432 | -1,20092 | -1,31942 | -1,29383 | -1,37866 |
| ***Acot1*** | -3,43591 | -0,78669 | -0,8108 | -0,81683 | -0,67339 |
| ***Cyp4a14*** | -0,75272 | -0,24857 | -1,13345 | -2,35025 | -2,68532 |
| ***Ccnd1*** | -2,06339 | -1,47819 | -1,8214 | -1,3163 | -0,59342 |
| ***Steap2*** | -1,37 | -2,82227 | -1,71481 | -1,3912 | -0,91002 |
| ***Cd36*** | -1,76494 | -1,54792 | -2,50848 | -1,88358 | -1,04423 |

**Table S23:** Activation Z-score of proteins involved in fatty acid metabolism male vs. female. Activation Z-score was calculated with IPA software from Qiagen.

| **Pathway Summary** | -0,607 | -1,005 | -1,229 | -0,319 | -0,433 |
| --- | --- | --- | --- | --- | --- |
| **proteins in the fatty acid metabolism network** | **0 h** | **24 h** | **48 h** | **72 h** | **96 h** |
| **SLCO1B3** | 9,933269 | 1,519721 | 14,51829 | 12,2419 | 16,8349 |
| **SLC25A10** | N/A | 1,58732 | N/A | 2,958949 | 27,66288 |
| **GSTP1** | 6,86094 | 6,433403 | 4,963562 | 3,582487 | 2,55001 |
| **PON1** | 5,379143 | -1,29146 | 4,020848 | 3,809965 | 3,577052 |
| **ME1** | 3,093078 | 2,647354 | 3,215682 | 3,393881 | 1,90172 |
| **FABP1** | 6,053154 | 1,046859 | 3,180378 | 2,118816 | 1,822998 |
| **NUDT7** | 2,489954 | 2,11357 | 2,314331 | 2,588426 | 1,380584 |
| **HSD17B8** | 3,582373 | 1,246456 | 1,758038 | 1,226057 | 2,582128 |
| **CYP2C8** | 2,033738 | 1,140463 | 1,665818 | 1,322788 | 1,329823 |
| **EPHX2** | 1,644387 | 1,304006 | 1,508459 | 1,50264 | 1,467997 |
| **ABAT** | 2,389401 | -1,14232 | 2,132946 | 1,873112 | 1,786624 |
| **MTTP** | 1,713662 | 1,395471 | 1,490981 | 1,158708 | 1,260068 |
| **CYP2E1** | 1,447992 | 1,182643 | 1,328891 | 1,330951 | 1,274404 |
| **CYP4A22** | 1,323678 | 1,603115 | 1,392194 | 1,041138 | 1,191638 |
| **SCP2** | 1,343139 | 1,457684 | 1,376149 | 1,088662 | 1,079751 |
| **FH** | 1,382264 | 1,188151 | 1,286783 | 1,059095 | 1,187506 |
| **GLYAT** | 1,246883 | 1,225533 | 1,277698 | 1,15999 | 1,136217 |
| **ACAA1** | 1,404985 | 1,025449 | 1,266997 | 1,195626 | 1,083982 |
| **EPHX1** | 1,087181 | 1,266837 | 1,190451 | 1,232295 | 1,024527 |
| **AKR1C1/AKR1C2** | 1,443584 | -1,14832 | 1,328085 | 1,599962 | 2,486267 |
| **BAAT** | 6,162764 | -1,30705 | 1,273803 | -1,25824 | -1,17353 |
| **CYP27A1** | -1,10746 | 1,270726 | 1,230232 | 1,1481 | 1,093846 |
| **ACSL1** | -1,02867 | 1,204015 | 1,192691 | 1,133303 | 1,053531 |
| **SUCLG2** | 1,041624 | 1,168107 | 1,093315 | -1,00523 | 1,183798 |
| **ACSM1** | 1,442239 | -1,10595 | 1,31249 | -1,01903 | 1,154036 |
| **PDHB** | 1,484927 | -1,48361 | 1,056859 | 1,374551 | -1,01432 |
| **ACADS** | -1,16066 | 1,043016 | 1,095458 | 1,013216 | -1,00239 |
| **CYP1A2** | -1,20296 | 1,034577 | 1,082641 | 1,044196 | -1,04595 |
| **ACADVL** | -1,39433 | 1,074727 | 1,04507 | 1,100853 | -1,06107 |
| **DBI** | 1,067842 | -1,29738 | 1,035173 | -1,26747 | 1,078351 |
| **DLST** | -1,21147 | 1,12905 | 1,111508 | -1,00573 | -1,06782 |
| **CPT2** | 1,00547 | -1,05575 | -1,04768 | -1,11747 | 1,097332 |
| **HSD17B4** | -1,08485 | 1,037723 | 1,010027 | -1,10006 | -1,06344 |
| **ATP5PF** | -1,27354 | 1,206059 | 1,067826 | -1,11756 | -1,135 |
| **IL16** | -1,2305 | 1,100174 | 1,042755 | -1,1386 | -1,03362 |
| **Cyp2c54** | -1,27242 | 1,179567 | 1,095292 | -1,21047 | -1,17042 |
| **PCCB** | -1,44922 | 1,184385 | 1,104738 | -1,10418 | -1,26609 |
| **APOE** | 1,386757 | -1,56625 | -1,25116 | -1,35046 | 1,016212 |
| **ANXA6** | -1,24158 | 1,054998 | -1,00375 | -1,00711 | -1,01955 |
| **CANX** | -1,24802 | -1,04434 | -1,07101 | 1,029669 | -1,04539 |
| **ACAA2** | -1,22944 | -1,1139 | 1,043495 | -1,04608 | -1,05174 |
| **ABCD3** | -1,27354 | 1,073769 | -1,0325 | -1,05477 | -1,14028 |
| **MDH1** | -1,24925 | -1,12296 | -1,03386 | 1,010285 | -1,0802 |
| **ACSL5** | -1,36781 | 1,015752 | -1,02408 | -1,05963 | -1,08129 |
| **SLC27A2** | -1,39595 | 1,018598 | -1,03483 | -1,05286 | -1,0929 |
| **CLU** | -1,56022 | -1,08343 | -1,13591 | -1,08624 | 1,000076 |
| **SUCLG1** | -1,2527 | -1,02268 | -1,00005 | -1,11612 | -1,12311 |
| **PCCA** | -1,28063 | -1,03505 | -1,01684 | -1,10886 | -1,11309 |
| **MDH2** | -1,37621 | -1,01754 | -1,01961 | -1,05572 | -1,20048 |
| **GC** | -1,2427 | -1,0565 | -1,10506 | -1,12431 | -1,14606 |
| **SUCLA2** | -1,40282 | -1,0777 | -1,00557 | -1,10284 | -1,10762 |
| **HSPA8** | -1,34774 | -1,07792 | -1,12318 | -1,06946 | -1,07892 |
| **PRDX6** | -1,27106 | -1,14811 | -1,05908 | -1,15106 | -1,13163 |
| **ACAT1** | -1,37874 | -1,17914 | -1,05347 | -1,05319 | -1,1087 |
| **CES1** | -1,41916 | -1,02444 | -1,09682 | -1,17358 | -1,14952 |
| **PITPNM1** | -1,38031 | -1,34043 | -1,1198 | -1,00603 | -1,0364 |
| **RHOA** | -1,245 | -1,14683 | -1,18287 | -1,26674 | -1,15313 |
| **UGT1A10** | -1,44208 | -1,1896 | -1,17523 | -1,29725 | -1,31965 |
| **CBR1** | -1,59879 | -1,07398 | -1,239 | -1,26817 | -1,24626 |
| **ACADL** | -1,62185 | -1,24181 | -1,1833 | -1,21001 | -1,27713 |
| **HACL1** | -1,32022 | -1,54462 | -1,27234 | -1,28269 | -1,18184 |
| **FABP5** | -1,80466 | -1,68977 | -1,47194 | -1,26345 | -1,36353 |
| **RGN** | -1,76745 | -1,68733 | -1,45828 | -1,35929 | -1,3299 |
| **ALB** | -1,41802 | -1,6364 | -1,62211 | -1,50474 | -1,53956 |
| **APOA1** | -1,45626 | -1,32673 | -1,61761 | -1,71124 | -1,64511 |

**Table S24:** Activation Z-score of proteins involved in the metabolism of terpenoids male vs. female. Activation Z-score was calculated with IPA software from Qiagen.

| **Pathway Summary** | -0,835 | -1,071 | -0,751 | -1,992 | -0,966 |
| --- | --- | --- | --- | --- | --- |
| **genes in the metabolism of terpenoid network** | **0h** | **24h** | **48h** | **72h** | **96h** |
| **CYP7B1** | 31,2713 | 6,058976 | 16,8092 | 10,00731 | 4,657266 |
| **G6PC** | 14,70826 | 1,324065 | 12,30242 | 7,972186 | 28,70709 |
| **PON1** | 5,379143 | -1,29146 | 4,020848 | 3,809965 | 3,577052 |
| **HSD17B8** | 3,582373 | 1,246456 | 1,758038 | 1,226057 | 2,582128 |
| **XDH** | 2,626494 | 1,171979 | 2,326047 | 1,455116 | 2,697846 |
| **FDPS** | 2,468791 | 1,059412 | 1,940406 | 1,667676 | 1,572961 |
| **COMT** | 1,969446 | 1,441108 | 1,502034 | 1,457034 | 1,348906 |
| **CYP2C8** | 2,033738 | 1,140463 | 1,665818 | 1,322788 | 1,329823 |
| **CYP2E1** | 1,447992 | 1,182643 | 1,328891 | 1,330951 | 1,274404 |
| **SCP2** | 1,343139 | 1,457684 | 1,376149 | 1,088662 | 1,079751 |
| **ACAA1** | 1,404985 | 1,025449 | 1,266997 | 1,195626 | 1,083982 |
| **AMACR** | 1,120281 | 1,125937 | 1,297034 | 1,204147 | 1,106829 |
| **ACAT2** | 3,021496 | -1,11332 | 1,611311 | 1,027102 | 1,257862 |
| **AKR1C1/AKR1C2** | 1,443584 | -1,14832 | 1,328085 | 1,599962 | 2,486267 |
| **ALDH1A1** | 1,11525 | 1,153403 | 1,120552 | 1,118103 | 1,04725 |
| **PHYH** | 1,58874 | 1,139995 | 1,099729 | -1,08817 | 1,123978 |
| **Aldh1a7** | 1,094762 | -1,0265 | 1,155866 | 1,246114 | 1,305874 |
| **BAAT** | 6,162764 | -1,30705 | 1,273803 | -1,25824 | -1,17353 |
| **CYP27A1** | -1,10746 | 1,270726 | 1,230232 | 1,1481 | 1,093846 |
| **UGT2B28** | -1,11032 | 1,301837 | 1,217012 | 1,11536 | 1,110968 |
| **CYP3A7** | -1,13926 | 1,078369 | 1,084324 | 1,118546 | 1,085527 |
| **Ugt1a7c** | -1,08683 | 1,266793 | 1,096276 | 1,048447 | -1,34284 |
| **CYP1A2** | -1,20296 | 1,034577 | 1,082641 | 1,044196 | -1,04595 |
| **DBI** | 1,067842 | -1,29738 | 1,035173 | -1,26747 | 1,078351 |
| **CYP3A5** | -1,14579 | 1,256053 | 1,050053 | -1,0938 | -1,07644 |
| **HSD17B4** | -1,08485 | 1,037723 | 1,010027 | -1,10006 | -1,06344 |
| **YWHAG** | -1,37049 | 1,09901 | 1,001474 | -1,13431 | -1,09696 |
| **APOE** | 1,386757 | -1,56625 | -1,25116 | -1,35046 | 1,016212 |
| **DHRS4** | -1,16499 | 1,011104 | -1,02881 | -1,13912 | -1,14333 |
| **Rdh7** | -1,2674 | 1,024616 | -1,04587 | -1,13765 | -1,08005 |
| **POR** | -1,01605 | -1,24295 | -1,0314 | -1,00985 | -1,02347 |
| **ENO1** | -1,19739 | -1,01702 | -1,07996 | -1,05187 | -1,02516 |
| **SEC14L2** | -1,34132 | -1,07693 | -1,06188 | -1,08825 | -1,09722 |
| **GC** | -1,2427 | -1,0565 | -1,10506 | -1,12431 | -1,14606 |
| **HSPA8** | -1,34774 | -1,07792 | -1,12318 | -1,06946 | -1,07892 |
| **ADH5** | -1,16931 | -1,08124 | -1,18845 | -1,15667 | -1,17776 |
| **CES1** | -1,41916 | -1,02444 | -1,09682 | -1,17358 | -1,14952 |
| **ADH1C** | -1,1534 | -1,27919 | -1,11564 | -1,1902 | -1,1977 |
| **RHOA** | -1,245 | -1,14683 | -1,18287 | -1,26674 | -1,15313 |
| **YWHAH** | -1,36216 | -1,1568 | -1,13356 | -1,17143 | -1,20388 |
| **HSD11B1** | -1,4426 | -1,07828 | -1,14631 | -1,23588 | -1,16432 |
| **CBR1** | -1,59879 | -1,07398 | -1,239 | -1,26817 | -1,24626 |
| **AKR1D1** | -1,33146 | -1,65232 | -1,19748 | -1,15677 | -1,2221 |
| **APOA1** | -1,45626 | -1,32673 | -1,61761 | -1,71124 | -1,64511 |
| **Sult1a1** | -2,20512 | -2,22742 | -1,86171 | -1,57934 | -1,84231 |
| **CYP2B6** | -2,47496 | -2,25021 | -2,96652 | -2,69975 | -3,19004 |
| **RDH11** | -1210,96 | 28,2579 | 1,97793 | N/A | -6,19072 |

**Table S25:** Activation Z-score of proteins involved in the synthesis of lipids male vs. female. Activation Z-score was calculated with IPA software from Qiagen.

| **Pathway Summary** | -1,349 | -0,01 | -0,658 | -0,871 | -1,868 |
| --- | --- | --- | --- | --- | --- |
| **proteins in the synthesis of lipid network** | **0 h** | **24 h** | **48 h** | **72 h** | **96 h** |
| **CYP7B1** | 31,2713 | 6,058976 | 16,8092 | 10,00731 | 4,657266 |
| **G6PC** | 14,70826 | 1,324065 | 12,30242 | 7,972186 | 28,70709 |
| **PKLR** | 13,78798 | -1,80165 | 7,576125 | 3,425993 | 4,700936 |
| **PON1** | 5,379143 | -1,29146 | 4,020848 | 3,809965 | 3,577052 |
| **ME1** | 3,093078 | 2,647354 | 3,215682 | 3,393881 | 1,90172 |
| **FABP1** | 6,053154 | 1,046859 | 3,180378 | 2,118816 | 1,822998 |
| **HSD17B8** | 3,582373 | 1,246456 | 1,758038 | 1,226057 | 2,582128 |
| **XDH** | 2,626494 | 1,171979 | 2,326047 | 1,455116 | 2,697846 |
| **PRDX2** | 3,574061 | 1,031087 | 2,268723 | 1,663035 | 1,61625 |
| **GYS2** | 3,105212 | 1,864348 | 2,548107 | 1,101928 | 1,280565 |
| **FDPS** | 2,468791 | 1,059412 | 1,940406 | 1,667676 | 1,572961 |
| **CYP2C8** | 2,033738 | 1,140463 | 1,665818 | 1,322788 | 1,329823 |
| **EPHX2** | 1,644387 | 1,304006 | 1,508459 | 1,50264 | 1,467997 |
| **ABAT** | 2,389401 | -1,14232 | 2,132946 | 1,873112 | 1,786624 |
| **MTTP** | 1,713662 | 1,395471 | 1,490981 | 1,158708 | 1,260068 |
| **HSD17B11** | 18,77344 | -2,77045 | 4,807205 | -9,41318 | -4,44879 |
| **CYP2E1** | 1,447992 | 1,182643 | 1,328891 | 1,330951 | 1,274404 |
| **SCP2** | 1,343139 | 1,457684 | 1,376149 | 1,088662 | 1,079751 |
| **AMACR** | 1,120281 | 1,125937 | 1,297034 | 1,204147 | 1,106829 |
| **ACAT2** | 3,021496 | -1,11332 | 1,611311 | 1,027102 | 1,257862 |
| **AKR1C1/AKR1C2** | 1,443584 | -1,14832 | 1,328085 | 1,599962 | 2,486267 |
| **ALDH1A1** | 1,11525 | 1,153403 | 1,120552 | 1,118103 | 1,04725 |
| **Aldh1a7** | 1,094762 | -1,0265 | 1,155866 | 1,246114 | 1,305874 |
| **BAAT** | 6,162764 | -1,30705 | 1,273803 | -1,25824 | -1,17353 |
| **HSPA5** | -1,05944 | 1,282023 | 1,164393 | 1,173326 | 1,122079 |
| **CYP27A1** | -1,10746 | 1,270726 | 1,230232 | 1,1481 | 1,093846 |
| **ACSL1** | -1,02867 | 1,204015 | 1,192691 | 1,133303 | 1,053531 |
| **MTARC2** | 2,239569 | -1,12238 | 1,377651 | -1,02484 | 1,164483 |
| **ACSM1** | 1,442239 | -1,10595 | 1,31249 | -1,01903 | 1,154036 |
| **PDHB** | 1,484927 | -1,48361 | 1,056859 | 1,374551 | -1,01432 |
| **CYP1A2** | -1,20296 | 1,034577 | 1,082641 | 1,044196 | -1,04595 |
| **ACADVL** | -1,39433 | 1,074727 | 1,04507 | 1,100853 | -1,06107 |
| **DBI** | 1,067842 | -1,29738 | 1,035173 | -1,26747 | 1,078351 |
| **ACOX2** | 1,412405 | -1,03423 | 1,100172 | -1,01748 | -1,09563 |
| **HSD17B4** | -1,08485 | 1,037723 | 1,010027 | -1,10006 | -1,06344 |
| **ATP5PF** | -1,27354 | 1,206059 | 1,067826 | -1,11756 | -1,135 |
| **IL16** | -1,2305 | 1,100174 | 1,042755 | -1,1386 | -1,03362 |
| **COX7A2** | -1,30771 | 1,080881 | 1,005939 | -1,07396 | -1,07617 |
| **YWHAG** | -1,37049 | 1,09901 | 1,001474 | -1,13431 | -1,09696 |
| **APOE** | 1,386757 | -1,56625 | -1,25116 | -1,35046 | 1,016212 |
| **ABCD3** | -1,27354 | 1,073769 | -1,0325 | -1,05477 | -1,14028 |
| **PHB** | -1,25731 | -1,01503 | 1,01242 | -1,07633 | -1,09358 |
| **Rdh7** | -1,2674 | 1,024616 | -1,04587 | -1,13765 | -1,08005 |
| **ACSL5** | -1,36781 | 1,015752 | -1,02408 | -1,05963 | -1,08129 |
| **SLC27A2** | -1,39595 | 1,018598 | -1,03483 | -1,05286 | -1,0929 |
| **CLU** | -1,56022 | -1,08343 | -1,13591 | -1,08624 | 1,000076 |
| **PGD** | -1,74239 | -2,17847 | 1,040307 | -1,21359 | -1,17486 |
| **POR** | -1,01605 | -1,24295 | -1,0314 | -1,00985 | -1,02347 |
| **BHMT** | -1,26236 | -1,05999 | -1,00957 | -1,01573 | -1,11119 |
| **IDH1** | -1,31362 | -1,06028 | -1,0369 | -1,01619 | -1,08128 |
| **DPP4** | -1,20859 | -1,0021 | -1,04133 | -1,15811 | -1,13298 |
| **SEC14L2** | -1,34132 | -1,07693 | -1,06188 | -1,08825 | -1,09722 |
| **HSPA8** | -1,34774 | -1,07792 | -1,12318 | -1,06946 | -1,07892 |
| **ACAT1** | -1,37874 | -1,17914 | -1,05347 | -1,05319 | -1,1087 |
| **ADH5** | -1,16931 | -1,08124 | -1,18845 | -1,15667 | -1,17776 |
| **CES1** | -1,41916 | -1,02444 | -1,09682 | -1,17358 | -1,14952 |
| **PITPNM1** | -1,38031 | -1,34043 | -1,1198 | -1,00603 | -1,0364 |
| **ADH1C** | -1,1534 | -1,27919 | -1,11564 | -1,1902 | -1,1977 |
| **RHOA** | -1,245 | -1,14683 | -1,18287 | -1,26674 | -1,15313 |
| **CYB5A** | -1,49225 | -1,01019 | -1,13297 | -1,2299 | -1,17084 |
| **HSD17B13** | -1,38576 | -1,0408 | -1,17418 | -1,23351 | -1,2044 |
| **HSD11B1** | -1,4426 | -1,07828 | -1,14631 | -1,23588 | -1,16432 |
| **ACADL** | -1,62185 | -1,24181 | -1,1833 | -1,21001 | -1,27713 |
| **AKR1D1** | -1,33146 | -1,65232 | -1,19748 | -1,15677 | -1,2221 |
| **ALDH3A2** | -1,20044 | -1,74082 | -1,15959 | -2,14406 | -1,12836 |
| **FABP5** | -1,80466 | -1,68977 | -1,47194 | -1,26345 | -1,36353 |
| **RGN** | -1,76745 | -1,68733 | -1,45828 | -1,35929 | -1,3299 |
| **ALB** | -1,41802 | -1,6364 | -1,62211 | -1,50474 | -1,53956 |
| **APOA1** | -1,45626 | -1,32673 | -1,61761 | -1,71124 | -1,64511 |
| **FMO3** | -2,73276 | -1,57537 | -1,37281 | -1,73574 | -1,54146 |

**Table S26:** Activation Z-score of proteins involved in the transport of lipids male vs. female. Activation Z-score was calculated with IPA software from Qiagen.

| **Pathway Summary** | -0,821 | -1,631 | -1,513 | N/A | -1,054 |
| --- | --- | --- | --- | --- | --- |
| **proteins in the transport of lipid network** | **0 h** | **24 h** | **48 h** | **72 h** | **96 h** |
| **SLCO1B3** | 9,933269 | 1,519721 | 14,51829 | 12,2419 | 16,8349 |
| **SLC25A10** | N/A | 1,58732 | N/A | 2,958949 | 27,66288 |
| **PON1** | 5,379143 | -1,29146 | 4,020848 | 3,809965 | 3,577052 |
| **FABP1** | 6,053154 | 1,046859 | 3,180378 | 2,118816 | 1,822998 |
| **MTTP** | 1,713662 | 1,395471 | 1,490981 | 1,158708 | 1,260068 |
| **SCP2** | 1,343139 | 1,457684 | 1,376149 | 1,088662 | 1,079751 |
| **EPHX1** | 1,087181 | 1,266837 | 1,190451 | 1,232295 | 1,024527 |
| **AKR1C1/AKR1C2** | 1,443584 | -1,14832 | 1,328085 | 1,599962 | 2,486267 |
| **ACSL1** | -1,02867 | 1,204015 | 1,192691 | 1,133303 | 1,053531 |
| **CPT2** | 1,00547 | -1,05575 | -1,04768 | -1,11747 | 1,097332 |
| **APOE** | 1,386757 | -1,56625 | -1,25116 | -1,35046 | 1,016212 |
| **ANXA6** | -1,24158 | 1,054998 | -1,00375 | -1,00711 | -1,01955 |
| **CANX** | -1,24802 | -1,04434 | -1,07101 | 1,029669 | -1,04539 |
| **ABCD3** | -1,27354 | 1,073769 | -1,0325 | -1,05477 | -1,14028 |
| **ACSL5** | -1,36781 | 1,015752 | -1,02408 | -1,05963 | -1,08129 |
| **SLC27A2** | -1,39595 | 1,018598 | -1,03483 | -1,05286 | -1,0929 |
| **CLU** | -1,56022 | -1,08343 | -1,13591 | -1,08624 | 1,000076 |
| **GC** | -1,2427 | -1,0565 | -1,10506 | -1,12431 | -1,14606 |
| **HSPA8** | -1,34774 | -1,07792 | -1,12318 | -1,06946 | -1,07892 |
| **PRDX6** | -1,27106 | -1,14811 | -1,05908 | -1,15106 | -1,13163 |
| **ACAT1** | -1,37874 | -1,17914 | -1,05347 | -1,05319 | -1,1087 |
| **CES1** | -1,41916 | -1,02444 | -1,09682 | -1,17358 | -1,14952 |
| **PITPNM1** | -1,38031 | -1,34043 | -1,1198 | -1,00603 | -1,0364 |
| **RHOA** | -1,245 | -1,14683 | -1,18287 | -1,26674 | -1,15313 |
| **FABP5** | -1,80466 | -1,68977 | -1,47194 | -1,26345 | -1,36353 |
| **ALB** | -1,41802 | -1,6364 | -1,62211 | -1,50474 | -1,53956 |
| **APOA1** | -1,45626 | -1,32673 | -1,61761 | -1,71124 | -1,64511 |

**Table S27:** Activation Z-score of proteins involved in the oxidation of lipids male vs. female. Activation Z-score was calculated with IPA software from Qiagen.

| **Pathway Summary** | 0,13 | 1,137 | 0,933 | 0,055 | 0,794 |
| --- | --- | --- | --- | --- | --- |
| **proteins in the oxidation of lipid network** | **0 h** | **24 h** | **48 h** | **72 h** | **96 h** |
| **HSD17B10** | 7,388114 | 1,360734 | 4,519519 | 1,661595 | 1,922858 |
| **PON1** | 5,379143 | -1,29146 | 4,020848 | 3,809965 | 3,577052 |
| **FABP1** | 6,053154 | 1,046859 | 3,180378 | 2,118816 | 1,822998 |
| **CYCS** | 2,500362 | 1,404663 | 1,656574 | 1,142855 | 1,291703 |
| **CYP2C8** | 2,033738 | 1,140463 | 1,665818 | 1,322788 | 1,329823 |
| **MTTP** | 1,713662 | 1,395471 | 1,490981 | 1,158708 | 1,260068 |
| **CYP2E1** | 1,447992 | 1,182643 | 1,328891 | 1,330951 | 1,274404 |
| **SCP2** | 1,343139 | 1,457684 | 1,376149 | 1,088662 | 1,079751 |
| **ACAA1** | 1,404985 | 1,025449 | 1,266997 | 1,195626 | 1,083982 |
| **AKR1C1/AKR1C2** | 1,443584 | -1,14832 | 1,328085 | 1,599962 | 2,486267 |
| **ALDH1A1** | 1,11525 | 1,153403 | 1,120552 | 1,118103 | 1,04725 |
| **IMMT** | 1,045195 | 1,160379 | 1,137923 | 1,138391 | 1,048817 |
| **PHYH** | 1,58874 | 1,139995 | 1,099729 | -1,08817 | 1,123978 |
| **Aldh1a7** | 1,094762 | -1,0265 | 1,155866 | 1,246114 | 1,305874 |
| **CYP27A1** | -1,10746 | 1,270726 | 1,230232 | 1,1481 | 1,093846 |
| **ACSL1** | -1,02867 | 1,204015 | 1,192691 | 1,133303 | 1,053531 |
| **ACADS** | -1,16066 | 1,043016 | 1,095458 | 1,013216 | -1,00239 |
| **HADHB** | -1,20335 | 1,110141 | 1,029023 | -1,03476 | 1,016435 |
| **CYP1A2** | -1,20296 | 1,034577 | 1,082641 | 1,044196 | -1,04595 |
| **ACADVL** | -1,39433 | 1,074727 | 1,04507 | 1,100853 | -1,06107 |
| **ACOX2** | 1,412405 | -1,03423 | 1,100172 | -1,01748 | -1,09563 |
| **ECI1** | -1,09015 | 1,062955 | 1,102756 | -1,00703 | -1,01257 |
| **CYP3A5** | -1,14579 | 1,256053 | 1,050053 | -1,0938 | -1,07644 |
| **CPT2** | 1,00547 | -1,05575 | -1,04768 | -1,11747 | 1,097332 |
| **HSD17B4** | -1,08485 | 1,037723 | 1,010027 | -1,10006 | -1,06344 |
| **HADHA** | -1,32721 | 1,156054 | 1,02004 | -1,03649 | -1,18022 |
| **APOE** | 1,386757 | -1,56625 | -1,25116 | -1,35046 | 1,016212 |
| **ACAA2** | -1,22944 | -1,1139 | 1,043495 | -1,04608 | -1,05174 |
| **ABCD3** | -1,27354 | 1,073769 | -1,0325 | -1,05477 | -1,14028 |
| **ACSL5** | -1,36781 | 1,015752 | -1,02408 | -1,05963 | -1,08129 |
| **SLC27A2** | -1,39595 | 1,018598 | -1,03483 | -1,05286 | -1,0929 |
| **POR** | -1,01605 | -1,24295 | -1,0314 | -1,00985 | -1,02347 |
| **ECHS1** | -1,3722 | -1,02745 | -1,03117 | -1,16217 | -1,12699 |
| **ADH5** | -1,16931 | -1,08124 | -1,18845 | -1,15667 | -1,17776 |
| **HADH** | -1,16544 | -1,29837 | -1,10253 | -1,28832 | -1,07399 |
| **ADH1C** | -1,1534 | -1,27919 | -1,11564 | -1,1902 | -1,1977 |
| **CISD1** | -1,53586 | -1,0861 | -1,14281 | -1,11638 | -1,14632 |
| **HSD11B1** | -1,4426 | -1,07828 | -1,14631 | -1,23588 | -1,16432 |
| **PECR** | -1,54701 | -1,12179 | -1,12935 | -1,15299 | -1,24297 |
| **ACADL** | -1,62185 | -1,24181 | -1,1833 | -1,21001 | -1,27713 |
| **HACL1** | -1,32022 | -1,54462 | -1,27234 | -1,28269 | -1,18184 |
| **ALDH3A2** | -1,20044 | -1,74082 | -1,15959 | -2,14406 | -1,12836 |
| **APOA1** | -1,45626 | -1,32673 | -1,61761 | -1,71124 | -1,64511 |
| **CYP2B6** | -2,47496 | -2,25021 | -2,96652 | -2,69975 | -3,19004 |

**Table S28:** Activation Z-score of proteins involved cholesterol transport male vs. female. Activation Z-score was calculated with IPA software from Qiagen.

| **Pathway Summary** | -1,317 | -1,914 | -1,914 | N/A | -1,678 |
| --- | --- | --- | --- | --- | --- |
| **proteom in the cholesterol transport network** | **0 h** | **24 h** | **48 h** | **72 h** | **96 h** |
| **PON1** | 5,379143 | -1,29146 | 4,020848 | 3,809965 | 3,577052 |
| **FABP1** | 6,053154 | 1,046859 | 3,180378 | 2,118816 | 1,822998 |
| **MTTP** | 1,713662 | 1,395471 | 1,490981 | 1,158708 | 1,260068 |
| **SCP2** | 1,343139 | 1,457684 | 1,376149 | 1,088662 | 1,079751 |
| **ACSL1** | -1,02867 | 1,204015 | 1,192691 | 1,133303 | 1,053531 |
| **APOE** | 1,386757 | -1,56625 | -1,25116 | -1,35046 | 1,016212 |
| **ANXA6** | -1,24158 | 1,054998 | -1,00375 | -1,00711 | -1,01955 |
| **CANX** | -1,24802 | -1,04434 | -1,07101 | 1,029669 | -1,04539 |
| **CLU** | -1,56022 | -1,08343 | -1,13591 | -1,08624 | 1,000076 |
| **ACAT1** | -1,37874 | -1,17914 | -1,05347 | -1,05319 | -1,1087 |
| **CES1** | -1,41916 | -1,02444 | -1,09682 | -1,17358 | -1,14952 |
| **RHOA** | -1,245 | -1,14683 | -1,18287 | -1,26674 | -1,15313 |
| **ALB** | -1,41802 | -1,6364 | -1,62211 | -1,50474 | -1,53956 |
| **APOA1** | -1,45626 | -1,32673 | -1,61761 | -1,71124 | -1,64511 |

**Table S29:** Activation Z-score of proteins involved the transport of steroids male vs. female. Activation Z-score was calculated with IPA software from Qiagen.

| **Pathway Summary** | -1,205 | -1,499 | -1,499 | N/A | -1,919 |
| --- | --- | --- | --- | --- | --- |
| **proteins in the Transport of steroid network** | **0 h** | **24 h** | **48 h** | **72 h** | **96 h** |
| **SLCO1B3** | 9,933269 | 1,519721 | 14,51829 | 12,2419 | 16,8349 |
| **PON1** | 5,379143 | -1,29146 | 4,020848 | 3,809965 | 3,577052 |
| **FABP1** | 6,053154 | 1,046859 | 3,180378 | 2,118816 | 1,822998 |
| **MTTP** | 1,713662 | 1,395471 | 1,490981 | 1,158708 | 1,260068 |
| **SCP2** | 1,343139 | 1,457684 | 1,376149 | 1,088662 | 1,079751 |
| **EPHX1** | 1,087181 | 1,266837 | 1,190451 | 1,232295 | 1,024527 |
| **AKR1C1/AKR1C2** | 1,443584 | -1,14832 | 1,328085 | 1,599962 | 2,486267 |
| **ACSL1** | -1,02867 | 1,204015 | 1,192691 | 1,133303 | 1,053531 |
| **APOE** | 1,386757 | -1,56625 | -1,25116 | -1,35046 | 1,016212 |
| **ANXA6** | -1,24158 | 1,054998 | -1,00375 | -1,00711 | -1,01955 |
| **CANX** | -1,24802 | -1,04434 | -1,07101 | 1,029669 | -1,04539 |
| **CLU** | -1,56022 | -1,08343 | -1,13591 | -1,08624 | 1,000076 |
| **GC** | -1,2427 | -1,0565 | -1,10506 | -1,12431 | -1,14606 |
| **HSPA8** | -1,34774 | -1,07792 | -1,12318 | -1,06946 | -1,07892 |
| **ACAT1** | -1,37874 | -1,17914 | -1,05347 | -1,05319 | -1,1087 |
| **CES1** | -1,41916 | -1,02444 | -1,09682 | -1,17358 | -1,14952 |
| **RHOA** | -1,245 | -1,14683 | -1,18287 | -1,26674 | -1,15313 |
| **ALB** | -1,41802 | -1,6364 | -1,62211 | -1,50474 | -1,53956 |
| **APOA1** | -1,45626 | -1,32673 | -1,61761 | -1,71124 | -1,64511 |

**Table S30:** Activation Z-score of proteins involved in the concentration of fatty acids male vs. female. Activation Z-score was calculated with IPA software from Qiagen.

| **Pathway Summary** | 0,462 | -0,626 | -2,189 | 0,692 | -1,169 |
| --- | --- | --- | --- | --- | --- |
| **proteins in the concentration of fatty acid network** | **0 h** | **24 h** | **48 h** | **72 h** | **96 h** |
| ***PCK1*** | 24,1331 | 1,840876 | 139,9636 | 3,253179 | 33,55668 |
| ***G6PC*** | 14,70826 | 1,324065 | 12,30242 | 7,972186 | 28,70709 |
| ***PON1*** | 5,379143 | -1,29146 | 4,020848 | 3,809965 | 3,577052 |
| ***FABP1*** | 6,053154 | 1,046859 | 3,180378 | 2,118816 | 1,822998 |
| ***XDH*** | 2,626494 | 1,171979 | 2,326047 | 1,455116 | 2,697846 |
| ***EPHX2*** | 1,644387 | 1,304006 | 1,508459 | 1,50264 | 1,467997 |
| ***AIFM1*** | 1,618712 | 1,35612 | 1,546096 | 1,548521 | 1,124865 |
| ***MTTP*** | 1,713662 | 1,395471 | 1,490981 | 1,158708 | 1,260068 |
| ***GPD2*** | 1,506992 | 1,278125 | 1,629196 | 1,355077 | 1,22815 |
| ***CYP2E1*** | 1,447992 | 1,182643 | 1,328891 | 1,330951 | 1,274404 |
| ***SCP2*** | 1,343139 | 1,457684 | 1,376149 | 1,088662 | 1,079751 |
| ***AMACR*** | 1,120281 | 1,125937 | 1,297034 | 1,204147 | 1,106829 |
| ***PHYH*** | 1,58874 | 1,139995 | 1,099729 | -1,08817 | 1,123978 |
| ***HSPA5*** | -1,05944 | 1,282023 | 1,164393 | 1,173326 | 1,122079 |
| ***CYP27A1*** | -1,10746 | 1,270726 | 1,230232 | 1,1481 | 1,093846 |
| ***ACSL1*** | -1,02867 | 1,204015 | 1,192691 | 1,133303 | 1,053531 |
| ***HSD17B12*** | -1,1006 | 1,138275 | 1,108012 | 1,007579 | 1,059565 |
| ***CES1G*** | 1,294614 | -1,03109 | 1,138688 | -1,00788 | 1,024478 |
| ***CBS/CBSL*** | 1,08434 | -1,06933 | 1,014248 | -1,21461 | 1,455808 |
| ***CYP1A2*** | -1,20296 | 1,034577 | 1,082641 | 1,044196 | -1,04595 |
| ***DBI*** | 1,067842 | -1,29738 | 1,035173 | -1,26747 | 1,078351 |
| ***ECI1*** | -1,09015 | 1,062955 | 1,102756 | -1,00703 | -1,01257 |
| ***LOC102724788/PRODH*** | -1,12861 | -1,04496 | 1,136844 | 1,106199 | -1,05634 |
| ***HSD17B4*** | -1,08485 | 1,037723 | 1,010027 | -1,10006 | -1,06344 |
| ***HADHA*** | -1,32721 | 1,156054 | 1,02004 | -1,03649 | -1,18022 |
| ***APOE*** | 1,386757 | -1,56625 | -1,25116 | -1,35046 | 1,016212 |
| ***GPX1*** | -1,40426 | -1,20379 | -1,05074 | 1,014278 | -1,14257 |
| ***IDH1*** | -1,31362 | -1,06028 | -1,0369 | -1,01619 | -1,08128 |
| ***PRDX3*** | -1,23476 | -1,05035 | -1,03464 | -1,09787 | -1,14283 |
| ***PRDX6*** | -1,27106 | -1,14811 | -1,05908 | -1,15106 | -1,13163 |
| ***CES1*** | -1,41916 | -1,02444 | -1,09682 | -1,17358 | -1,14952 |
| ***HADH*** | -1,16544 | -1,29837 | -1,10253 | -1,28832 | -1,07399 |
| ***HSD11B1*** | -1,4426 | -1,07828 | -1,14631 | -1,23588 | -1,16432 |
| ***ACADL*** | -1,62185 | -1,24181 | -1,1833 | -1,21001 | -1,27713 |
| ***FABP5*** | -1,80466 | -1,68977 | -1,47194 | -1,26345 | -1,36353 |
| ***FMO3*** | -2,73276 | -1,57537 | -1,37281 | -1,73574 | -1,54146 |

**Table S31:** Activation Z-score of genes involved in the concentration of lipids is male vs. female. Activation Z-score was calculated with IPA software from Qiagen.

| **Pathway Summary** | -0,955 | -0,698 | -0,339 | 1,355 | -2,226 |
| --- | --- | --- | --- | --- | --- |
| **proteins in the concentration of lipid network** | **0 h** | **24 h** | **48 h** | **72 h** | **96 h** |
| ***PCK1*** | 24,1331 | 1,840876 | 139,9636 | 3,253179 | 33,55668 |
| ***STOML2*** | 29,7317 | 1,377423 | 23,65034 | 6,418508 | 10,02561 |
| ***G6PC*** | 14,70826 | 1,324065 | 12,30242 | 7,972186 | 28,70709 |
| ***SLCO1B3*** | 9,933269 | 1,519721 | 14,51829 | 12,2419 | 16,8349 |
| ***PON1*** | 5,379143 | -1,29146 | 4,020848 | 3,809965 | 3,577052 |
| ***FABP1*** | 6,053154 | 1,046859 | 3,180378 | 2,118816 | 1,822998 |
| ***XDH*** | 2,626494 | 1,171979 | 2,326047 | 1,455116 | 2,697846 |
| ***COMT*** | 1,969446 | 1,441108 | 1,502034 | 1,457034 | 1,348906 |
| ***EPHX2*** | 1,644387 | 1,304006 | 1,508459 | 1,50264 | 1,467997 |
| ***AIFM1*** | 1,618712 | 1,35612 | 1,546096 | 1,548521 | 1,124865 |
| ***MTTP*** | 1,713662 | 1,395471 | 1,490981 | 1,158708 | 1,260068 |
| ***GPD2*** | 1,506992 | 1,278125 | 1,629196 | 1,355077 | 1,22815 |
| ***CYP2E1*** | 1,447992 | 1,182643 | 1,328891 | 1,330951 | 1,274404 |
| ***VAMP3*** | 1,374255 | 1,559269 | 1,156344 | 1,130481 | 1,18151 |
| ***SCP2*** | 1,343139 | 1,457684 | 1,376149 | 1,088662 | 1,079751 |
| ***AMACR*** | 1,120281 | 1,125937 | 1,297034 | 1,204147 | 1,106829 |
| ***ACAT2*** | 3,021496 | -1,11332 | 1,611311 | 1,027102 | 1,257862 |
| ***ALDH1A1*** | 1,11525 | 1,153403 | 1,120552 | 1,118103 | 1,04725 |
| ***PHYH*** | 1,58874 | 1,139995 | 1,099729 | -1,08817 | 1,123978 |
| ***HSPA5*** | -1,05944 | 1,282023 | 1,164393 | 1,173326 | 1,122079 |
| ***CYP27A1*** | -1,10746 | 1,270726 | 1,230232 | 1,1481 | 1,093846 |
| ***ACSL1*** | -1,02867 | 1,204015 | 1,192691 | 1,133303 | 1,053531 |
| ***CYP3A7*** | -1,13926 | 1,078369 | 1,084324 | 1,118546 | 1,085527 |
| ***HSD17B12*** | -1,1006 | 1,138275 | 1,108012 | 1,007579 | 1,059565 |
| ***CES1G*** | 1,294614 | -1,03109 | 1,138688 | -1,00788 | 1,024478 |
| ***CBS/CBSL*** | 1,08434 | -1,06933 | 1,014248 | -1,21461 | 1,455808 |
| ***CYP1A2*** | -1,20296 | 1,034577 | 1,082641 | 1,044196 | -1,04595 |
| ***DBI*** | 1,067842 | -1,29738 | 1,035173 | -1,26747 | 1,078351 |
| ***SOD2*** | 1,197649 | -1,0282 | 1,130983 | -1,04954 | -1,03783 |
| ***ECI1*** | -1,09015 | 1,062955 | 1,102756 | -1,00703 | -1,01257 |
| ***LOC102724788/PRODH*** | -1,12861 | -1,04496 | 1,136844 | 1,106199 | -1,05634 |
| ***CYP3A5*** | -1,14579 | 1,256053 | 1,050053 | -1,0938 | -1,07644 |
| ***GULO*** | -1,14722 | 1,107664 | 1,011663 | -1,05884 | -1,05326 |
| ***HSD17B4*** | -1,08485 | 1,037723 | 1,010027 | -1,10006 | -1,06344 |
| ***HADHA*** | -1,32721 | 1,156054 | 1,02004 | -1,03649 | -1,18022 |
| ***APOE*** | 1,386757 | -1,56625 | -1,25116 | -1,35046 | 1,016212 |
| ***ANXA6*** | -1,24158 | 1,054998 | -1,00375 | -1,00711 | -1,01955 |
| ***VDAC1*** | -1,22472 | 1,02935 | -1,00297 | -1,08593 | -1,07471 |
| ***H6PD*** | -1,36862 | -1,17612 | -1,06536 | 1,026087 | -1,17327 |
| ***GPX1*** | -1,40426 | -1,20379 | -1,05074 | 1,014278 | -1,14257 |
| ***CLU*** | -1,56022 | -1,08343 | -1,13591 | -1,08624 | 1,000076 |
| ***VDAC2*** | -1,69312 | 1,01662 | -1,1055 | -1,10678 | -1,32373 |
| ***POR*** | -1,01605 | -1,24295 | -1,0314 | -1,00985 | -1,02347 |
| ***BHMT*** | -1,26236 | -1,05999 | -1,00957 | -1,01573 | -1,11119 |
| ***IDH1*** | -1,31362 | -1,06028 | -1,0369 | -1,01619 | -1,08128 |
| ***PRDX3*** | -1,23476 | -1,05035 | -1,03464 | -1,09787 | -1,14283 |
| ***SEC14L2*** | -1,34132 | -1,07693 | -1,06188 | -1,08825 | -1,09722 |
| ***GC*** | -1,2427 | -1,0565 | -1,10506 | -1,12431 | -1,14606 |
| ***PRDX6*** | -1,27106 | -1,14811 | -1,05908 | -1,15106 | -1,13163 |
| ***ACAT1*** | -1,37874 | -1,17914 | -1,05347 | -1,05319 | -1,1087 |
| ***CTSD*** | -1,32006 | -1,09217 | -1,10985 | -1,12364 | -1,14063 |
| ***CES1*** | -1,41916 | -1,02444 | -1,09682 | -1,17358 | -1,14952 |
| ***HADH*** | -1,16544 | -1,29837 | -1,10253 | -1,28832 | -1,07399 |
| ***ADH1C*** | -1,1534 | -1,27919 | -1,11564 | -1,1902 | -1,1977 |
| ***ARHGDIA*** | -1,33882 | -1,14896 | -1,24658 | -1,02643 | -1,23221 |
| ***RHOA*** | -1,245 | -1,14683 | -1,18287 | -1,26674 | -1,15313 |
| ***CISD1*** | -1,53586 | -1,0861 | -1,14281 | -1,11638 | -1,14632 |
| ***HSD11B1*** | -1,4426 | -1,07828 | -1,14631 | -1,23588 | -1,16432 |
| ***LMNA*** | -1,16568 | -1,29152 | -1,26055 | -1,23843 | -1,13491 |
| ***GSTK1*** | -1,52816 | -1,30558 | -1,10458 | -1,0842 | -1,26309 |
| ***ACADL*** | -1,62185 | -1,24181 | -1,1833 | -1,21001 | -1,27713 |
| ***FABP5*** | -1,80466 | -1,68977 | -1,47194 | -1,26345 | -1,36353 |
| ***RGN*** | -1,76745 | -1,68733 | -1,45828 | -1,35929 | -1,3299 |
| ***ALB*** | -1,41802 | -1,6364 | -1,62211 | -1,50474 | -1,53956 |
| ***APOA1*** | -1,45626 | -1,32673 | -1,61761 | -1,71124 | -1,64511 |
| ***FMO3*** | -2,73276 | -1,57537 | -1,37281 | -1,73574 | -1,54146 |

**Table S32:** Activation Z-score of proteins involved hepatic steatosis male vs. female. Activation Z-score was calculated with IPA software from Qiagen.

| **Pathway Summary** | -0,176 | 2,159 | 0,842 | 2,2 | 0,14 |
| --- | --- | --- | --- | --- | --- |
| **proteins in the hepatic steatosis network** | **0 h** | **24 h** | **48 h** | **72 h** | **96 h** |
| **PCK1** | 24,1331 | 1,840876 | 139,9636 | 3,253179 | 33,55668 |
| **G6PC** | 14,70826 | 1,324065 | 12,30242 | 7,972186 | 28,70709 |
| **GSTP1** | 6,86094 | 6,433403 | 4,963562 | 3,582487 | 2,55001 |
| **MTTP** | 1,713662 | 1,395471 | 1,490981 | 1,158708 | 1,260068 |
| **GPD2** | 1,506992 | 1,278125 | 1,629196 | 1,355077 | 1,22815 |
| **CYP2E1** | 1,447992 | 1,182643 | 1,328891 | 1,330951 | 1,274404 |
| **DDC** | 1,394414 | 1,244992 | 1,269643 | 1,183337 | 1,141864 |
| **PHYH** | 1,58874 | 1,139995 | 1,099729 | -1,08817 | 1,123978 |
| **ACSL1** | -1,02867 | 1,204015 | 1,192691 | 1,133303 | 1,053531 |
| **GNMT** | 1,38478 | -1,29053 | 1,182726 | 1,080915 | 1,163164 |
| **GPT** | 2,345906 | 1,002049 | 1,165189 | -1,0813 | -1,13605 |
| **Ces1g** | 1,294614 | -1,03109 | 1,138688 | -1,00788 | 1,024478 |
| **PDHB** | 1,484927 | -1,48361 | 1,056859 | 1,374551 | -1,01432 |
| **CBS/CBSL** | 1,08434 | -1,06933 | 1,014248 | -1,21461 | 1,455808 |
| **ACADS** | -1,16066 | 1,043016 | 1,095458 | 1,013216 | -1,00239 |
| **ACADVL** | -1,39433 | 1,074727 | 1,04507 | 1,100853 | -1,06107 |
| **ACOX2** | 1,412405 | -1,03423 | 1,100172 | -1,01748 | -1,09563 |
| **SOD2** | 1,197649 | -1,0282 | 1,130983 | -1,04954 | -1,03783 |
| **CPT2** | 1,00547 | -1,05575 | -1,04768 | -1,11747 | 1,097332 |
| **HSD17B4** | -1,08485 | 1,037723 | 1,010027 | -1,10006 | -1,06344 |
| **HADHA** | -1,32721 | 1,156054 | 1,02004 | -1,03649 | -1,18022 |
| **APOE** | 1,386757 | -1,56625 | -1,25116 | -1,35046 | 1,016212 |
| **KRT8** | 1,252915 | -1,11096 | -1,12863 | -1,07791 | -1,03113 |
| **ACAA2** | -1,22944 | -1,1139 | 1,043495 | -1,04608 | -1,05174 |
| **PC** | -1,25304 | -1,07147 | 1,007253 | -1,12885 | -1,07638 |
| **CLU** | -1,56022 | -1,08343 | -1,13591 | -1,08624 | 1,000076 |
| **POR** | -1,01605 | -1,24295 | -1,0314 | -1,00985 | -1,02347 |
| **BHMT** | -1,26236 | -1,05999 | -1,00957 | -1,01573 | -1,11119 |
| **DPP4** | -1,20859 | -1,0021 | -1,04133 | -1,15811 | -1,13298 |
| **PCCA** | -1,28063 | -1,03505 | -1,01684 | -1,10886 | -1,11309 |
| **HSD17B13** | -1,38576 | -1,0408 | -1,17418 | -1,23351 | -1,2044 |
| **HSD11B1** | -1,4426 | -1,07828 | -1,14631 | -1,23588 | -1,16432 |
| **LMNA** | -1,16568 | -1,29152 | -1,26055 | -1,23843 | -1,13491 |
| **ACADL** | -1,62185 | -1,24181 | -1,1833 | -1,21001 | -1,27713 |
| **MAT1A** | -2,2044 | -1,49102 | -1,30664 | -1,26116 | -1,25024 |
| **FABP5** | -1,80466 | -1,68977 | -1,47194 | -1,26345 | -1,36353 |
| **RGN** | -1,76745 | -1,68733 | -1,45828 | -1,35929 | -1,3299 |

**Table S33:** Activation Z-score of genes involved in bile acid biosynthesis male vs. female. Activation Z-score was calculated with IPA software from Qiagen.

| **Pathway Summary** | N/A | 2,828 | 0,707 | -1,89 | -2,646 |
| --- | --- | --- | --- | --- | --- |
| **genes in the bile acid biosynthesis, neutral pathway network** | **0 h** | **24 h** | **48 h** | **72 h** | **96 h** |
| ***Scp2*** | 0,905224 | 2,031537 | 0,985545 | 0,350656 | 0,253287 |
| ***Cyp8b1*** | 0,538241 | 1,700563 | 1,224542 | -0,39323 | -1,03816 |
| ***Cyp27a1*** | 0,099837 | 0,68324 | 0,612828 | 0,138729 | -0,20747 |
| ***Hsd3b7*** | 0,077291 | 0,404465 | 0,271247 | -0,00801 | -0,07627 |
| ***Akr1c4*** | 0,140055 | 2,312705 | 0,392458 | -1,04147 | -1,21843 |
| ***Slc27a5*** | -0,19069 | 0,503285 | 0,492936 | -0,04094 | -0,59322 |
| ***Akr1c1/akr1c2*** | 0,211396 | 0,14172 | -0,08444 | -0,23582 | -0,10301 |
| ***Amacr*** | 0,266114 | 0,465913 | -0,78026 | -0,35673 | -0,34628 |
| ***Baat*** | -0,02758 | -0,14993 | -0,88211 | -0,74723 | -0,82395 |
| ***Cyp7a1*** | -0,10229 | 1,351961 | -0,28199 | -1,67746 | -2,37028 |
| ***Akr1d1*** | -0,74179 | 0,643042 | -0,61832 | -1,92256 | -2,62267 |
| ***Akr1c3*** | -0,27282 | -0,12655 | -2,54033 | -1,89342 | -0,62448 |

**Table S34:** Activation Z-score of genes involved in the superpathway of inositol phosphate compounds male vs. female. Activation Z-score was calculated with IPA software from Qiagen.

| **Pathway Summary** | 0,522 | -2,393 | 0,6 | -0,392 | 0,229 |
| --- | --- | --- | --- | --- | --- |
| **genes in the superpathway of inositol phosphate compounds network** | **0 h** | **24 h** | **48 h** | **72 h** | **96 h** |
| ***Ca3*** | 0.401537 | 2.274623 | 2.373561 | -0.43058 | -0.90828 |
| ***Dusp5*** | -0.49416 | 0.162825 | 0.724155 | 0.872127 | 0.830301 |
| ***Ptprn*** | 0.319173 | 0.08963 | 0.596419 | 0.45006 | 0.459358 |
| ***Dusp10*** | 0.156704 | 0.533341 | 0.228137 | 0.295862 | 0.659126 |
| ***Socs3*** | 0.648692 | 0.44729 | 0.310238 | 0.230759 | 0.070863 |
| ***Ppp2r5b*** | 0.54375 | 0.20016 | 0.209439 | 0.266752 | 0.210274 |
| ***Ppp2r3d*** | 0.086701 | 0.406858 | 0.228016 | 0.253038 | 0.36096 |
| ***Itpkc*** | -0.0009 | 0.510679 | 0.271302 | 0.298879 | 0.234149 |
| ***Ippk*** | 0.340871 | 0.161164 | 0.404669 | 0.194276 | 0.126026 |
| ***Itpka*** | 0.056002 | 0.111233 | 0.385123 | 0.304081 | 0.351446 |
| ***Ptpn23*** | 0.057579 | 0.335796 | 0.238826 | 0.43258 | 0.139723 |
| ***Ppp1r13b*** | 0.254983 | 0.316981 | 0.120109 | 0.260822 | 0.207424 |
| ***Ppm1h*** | 0.053416 | 0.304404 | 0.232689 | 0.288531 | 0.209728 |
| ***Itpkb*** | 0.035169 | 0.161499 | 0.305119 | 0.338989 | 0.23957 |
| ***Dusp27*** | 0.014179 | 0.489749 | 0.1135 | 0.250319 | 0.201684 |
| ***Ptpn2*** | 0.040989 | 0.658477 | 0.237455 | 0.140474 | -0.00844 |
| ***Ephx2*** | 0.424815 | 0.609694 | -0.06428 | 0.03513 | 0.023852 |
| ***Pip5k1b*** | -0.1052 | 0.344318 | 0.046992 | 0.21469 | 0.522942 |
| ***Ppfia1*** | 0.563183 | -0.00267 | 0.136289 | 0.263459 | -0.00644 |
| ***Ppp1r14a*** | 0.169126 | 0.486704 | 0.296002 | -0.20894 | 0.158245 |
| ***Plcb2*** | 0.164871 | 0.199211 | 0.118978 | 0.264859 | 0.1516 |
| ***Dusp8*** | 0.041582 | 0.360798 | 0.155462 | 0.252489 | 0.085253 |
| ***Plcd3*** | 0.154834 | 0.323924 | 0.119373 | 0.10714 | 0.169208 |
| ***Ppip5k1*** | 0.183285 | 0.214568 | 0.138216 | 0.193284 | 0.130378 |
| ***Dot1l*** | 0.262878 | 0.051006 | 0.11195 | 0.261655 | 0.128831 |
| ***Itpk1*** | 0.148677 | 0.304622 | 0.102269 | 0.126147 | 0.126457 |
| ***Pald1*** | 0.182723 | 0.388281 | 0.223631 | -0.076 | 0.087413 |
| ***Ipmk*** | 0.046001 | 0.271156 | 0.139187 | 0.141869 | 0.188646 |
| ***Styxl1*** | 0.085434 | 0.38235 | 0.216316 | 0.16633 | -0.06371 |
| ***Synj2*** | 0.53206 | 0.072132 | 0.075766 | 0.028447 | 0.074279 |
| ***Ppp1r1a*** | 0.112463 | 0.496859 | 0.091414 | -0.06973 | 0.141357 |
| ***Dusp28*** | -0.04355 | 0.467248 | 0.025673 | 0.02132 | 0.286107 |
| ***Ppfia3*** | 0.158383 | 0.393662 | 0.039268 | -0.09446 | 0.257249 |
| ***Pip5kl1*** | 0.04201 | 0.114227 | 0.107806 | 0.130713 | 0.353853 |
| ***Pi4k2a*** | 0.204324 | 0.087389 | 0.248339 | 0.139342 | 0.058594 |
| ***Ppp1r1b*** | 0.140007 | 0.073755 | 0.165653 | 0.11447 | 0.208167 |
| ***Ppip5k2*** | 0.2409 | 0.267555 | 0.039909 | 0.157937 | -0.0054 |
| ***Wbp11*** | 0.24718 | 0.167359 | 0.170837 | 0.1247 | -0.01957 |
| ***Nudt11*** | 0.207744 | 0.295592 | 0.190126 | -0.09366 | 0.081967 |
| ***Plpp7*** | 0.039665 | 0.158706 | 0.235529 | 0.065804 | 0.157987 |
| ***Pip4k2a*** | 0.009654 | 0.243289 | 0.314009 | 0.020464 | -0.0268 |
| ***Cilp*** | 0.196196 | 0.112743 | 0.211944 | 0.125967 | -0.10353 |
| ***Pip4p1*** | 0.173227 | 0.199086 | -0.01227 | 0.136314 | 0.033213 |
| ***Tpte2*** | 0.196855 | 0.242189 | 0.091417 | -0.1264 | 0.116997 |
| ***Pdxp*** | -0.06665 | 0.347624 | -0.07508 | 0.238516 | 0.04883 |
| ***Noct*** | -0.88356 | -0.31637 | 0.390114 | 0.667259 | 0.621873 |
| ***Pik3c2a*** | 0.005251 | -0.19112 | 0.220942 | 0.330514 | 0.110934 |
| ***Plcg1*** | 0.076618 | 0.17445 | 0.163278 | -0.05111 | 0.113243 |
| ***Ptprh*** | -0.12888 | 0.316562 | 0.014001 | 0.01256 | 0.24727 |
| ***Pik3r5*** | -0.01583 | 0.191325 | 0.133672 | -0.09727 | 0.247275 |
| ***Ppp4c*** | 0.13302 | 0.104166 | 0.13209 | 0.008344 | 0.07041 |
| ***Ppfibp2*** | 0.192439 | -0.12778 | 0.455438 | 0.068937 | -0.15047 |
| ***Ppp1r12c*** | 0.160747 | -0.03325 | 0.150428 | 0.094262 | 0.048543 |
| ***Ptprj*** | -0.24338 | -0.06362 | 0.132742 | 0.285686 | 0.29556 |
| ***Pik3c2b*** | 0.118141 | 0.169816 | 0.018153 | 0.04807 | 0.042186 |
| ***Pp2d1*** | -0.18443 | 0.106249 | 0.122304 | 0.031609 | 0.320249 |
| ***Acp6*** | 0.00336 | 0.217946 | 0.265117 | 0.028942 | -0.12906 |
| ***Pik3r6*** | -0.14476 | 0.123504 | 0.067777 | 0.110262 | 0.229527 |
| ***Nudt9*** | 0.115601 | 0.085854 | -0.00769 | 0.107995 | 0.022186 |
| ***Ptpn22*** | 0.143049 | 0.26221 | -0.00452 | 0.032591 | -0.12569 |
| ***Ip6k3*** | -0.01199 | 0.02635 | 0.094784 | -0.05264 | 0.250214 |
| ***Ptpn7*** | -0.01192 | 0.099795 | -0.02053 | -0.01408 | 0.250783 |
| ***Pip4k2c*** | -0.0982 | -0.04323 | 0.172879 | 0.128695 | 0.116593 |
| ***Sec16a*** | 0.240918 | -0.20771 | 0.069636 | 0.146102 | 0.020141 |
| ***Plcg2*** | 0.245478 | 0.197959 | -0.16078 | -0.02148 | -0.03416 |
| ***Dusp15*** | -0.02149 | 0.266653 | 0.10387 | -0.1665 | 0.04417 |
| ***Ptpn13*** | 0.152642 | 0.174414 | -0.14312 | 0.010445 | 0.031904 |
| ***G6pc2*** | -0.25135 | 0.21277 | 0.01594 | 0.100865 | 0.123581 |
| ***Pi4ka*** | 0.227025 | -0.35982 | 0.24356 | 0.245809 | -0.15747 |
| ***Ppp1r12a*** | 0.098903 | -0.20144 | 0.063026 | 0.192264 | 0.039148 |
| ***Tns3*** | -0.19971 | -0.21207 | 0.363141 | 0.094027 | 0.144582 |
| ***Alpl*** | 0.204856 | -0.35737 | 0.182296 | 0.151287 | -4E-05 |
| ***Ip6k1*** | 0.16336 | -0.01692 | 0.1482 | -0.08564 | -0.03103 |
| ***Pip5k1a*** | 0.06321 | -0.05888 | 0.018467 | 0.045516 | 0.103757 |
| ***Plcb3*** | 0.100853 | 0.006733 | -0.07192 | 0.02189 | 0.106291 |
| ***Synj1*** | 0.053365 | -0.21435 | 0.100531 | 0.094958 | 0.112213 |
| ***Eya4*** | -0.20085 | -0.04016 | 0.024283 | -0.05669 | 0.417637 |
| ***Pik3r2*** | 0.25572 | -0.09114 | 0.075034 | -0.03299 | -0.0685 |
| ***Pgam5*** | -0.05749 | -0.00839 | 0.044403 | 0.029856 | 0.127542 |
| ***Dusp11*** | 0.106243 | 0.006437 | -0.0123 | 0.092831 | -0.06284 |
| ***Inppl1*** | 0.322216 | -0.24926 | -0.13938 | 0.17604 | 0.003393 |
| ***Ilkap*** | 0.219149 | -0.04945 | 0.104791 | -0.04524 | -0.12361 |
| ***Ptpro*** | 0.078478 | 0.058644 | 0.238367 | -0.32473 | 0.048237 |
| ***Ptpn11*** | 0.277848 | -0.11732 | -0.15029 | 0.091185 | -0.0196 |
| ***Ppp1r16b*** | -0.14016 | 0.160949 | 0.147122 | -0.05253 | -0.05914 |
| ***Plcd1*** | -0.22994 | 0.19987 | 0.134655 | -0.07614 | 0.012606 |
| ***Set*** | 0.114621 | -0.38183 | 0.134717 | 0.162169 | 0.001224 |
| ***Ptpn1*** | 0.437937 | -0.19246 | -0.10771 | -0.12734 | 1.21E-05 |
| ***Pptc7*** | -0.13499 | 0.069644 | 0.110461 | 0.048371 | -0.0878 |
| ***Ip6k2*** | 0.115637 | 0.358906 | -0.08912 | -0.22299 | -0.17179 |
| ***Nudt3*** | 0.084008 | -0.06437 | 0.06127 | -0.08734 | -0.00345 |
| ***Pikfyve*** | 0.058301 | -0.00399 | 0.001554 | -0.02374 | -0.04363 |
| ***Ppp5c*** | 0.053311 | -0.00982 | -0.0183 | 0.007287 | -0.06057 |
| ***Plcb4*** | -0.04048 | 0.006694 | -0.11092 | -0.05022 | 0.159453 |
| ***Pik3r3*** | 0.293126 | -0.01107 | -0.15181 | -0.15931 | -0.02902 |
| ***Ppp1r14b*** | 0.142972 | -0.39431 | 0.030588 | 0.043426 | 0.116188 |
| ***Dusp21*** | 0.043405 | 0.004763 | 0.084511 | -0.15989 | -0.04354 |
| ***Nudt16*** | 0.025448 | 0.079923 | 0.030247 | -0.12524 | -0.08767 |
| ***Pdcd1*** | -0.00604 | -0.1547 | 0.023916 | 0.008132 | 0.034102 |
| ***Ptprf*** | 0.042717 | -0.03231 | -0.07087 | 0.078343 | -0.12215 |
| ***Ptpn20*** | -0.14898 | 0.04193 | -0.01656 | 0.124371 | -0.10872 |
| ***Inpp5j*** | -0.14525 | 0.163613 | -0.01849 | 0.088024 | -0.20384 |
| ***Ppp2r5d*** | 0.002409 | 0.042909 | -0.12701 | -0.10047 | 0.063347 |
| ***Ppp1r1c*** | -0.27586 | 0.104092 | -0.00758 | 0.053099 | 0.005664 |
| ***Cdipt*** | 0.109616 | -0.26769 | -0.09877 | 0.049172 | 0.077938 |
| ***Cdc25b*** | -0.20065 | 0.132434 | -0.28604 | 0.068269 | 0.153569 |
| ***Mtmr9*** | 0.184884 | -0.30989 | -0.11936 | 0.019357 | 0.090072 |
| ***Cdc25a*** | 0.247836 | 0.127974 | -0.01992 | -0.29305 | -0.20624 |
| ***Pik3ca*** | 0.052469 | 0.079205 | -0.0719 | -0.11625 | -0.08746 |
| ***Inpp5d*** | -0.02137 | 0.235639 | -0.03798 | -0.0511 | -0.2777 |
| ***Ssh3*** | 0.133561 | -0.08132 | -0.19492 | -0.03091 | 0.011427 |
| ***Plcd4*** | 0.050075 | -0.09895 | 0.070874 | -0.36691 | 0.18131 |
| ***Ppp1ca*** | -0.23883 | -0.18626 | 0.072024 | 0.083722 | 0.101551 |
| ***Pi4kb*** | 0.156048 | -0.16093 | 0.047615 | -0.00174 | -0.21587 |
| ***Dusp1*** | -0.19725 | -0.16361 | -0.0032 | 0.016991 | 0.170893 |
| ***Dusp13*** | -0.23168 | 0.054823 | -0.08683 | -0.04967 | 0.111607 |
| ***Pik3cd*** | -0.26532 | 0.047348 | 0.051176 | 0.075089 | -0.11023 |
| ***Ppp1r8*** | 0.117055 | -0.08432 | -0.05752 | -0.17902 | -0.00159 |
| ***Nt5c*** | 0.186892 | -0.13818 | -0.1346 | -0.09917 | -0.0306 |
| ***Nudt15*** | -0.1395 | 0.178961 | -0.11015 | -0.01179 | -0.1382 |
| ***Pip4k2b*** | 0.071975 | -0.51034 | -0.06187 | 0.152456 | 0.126419 |
| ***Ppfia2*** | -0.00437 | -0.02307 | 0.089932 | -0.24735 | -0.04213 |
| ***Ppp4r1*** | 0.106987 | -0.28949 | -0.13323 | -0.00462 | 0.092931 |
| ***Rasa1*** | 0.043313 | -0.05235 | -0.04907 | -0.01759 | -0.16055 |
| ***Nudt5*** | 0.273262 | -0.52989 | 0.006132 | -0.04722 | 0.052914 |
| ***Plcz1*** | -0.03042 | -0.04838 | -0.01187 | 0.080702 | -0.24691 |
| ***Sacm1l*** | -0.03572 | -0.0676 | -0.07831 | 0.008648 | -0.08617 |
| ***Ptpa*** | 0.113733 | -0.34736 | -0.21522 | 0.135324 | 0.051821 |
| ***Mtmr2*** | 0.082731 | 0.023319 | -0.09211 | -0.28693 | -0.00688 |
| ***Atp1a1*** | -0.15936 | -0.41783 | 0.023192 | 0.219062 | 0.054036 |
| ***Ppp2r3a*** | -0.2392 | -0.06188 | 0.091781 | 0.069591 | -0.17016 |
| ***Inpp5a*** | 0.324244 | -0.75624 | -0.2071 | 0.134429 | 0.193138 |
| ***Pik3r1*** | 0.090286 | -0.57779 | -0.2358 | 0.144529 | 0.263983 |
| ***Inpp5f*** | -0.05337 | -0.26163 | -0.06358 | 0.04925 | 0.013733 |
| ***Ptprc*** | 0.150834 | -0.04784 | 0.027627 | -0.21256 | -0.24261 |
| ***Ptpn12*** | -0.11226 | -0.20215 | -0.1271 | 0.055904 | 0.035783 |
| ***Ppp2r5e*** | 0.130195 | 0.014167 | -0.20408 | -0.17612 | -0.11958 |
| ***Plch1*** | 0.099831 | -0.20385 | -0.23486 | -0.02397 | -0.00027 |
| ***Pi4k2b*** | 0.056801 | -0.08395 | 0.033039 | -0.11308 | -0.25691 |
| ***Pik3cg*** | -0.02541 | -0.17348 | -0.01282 | 0.058653 | -0.23801 |
| ***Nudt4*** | -0.17071 | 0.369059 | -0.16415 | -0.21063 | -0.2155 |
[truncated: 62,665 more chars]
