# Supplementary material for: Sex-dependent dynamics of metabolism in primary mouse hepatocytes
Source: Arch Toxicol. 2021 Jul 9;95(9):3001–13. doi: 10.1007/s00204-021-03118-9 (PMC8380230; doi:10.1007/s00204-021-03118-9)

– SUPPLEMENTARY INFORMATION –

ARCHIVES OF TOXICOLOGY

Sex-dependent dynamics of metabolism in primary mouse hepatocytes

Luise Hochmuth, Christiane Körner, Fritzi Ott, Daniela Volke, Kaja Blagotinšek Cokan, Peter Juvan, Mario Brosch, Ute Hofmann, Ralf Hoffmann, Damjana Rozman, Thomas Berg, Madlen Matz-Soja

Correspondence: Madlen Matz-Soja (madlen.matz@medizin.uni-leipzig.de), Division of Hepatology, Clinic and Polyclinic for Oncology, Gastroenterology, Hepatology, Infectious Diseases, and Pneumology, University Hospital Leipzig, Leipzig, Germany

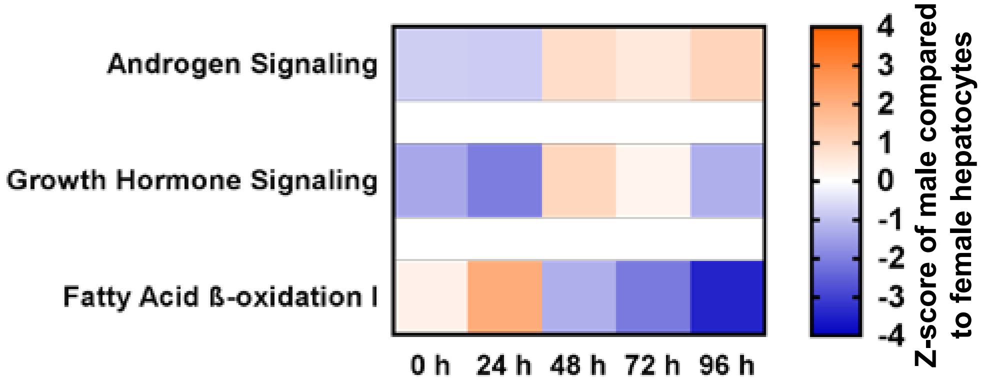

◄ Click here

Supplement 5 - interactive pdf: Selected signaling pathways and their detailed dynamics during culture of primary hepatocytes of both sexes. The heatmap shows the activation Z-score analysis based on RNA array data of male compared to female hepatocytes. The activation Z-score was calculated with IPA software. The p-value cut off of 0.05 was used for calculation. A click on the colored squares reveals the detailed pathway analysis of the appropriate pathway of male compared to female hepatocytes during culture done by IPA. Nodes with color gradients represent complexes whose individual components are regulated differently. Lines symbolize direct interaction. Dashed lines symbolize indirect interaction.

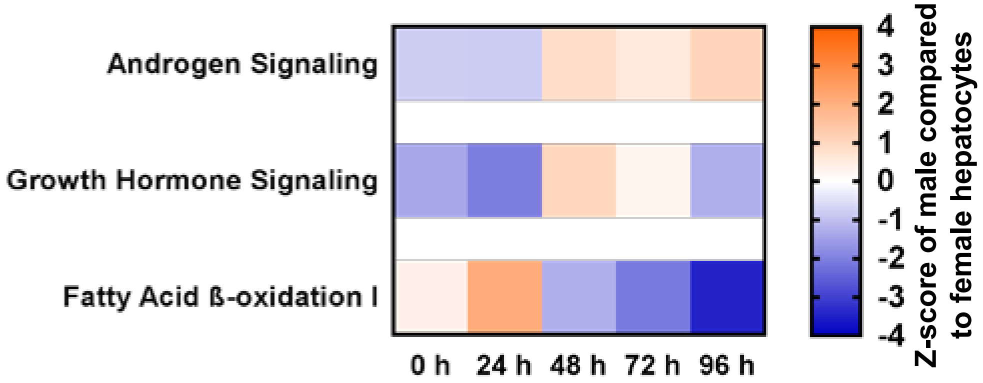

Androgen Signaling 0 h

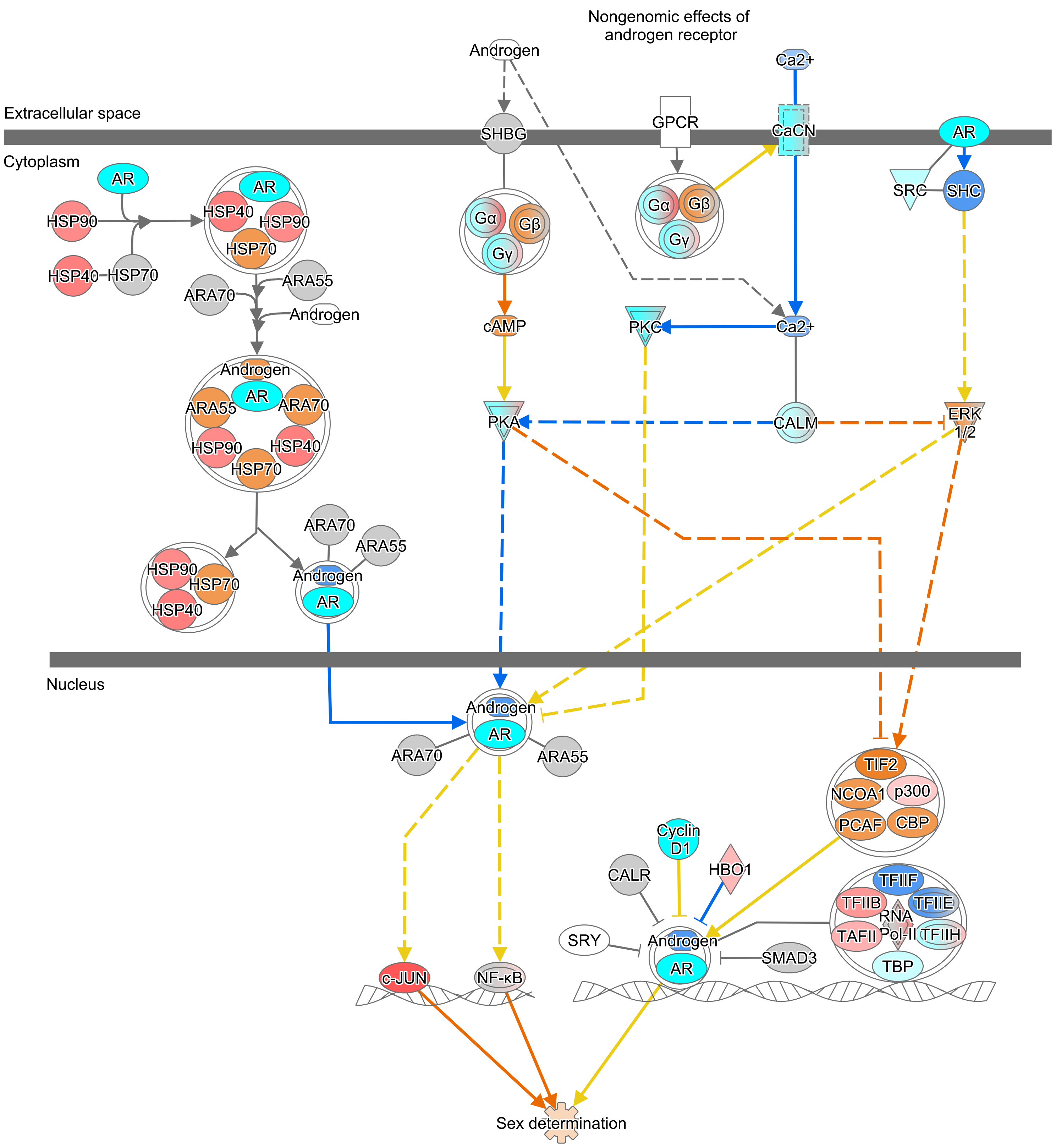

Supplement 5 - interactive pdf: Selected signaling pathways and their detailed dynamics during culture of primary hepatocytes of both sexes. The heatmap shows the activation Z-score analysis based on RNA array data of male compared to female hepatocytes. The activation Z-score was calculated with IPA software. The p-value cut off of 0.05 was used for calculation. A click on the colored squares reveals the detailed pathway analysis of the appropriate pathway of male compared to female hepatocytes during culture done by IPA. Nodes with color gradients represent complexes whose individual components are regulated differently. Lines symbolize direct interaction. Dashed lines symbolize indirect interaction.

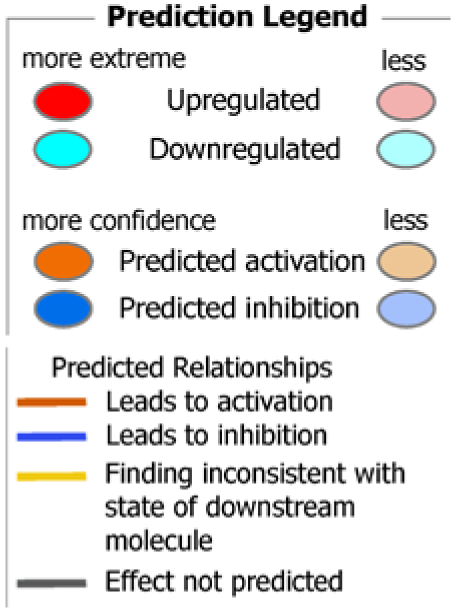

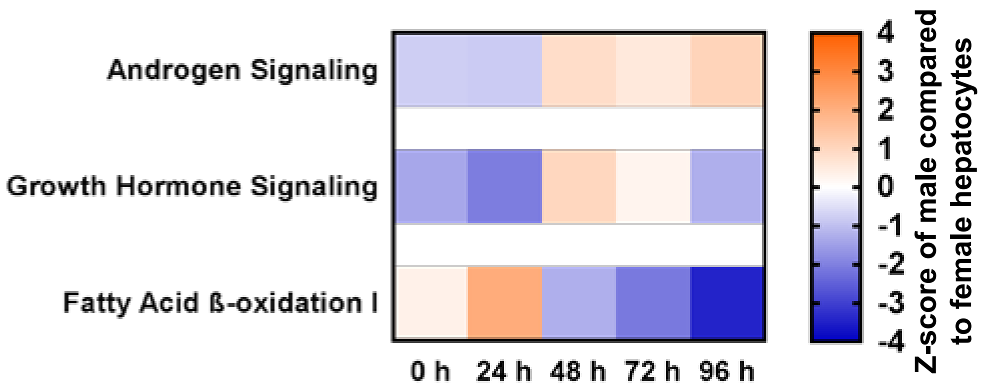

Supplement 5 - interactive pdf: Selected signaling pathways and their detailed dynamics during culture of primary hepatocytes of both sexes. The heatmap shows the activation Z-score analysis based on RNA array data of male compared to female hepatocytes. The activation Z-score was calculated with IPA software. The p-value cut off of 0.05 was used for calculation. A click on the colored squares reveals the detailed pathway analysis of the appropriate pathway of male compared to female hepatocytes during culture done by IPA. Nodes with color gradients represent complexes whose individual components are regulated differently. Lines symbolize direct interaction. Dashed lines symbolize indirect interaction.

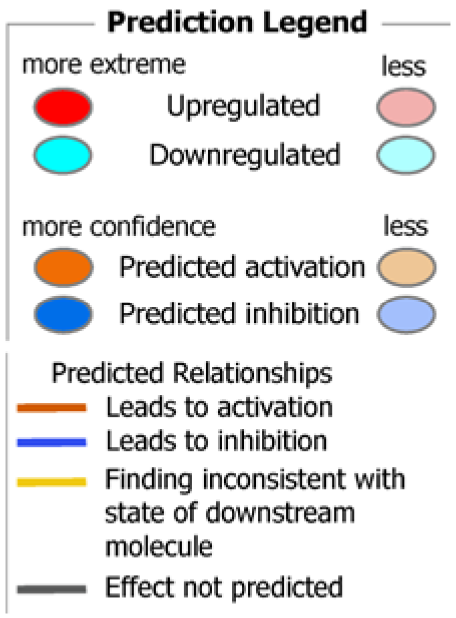

Androgen Signaling 24 h

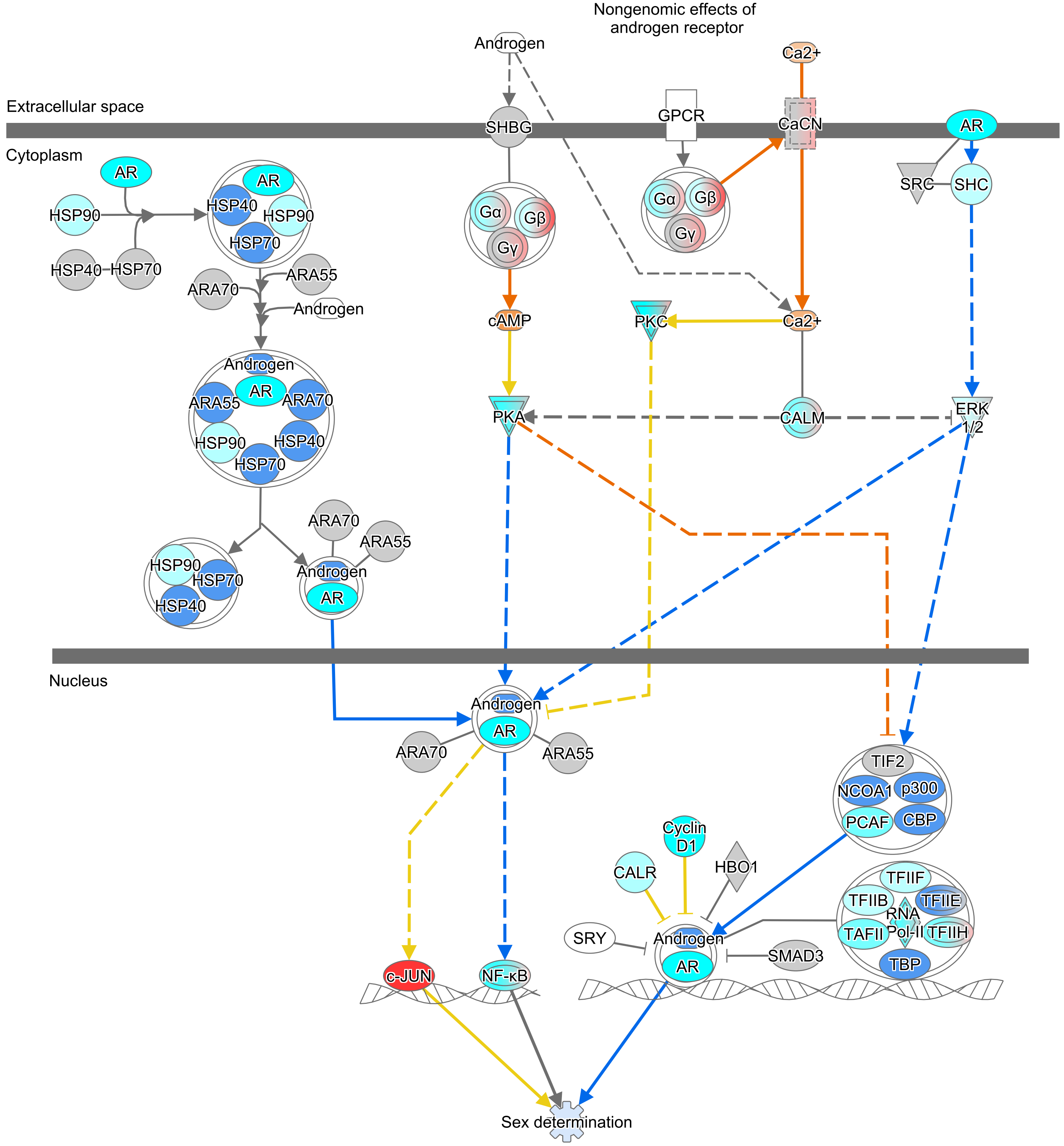

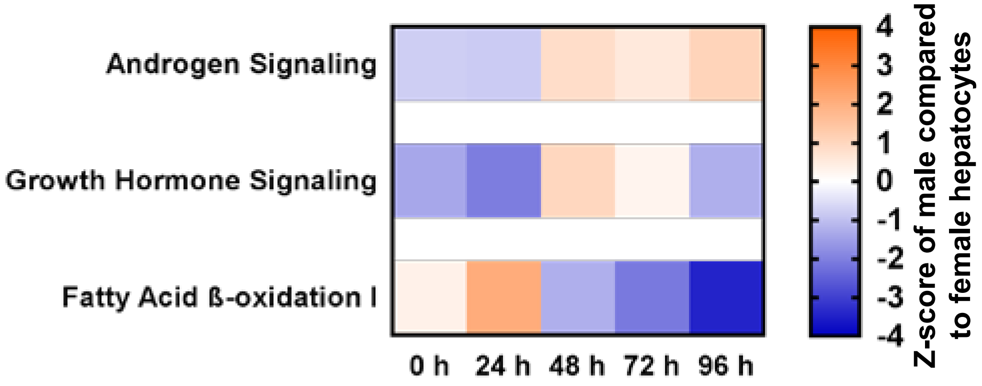

Supplement 5 - interactive pdf: Selected signaling pathways and their detailed dynamics during culture of primary hepatocytes of both sexes. The heatmap shows the activation Z-score analysis based on RNA array data of male compared to female hepatocytes. The activation Z-score was calculated with IPA software. The p-value cut off of 0.05 was used for calculation. A click on the colored squares reveals the detailed pathway analysis of the appropriate pathway of male compared to female hepatocytes during culture done by IPA. Nodes with color gradients represent complexes whose individual components are regulated differently. Lines symbolize direct interaction. Dashed lines symbolize indirect interaction.

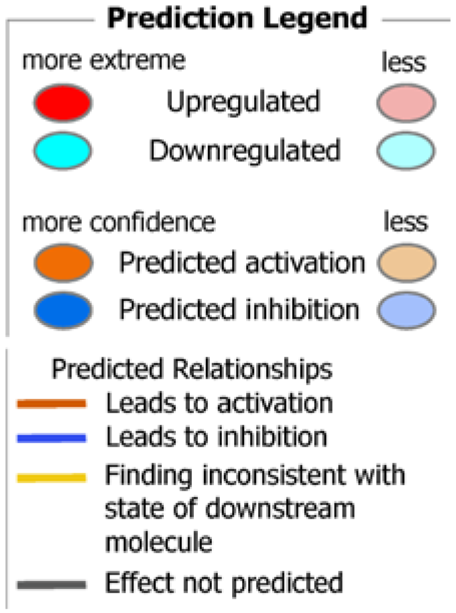

Androgen Signaling 48 h

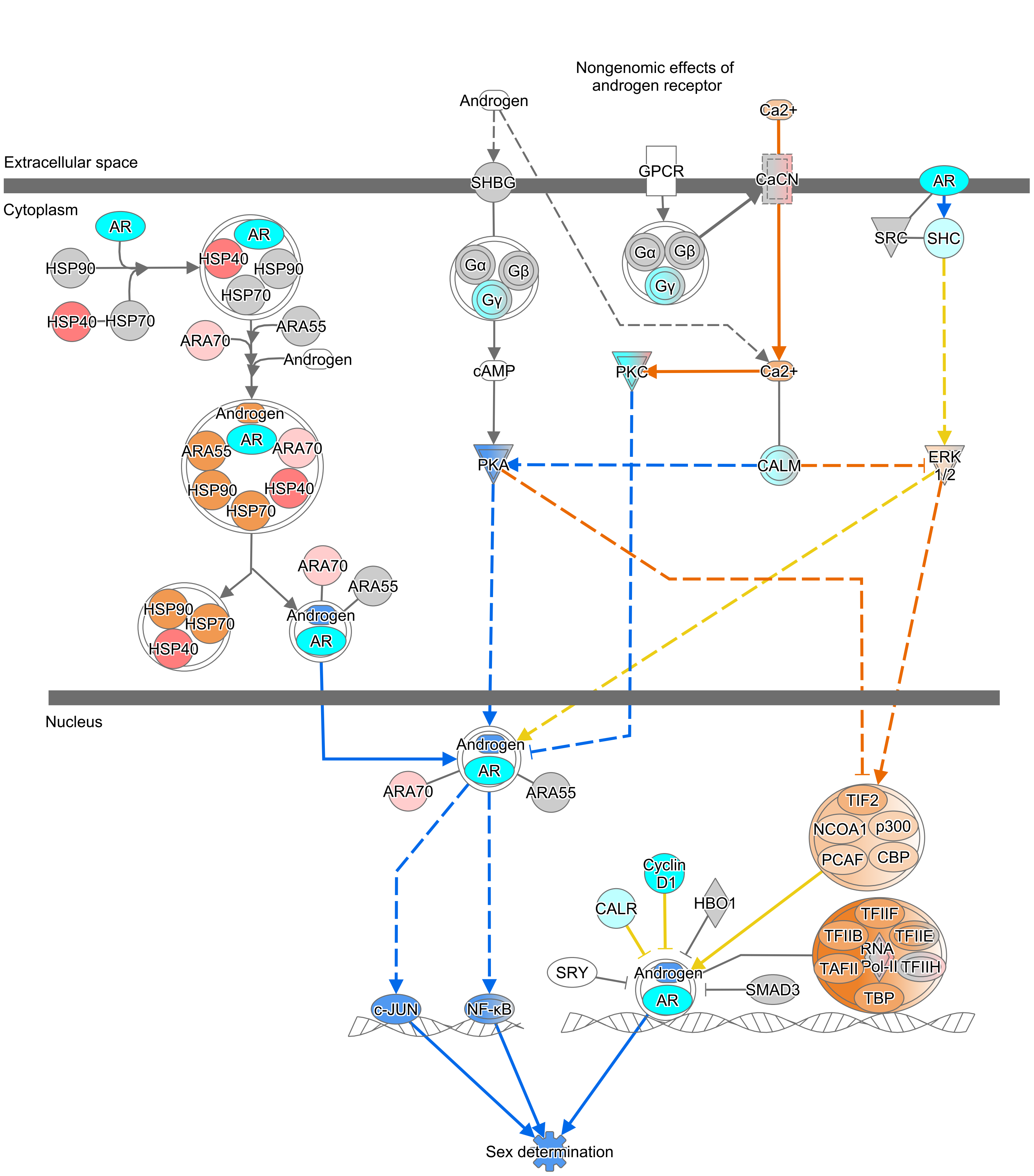

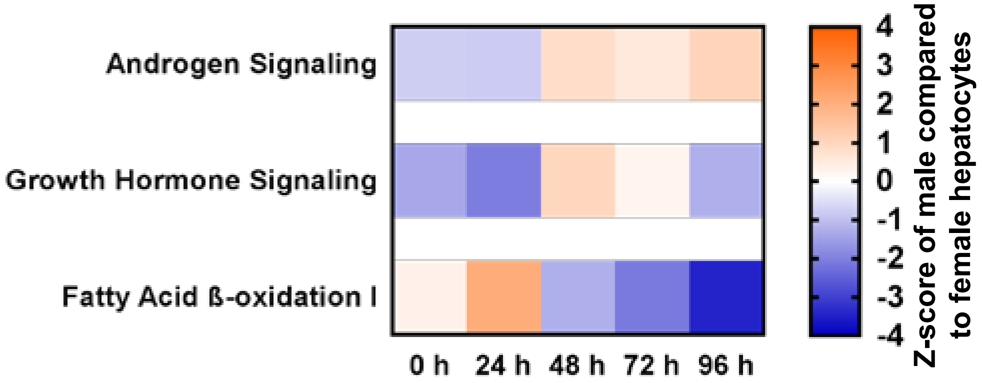

Supplement 5 - interactive pdf: Selected signaling pathways and their detailed dynamics during culture of primary hepatocytes of both sexes. The heatmap shows the activation Z-score analysis based on RNA array data of male compared to female hepatocytes. The activation Z-score was calculated with IPA software. The p-value cut off of 0.05 was used for calculation. A click on the colored squares reveals the detailed pathway analysis of the appropriate pathway of male compared to female hepatocytes during culture done by IPA. Nodes with color gradients represent complexes whose individual components are regulated differently. Lines symbolize direct interaction. Dashed lines symbolize indirect interaction.

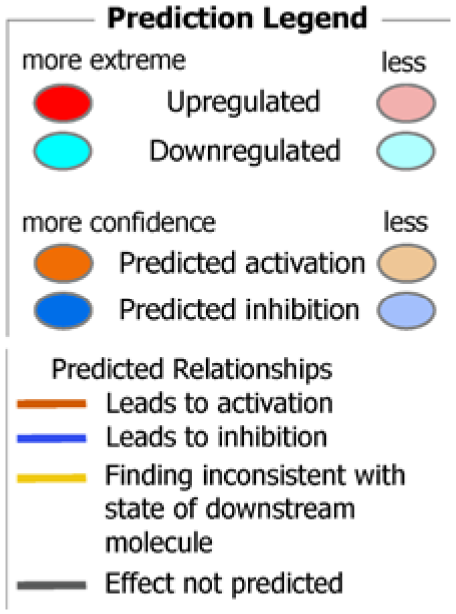

Androgen Signaling 72 h

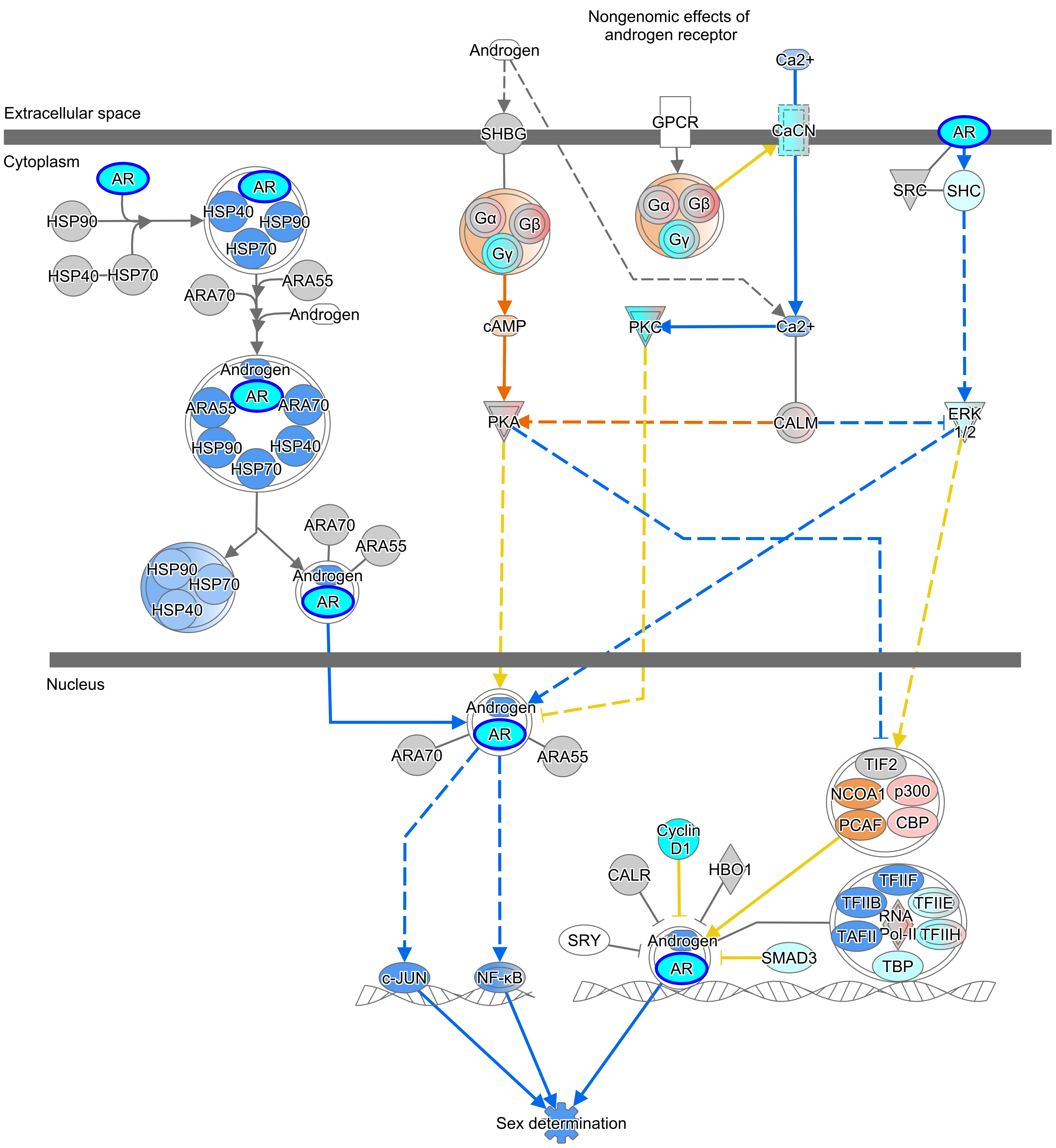

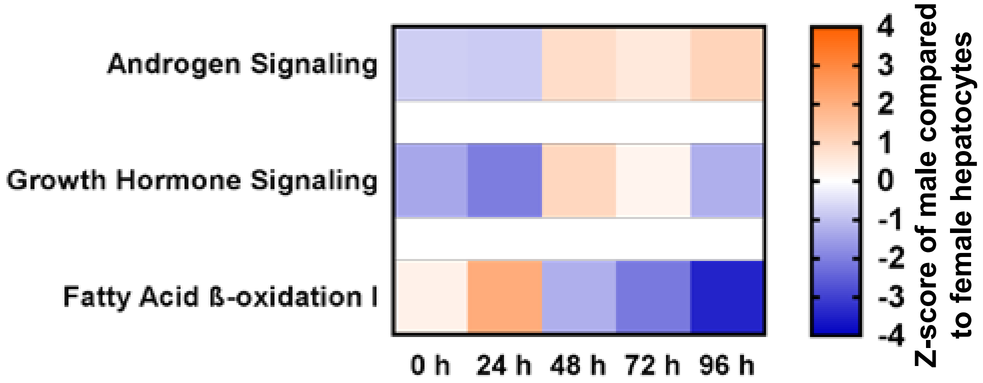

Supplement 5 - interactive pdf: Selected signaling pathways and their detailed dynamics during culture of primary hepatocytes of both sexes. The heatmap shows the activation Z-score analysis based on RNA array data of male compared to female hepatocytes. The activation Z-score was calculated with IPA software. The p-value cut off of 0.05 was used for calculation. A click on the colored squares reveals the detailed pathway analysis of the appropriate pathway of male compared to female hepatocytes during culture done by IPA. Nodes with color gradients represent complexes whose individual components are regulated differently. Lines symbolize direct interaction. Dashed lines symbolize indirect interaction.

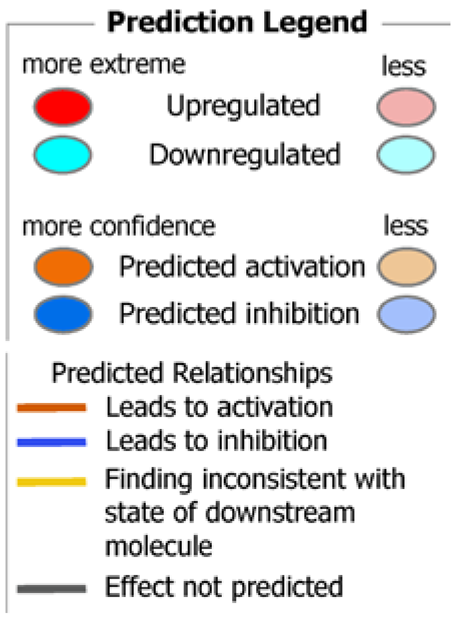

Androgen Signaling 96 h

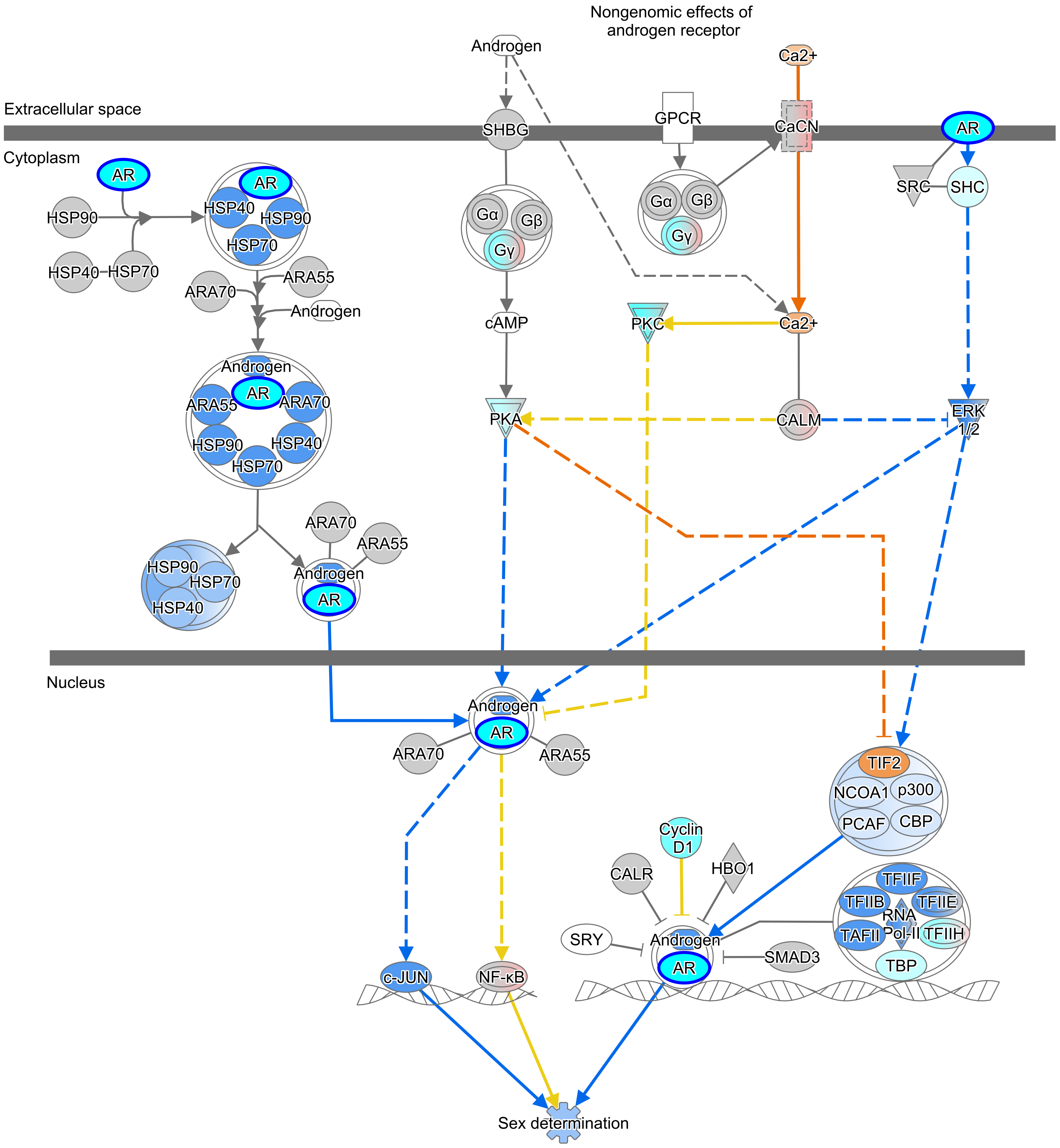

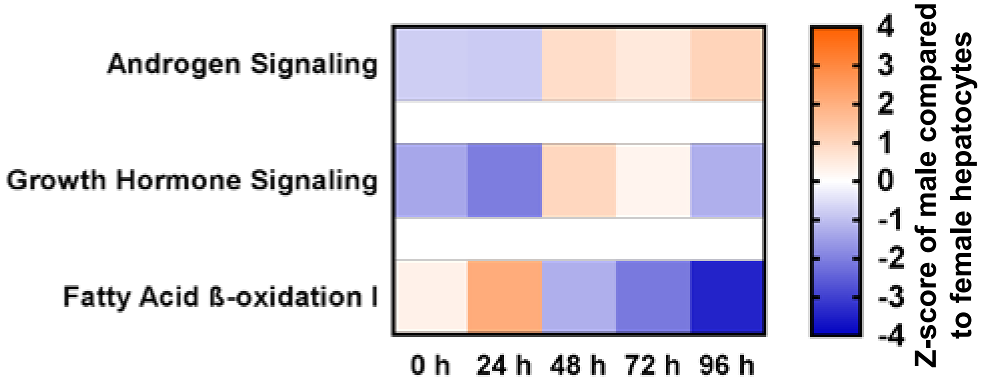

Growth Hormone Signaling 0 h

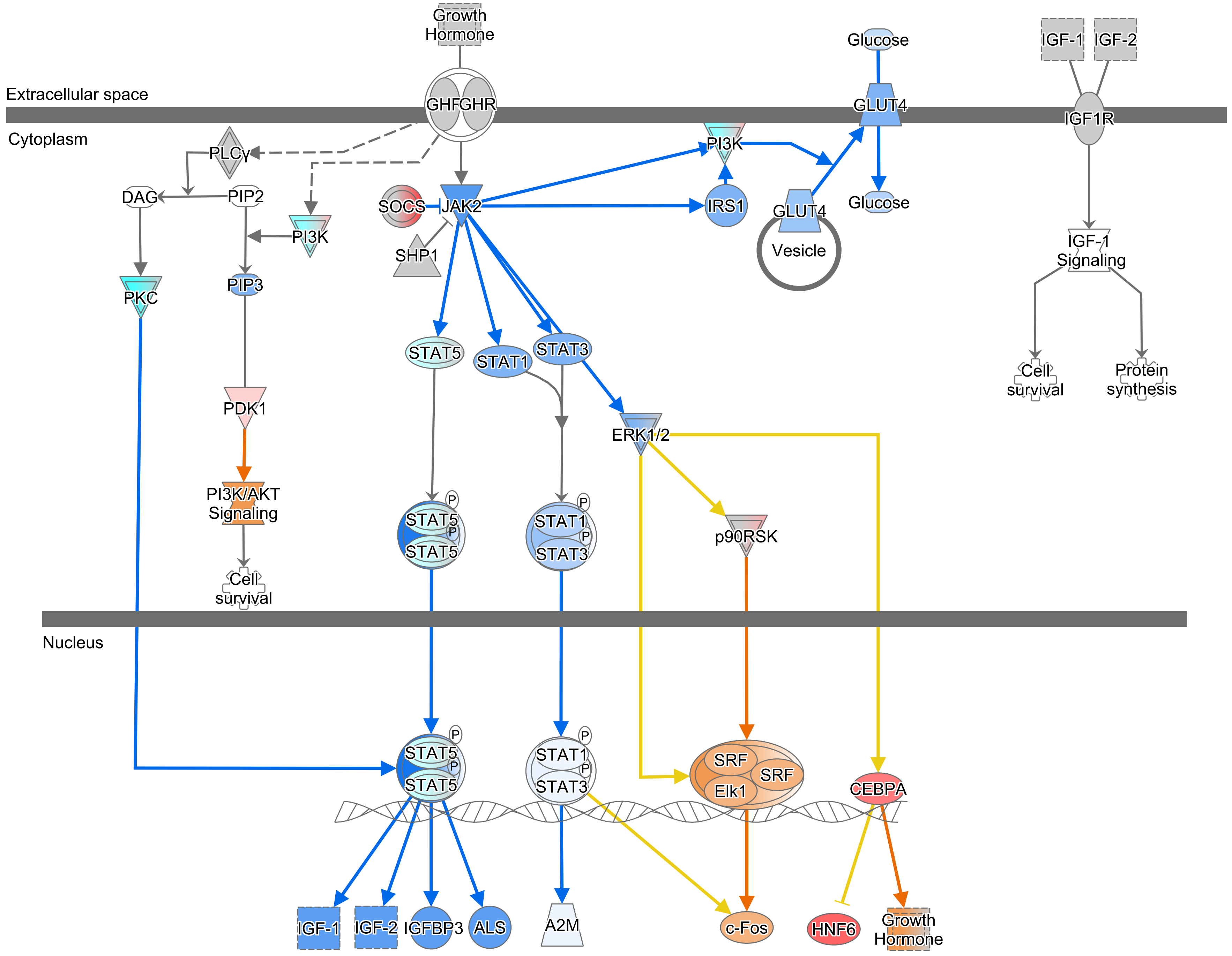

Supplement 5 - interactive pdf: Selected signaling pathways and their detailed dynamics during culture of primary hepatocytes of both sexes. The heatmap shows the activation Z-score analysis based on RNA array data of male compared to female hepatocytes. The activation Z-score was calculated with IPA software. The p-value cut off of 0.05 was used for calculation. A click on the colored squares reveals the detailed pathway analysis of the appropriate pathway of male compared to female hepatocytes during culture done by IPA. Nodes with color gradients represent complexes whose individual components are regulated differently. Lines symbolize direct interaction. Dashed lines symbolize indirect interaction.

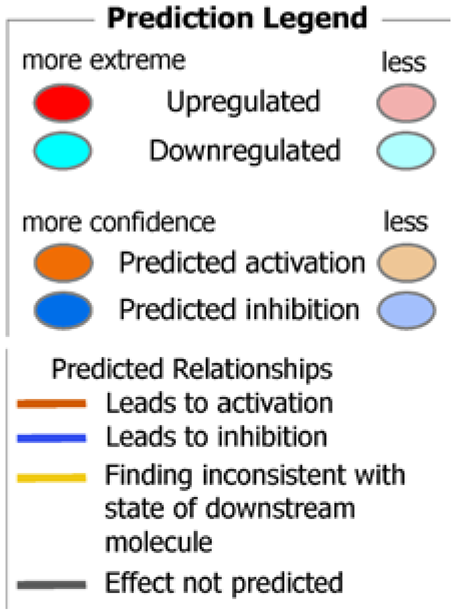

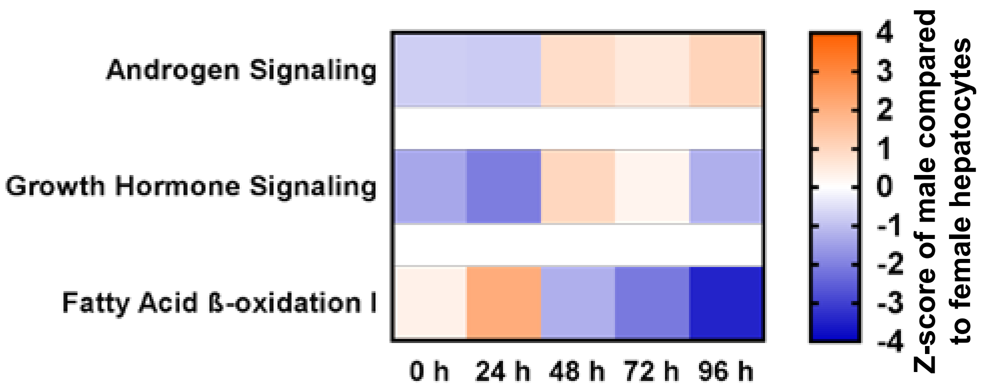

Growth Hormone Signaling 24 h

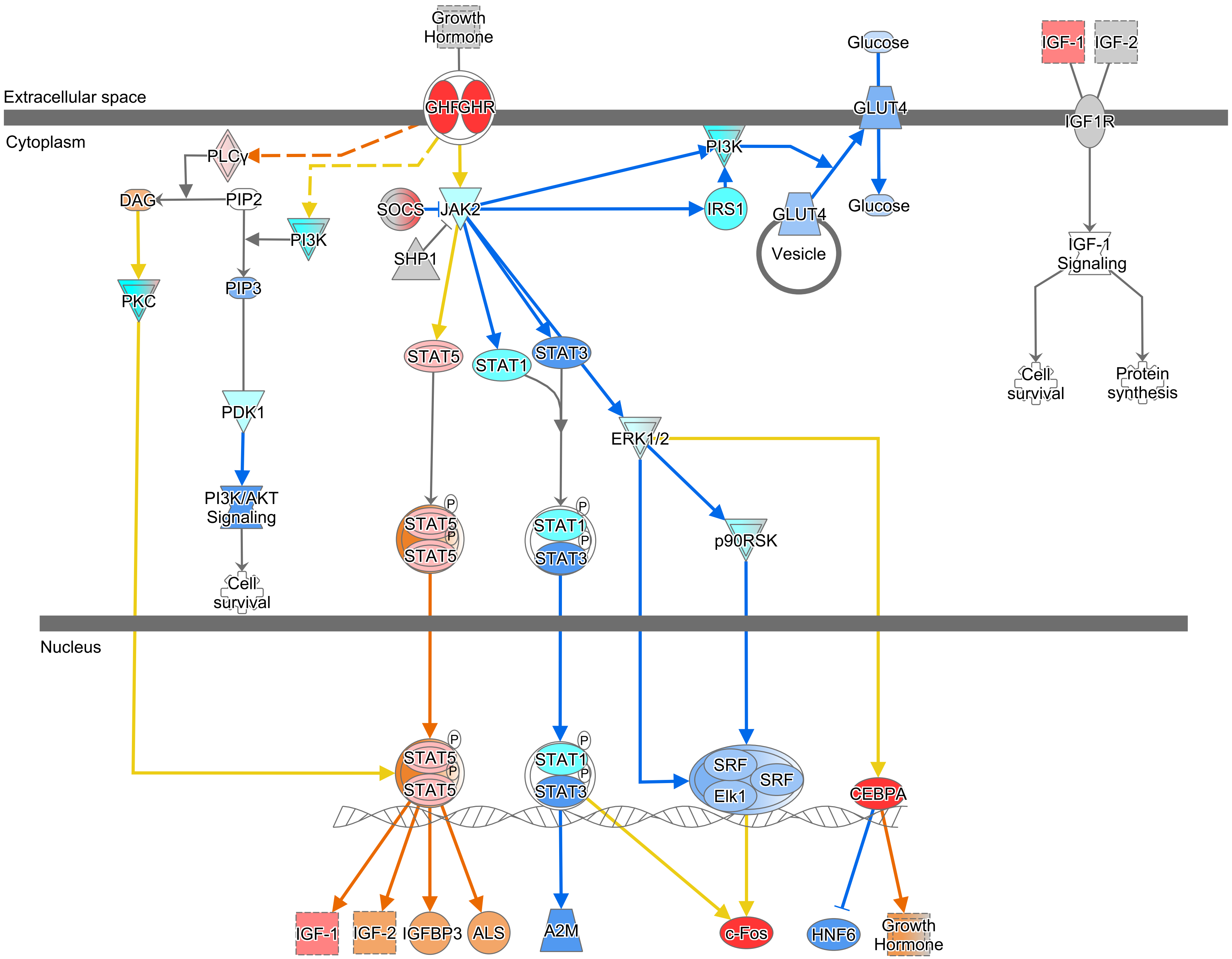

Supplement 5 - interactive pdf: Selected signaling pathways and their detailed dynamics during culture of primary hepatocytes of both sexes. The heatmap shows the activation Z-score analysis based on RNA array data of male compared to female hepatocytes. The activation Z-score was calculated with IPA software. The p-value cut off of 0.05 was used for calculation. A click on the colored squares reveals the detailed pathway analysis of the appropriate pathway of male compared to female hepatocytes during culture done by IPA. Nodes with color gradients represent complexes whose individual components are regulated differently. Lines symbolize direct interaction. Dashed lines symbolize indirect interaction.

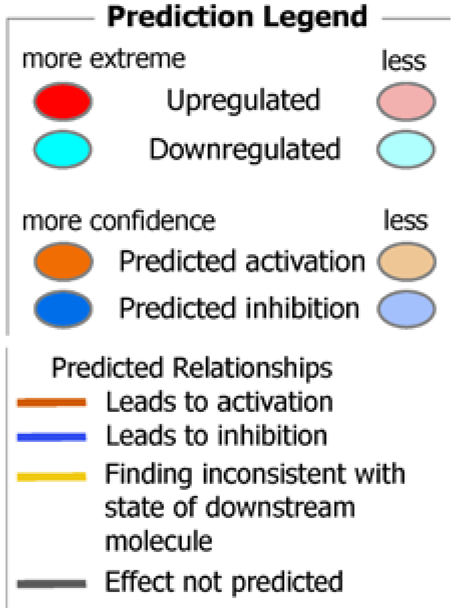

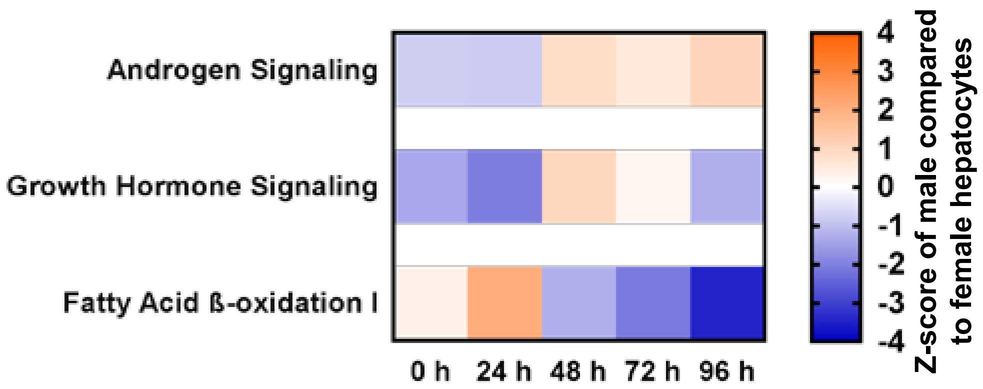

Growth Hormone Signaling 48 h

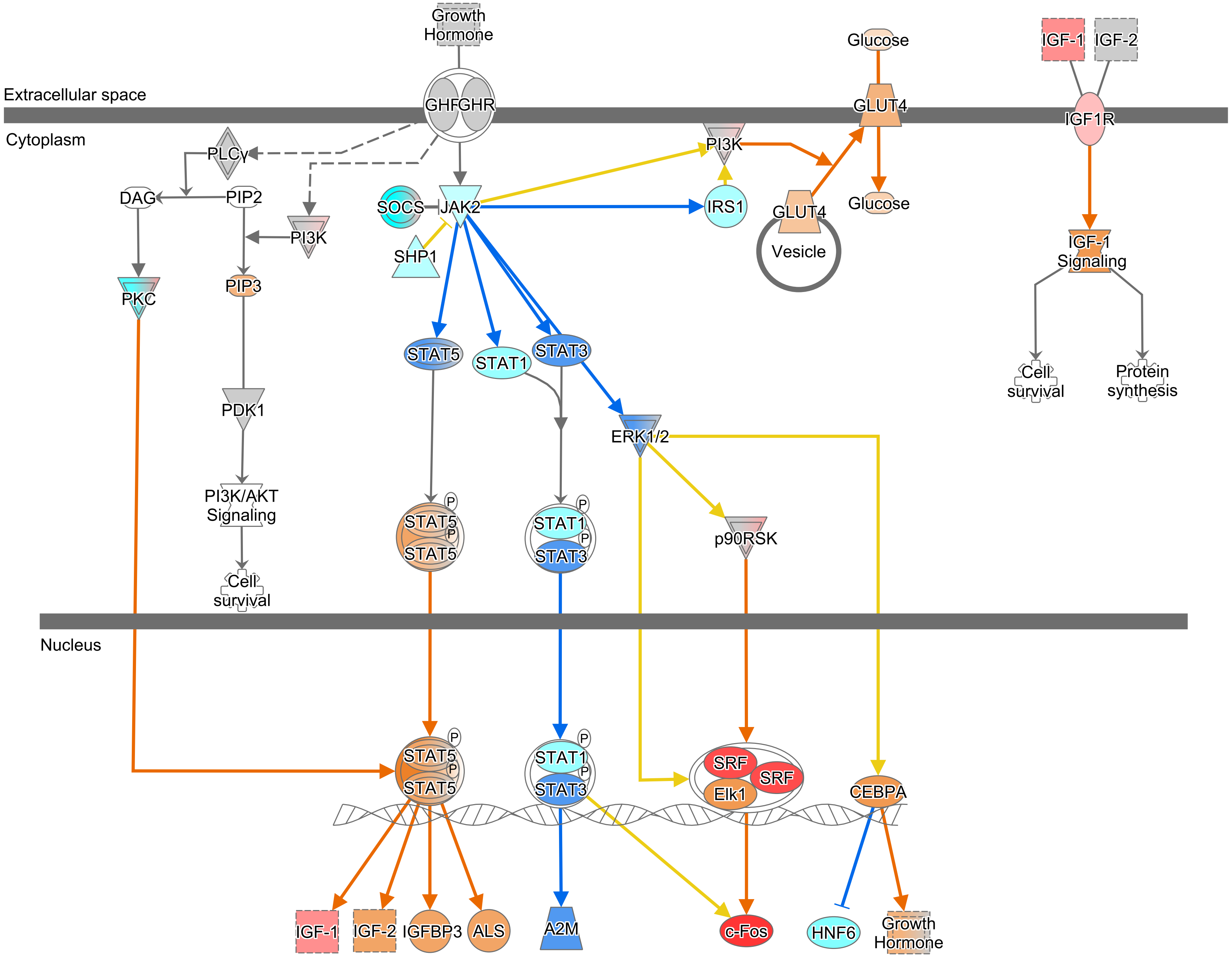

Supplement 5 - interactive pdf: Selected signaling pathways and their detailed dynamics during culture of primary hepatocytes of both sexes. The heatmap shows the activation Z-score analysis based on RNA array data of male compared to female hepatocytes. The activation Z-score was calculated with IPA software. The p-value cut off of 0.05 was used for calculation. A click on the colored squares reveals the detailed pathway analysis of the appropriate pathway of male compared to female hepatocytes during culture done by IPA. Nodes with color gradients represent complexes whose individual components are regulated differently. Lines symbolize direct interaction. Dashed lines symbolize indirect interaction.

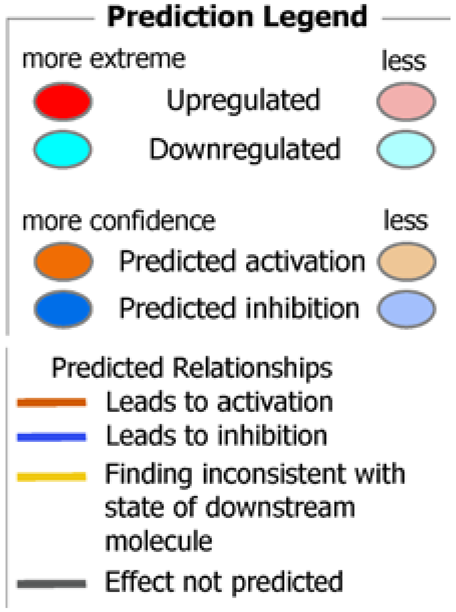

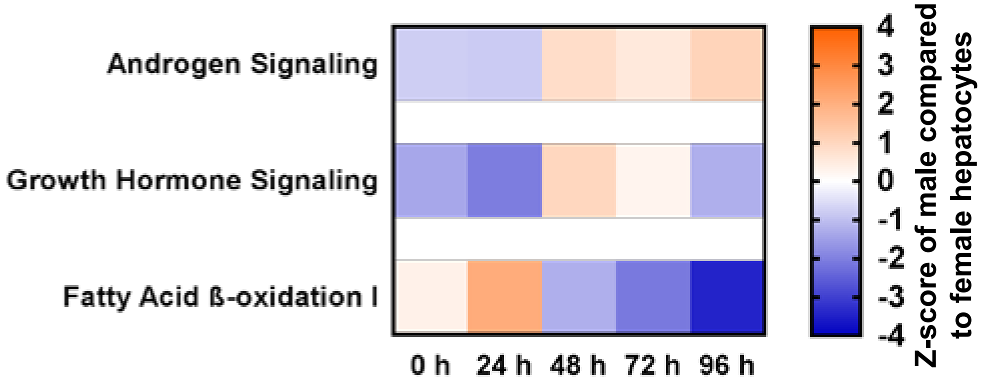

Growth Hormone Signaling 72 h

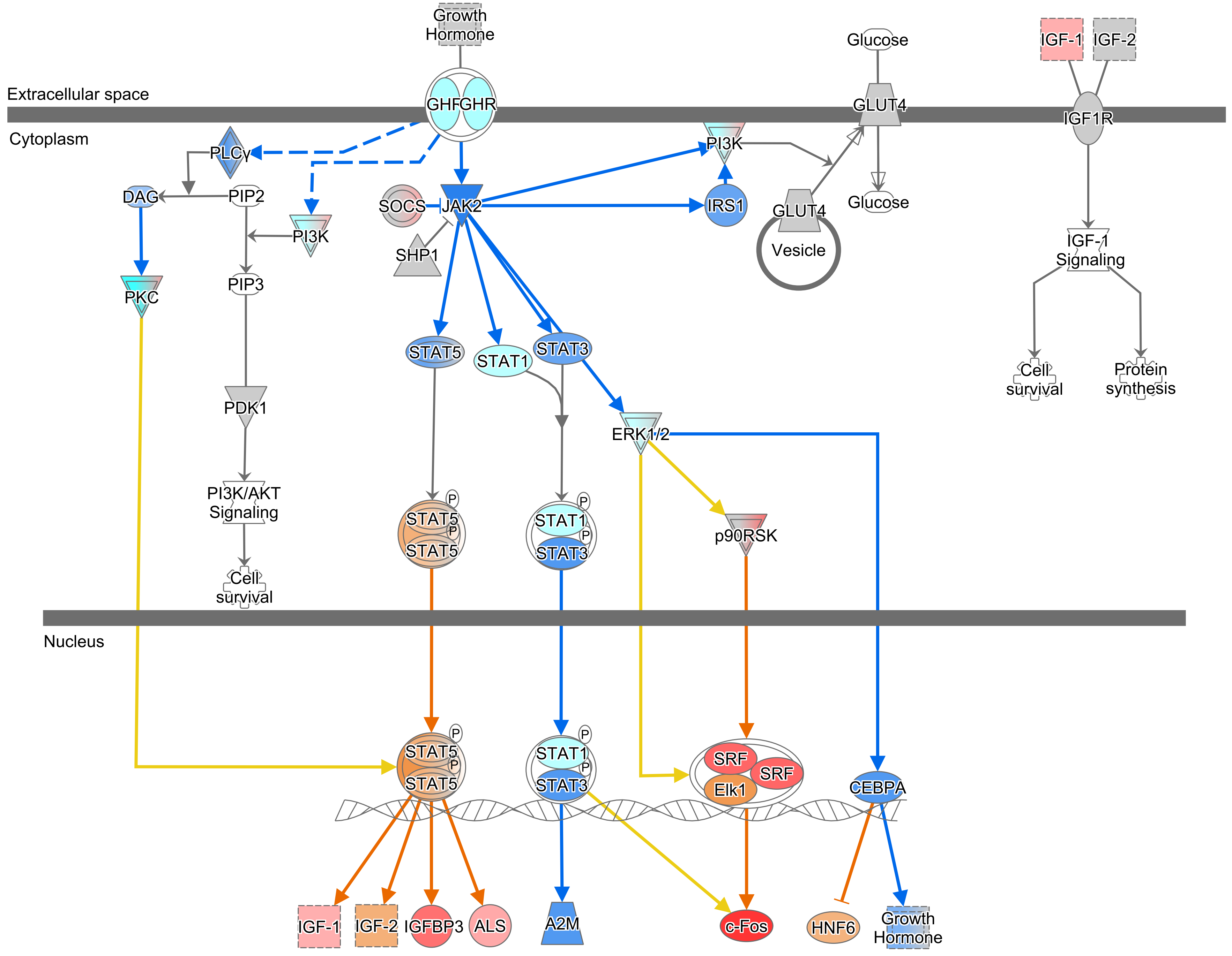

Supplement 5 - interactive pdf: Selected signaling pathways and their detailed dynamics during culture of primary hepatocytes of both sexes. The heatmap shows the activation Z-score analysis based on RNA array data of male compared to female hepatocytes. The activation Z-score was calculated with IPA software. The p-value cut off of 0.05 was used for calculation. A click on the colored squares reveals the detailed pathway analysis of the appropriate pathway of male compared to female hepatocytes during culture done by IPA. Nodes with color gradients represent complexes whose individual components are regulated differently. Lines symbolize direct interaction. Dashed lines symbolize indirect interaction.

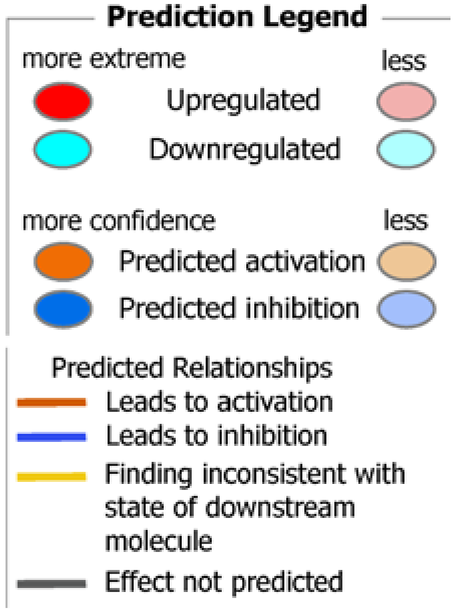

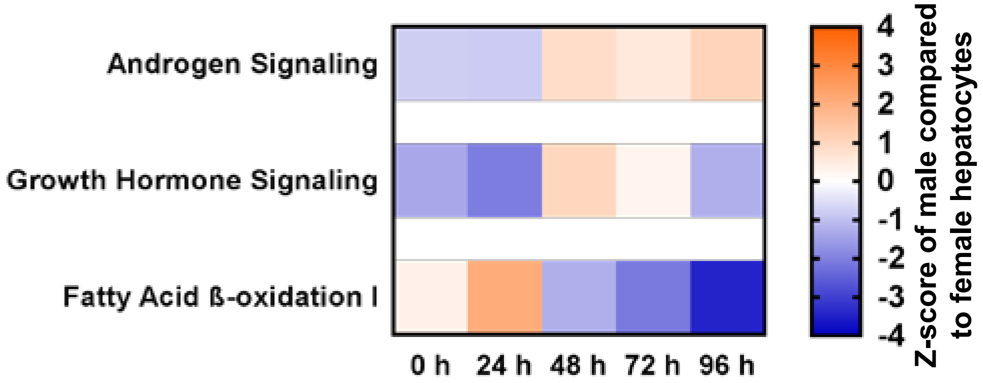

Supplement 5 - interactive pdf: Selected signaling pathways and their detailed dynamics during culture of primary hepatocytes of both sexes. The heatmap shows the activation Z-score analysis based on RNA array data of male compared to female hepatocytes. The activation Z-score was calculated with IPA software. The p-value cut off of 0.05 was used for calculation. A click on the colored squares reveals the detailed pathway analysis of the appropriate pathway of male compared to female hepatocytes during culture done by IPA. Nodes with color gradients represent complexes whose individual components are regulated differently. Lines symbolize direct interaction. Dashed lines symbolize indirect interaction.

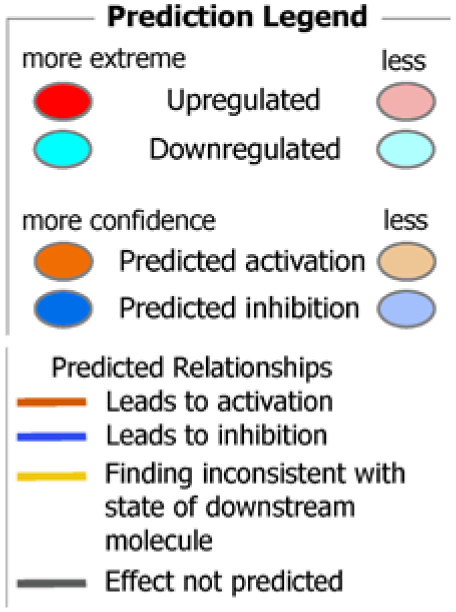

Growth Hormone Signaling 96 h

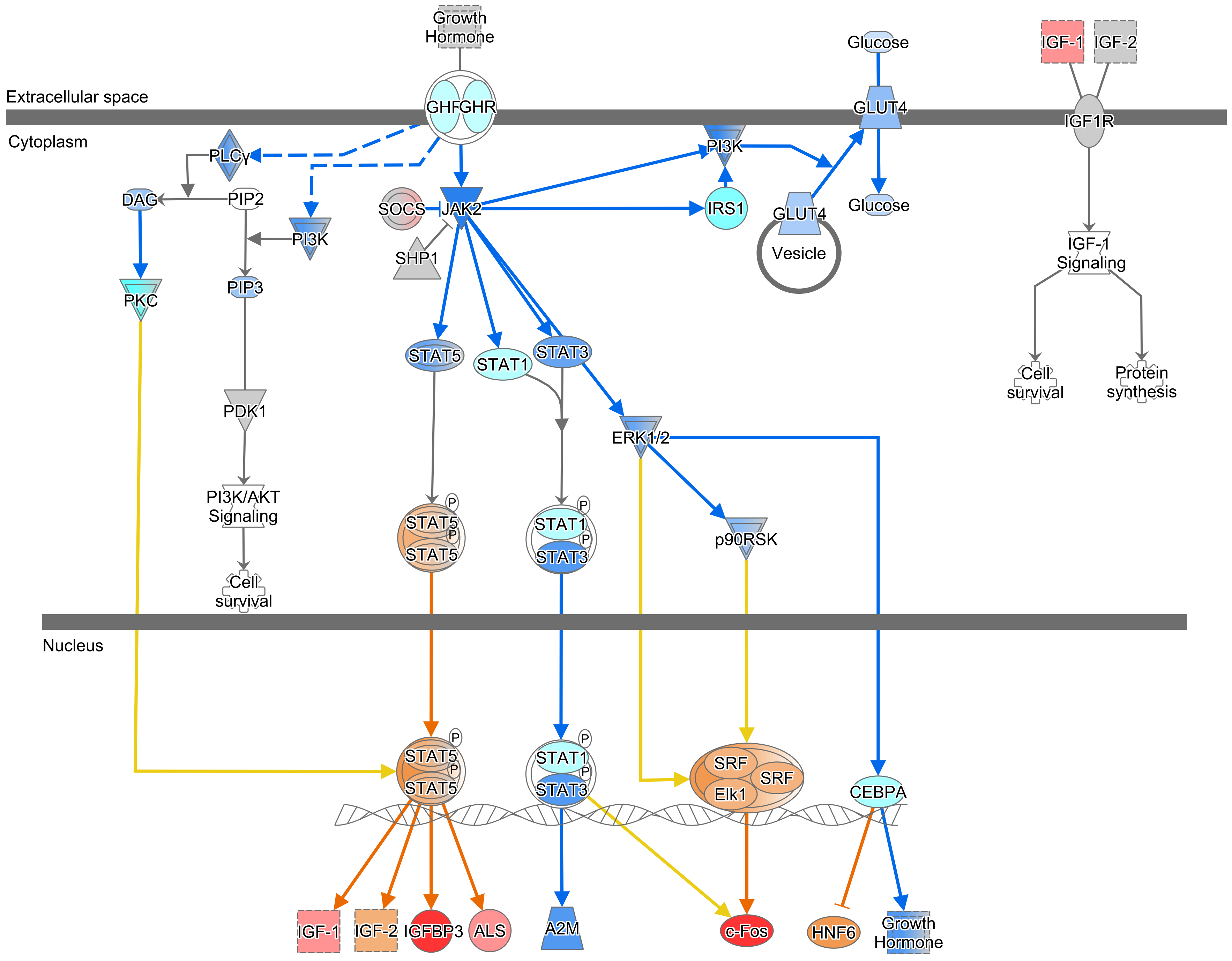

Fatty Acid β-oxidation 0 h

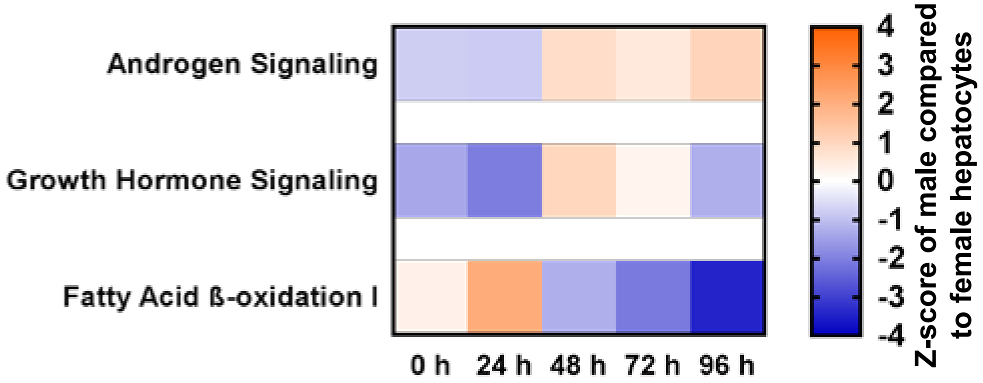

Supplement 5 - interactive pdf: Selected signaling pathways and their detailed dynamics during culture of primary hepatocytes of both sexes. The heatmap shows the activation Z-score analysis based on RNA array data of male compared to female hepatocytes. The activation Z-score was calculated with IPA software. The p-value cut off of 0.05 was used for calculation. A click on the colored squares reveals the detailed pathway analysis of the appropriate pathway of male compared to female hepatocytes during culture done by IPA. Nodes with color gradients represent complexes whose individual components are regulated differently. Lines symbolize direct interaction. Dashed lines symbolize indirect interaction.

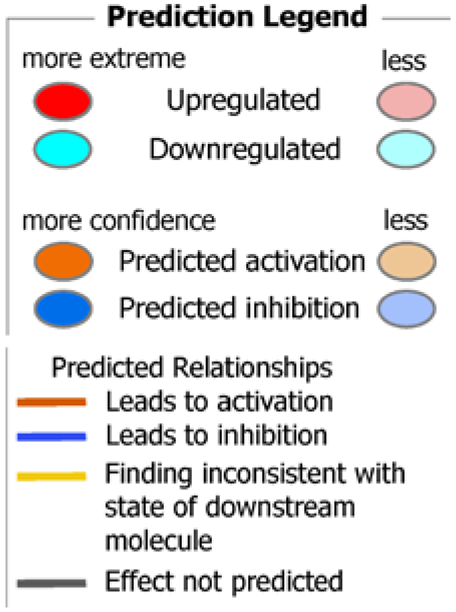

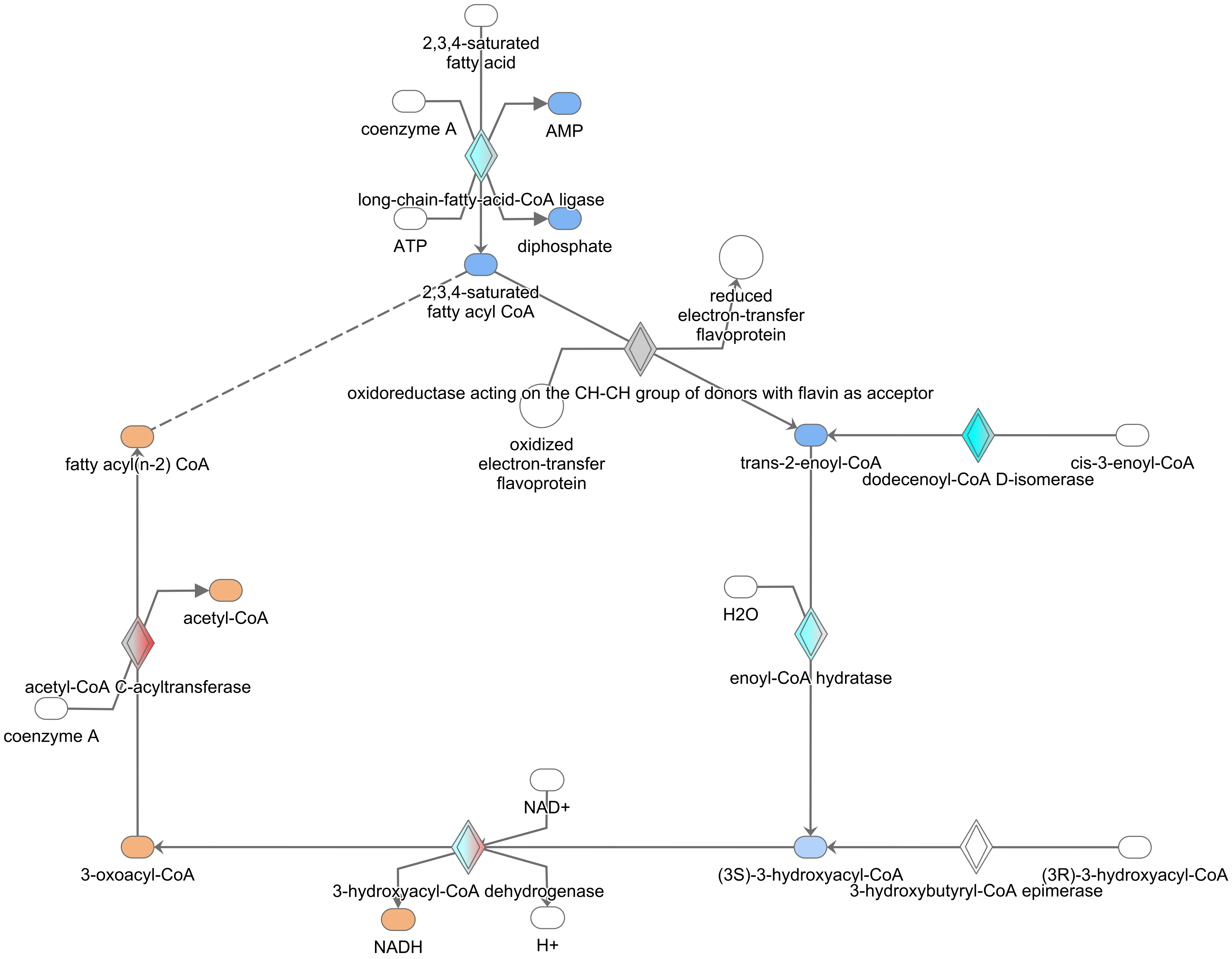

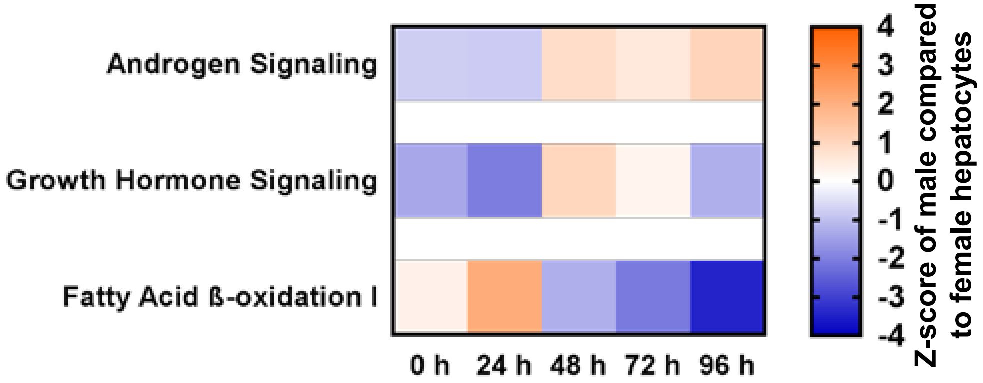

Fatty Acid β-oxidation 24 h

Supplement 5 - interactive pdf: Selected signaling pathways and their detailed dynamics during culture of primary hepatocytes of both sexes. The heatmap shows the activation Z-score analysis based on RNA array data of male compared to female hepatocytes. The activation Z-score was calculated with IPA software. The p-value cut off of 0.05 was used for calculation. A click on the colored squares reveals the detailed pathway analysis of the appropriate pathway of male compared to female hepatocytes during culture done by IPA. Nodes with color gradients represent complexes whose individual components are regulated differently. Lines symbolize direct interaction. Dashed lines symbolize indirect interaction.

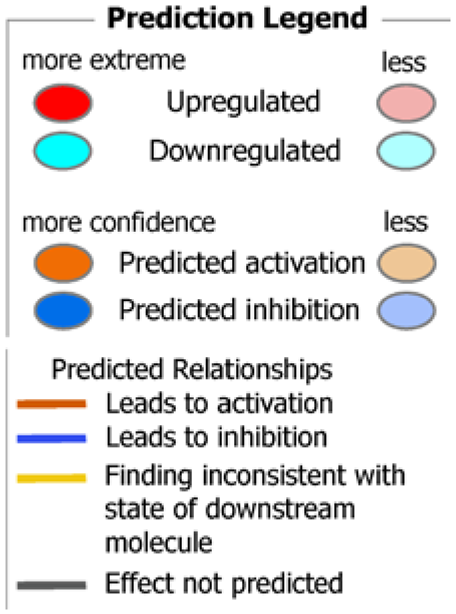

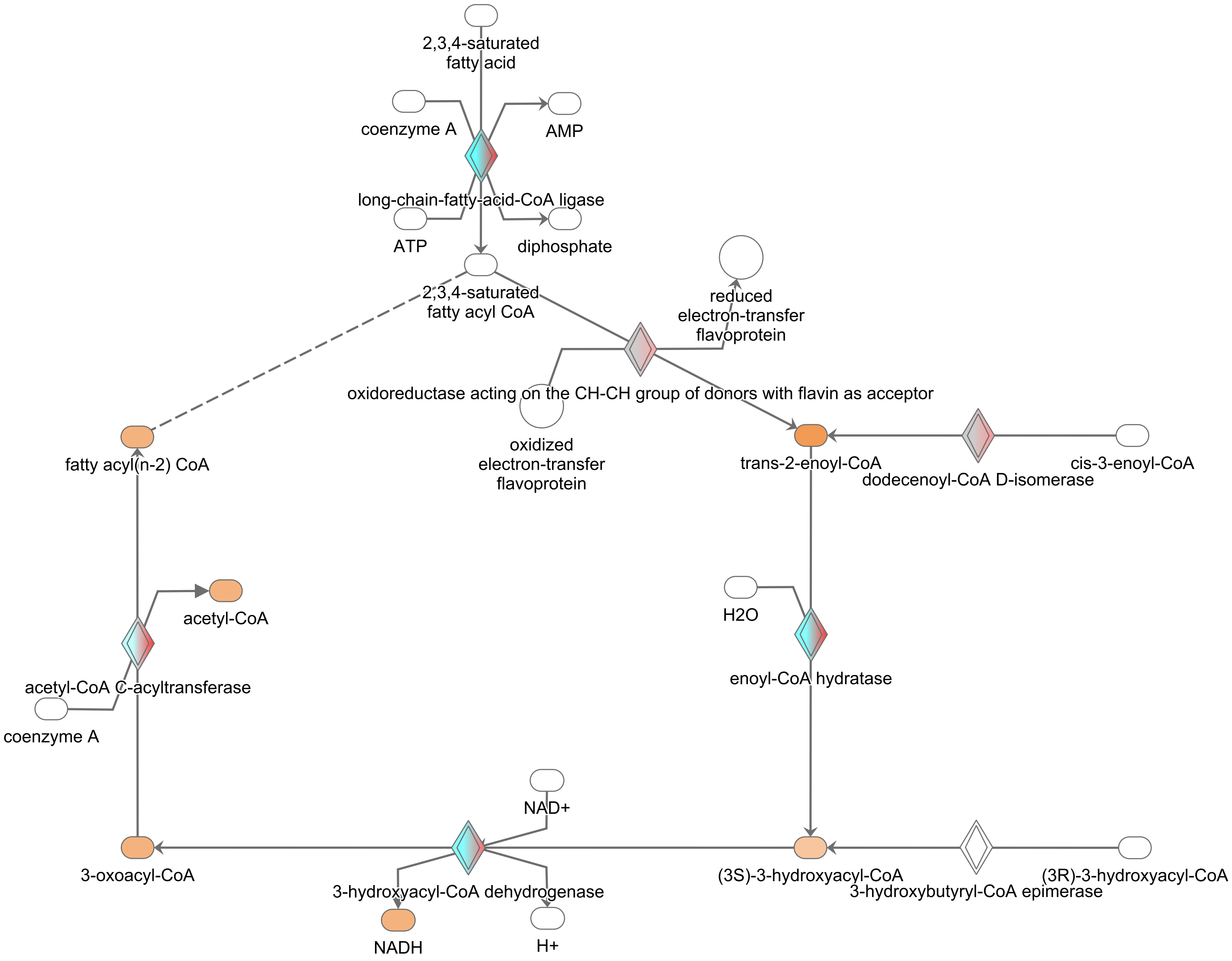

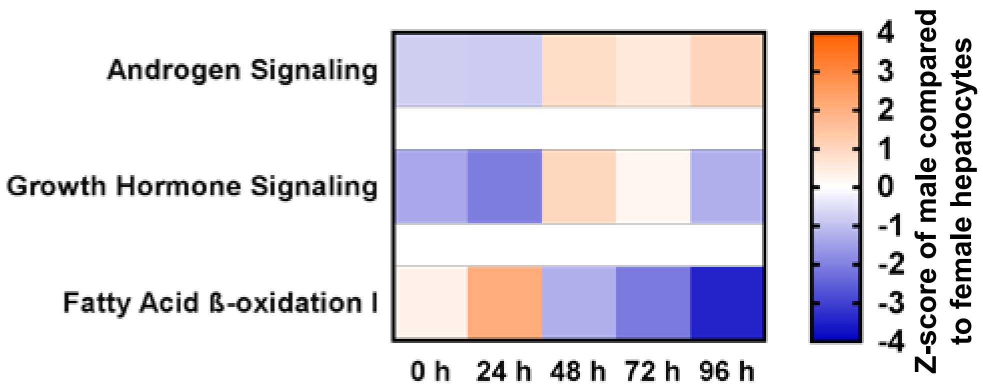

Supplement 5 - interactive pdf: Selected signaling pathways and their detailed dynamics during culture of primary hepatocytes of both sexes. The heatmap shows the activation Z-score analysis based on RNA array data of male compared to female hepatocytes. The activation Z-score was calculated with IPA software. The p-value cut off of 0.05 was used for calculation. A click on the colored squares reveals the detailed pathway analysis of the appropriate pathway of male compared to female hepatocytes during culture done by IPA. Nodes with color gradients represent complexes whose individual components are regulated differently. Lines symbolize direct interaction. Dashed lines symbolize indirect interaction.

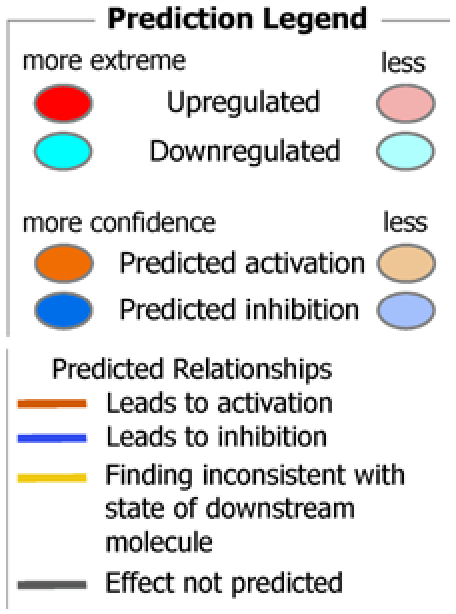

Fatty Acid β-oxidation 48 h

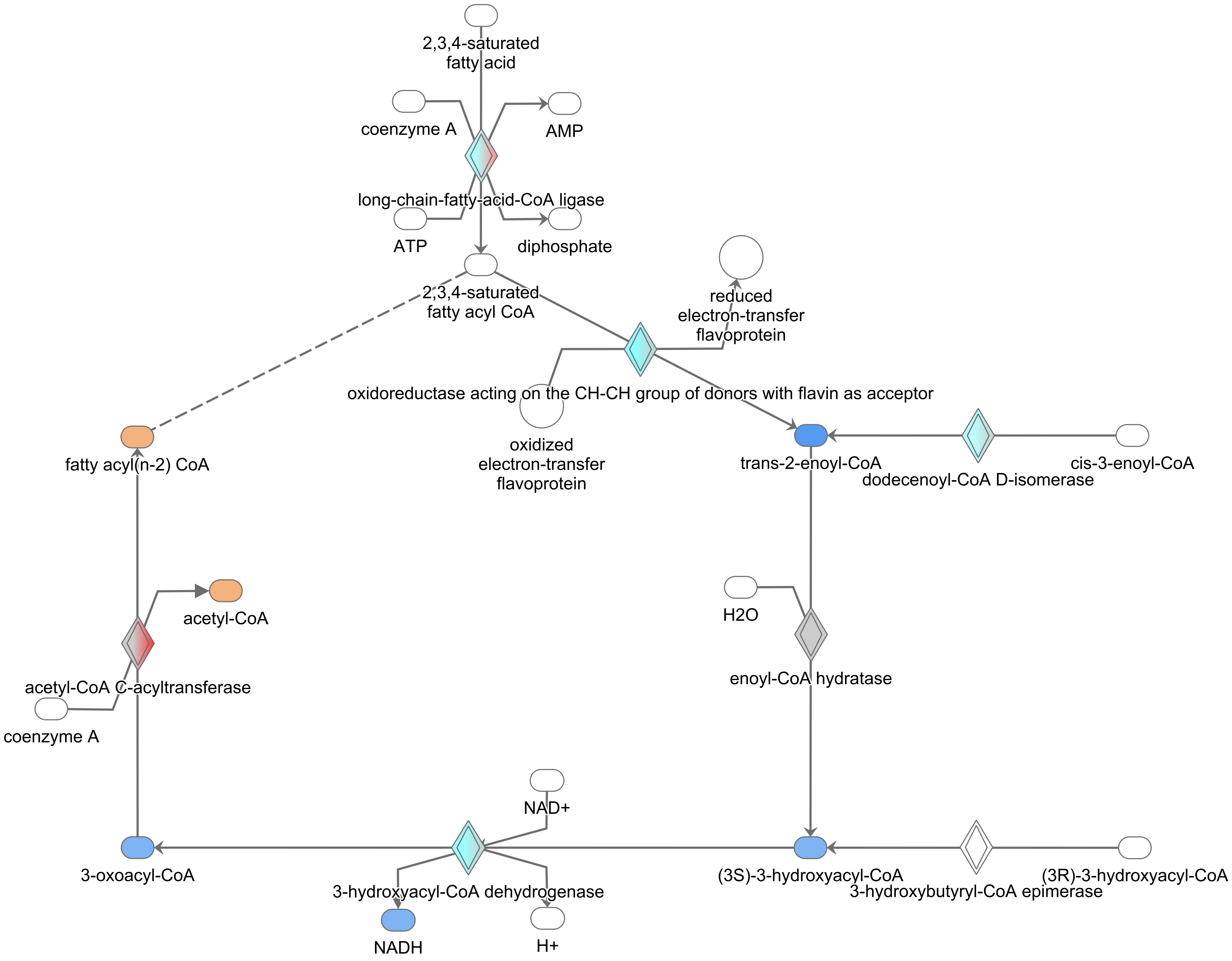

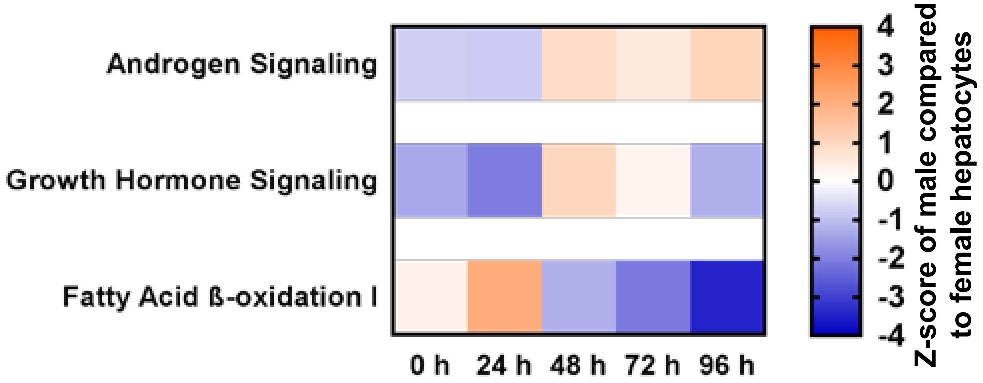

Supplement 5 - interactive pdf: Selected signaling pathways and their detailed dynamics during culture of primary hepatocytes of both sexes. The heatmap shows the activation Z-score analysis based on RNA array data of male compared to female hepatocytes. The activation Z-score was calculated with IPA software. The p-value cut off of 0.05 was used for calculation. A click on the colored squares reveals the detailed pathway analysis of the appropriate pathway of male compared to female hepatocytes during culture done by IPA. Nodes with color gradients represent complexes whose individual components are regulated differently. Lines symbolize direct interaction. Dashed lines symbolize indirect interaction.

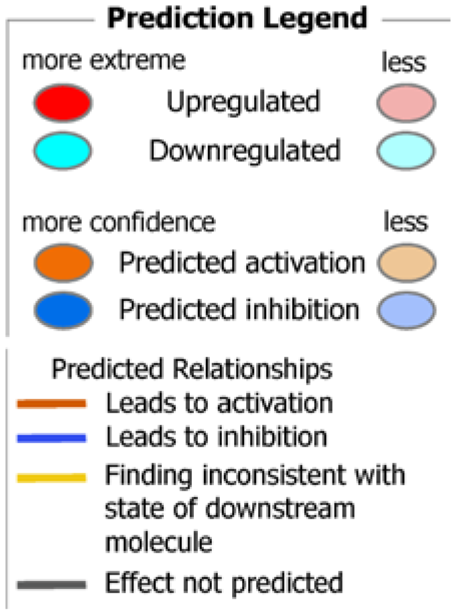

Fatty Acid β-oxidation 72 h

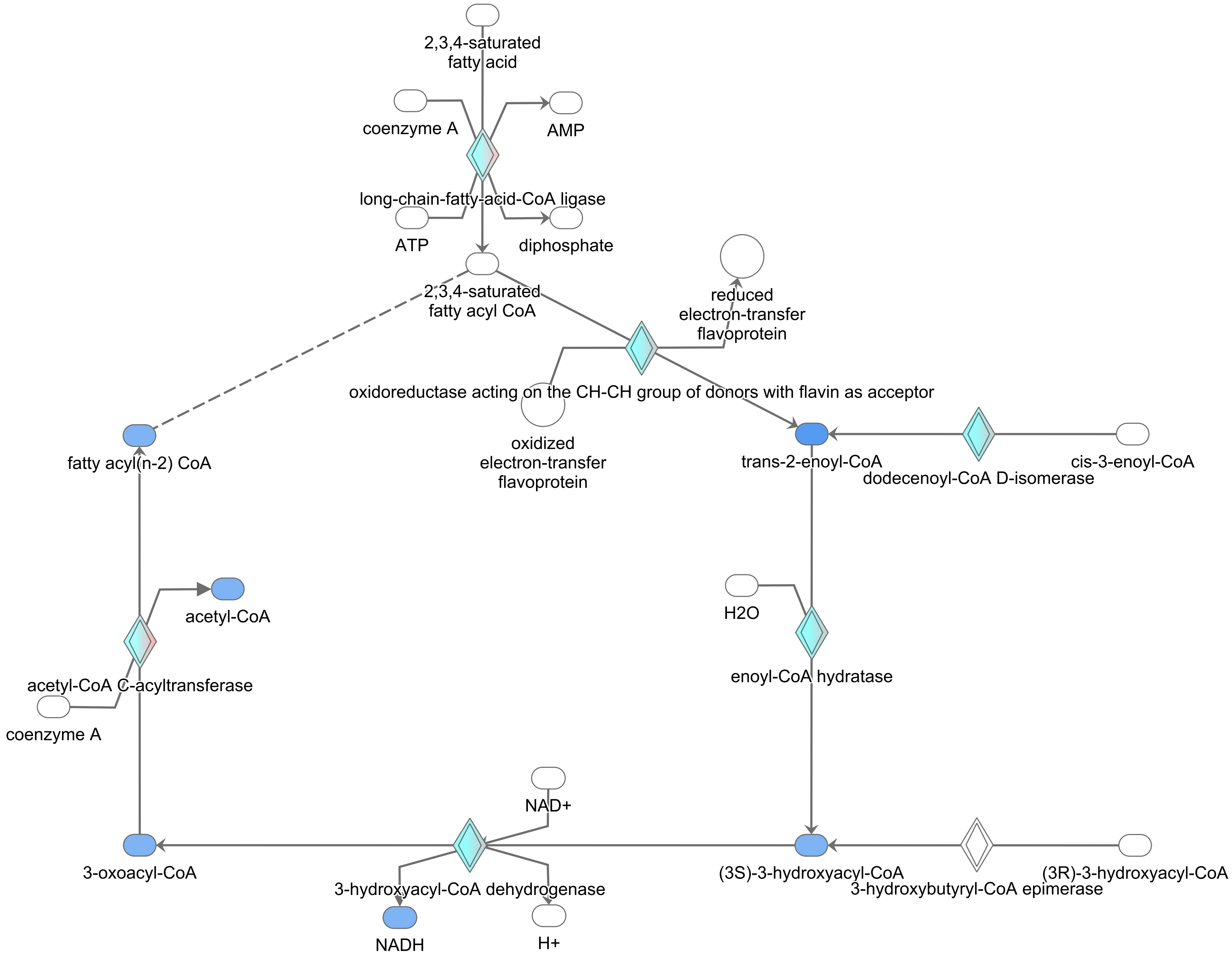

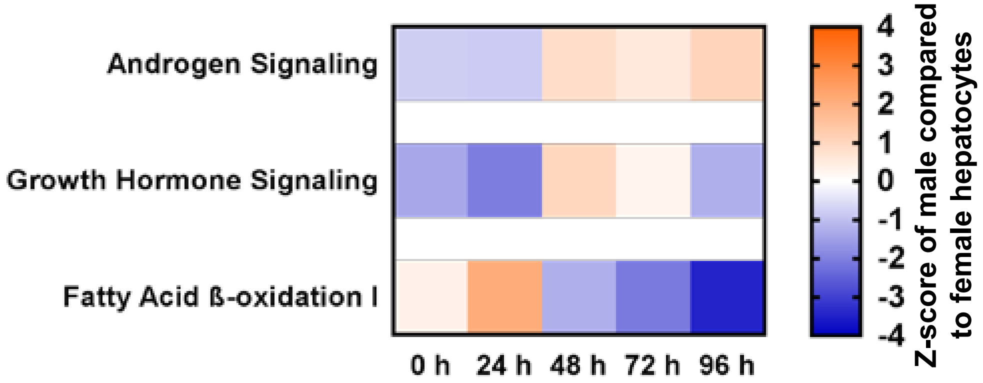

Supplement 5 - interactive pdf: Selected signaling pathways and their detailed dynamics during culture of primary hepatocytes of both sexes. The heatmap shows the activation Z-score analysis based on RNA array data of male compared to female hepatocytes. The activation Z-score was calculated with IPA software. The p-value cut off of 0.05 was used for calculation. A click on the colored squares reveals the detailed pathway analysis of the appropriate pathway of male compared to female hepatocytes during culture done by IPA. Nodes with color gradients represent complexes whose individual components are regulated differently. Lines symbolize direct interaction. Dashed lines symbolize indirect interaction.

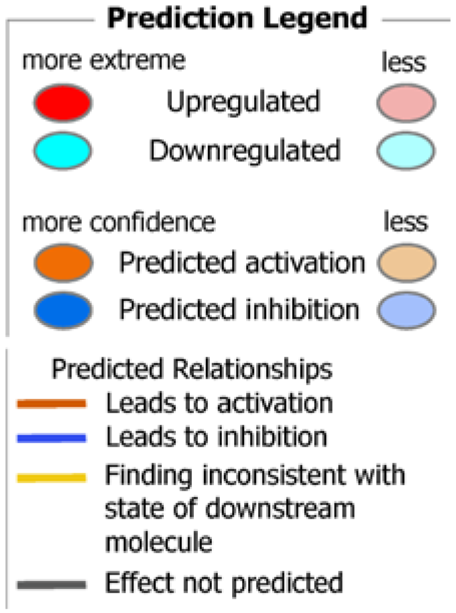

Fatty Acid β-oxidation 96 h

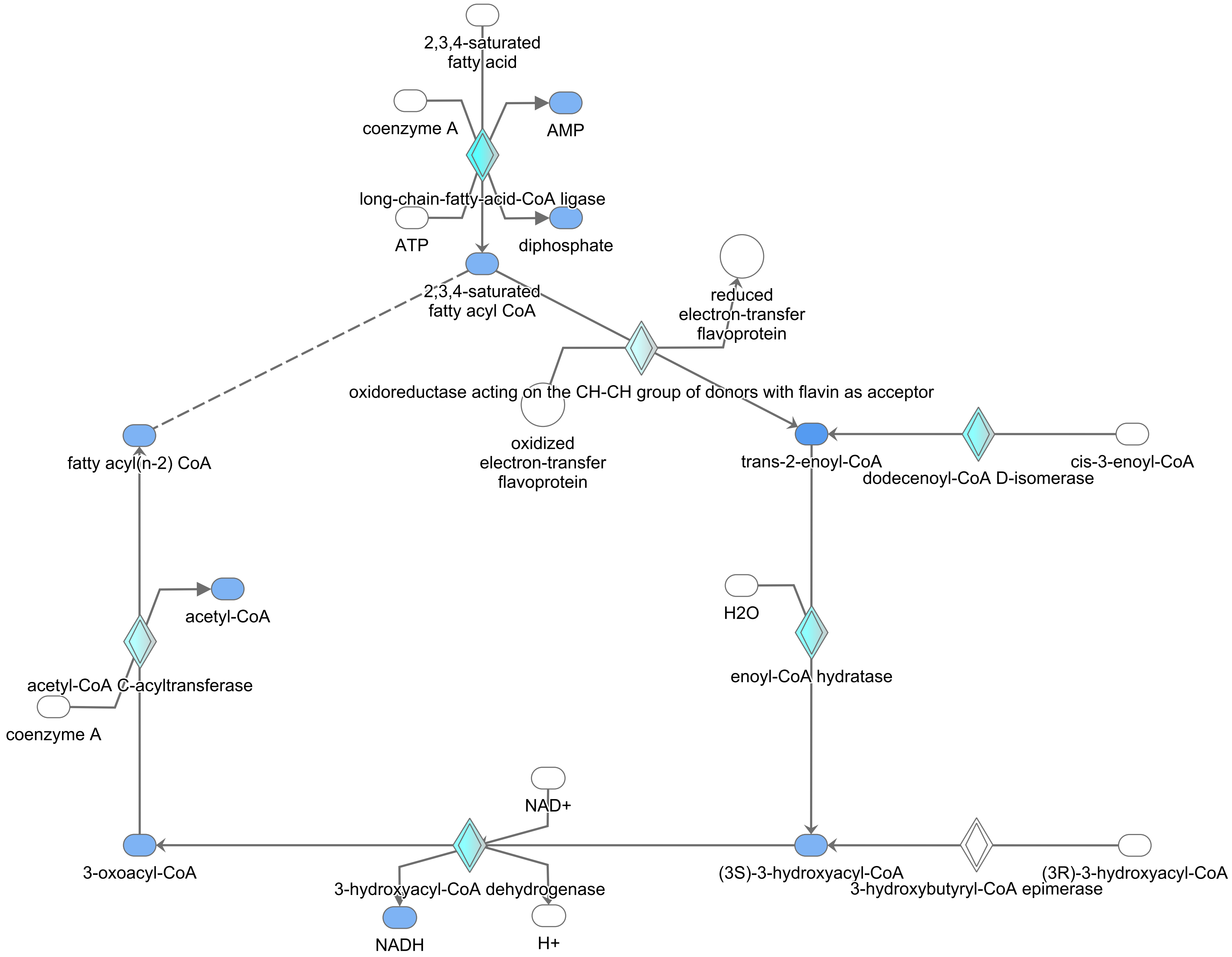

Supplement: Supplementary file 5 — Supplementary file5 (PDF 18016 kb) [file 204_2021_3118_MOESM5_ESM.pdf]
